# PNN Acetate Ruthenium(II) Complexes as Active Catalysts for Fatty Ester Hydrogenation

**DOI:** 10.1002/cssc.70979

**Published:** 2026-08-21

**Authors:** Maurizio Ballico, Tasnim Al‐Kassous, Lucas Carreras, Alexandra Heidecker, Jonathan Barrios Rivera, Sara Roudani, Walter Baratta

**Affiliations:** ^1^ Dipartimento di Scienze AgroAlimentari Ambientali e Animali (DI4A) Università di Udine Udine Italy; ^2^ Johnson Matthey Cambridge UK; ^3^ Catalysis Research Center Technical University of Munich Garching b. München Germany

**Keywords:** carboxylates, fatty esters, hydrogenation, reduction, ruthenium

## Abstract

The pincer acetate complexes *trans*‐[Ru(η^1^‐OCOCH_3_)_2_(PNN)(PPh_3_)] (PNN = N‐(2‐(diphenylphosphaneyl)benzyl)‐1‐(pyridin‐2‐yl)methanamine (PNN’) **1,** 2‐(diphenylphosphaneyl)‐N‐(pyridin‐2‐ylmethyl)ethan‐1‐amine (PNN^G^) **2**) are synthesized from [Ru(η^2^‐OCOCH_3_)_2_(PPh_3_)_2_] and the corresponding PNN ligands in acetone. Reaction of [RuH(η^2^‐OCOCH_3_)(PPh_3_)_3_] with the PNN ligands in *n*‐heptane gives *trans*‐[RuH(η^1^‐OCOCH_3_)(PNN)(PPh_3_)] (PNN = PNN’ **3**, PNN^G^ **4**). Substitution of PPh_3_ in **1** with PPh_2_Me gives *trans*‐[Ru(η^1^‐OAc)_2_(PNN’)(PPh_2_Me)] (**5**) in toluene, whereas starting from **2** results in *trans*‐[Ru(η^1^‐OAc)_2_(PNN^G^)(PPh_2_Me)] (**6**) and *cis*‐[Ru(η^2^‐OAc)(PNN^G^)(PPh_2_Me)]OAc (**7**) (in 1:1 ratio). The monocarbonyl [Ru(η^2^‐OAc)(CO)(PNN’)]OAc (**8**) and *cis*‐[Ru(η^1^‐OAc)_2_(CO)(PNN’)] (**9**) (in 3:1 ratio) are formed from [Ru(η^1^‐OAc)(η^2^‐OAc)(CO)(PPh_3_)_2_] with PNN’, whereas reaction of the latter precursor with 1‐(2‐(diphenylphosphaneyl)phenyl)‐N‐(2‐(pyridin‐2‐yl)ethyl)methanimine (PNN(C_2_)_imine_) affords *cis*‐[Ru(η^1^‐OAc)_2_(CO)(PNN(C_2_)_imine_)] (**10**). Acetate complexes **1**‐**9** catalyze the homogeneous hydrogenation of methyl decanoate to 1‐decanol with NaOMe under mild reaction conditions (T = 40 °C, H_2_ pressure 27.5 bar) and low catalyst loading (S/C 1000–100 000) in the absence of solvents. Complex **2** displays extremely high catalytic activity for the reduction of fatty acid methyl and ethyl esters at S/C of 50 000–100 000, with high chemoselectivity and without C=C bond reduction. In addition, **1**‐**4** and **8/9** are found to catalyze the base‐free hydrogenation of aldehydes. NMR studies show that **1** reacts with H_2_ (5 bar), affording the monohydride **3**, which, with KO^*t*^Bu and hydrogen, reduces methyl benzoate to benzyl alcohol.

## Graphical abstract

The acetate PNN ruthenium complexes efficiently catalyze the hydrogenation of methyl decanoate (model for the FAMEs reduction) to 1‐decanol without solvents and under mild reaction conditions. Selective hydrogenation of long‐chain and aromatic methyl‐ and ethyl esters has been achieved with *trans*‐[Ru(η^1^‐OAc)_2_(PNN^G^)(PPh_3_)] at extremely high S/C (up to 100 000).

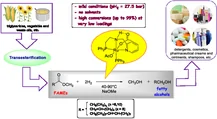

## 1 Introduction

The reduction of polar double bonds is a fundamental transformation in organic synthesis [1, 2], and great attention has been devoted to the catalytic homogeneous hydrogenation of carbonyl compounds, following the pioneering contributions of Noyori and coworkers, who demonstrated the pivotal role of the ligand “NH function” on Ru complexes [3]. Conversely, the hydrogenation of esters and amides, which show a lower electrophilic C=O bond [4], has been achieved later through the development of polydentate P, N, and S ligands. In this context, efficient catalysts have been designed using non‐innocent pincer ligands, affording robust catalytically active metal fragments and making them ideal platforms for a wide range of transition metal‐mediated reactions [5, 6, 7, 8, 9, 10, 11, 12, 13, 14, 15]. It is worth pointing out that the reduction of esters to alcohols is a process of crucial importance in both academia and industry, driven by the growing interest in converting of bio‐based raw materials, such as seeds and vegetable oils, into bulk chemicals and fatty alcohols (FAls) [16]. Thus, octyl, cetyl, and stearyl alcohols are sustainable key intermediates for the preparation of pharmaceuticals, emulsifiers, fragrances, lubricants, and detergents, with a production of multi‐million tons per year [17]. Conventional procedures entailing stoichiometric amounts of metal hydrides (i.e., LiAlH_4_, NaBH_4_, DIBAL‐H) involve a troublesome workup with a significant amount of waste [18]. On the other hand, catalytic hydrogenation with H_2_ is a highly atom‐economic and environmentally sustainable process carried out in industrial plants using heterogeneous catalysts based on Cu‐Cr and Ni, and also Ru, Rh, Pd, and Re systems [19, 20, 21, 22]. This process requires high H_2_ pressures and temperatures (*>*200 atm, *>*250 °C), and it is generally not chemoselective. By contrast, the use of homogeneous catalysts allows the reaction to occur under mild conditions and with higher chemoselectivities [23].

After the seminal work of Milstein in 2005 on the alcohol dehydrogenation to esters catalyzed by pincer PNN ruthenium complexes, great attention has been focused on the ester hydrogenation [24, 25, 26]. In this context, the derivatives [RuCl_2_(PN)_2_] [27, 28], “Ru‐MACHO” [RuHCl(CO)(PNP)] [29, 30, 31], [RuCl_2_(PPh_3_)(PNN)] [32, 33], [MHCl(CO)(PNN)] [34, 35, 36, 37] (M = Ru, Os), Gusev Catalyst [RuCl_2_(PPh_3_)(SNS)] [38], [RuCl_2_(PPh_3_)(SNN)] [39, 40] and the Ru‐CNC system [29, 41] allow the hydrogenation of a number of nonactivated esters under relatively mild conditions (T *≤* 120 °C and H_2_ pressure *≤* 50 bar). Besides, the trihydroquinoline flexible PNN system *fac*‐[RuH(PPh_3_)(CO)(PNN)], in combination with NaBH_4_, catalyzes the hydrogenation of esters to their corresponding alcohols at low loadings [42]. Interestingly, quinaldine‐based PNN Ru systems have been found to catalyze the direct alcoholysis of natural oils to fatty acids methyl esters (FAMEs) and hydrogenation to FAls employing a two‐phase solvent system [43].

Hydrogenation of polyesters has also been reported as an efficient method for hydrogenative depolymerization of polyesters, like polyethylene terephthalate (PET), into corresponding diols with Ru‐PNN complexes [44, 45, 46]. In addition, Ru‐PNN complexes have been shown to catalyze alcohols dehydrogenation, affording ketones [47], and esters via the Claisen–Tishchenko reaction [48, 49] and the coupling of ethylene glycol to primary amines leading to *N*‐substituted oxalamides [50]. Recently, our group has reported the isolation of carboxylate Ru‐SNS Gusev complexes, which are highly active and productive in the hydrogenation of FAME substrates at low H_2_ pressure and temperature [51]. It is worth pointing out that carboxylate ligands can stabilize the complexes through different coordination modes, easily interact with the protic media, facilitating exchange and activation reactions (i.e., H_2_ splitting), in comparison to more widespread chloride ligands.

Herein we report a straightforward preparation of acetate ruthenium complexes containing PNN pincer‐type ligands, obtained from accessible acetate ruthenium precursors. High activity and selectivity have been observed for the homogeneous hydrogenation of methyl and ethyl esters to alcohols, with FAME substrates easily reduced under mild conditions and with S/C up to 100 000, in absence of solvents.

## 2 Results and Discussion

### 2.1 Synthesis of PNN Acetate Ruthenium Complexes

**SCHEME 1:**
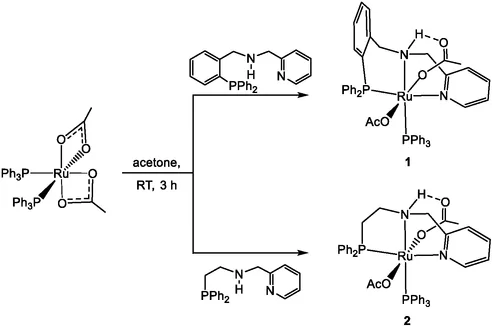
Synthesis of bis‐acetate PNN ruthenium complexes.

Treatment of [Ru(η^2^‐OAc)_2_(PPh_3_)_2_] with N‐(2‐(diphenylphosphaneyl)benzyl)‐1‐(pyridin‐2‐yl)methanamine (PNN’) [52] (1 equiv) in acetone at room temperature for 3 h afforded the neutral derivative *trans*‐[Ru(η^1^‐OAc)_2_(PNN’)(PPh_3_)] (**1**), as a dark‐yellow precipitate in 85% yield, by displacement of one of the triphenylphosphine ligands (Scheme 1).

Complex **1** can be also obtained in toluene, followed by precipitation with diethyl ether (80% yield). The ^31^P{^1^H NMR spectrum of **1** in the toluene‐*d* ^8^ shows two doublets at δ 46.6 and 38.5 ppm with ^2^ *J*(P,P) = 29.3 Hz, indicating a *cis* arrangement of the two phosphorus atoms. The ^1^H‐^31^P HMBC 2D NMR experiments revealed a *trans* PPh_3_ to N atom arrangement with a *mer* PNN’ ligand, according to the chloride *trans*‐[RuCl_2_(PNN’)(PPh_3_)] structure [52]. In addition, **1** displays an intramolecular NH ‐acetate hydrogen bond with a δ_H_ signal at 10.96 ppm and *δ*_C_ at 181.1 ppm, whereas the other acetate is at 177.6 ppm. The presence of the acetate ligands makes **1** highly soluble in several organic solvents, including toluene, CH_2_Cl_2_, alcohols. The derivative *trans*‐[Ru(η^1^‐OAc)_2_(PNN^G^)(PPh_3_)] (**2**) (81% yield) was synthesized following the procedure used for **1**, by reaction of [Ru(η^2^‐OAc)_2_(PPh_3_)_2_] with 2‐(diphenylphosphaneyl)‐N‐(pyridin‐2‐ylmethyl)ethan‐1‐amine [33] (PNN^G^ ligand) (Scheme 1) and displays a structure similar to **1** with two doublets at δ_P_ 53.8 and 47.6 ppm (^2^ *J*(P,P) = 29.3 Hz) and an NH moiety signal at δ_H_ 11.40 ppm.

**SCHEME 2:**
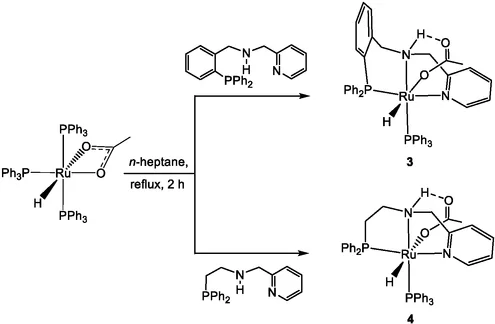
Synthesis of monohydride acetate PNN ruthenium complexes.

The ruthenium monohydride *trans*‐[RuH(η^1^‐OAc)(PNN’)(PPh_3_)] (**3**) was obtained in 76% yield by reaction of [RuH(η^2^‐OAc)(PPh_3_)_3_] with PNN’ (1 equiv) in *n*‐heptane at reflux for 2 h (Scheme 2).

The hydride ligand of **3** appears as a triplet at δ_H_ −18.62 ppm with ^2^ *J*(H,P) = 27.5 Hz, *cis* to the two phosphorus atoms and *trans* to the acetate, similarly to *trans*‐[RuHCl(PNN’)(PPh_3_)] [52]. ^1^H‐^1^H NOESY 2D NMR experiments in toluene‐*d* ^8^ show NOE interactions between Ru‐H and the phenyl protons of PPh_3_ (at δ 7.79 and 7.20 ppm) and with the CH_2_ signals at δ 4.19 and 3.95 ppm, but no exchange with the NH proton. A hydrogen bond between the NH and carbonyl of the acetate is present, as inferred from the upfield shifted NH at *δ*_H_ 10.12 ppm. Complex **3** is highly soluble in aromatic solvents and slowly reacts in CHCl_3,_ affording chlorinated species. Similarly to **3**, *trans*‐[RuH(η^1^‐OAc)(PNN^G^)(PPh_3_)] (**4**) was prepared by reaction of [RuH(η^2^‐OAc)(PPh_3_)_3_] with the PNN^G^ ligand and isolated in 71% yield (Scheme 2). The ^1^H NMR resonance of the Ru‐H is at *δ* −18.40 ppm (dd with ^2^ *J*(H,P) of 26.8 and 24.3 Hz, respectively), whereas the downfield‐shifted triplet at δ 10.62 ppm (^3^ *J*(H,H) = 8.7 Hz) is for the NH moiety.

**SCHEME 3:**
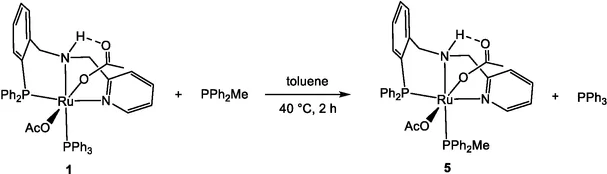
Synthesis of the bis‐acetate ruthenium PPh_2_Me complex **5**.

Treatment of **1** with PPh_2_Me (1.1 equiv) in toluene at 40 °C for 2 h affords *trans*‐[Ru(η^1^‐OAc)_2_(PNN’)(PPh_2_Me)] (**5**), isolated in 87% yield, by substitution of PPh_3_ (Scheme 3).

**SCHEME 4:**
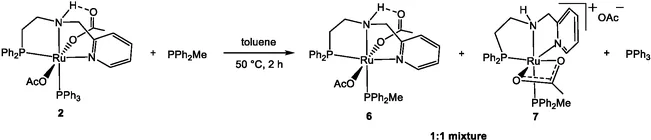
Synthesis of the 1:1 mixture of ruthenium PPh_2_Me complexes **6**/**7**.

In the ^31^P{^1^H} NMR spectrum, **5** displays two doublets at δ 48.8 and 28.1 ppm with ^2^ *J*(P,P) of 29.3 Hz, whereas the low‐field ^1^H NMR resonance at δ 10.92 ppm is for the NH, resulting in a hydrogen bond interaction with one acetate, as for **1**‐**4**. Conversely, reaction of **2** with PPh_2_Me in toluene at 50 °C for 2 h led to a 1:1 mixture of the *trans*‐[Ru(η^1^‐OAc)_2_(PNN^G^)(PPh_2_Me)] (**6**) and the cationic *cis*‐[Ru(η^2^‐OAc)(PNN^G^)(PPh_2_Me)]OAc (**7**) species, isolated in 84% yield (Scheme 4).

The ^31^P{^1^H} NMR spectrum of **6**/**7** in toluene‐*d* ^8^ displays two doublets at δ 67.0 and 30.9 ppm (^2^ *J*(P,P) = 29.3 Hz) for the neutral *trans* **6** and two doublets at δ 62.0 and 29.9 ppm (^2^ *J*(P,P) = 31.3 Hz) for the cationic *cis* **7** derivative in which the PNN^G^ ligand adopt a *fac*‐configuration with a chelate acetate. As in **1–4**, complex **6** shows a low‐field amino signal at δ_H_ at 11.14 ppm, for the intramolecular hydrogen bond interaction of NH with one acetate substituent.

**SCHEME 5:**
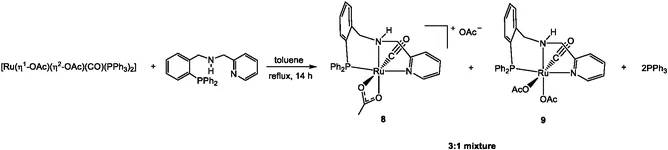
Synthesis of the PNN’ monocarbonyl complexes **8/9**.

Reaction of the monocarbonyl precursor [Ru(η^1^‐OAc)(η^2^‐OAc)(CO)(PPh_3_)_2_] with PNN’ in toluene at reflux for 14 h afforded a mixture of the two isomers [Ru(η^2^‐OAc)(CO)(PNN’)]OAc (**8**) and *cis*‐[Ru(η^1^‐OAc)_2_(CO)(PNN’)] (**9**) in a 3:1 molar ratio (70% yield) by displacement of the two PPh_3_ ligands (Scheme 5).

The ^31^P{^1^H} NMR spectrum in CD_3_OD shows two singlets at δ 42.5 and 55.1 ppm for the cationic **8** and the neutral **9** complexes, respectively, displaying a *mer* arrangement of the PNN’ ligand. For **8** the η^2^‐acetate ligand shows a *δ*_H_ at 1.48 ppm, whereas the OAc counterion is at δ_H_ 1.89 ppm. Instead, the two η^1^‐acetate methyl groups in **9** are at *δ*_H_ 2.05 and 1.36 ppm. The NH resonances of **8** and **9** are at *δ*_H_ = 10.16 and 8.95 ppm for **8** and **9**, respectively. In the ^13^C{^1^H} NMR spectrum, the signals for Ru‐CO of **8** and **9** appear as doublets at δ 205.5 (^2^ *J*(C,P) = 17.6 Hz) and 207.2 ppm (^2^ *J*(C,P) = 14.3 Hz), indicating a *cis* P‐Ru‐CO arrangement for the two isomers [53]. The presence of a weak NOE effect between the *ortho*‐pyridine proton at δ 8.83 ppm and the methyl group of the acetate bound to the ruthenium at δ 1.48 ppm in **8** is consistent with the CO and NH moieties *trans* to the η^2^‐acetate, as inferred from 2D ^1^H‐^1^H NOESY NMR experiments (Figure S63).

**SCHEME 6:**
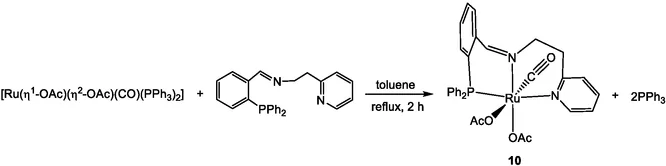
Synthesis of the neutral monocarbonyl derivative **10**.

The monocarbonyl complex *cis*‐[Ru(η^1^‐OAc)_2_(CO)(PNN(C_2_)_imine_)] (**10**) has been synthesized by reaction of the [Ru(η^1^‐OAc)(η^2^‐OAc)(CO)(PPh_3_)_2_] precursor with the imine ligand 1‐(2‐(diphenylphosphaneyl)phenyl)‐N‐(2‐(pyridin‐2‐yl)ethyl)methanimine [52, 54] (PNN(C_2_)_imine_) in a slight excess (1.05 equiv.) in toluene at reflux for 2 h in 87% yield (Scheme 6).

**FIGURE 1:**
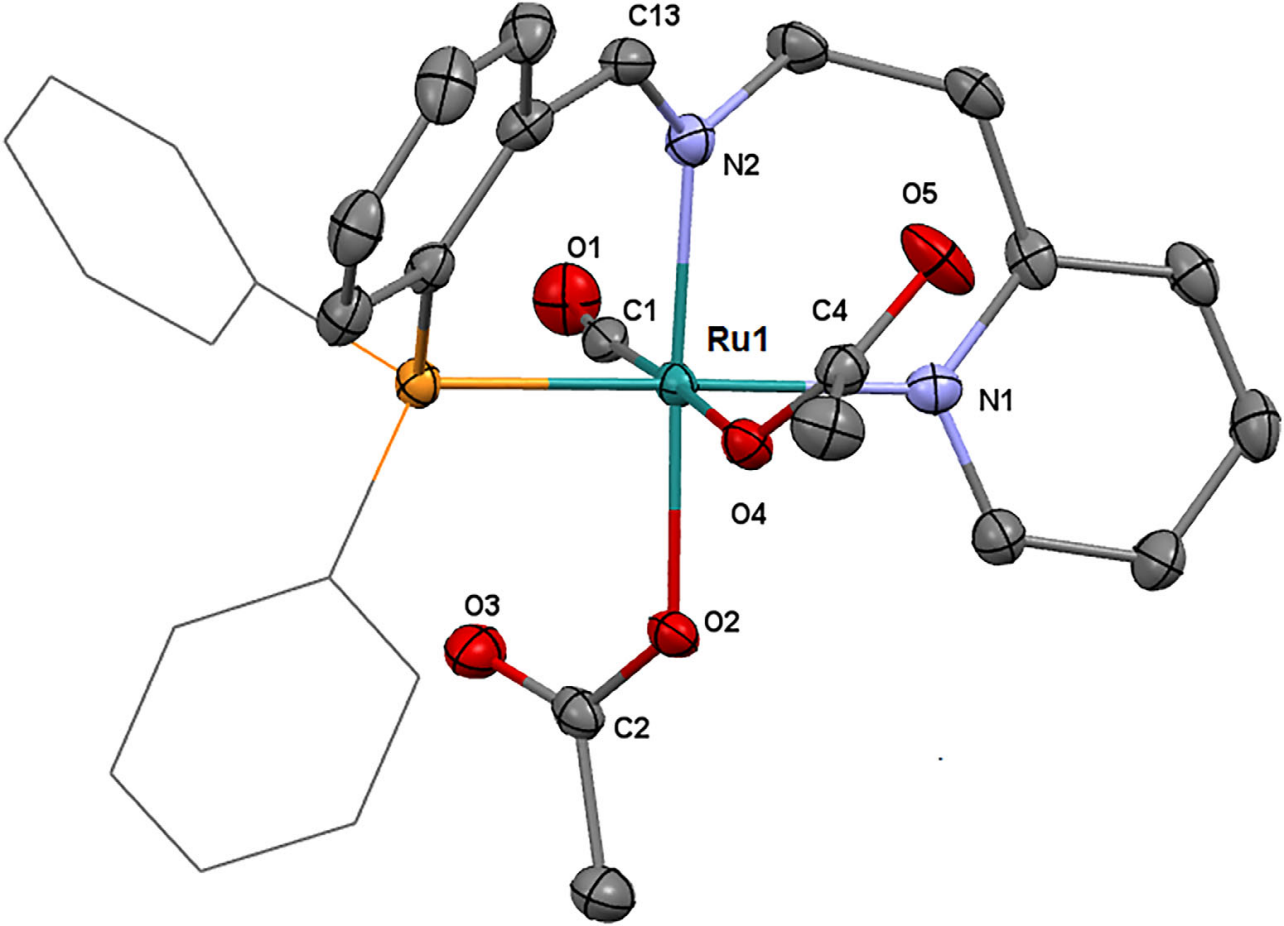
ORTEP representation of compound **10** in the solid state (CCDC 2 553 530). Ellipsoids are drawn at the 50% probability level. Hydrogen atoms and co‐crystallized solvent molecules are omitted, and phenyl groups are simplified as wireframes for clarity. Selected bond lengths [Å] and angles [°]: Ru1–C1 1.833(4), Ru1–O2 2.069(2), Ru1–N1 2.191(3), Ru1–N2 2.054(3), Ru1–O4 2.119(2), Ru1–P1 2.2796(10), O1‐C1 1.164(4), N2‐C13 1.282(5), C1‐Ru1‐N2 89.9(2), N2‐Ru1‐O2 174.4(1), N2‐Ru1‐O4 91.4(1), C1‐Ru1‐N1 94.3(1), O2‐Ru1‐N1 87.9(1), C1‐Ru1‐P1 92.6(1), O2‐Ru1‐P1 92.00(8), N1‐Ru1‐P1 173.06(8), C1‐Ru1‐O2 95.6(1), C1‐Ru1‐O4 177.15(1), O2‐Ru1‐O4 82.6(1), N2‐Ru1‐N1 90.6(1), O4‐Ru1‐N1 87.9(1), N2‐Ru1‐P1 88.77(9), O4‐Ru1‐P1 85.20(8), C13‐N2‐Ru1 128.2(3), O1‐C1‐Ru1 174.6(3).

The employment of a more rigid and longer chain PNN ligand leads to the formation of the neutral derivative **10**, showing a *mer* tridentate ligand and two *cis* acetates, with a different configuration with respect to the related chloride *mer*,*trans*‐[RuCl_2_(CO)(PNN(C_2_)_imine_)] [55]. The resonance of the *H*C=N proton is at *δ*_H_ 8.40 ppm, whereas Ru‐CO and the imine carbons appear as doublets at δ_C_ 204.6 (^2^ *J*(CP) = 16.9 Hz) and 168.0 ppm (^3^ *J*(C,P) = 5.9 Hz), respectively. The molecular structure of **10** was definitively confirmed by an X‐ray diffraction analysis carried out on a single crystal, showing a ruthenium center in a pseudo‐octahedral environment (Figure 1).

The Ru‐N1 distance is longer compared to that of the bond Ru‐N2 (2.191(3) versus 2.054(3) Å) due to the higher *trans* influence of the phosphine. The N2‐Ru1‐O2 and N1‐Ru1‐P1 angles are 174.4(1)° and 173.06(8)°, respectively, and the chelate rings are not strained (N2‐Ru1‐P1 88.77(9)° and N2‐Ru1‐N1 90.6(1)°) as expected for six‐membered rings. The Ru‐C‐O moiety is essentially linear (174.6(3)°), while the Ru1‐C1 distance (1.833(4) Å) is relatively short and in line with other ruthenium carbonyl complexes [55, 56]. Finally, the N2‐C13 bond length is for the imino group (1.282(5) Å), consistent with related Ru imino derivatives [57].

### 2.2 Hydrogenation of Esters Catalyzed by Acetate PNN Ruthenium Complexes

**SCHEME 7:**
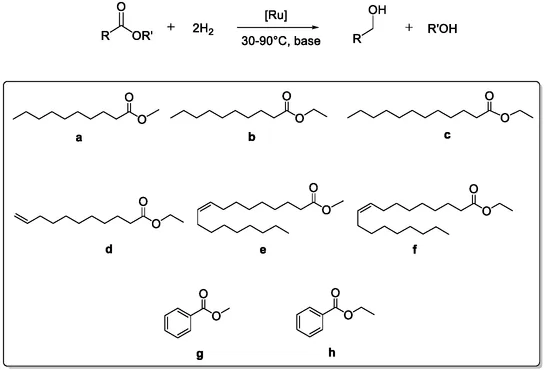
Hydrogenation of methyl and ethyl esters catalyzed by complexes **1–** **9**.

The catalytic activity of the acetate **1**‐**9** has been examined on the hydrogenation of methyl and ethyl esters under mild H_2_ pressure (27.5 bar) with substrate/catalyst (S/C) ratios ranging between 10 000 and 100 000, neat or in solution, with a temperature range between 30 and 90 °C (Scheme 7).

In addition, their activity has been compared with that of the related chloride derivatives *trans*‐[RuCl_2_(PNN’)(PPh_3_)] [58] and *trans*‐[RuCl_2_(PNN^G^)(PPh_3_)] [33]. It is worth noting that methyl esters are the standard substrates in industry, although ethyl esters display generally a better conversion with respect to methyl esters, because of the inhibiting effect of the methanol by‐product [20, 59]. The hydrogenation of methyl decanoate **a** into 1‐decanol and MeOH is a good model for the reduction of fatty acid methyl esters (FAME), and it has been carried out with catalysts **1–** **9** without addition of solvent and in the presence of NaOMe 50 mol% at 40 and 90 °C (Table 1).

**TABLE 1:** Catalytic hydrogenation of methyl decanoate a into 1‐decanol with **1–9**, *trans*‐[RuCl_2_(PNN’)(PPh_3_)] and *trans*‐[RuCl_2_(PNN^G^)(PPh_3_)] (S/C = 1000–50 000), NaOMe 50 mol% at 27.5 bar of H_2_ at 40 and 90 °C, neat, 16 h.

Entry	Complex	S/C	T, °C	Total Conv.^a^, %	Conv. to 1‐decanol^a^, **%**
1	**1**	10 000	40	87	79
2	**1**	10 000	90	98	98
3	**2**	10 000	40	100	100
4	**2**	10 000	90	83	75
5	**2**	50 000	40	98	98
6	**3**	10 000	40	31	9
7	**3**	10 000	90	49	30
8	**4**	10 000	40	94	89
9	**4**	10 000	90	70	54
10	**5**	10 000	40	89	83
11	**5**	10 000	90	92	87
12	**6/7**	10 000	40	100	100
13	**6/7**	10 000	90	82	71
14	**8/9** b	1000	90	85	79
15	**8/9** c	1000	90	99	99
16	[RuCl_2_(PNN’)(PPh_3_)]	10 000	40	7	0
17	[RuCl_2_(PNN^G^)(PPh_3_)]	10 000	40	99	97

a Determined by GC analyses.

b Using MeTHF as solvent.

c Using EtOH as solvent.

At S/C = 10 000, full conversion is achieved after 16 h at 40 °C with the PPh_3_‐substituted complex **2** and the PPh_2_Me‐derived mixture of **6**/**7** (entries 3 and 12 of Table 1) that contain the PNN^G^ ligand, while the PNN’ derivatives **1** and **5** give the same result at 90 °C (entries 2 and 11). On the other hand, the carbonyl **8/9** mixture affords 99% conv. at S/C 1000 and 90 °C (entry 15), but shows poor activity at S/C 10 000 (8% conv., entry 17 of Table S1). The same behavior has been observed for the imino complex **10** (5% total conv. at S/C 10 000, entry 21 of Table S1). When the conversion is not complete, the presence of trans‐esterification products is observed in addition to the expected alcohols. Reducing the catalyst loading (S/C 50 000), high conversion is observed for **2** (98%, entry 5). It is worth noting that with 30 mol% of NaOMe and S/**2** 50 000, a lower conversion to alcohol is obtained (71%), and no reaction takes place without base, in agreement with the studies on the *trans*‐[RuCl_2_(PNS)(PPh_3_)] system [60]. The presence of the electron‐donating PPh_2_Me in **5** and **6**/**7** leads to less active and more stable complexes compared to the PPh_3_ derivatives **1** and **2**, thus preventing the release of phosphine that has been observed when these compounds are reacted with strong bases and H_2_. Interestingly, by increasing the temperature to 90 °C, the PNN’ derivatives display an enhanced activity at S/C 10 000, indicating a higher stability of the active dihydride species (entries 2, 7, 11), while the PNN^G^ complexes lead to incomplete hydrogenation (entries 4, 9, and 13). Thus, the PNN^G^ monohydride **4** leads to 89% conv. of 1‐decanol at S/C = 10 000 (entry 8), whereas 19% total conversion is attained at S/C = 50 000 (entry 7 of Table S1). The lower activity of **4** compared to the diacetate **2** can be ascribed to the higher air and moisture sensitivity of **4,** and the same trend was observed for the RuPNN’ hydride **3** (entry 5 of Table S1).

Catalyst **2** has been tested in the hydrogenation of several methyl and ethyl esters, using NaOMe and NaOEt, respectively, to avoid undesired transesterification reactions (Scheme 7; Tables 2 and S2).

**TABLE 2:** Catalytic hydrogenation of esters into alcohols with 2 with base (50 mol%) at 27.5 bar of H_2_, neat, 16 h.

Entry	Substrate	S/C	Base	T, °C	Total Conv.^a^, %	Conv. to alcohol^a^, %
1	**b**	50 000	NaOEt	40	79^b^	68^b^
2		100 000	NaOEt	40	97	97
3	**c**	44 000	NaOEt	40	99	98
4	**d**	10 000	NaOEt	30	91	91
5	**e** c	25 000	NaOMe	60	100	100
6		50 000	NaOMe	50	80	80
7	**f** c	50 000	NaOEt	40	97	97
8		100 000	NaOEt	40	97	97
9	**g**	10 000	NaOEt	40	96	96
10		25 000	NaOEt	40	82	79
11		50 000	NaOEt	40	72	67
12		100 000	NaOEt	40	66	57
13	**h**	100 000	NaOEt	40	98	98

a Determined by GC analysis.

b Using toluene as solvent.

c Substrates **e** and **f** were of technical grade and contain methyl or ethyl oleate (>76‐80%) but also other polyunsaturated (methyl or ethyl linoleate and linolenate) and saturated esters (methyl or ethyl palmitate and stearate): the reported conversions are referred to the hydrogenation of the real methyl or ethyl oleate present in the starting material, although the reduction of the other esters to the respective alcohols (linoleyl alcohol, palmityl alcohol, etc.) has been also observed (see ESI).

With this catalyst, the hydrogenation of ethyl decanoate **b** occurs more easily compared to methyl substrate **a**. Thus, complex **2** hydrogenates **b** to 1‐decanol with 68% conv. at S/C 50 000 in toluene and reduces almost quantitatively **b** at 40 °C with S/C 100 000 in neat conditions in 16 h (entries 1–2 of Table 2), while [RuCl_2_(PNN^G^)(PPh_3_)] gives 87% total conv. (entry 1 of Table S2). Ethyl dodecanoate **c** is completely hydrogenated to the corresponding alcohol by **2** at S/C of 44 000, without solvent, at 40 °C (entry 3). Employing **2**, the unsaturated ethyl 10‐undecenoate **d** gives 91% conversion with high selectivity for 10‐undecen‐1‐ol at S/C 10 000 and without remarkable hydrogenation or isomerization of the C=C bond (< 1% of 1‐undecanol; entry 4) [39]. Methyl oleate **e** is quantitatively reduced with **2** to (*Z*)‐octadec‐9‐en‐1‐ol with preservation of the C=C bond at S/C 25 000 at 60 °C (entry 5), whereas the conversion drops to 80% at S/C 50 000 at 50 °C (entry 6). Ethyl oleate **f** is more easily hydrogenated and affords the oleyl alcohol at a lower temperature (40 °C) at S/C 50 000–100 000 with **2** (entries 7 and 8) and, with similar results, the related chloride derivative (entries 9–10 of Table S2). Finally, **2** affords 96 to 57% conv. of methyl benzoate **g** at S/C 10 000–100 000 using NaOEt that increases the activity via transesterification (entries 9–12). The ethyl substrate **h** was completely hydrogenated to benzylic alcohol at S/C 100 000 at 40 °C by **2** in the absence of solvents (entry 13).

These results indicate that Ru PNN acetate complexes are highly active catalysts for the hydrogenation of several methyl and ethyl esters, and the acetate complex **2** displays the best activity, achieving S/C 10 000–50 000 in the hydrogenation of FAMEs. High selectivity was always obtained for the hydrogenation of the unsaturated esters with retention of the C=C bond.

### 2.3 Base‐Free Hydrogenation of Aldehydes Catalyzed by Acetate PNN Ruthenium Complexes

**SCHEME 8:**
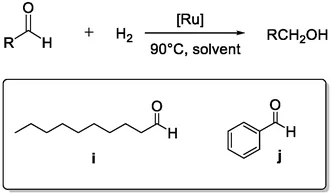
Base‐free hydrogenation of aldehydes catalyzed by complexes **1–** **4**, **8/9**.

On the basis of the efficiency of the acetate Ru complexes in the hydrogenation of ester substrates, a series of experiments were carried out to probe their performance also in the hydrogenation of aldehydes conducted in the same conditions of H_2_ pressure (27.5 bar), temperature (90 °C), and time (16 h), using different alcohols as solvents, with S/C loadings ranging between 1000 and 5000, but in the absence of base due to the more reactive nature of aldehydes in the presence of these catalysts (Scheme 8).

Base‐free conditions avoid undesired base‐catalyzed aldol condensation side reactions and the limits imposed by the tolerance of functional groups [61]. Catalysts **1**‐**4** have proven to quantitatively hydrogenate 1‐decanal **i** to 1‐decanol at S/C 1000 in alcohol media. The PNN’ complex **1** has given a conversion for **i** of 89, 100, and 93% in MeOH, EtOH, and *i*PrOH, respectively, after 16 h (entries 1–3 of Table 3). Quantitative hydrogenation has also been achieved with **2**, **3**, **4,** and **8**/**9** in ethanol, while *trans*‐[RuCl_2_(PNN’)(PPh_3_)] gives 42% conv. under the same conditions (entries 4–7 and 21 of Table S3 in ESI). Related results have been obtained using different solvents (Table S3).

**TABLE 3:** Base‐free catalytic hydrogenation of aldehydes with 1‐4 and 8/9 (S/C = 1000) at 27.5 bar of H_2_, 90 °C after 16 h.

Entry	Complex	Substrate	Solvent	**Conv.** a **, %**
1	**1**	**i**	MeOH	89
2	**1**		EtOH	100
3	**1**		2‐propanol	93
4	**2**		EtOH	96
5	**3**		EtOH	100
6	**4**		EtOH	99
7	**8/9**		EtOH	93
8	**2**	**j**	MeOH	80
9	**4**		MeOH	82

a Determined by GC analyses.

Complexes **2** and **4** have also been tested in the hydrogenation of benzaldehyde **j** in MeOH, affording benzylic alcohol in 80 and 82%, respectively (entries 8 and 9).

### 2.4 Hydrogen Uptake During Catalysis and NMR Studies

**FIGURE 2:**
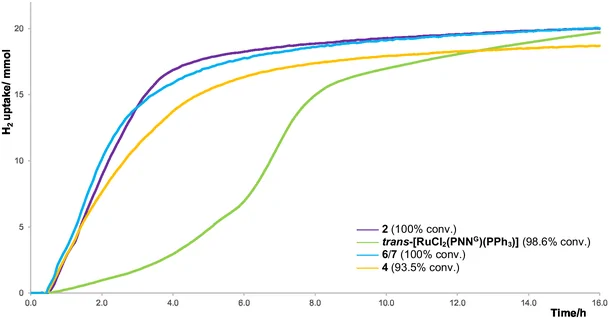
Curves of the hydrogen uptake for the hydrogenation of **a** with complexes **2**, **4**, and **6/7** and *trans*‐[RuCl_2_(PNN^G^)(PPh_3_)] (S/C = 10 000) at 40 °C, 27.5 bar of H_2_ and 50 mol% NaOMe.

To shed light on the steps involved in the ester hydrogenation, H_2_ uptake measurements and NMR studies have been carried out with the PNN^G^ complexes (Tables 1 and S1). The curve of hydrogen consumption for the hydrogenation of **a** with **2**, **4**, and **6/7** at S/C 10 000 is reported in Figure 2.

The acetate complexes **2**, **4**, and **6/7**, containing PPh_3_ and PPh_2_Me, show a short induction period compared to the *trans*‐[RuCl_2_(PNN^G^)(PPh_3_)], indicating that the carboxylates are more efficient, as they are activated more readily and provide quantitative hydrogenation in a shorter time. Under these conditions, monohydride **4** shows rapid activation, considering that traces of oxygen and water can easily give deactivation. Conversely, at 90 °C, the induction times of the acetate **2**, **4**, and *trans*‐[RuCl_2_(PNN^G^)(PPh_3_)] are more uniform (Figure S83), while at lower catalyst loading (S/C 50 000), incomplete hydrogenation is observed for the hydride **4** and for **6**/**7** (Figure S84). This behavior has also been observed with other substrates, with the methyl esters requiring longer reaction times with respect to the analogous ethyl derivatives. Thus, methyl oleate requires higher temperatures (50–60 °C) to achieve quantitative conversion (see Figures S88–S102).

**SCHEME 9:**
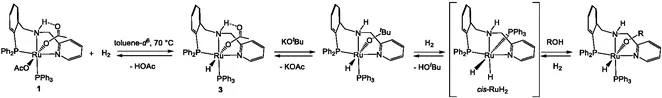
Equilibria among complex **1**, *trans*‐[RuH(X)(PNN’)(PPh_3_)], [RuH(PNN’)(PPh_3_)_2_]^+^X^−^ (X = OAc, O^*t*^Bu, OR) species.

NMR experiments have been conducted to investigate the formation of hydride ruthenium from the acetate pre‐catalysts during the ester hydrogenation. Reaction of **2** with H_2_ (5 bar) in toluene‐*d* ^8^ at 50 °C does not give the formation of new products. By contrast, addition of KO^*t*^Bu (3 equiv.) leads to complete conversion of **2** after 4 h into uncharacterized RuH species in low amounts (multiplet at δ_H_ = −16.6 ppm) in addition to releasing PPh_3_ and Ph_3_P=O (Figures S73‐S74). Conversely, treatment of **1** without base affords the monohydride **3** in small amounts (Scheme 9).

Addition of KO^*t*^Bu (3 equiv.) to **1** under H_2_ at 50 °C gives after 18 h a mixture of several monohydride species, namely *trans*‐[RuH(X)(PNN’)(PPh_3_)] (X = OAc, O^*t*^Bu) (Scheme 9), in addition to [RuH(PNN’)(PPh_3_)_2_]^+^X^‐^ (in traces) and [RuH(X)(PNN’)] species with PPh_3_ and Ph_3_P=O, without apparent formation of a dihydride complex [62], as inferred from ^1^H‐^1^H COSY 2D NMR experiments (Figures S75‐S78). At higher temperature (70 °C) and longer reaction times (24–48 h), PPh_3_ and Ph_3_P=O proportion increases. In addition, the monohydride complexes show signals at *δ*_P_ = 60–70 ppm and at *δ*_H_ = −3.9÷−22.9 ppm. In the ^1^H NMR spectra, the low‐field signals at *δ* = 17.5–13.5 ppm have been attributed to the formation of a low amount of Ru‐imine *H*C=N protons that may originate from the CH_2_NH moiety of the PNN’ ligand in the basic media (Figure S78).

Addition of **g** (5 equiv) to the mixture of RuH species obtained from **1** under H_2_ atmosphere (5 bar) and in the presence of KO^*t*^Bu (3 equiv) leads to a partial formation (50% conversion) of benzylic alcohol and MeOH at 70 °C in 16 h (Figure S79). This result confirms that this hydride mixture is catalytically active in ester hydrogenation, even if a dihydride species has not been detected [38]. In addition, no appreciable hydrogenation of **g** has been observed without KO^*t*^Bu, used due to its relative stability and convenience since its NMR signals do not overlap with those of the species under examination. Similar species observed for **1** have been detected starting from the monohydride **3** under H_2_ (5 bar) in toluene‐*d* ^8^ with KO^*t*^Bu (3 equiv.) at 50 °C after 16 h, affording several monohydride species (Figure S80). The only difference was a greater formation of the cationic compound [RuH(PNN’)(PPh_3_)_2_]^+^ (about 5%) characterized by the triplet at δ_P_ = 64.2 ppm and the doublet at δ_P_ = 51.9 ppm with ^2^ *J*(P,P) = 31.6 Hz and a quartet at δ_H_ = −10.74 ppm with ^2^ *J*(H,P) = 22.3 Hz, formed by reaction of **3** with PPh_3_ via decomposition of the RuH derivatives (Figures S81‐S82).

### 2.5 Proposed Mechanism for Hydrogenation of Esters

**SCHEME 10:**
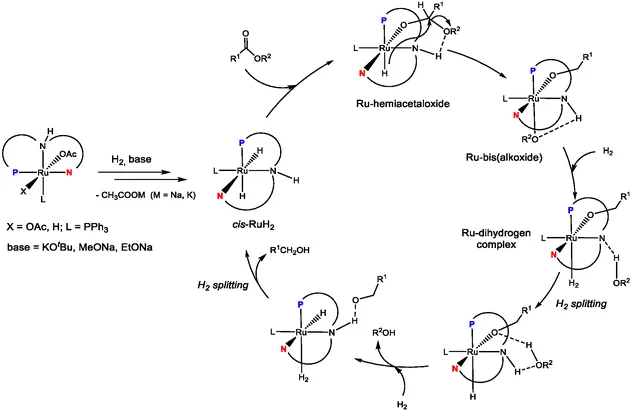
Proposed mechanism for ester hydrogenation catalyzed by the ruthenium acetate PNN complexes.

Based on these results, a mechanism for the catalytic hydrogenation of esters with **1**‐**6/7** is proposed, in agreement with Gusev's investigations on Ru PNN^G^ system and our studies on carboxylate Ru SNS complexes [38, 51]. Reaction of the acetate PNN complex with H_2_ in the presence of a strong base leads to a catalytically active dihydride species, in equilibrium with monohydride species (see Scheme 9), that reacts with the ester substrate to generate a hemiacetaloxide complex (Scheme 10).

Intramolecular nucleophilic substitution of the hydride leads to a Ru‐bis(alkoxide) containing an NH function, which reacts with H_2,_ affording a dihydrogen complex that forms a RuH(alkoxide) species via heterolytic H_2_ splitting and elimination of an alcohol product. Addition of a second H_2_ molecule leads to the dihydride complex, which closes the cycle with extrusion of the second alcohol product (Scheme 10). H_2_ activation is facilitated by the presence of the NH group [63, 64, 65] that interacts with the Ru‐alkoxide via a hydrogen bond, in agreement with the NMR data of **1**‐**6**/**7**, enhancing the reactivity. In addition, the elimination of monophosphine is detrimental for catalysis, leading to the decomposition of the hydride species.

NMR experiments have revealed that the mixture of monohydride species with H_2_ and base can reduce esters, possibly through a catalytically active RuH_2_ derivative, as reported by Gusev for *trans*‐[RuCl_2_(PNN^G^)(PPh_3_)] [61]. Moreover, the hydride species are easily protonated by alcohols, and therefore RuH(alkoxide) can be easily formed with the more acidic methanol versus ethanol, in agreement with the low activity of the methyl esters. The employment of strong bases in excess (NaOMe, NaOEt, KO^*t*^Bu) has a favorable effect on the ester hydrogenation, by reducing traces of H_2_O and carboxylic acids present in commercial esters and lowering the acidity of the alcohol products [60].

The NMR analysis of the hydrogenation reaction with the acetate complexes gave no evidence of the presumed hemiacetal or aldehyde intermediates of ester hydrogenation, as suggested by Milstein [66] and Saudan [28]. On the other hand, the isolation of a Ru‐hemiacetaloxide intermediate at low temperatures has been described by Bergens [67], using lactones, and also observed by Pidko [41] and Gusev [36] through the use of ESI‐MS techniques, whereas the preparation of fluoral hemiacetals from polyfluorinated esters have been described by Dub [60]. The NMR and H_2_ uptake experiments indicate that the generation of the active species occurs much more rapidly with the acetate versus the chloride derivatives, making these novel acetate PNN Ru complexes ideal for both catalytic applications in ester hydrogenation on account of their fast activation and robustness and for the mechanistic investigations due to their high solubility in hydrocarbon solvents and fast H_2_ activation.

The comparison of the catalytic activity of this class of complexes indicates that for the system [RuH_2_(L)(PNN)] the best performances are achieved with L = PPh_3_ and PPh_2_Me, while low efficiency has been observed with the π‐acceptor L = CO. In addition, the use of the imino ligand with a longer backbone between the two N atoms gives the non‐active system **10** [52]. Regarding aldehyde hydrogenation, which is an easier process with respect to ester hydrogenation, the monohydride ruthenium species, simply generated from the acetate PNN complexes with H_2_ and without base, can catalyze this reduction, as proposed by Dupau for Ru carboxylate catalysts [68]. The PNN derivatives that contain different phosphines and carbonyl ligands show a similar activity.

## 3 Conclusions

In summary, we have described easily accessible new acetate pincer ruthenium complexes from ruthenium acetate phosphine precursors and PNN ligands. These derivatives display high activity and chemoselectivity for the hydrogenation of esters to alcohols under mild reaction conditions. Thus, several methyl and ethyl esters and in particular FAMEs are easily and selectively hydrogenated in neat conditions at the C=O bond and without C=C reduction, under H_2_ at 27.5 bar and with a remarkably high S/C (up to 100 000). Short induction time indicates the beneficial effect of the Ru‐acetate catalysts versus the corresponding chloride complexes. In addition, hydrogenation of aldehydes without base has been observed with these complexes. Monohydride acetate complexes have also been observed in the reaction of the Ru diacetate catalysts with H_2_ in the presence of a base Ru, affording the hydrogenation of methyl benzoate. Studies are ongoing to acquire more evidence of the nature of the hydride species involved in the mechanism of the ester hydrogenation catalyzed by PNN acetate derivatives, including the use of these complexes in the hydrogenation of other C=O containing sustainable substrates.

## Funding

This study was supported by DM 1062/2021 PON 2014‐2020 Action IV.6 Green and DM737/2021 by NextGenerationEU.

## Conflicts of Interest

The authors declare no conflicts of interest.

## Supporting information

All the experimental procedures and characterization details of the synthesized compounds including NMR spectra, X‐ray crystallographic details of complex **10** and further data for the hydrogenation of esters catalyzed by the ruthenium derivatives are provided in the Supporting Information. The authors have cited additional references within the Supporting Information [69−86].

## Data Availability

The data that support the findings of this study are available from the corresponding author upon reasonable request and in the Supporting Information of this article.
