# Supplementary material for: PNN Acetate Ruthenium(II) Complexes as Active Catalysts for Fatty Ester Hydrogenation
Source: ChemSusChem. 2026 Aug 21;19(16):e70979. doi: 10.1002/cssc.70979 (PMC13496080; doi:10.1002/cssc.70979)
Supplement: Supplementary file 1 [file CSSC-19-e70979-s001.pdf]

ChemSusChem

PNN Acetate Ruthenium(II) Complexes as Active Catalysts for Fatty Ester Hydrogenation

Maurizio Ballico, Tasnim Al-Kassous, Lucas Carreras, Alexandra Heidecker, Jonathan Barrios Rivera, Sara Roudani, and Walter Baratta

Supporting Information

Table of Contents:

Experimental section Pag. S14

Materials and methods Pag. S14

Synthesis of *trans*-[Ru(η^1-OAc)$_2$(PNN')(PPh$_3$)] (1). *Method 1* Pag. S14

Synthesis of *trans*-[Ru(η^1-OAc)$_2$(PNN')(PPh$_3$)] (1). *Method 2* Pag. S15

Synthesis of *trans*-[Ru(η^1-OAc)$_2$(PNN^G)(PPh$_3$)] (2) Pag. S15

Synthesis of *trans*-[RuH(η^1-OAc)(PNN')(PPh$_3$)] (3) Pag. S15

Synthesis of *trans*-[RuH(η^1-OAc)(PNN^G)(PPh$_3$)] (4) Pag. S16

Synthesis of *trans*-[Ru(η^1-OAc)$_2$(PNN')(PPh$_2$Me)] (5) Pag. S16

Synthesis of *trans*-[Ru(η^1-OAc)$_2$(PNN^G)(PPh$_2$Me)] (6) and [Ru(η^2-OAc)(PNN^G)(PPh$_2$Me)]OAc (7) mixture Pag. S17

Synthesis of [Ru(η^2-OAc)(CO)(PNN')]OAc (8) and *cis*-[Ru(η^1-OAc)$_2$(CO)(PNN')] (9) mixture Pag. S18

Synthesis of *cis*-[Ru(η^1-OAc)$_2$(CO)(PNN(C$_2$)$_{imine}$)] (10) Pag. S19

Typical procedure for hydrogenation of methyl decanoate Pag. S19

Typical procedure for hydrogenation of methyl and ethyl esters Pag. S20

Typical procedure for base-free hydrogenation of aldehydes Pag. S20

Single-Crystal X-ray Crystallography Structure Determination of Compound 10 Pag. S20

Accession Codes Pag. S21

Figure S1. ^{31}P{^{1}H} NMR spectrum of *trans*-[Ru(η^1-OAc)$_2$(PNN')(PPh$_3$)] (**1**) Pag. S22

Figure S2. ^{1}H NMR spectrum of *trans*-[Ru(η^1-OAc)$_2$(PNN')(PPh$_3$)] (**1**) Pag. S23

Figure S3. ^{13}C{^{1}H} DEPTQ NMR spectrum of *trans*-[Ru(η^1-OAc)$_2$(PNN')(PPh$_3$)] (**1**) Pag. S24

Figure S4. ^{1}H-^{1}H COSY 2D NMR spectrum of *trans*-[Ru(η^1-OAc)$_2$(PNN')(PPh$_3$)] (**1**) Pag. S25

Figure S5. ^{1}H-^{13}C HSQC 2D NMR spectrum of *trans*-[Ru(η^1-OAc)$_2$(PNN')(PPh$_3$)] (**1**) Pag. S26

Figure S6. ^{1}H-^{13}C HMBC 2D NMR spectrum of *trans*-[Ru(η^1-OAc)$_2$(PNN')(PPh_3)] (**1**) Pag. S27

Figure S7. ^{1}H-^{31}P HMBC 2D NMR spectrum of *trans*-[Ru(η^1-OAc)$_2$(PNN')(PPh_3)] (**1**) Pag. S28

Figure S8. ^{1}H-^{1}H NOESY 2D NMR spectrum of *trans*-[Ru(η^1-OAc)$_2$(PNN')(PPh_3)] (**1**) Pag. S29

Figure S9. ^{1}H-^{15}N HSQC 2D NMR spectrum of *trans*-[Ru(η^1-OAc)$_2$(PNN')(PPh_3)] (**1**) Pag. S30

Figure S10. $^{31}P\{^{1}H\}$ NMR spectrum of *trans*-[Ru(η^1-OAc)$_2$(PNN^G)(PPh_3)] (**2**) Pag. S31

Figure S11. ^{1}H NMR spectrum of *trans*-[Ru(η^1-OAc)$_2$(PNN^G)(PPh_3)] (**2**) Pag. S32

Figure S12. $^{13}C\{^{1}H\}$ DEPTQ NMR spectrum of *trans*-[Ru(η^1-OAc)$_2$(PNN^G)(PPh_3)] (**2**) Pag. S33

Figure S13. ^{1}H-^{1}H COSY 2D NMR spectrum of *trans*-[Ru(η^1-OAc)$_2$(PNN^G)(PPh_3)] (**2**) Pag. S34

Figure S14. ^{1}H-^{13}C HSQC 2D NMR spectrum of *trans*-[Ru(η^1-OAc)$_2$(PNN^G)(PPh_3)] (**2**) Pag. S35

Figure S15. ^{1}H-^{13}C HMBC 2D NMR spectrum of *trans*-[Ru(η^1-OAc)$_2$(PNN^G)(PPh_3)] (**2**) Pag. S36

Figure S16. ^{1}H-^{31}P HMBC 2D NMR spectrum of *trans*-[Ru(η^1-OAc)$_2$(PNN^G)(PPh_3)] (**2**) Pag. S37

Figure S17. ^{1}H-^{1}H NOESY 2D NMR spectrum of *trans*-[Ru(η^1-OAc)$_2$(PNN^G)(PPh_3)] (**2**) Pag. S38

Figure S18. $^{31}P\{^{1}H\}$ NMR spectrum of *trans*-[RuH(η^1-OAc)$_2$(PNN')(PPh_3)] (**3**) Pag. S39

Figure S19. ^{1}H NMR spectrum of *trans*-[RuH(η^1-OAc)(PNN')(PPh_3)] (**3**) Pag. S40

Figure S20. $^{13}C\{^{1}H\}$ DEPTQ NMR spectrum of *trans*-[RuH(η^1-OAc)(PNN')(PPh_3)] (**3**) Pag. S41

Figure S21. ^{1}H-^{1}H COSY 2D NMR spectrum of *trans*-[RuH(η^1-OAc)(PNN')(PPh_3)] (**3**) Pag. S42

Figure S22. ^{1}H-^{13}C HSQC 2D NMR spectrum of *trans*-[RuH(η^1-OAc)(PNN')(PPh_3)] (**3**)

Pag. S43

Figure S23. ^{1}H-^{13}C HMBC 2D NMR spectrum of *trans*-[RuH(η^1-OAc)(PNN')(PPh_3)] (**3**)

Pag. S44

Figure S24. ^{1}H-^{31}P HMBC 2D NMR spectrum of *trans*-[RuH(η^1-OAc)(PNN')(PPh_3)] (**3**)

Pag. S45

Figure S25. ^{1}H-^{1}H NOESY 2D NMR spectrum of *trans*-[RuH(η^1-OAc)(PNN')(PPh_3)] (**3**)

Pag. S46

Figure S26. ^{1}H-^{15}N HSQC 2D NMR spectrum of *trans*-[RuH(η^1-OAc)(PNN')(PPh_3)] (**3**)

Pag. S47

Figure S27. ^{31}P{^{1}H} NMR spectrum of *trans*-[RuH(η^1-OAc)(PNN^G)(PPh_3)] (**4**) Pag. S48

Figure S28. ^{1}H NMR spectrum of *trans*-[RuH(η^1-OAc)(PNN^G)(PPh_3)] (**4**) Pag. S49

Figure S29. ^{13}C{^{1}H} DEPTQ NMR spectrum of *trans*-[RuH(η^1-OAc)(PNN^G)(PPh_3)] (**4**)

Pag. S50

Figure S30. ^{1}H-^{1}H COSY 2D NMR spectrum of *trans*-[RuH(η^1-OAc)(PNN^G)(PPh_3)] (**4**)

Pag. S51

Figure S31. ^{1}H-^{13}C HSQC 2D NMR spectrum of *trans*-[RuH(η^1-OAc)(PNN^G)(PPh_3)] (**4**)

Pag. S52

Figure S32. ^{1}H-^{13}C HMBC 2D NMR spectrum of *trans*-[RuH(η^1-OAc)(PNN^G)(PPh_3)] (**4**)

Pag. S53

Figure S33. ^{1}H-^{31}P HMBC 2D NMR spectrum of *trans*-[RuH(η^1-OAc)(PNN^G)(PPh_3)] (**4**)

Pag. S54

Figure S34. ^{1}H-^{1}H NOESY 2D NMR spectrum of *trans*-[RuH(η^1-OAc)(PNN^G)(PPh_3)] (**4**)

Pag. S55

Figure S35. ^{1}H-^{15}N HSQC 2D NMR spectrum of *trans*-[RuH(η^1-OAc)(PNN^G)(PPh_3)] (**4**)

Pag. S56

Figure S36. $^{31}P\{^1H\}$ NMR spectrum of *trans*-[Ru(η^1-OAc)$_2$(PNN')(PPh$_2$Me)] (**5**) Pag. S57

Figure S37. ^1H NMR spectrum of *trans*-[Ru(η^1-OAc)$_2$(PNN')(PPh$_2$Me)] (**5**) Pag. S58

Figure S38. $^{13}C\{^1H\}$ DEPTQ NMR spectrum of *trans*-[Ru(η^1-OAc)$_2$(PNN')(PPh$_2$Me)] (**5**) Pag. S59

Figure S39. ^1H-^1H COSY 2D NMR spectrum of *trans*-[Ru(η^1-OAc)$_2$(PNN')(PPh$_2$Me)] (**5**) Pag. S60

Figure S40. ^1H-^{13}C HSQC 2D NMR spectrum of *trans*-[Ru(η^1-OAc)$_2$(PNN')(PPh$_2$Me)] (**5**) Pag. S61

Figure S41. ^1H-^{13}C HMBC 2D NMR spectrum of *trans*-[Ru(η^1-OAc)$_2$(PNN')(PPh$_2$Me)] (**5**) Pag. S62

Figure S42. ^1H-^{31}P HMBC 2D NMR spectrum of *trans*-[Ru(η^1-OAc)$_2$(PNN')(PPh$_2$Me)] (**5**) Pag. S63

Figure S43. ^1H-^1H NOESY 2D NMR spectrum of *trans*-[Ru(η^1-OAc)$_2$(PNN')(PPh$_2$Me)] (**5**) Pag. S64

Figure S44. ^1H-^{15}N HSQC 2D NMR spectrum of *trans*-[Ru(η^1-OAc)$_2$(PNN')(PPh$_2$Me)] (**5**) Pag. S65

Figure S45. $^{31}P\{^1H\}$ NMR spectrum of the mixture of *trans*-[Ru(η^1-OAc)$_2$(PNN^G)(PPh$_2$Me)] (**6**) and [Ru(η^2-OAc)(PNN^G)(PPh$_2$Me)]OAc (**7**) Pag. S66

Figure S46. ^1H NMR spectrum of the mixture of *trans*-[Ru(η^1-OAc)$_2$(PNN^G)(PPh$_2$Me)] (**6**) and [Ru(η^2-OAc)(PNN^G)(PPh$_2$Me)]OAc (**7**) Pag. S67

Figure S47. $^{13}C\{^1H\}$ DEPTQ NMR spectrum of the mixture of *trans*-[Ru(η^1-OAc)$_2$(PNN^G)(PPh$_2$Me)] (**6**) and [Ru(η^2-OAc)(PNN^G)(PPh$_2$Me)]OAc (**7**) Pag. S68

Figure S48. ^1H-^1H COSY 2D NMR spectrum of the mixture of *trans*-[Ru(η^1-OAc)$_2$(PNN^G)(PPh$_2$Me)] (**6**) and [Ru(η^2-OAc)(PNN^G)(PPh$_2$Me)]OAc (**7**) Pag. S69

Figure S49. ^{1}H-^{13}C HSQC 2D NMR spectrum of the mixture of *trans*-[Ru(η^1-OAc)$_2$(PNN^G)(PPh$_2$Me)] (**6**) and [Ru(η^2-OAc)(PNN^G)(PPh$_2$Me)]OAc (**7**) Pag. S70

Figure S50. ^{1}H-^{13}C HMBC 2D NMR spectrum of the mixture of *trans*-[Ru(η^1-OAc)$_2$(PNN^G)(PPh$_2$Me)] (**6**) and [Ru(η^2-OAc)(PNN^G)(PPh$_2$Me)]OAc (**7**) Pag. S71

Figure S51. ^{1}H-^{31}P HMBC 2D NMR spectrum of the mixture of *trans*-[Ru(η^1-OAc)$_2$(PNN^G)(PPh$_2$Me)] (**6**) and [Ru(η^2-OAc)(PNN^G)(PPh$_2$Me)]OAc (**7**) Pag. S72

Figure S52. ^{1}H-^{1}H NOESY 2D NMR spectrum of the mixture of *trans*-[Ru(η^1-OAc)$_2$(PNN^G)(PPh$_2$Me)] (**6**) and [Ru(η^2-OAc)(PNN^G)(PPh$_2$Me)]OAc (**7**) Pag. S73

Figure S53. ^{1}H-^{15}N HSQC 2D NMR spectrum of the mixture of *trans*-[Ru(η^1-OAc)$_2$(PNN^G)(PPh$_2$Me)] (**6**) and [Ru(η^2-OAc)(PNN^G)(PPh$_2$Me)]OAc (**7**) Pag. S74

Figure S54. ^{31}P{^{1}H} NMR spectrum of the mixture of [Ru(η^2-OAc)(CO)(PNN')]OAc (**8**) and *cis*-[Ru(η^1-OAc)$_2$(CO)(PNN')] (**9**) in CD_3OD Pag. S75

Figure S55. ^{31}P{^{1}H} NMR spectrum of the mixture of [Ru(η^2-OAc)(CO)(PNN')]OAc (**8**) and *cis*-[Ru(η^1-OAc)$_2$(CO)(PNN')] (**9**) in CD_2Cl_2 Pag. S76

Figure S56. ^{1}H NMR spectrum of the mixture of [Ru(η^2-OAc)(CO)(PNN')]OAc (**8**) and *cis*-[Ru(η^1-OAc)$_2$(CO)(PNN')] (**9**) in CD_3OD Pag. S77

Figure S57. ^{1}H NMR spectrum of the mixture of [Ru(η^2-OAc)(CO)(PNN')]OAc (**8**) and *cis*-[Ru(η^1-OAc)$_2$(CO)(PNN')] (**9**) in CD_2Cl_2 Pag. S78

Figure S58. ^{13}C{^{1}H} DEPTQ NMR spectrum of the mixture of [Ru(η^2-OAc)(CO)(PNN')]OAc (**8**) and *cis*-[Ru(η^1-OAc)$_2$(CO)(PNN')] (**9**) in CD_2Cl_2 Pag. S79

Figure S59. ^{1}H-^{1}H COSY 2D NMR spectrum of the mixture of [Ru(η^2-OAc)(CO)(PNN')]OAc (**8**) and *cis*-[Ru(η^1-OAc)$_2$(CO)(PNN')] (**9**) in CD_2Cl_2 Pag. S80

Figure S60. ^{1}H-^{13}C HSQC 2D NMR spectrum of the mixture of [Ru(η^2-OAc)(CO)(PNN')]OAc (**8**) and *cis*-[Ru(η^1-OAc)$_2$(CO)(PNN')] (**9**) in CD_2Cl_2 Pag. S81

Figure S61. ^{1}H-^{13}C HMBC 2D NMR spectrum of the mixture of [Ru(η^2-OAc)(CO)(PNN')]OAc (**8**)

and *cis*-[Ru(η^1-OAc)$_2$(CO)(PNN')] (**9**) in CD_2Cl_2 Pag. S82

Figure S62. ^{1}H-^{31}P HMBC 2D NMR spectrum of the mixture of [Ru(η^2-OAc)(CO)(PNN')]OAc (**8**) and *cis*-[Ru(η^1-OAc)$_2$(CO)(PNN')] (**9**) in CD_2Cl_2 Pag. S83

Figure S63. ^{1}H-^{1}H NOESY 2D NMR spectrum of the mixture of [Ru(η^2-OAc)(CO)(PNN')]OAc (**8**) and *cis*-[Ru(η^1-OAc)$_2$(CO)(PNN')] (**9**) in CD_2Cl_2 Pag. S84

Figure S64. ^{1}H-^{15}N HSQC 2D NMR spectrum of the mixture of [Ru(η^2-OAc)(CO)(PNN')]OAc (**8**) and *cis*-[Ru(η^1-OAc)$_2$(CO)(PNN')] (**9**) in CD_2Cl_2 Pag. S85

Figure S65. ^{31}P{^{1}H} NMR spectrum of *cis*-[Ru(η^1-OAc)^2(CO)(PNN(C_2)$_{imine}$)] (**10**) Pag. S86

Figure S66. ^{1}H NMR spectrum of *cis*-[Ru(η^1-OAc)^2(CO)(PNN(C_2)$_{imine}$)] (**10**) Pag. S87

Figure S67. ^{13}C{^{1}H} DEPTQ NMR spectrum of *cis*-[Ru(η^1-OAc)^2(CO)(PNN(C_2)$_{imine}$)] (**10**) Pag. S88

Figure S68. ^{1}H-^{1}H COSY 2D NMR spectrum of *cis*-[Ru(η^1-OAc)^2(CO)(PNN(C_2)$_{imine}$)] (**10**) Pag. S89

Figure S69. ^{1}H-^{13}C HSQC 2D NMR spectrum of *cis*-[Ru(η^1-OAc)^2(CO)(PNN(C_2)$_{imine}$)] (**10**) Pag. S90

Figure S70. ^{1}H-^{13}C HMBC 2D NMR spectrum of *cis*-[Ru(η^1-OAc)^2(CO)(PNN(C_2)$_{imine}$)] (**10**) Pag. S91

Figure S71. ^{1}H-^{31}P HMBC 2D NMR spectrum of *cis*-[Ru(η^1-OAc)^2(CO)(PNN(C_2)$_{imine}$)] (**10**) Pag. S92

Figure S72. ^{1}H-^{1}H NOESY 2D NMR spectrum of *cis*-[Ru(η^1-OAc)^2(CO)(PNN(C_2)$_{imine}$)] (**10**) Pag. S93

Table S1. Further data regarding the catalytic hydrogenation of methyl decanoate with complexes **1-10**, *trans*-[$RuCl_2$(PNN')(PPh_3)] and *trans*-[$RuCl_2$(PNN^G)(PPh_3)] (S/C = 1000-100000), with NaOMe (50 mol%) at 27.5 bar of H_2 Pag. S94

Table S2. Further data regarding the catalytic hydrogenation of methyl and ethyl esters with complex

2 and *trans*-[$RuCl_2$(PNN^G)(PPh_3)] (S/C = 10000-100000), with base (50 mol%) at 27.5 bar of H_2 Pag. S95

Table S3. Further data regarding the base-free catalytic hydrogenation of 1-decanal with complexes **1-4**, **8**/**9**, *trans*-[$RuCl_2$(PNN')(PPh_3)] and *trans*-[$RuCl_2$(PNN^G)(PPh_3)], at 27.5 bar of H_2 and in absence of base Pag. S96

Figure S73. Effect of heating on the formation of ruthenium hydrides by reaction of *trans*-[Ru(η^1-OAc)$_2$(PNN^G)(PPh_3)] (**2**) with H_2 (5 bar) and KO^{t}Bu (3 equiv) in the $^{31}P\{^1H\}$ NMR spectra Pag. S97

Figure S74. ^{1}H NMR spectrum of the ruthenium hydride obtained by reaction of *trans*-[Ru(η^1-OAc)$_2$(PNN^G)(PPh_3)] (**2**) with H_2 (5 bar) and KO^{t}Bu (3 equiv) after heating at 50 °C for 4 h Pag. S98

Figure S75. Effect of heating on the formation of ruthenium hydrides by reaction of *trans*-[Ru(η^1-OAc)$_2$(PNN')(PPh_3)] (**1**) with H_2 (5 bar) and KO^{t}Bu (3 equiv) in the $^{31}P\{^1H\}$ NMR spectra Pag. S99

Figure S76. Effect of heating on the formation of ruthenium hydrides by reaction of *trans*-[Ru(η^1-OAc)$_2$(PNN')(PPh_3)] (**1**) with H_2 (5 bar) and KO^{t}Bu (3 equiv) in the hydride region of the ^{1}H NMR spectra Pag. S100

Figure S77. ^{1}H-^{1}H COSY 2D NMR spectrum of the mixture of ruthenium monohydrides obtained by reaction of *trans*-[Ru(η^1-OAc)$_2$(PNN')(PPh_3)] (**1**) with H_2 (5 bar) and KO^{t}Bu (3 equiv) after heating at 50 °C for 18 h (range 12.0/-22.0 ppm) Pag. S101

Figure S78. ^{1}H-^{1}H COSY 2D NMR spectrum of the mixture of ruthenium monohydrides obtained by reaction of *trans*-[Ru(η^1-OAc)$_2$(PNN')(PPh_3)] (**1**) with H_2 (5 bar) and KO^{t}Bu (3 equiv) after heating at 50 °C for 18 h (range 18.0/0.0 ppm) Pag. S102

Figure S79. Effect of heating (70 °C for 16 h) on the ^{1}H NMR spectrum of the mixture of ruthenium hydrides (obtained by reaction of *trans*-[Ru(η^1-OAc)$_2$(PNN')(PPh$_3$)] (**1**) with H_2 and KO^{t}Bu) after the addition of PhCOOMe (5 equiv) in H_2 (5 bar) Pag. S103

Figure S80. ^{1}H NMR spectrum of the ruthenium hydride mixture obtained by reaction of *trans*-[RuH(η^1-OAc)(PNN')(PPh$_3$)] (**3**) with H_2 (5 bar) and KO^{t}Bu (3 equiv) after heating at 50 °C for 16 h Pag. S104

Figure S81. ^{31}P{^{1}H} NMR spectrum of the mixture of ruthenium hydrides obtained by reaction of *trans*-[RuH(η^1-OAc)(PNN')(PPh$_3$)] (**3**) with H_2 (5 bar) and KO^{t}Bu (3 equiv) after heating at 50 °C for 16 h Pag. S105

Figure S82. ^{1}H-^{31}P HMBC 2D NMR spectrum of the mixture of ruthenium hydrides obtained by reaction of *trans*-[RuH(η^1-OAc)(PNN')(PPh$_3$)] (**3**) with H_2 (5 bar) and KO^{t}Bu (3 equiv) after heating at 50 °C for 16 h Pag. S106

Figure S83. Hydrogen uptake curve of complexes **2**,**4** and **6**/**7** and the *trans*-[RuCl$_2$(PNN^G)(PPh$_3$)] derivative for the solvent-free reduction of methyl decanoate **a** at 90 °C, 27.5 bar of H_2, 50 mol% NaOMe (S/C 10000) Pag. S107

Figure S84. Hydrogen uptake curve of complexes **2**,**4** and **6**/**7** and the *trans*-[RuCl$_2$(PNN^G)(PPh$_3$)] derivative for the solvent-free reduction of methyl decanoate **a** at 40 °C, 27.5 bar of H_2, 50 mol% NaOMe (S/C 50000) Pag. S107

Figure S85. Hydrogen uptake curve of complexes **2**,**4** and **6**/**7** and the *trans*-[RuCl$_2$(PNN^G)(PPh$_3$)] derivative for the solvent-free reduction of methyl decanoate **a** at 90 °C, 27.5 bar of H_2, 50 mol% NaOMe (S/C 50000) Pag. S108

Figure S86. Hydrogen uptake curve of complex **2** and the *trans*-[RuCl$_2$(PNN^G)(PPh$_3$)] derivative for the reduction of methyl decanoate **a** at 40 °C, 27.5 bar of H_2, 50 mol% NaOMe (S/C 100000) Pag. S108

Figure S87. Hydrogen uptake curve of complex **2** for the reduction of methyl decanoate **a** at 40 °C, 27.5 bar of H_2, 50 mol% NaOMe (S/C 50000) using toluene as solvent Pag. S109

Figure S88. Hydrogen uptake curve of complex **2** and the *trans*-[$RuCl_2(PNN^G)(PPh_3)$] derivative for the solvent-free reduction of ethyl decanoate **b** at 40 °C, 27.5 bar of H_2, 50 mol% NaOEt (S/C 100000) Pag. S109

Figure S89. Hydrogen uptake curve of complex **2** for the solvent-free reduction of ethyl dodecanoate **c** at 40 °C, 27.5 bar of H_2, 50 mol% NaOEt (S/C 44000) Pag. S110

Figure S90. Hydrogen uptake curve of complex **2** and the *trans*-[$RuCl_2(PNN^G)(PPh_3)$] derivative for the solvent-free reduction of ethyl 10-undecenoate **d** at 30 °C, 27.5 bar of H_2, 50 mol% NaOEt (S/C 10000) Pag. S110

Figure S91. Hydrogen uptake curve of complex **2** and the *trans*-[$RuCl_2(PNN^G)(PPh_3)$] derivative for the solvent-free reduction of methyl oleate **e** at 60 °C, 27.5 bar of H_2, 50 mol% NaOMe (S/C 25000) Pag. S111

Figure S92. Hydrogen uptake curve of complex **2** and the *trans*-[$RuCl_2(PNN^G)(PPh_3)$] derivative for the solvent-free reduction of methyl oleate **e** at 50 °C, 27.5 bar of H_2, 50 mol% NaOMe (S/C 50000) Pag. S111

Figure S93. Hydrogen uptake curve of complex **2** and the *trans*-[$RuCl_2(PNN^G)(PPh_3)$] derivative for the solvent-free reduction of methyl oleate **e** at 50 °C, 27.5 bar of H_2, 50 mol% NaOMe (S/C 100000) Pag. S112

Figure S94. Hydrogen uptake curve of complex **2** and the *trans*-[$RuCl_2(PNN^G)(PPh_3)$] derivative for the solvent-free reduction of ethyl oleate **f** at 40 °C, 27.5 bar of H_2, 50 mol% NaOEt (S/C 50000) Pag. S112

Figure S95. Hydrogen uptake curve of complex **2** and the *trans*-[$RuCl_2(PNN^G)(PPh_3)$] derivative for the solvent-free reduction of ethyl oleate **f** at 40 °C, 27.5 bar of H_2, 50 mol% NaOEt (S/C 100000) Pag. S113

Figure S96. Hydrogen uptake curve of complex **2** and the *trans*-[$RuCl_2(PNN^G)(PPh_3)$] derivative for the solvent-free reduction of methyl benzoate **g** at 40 °C, 27.5 bar of H_2, 50 mol% NaOEt (S/C 10000) Pag. S113

Figure S97. Hydrogen uptake curve of complex **2** and the *trans*-[$RuCl_2(PNN^G)(PPh_3)$] derivative for the solvent-free reduction of methyl benzoate **g** at 40 °C, 27.5 bar of H_2, 50 mol% NaOEt (S/C 25000) Pag. S114

Figure S98. Hydrogen uptake curve of complex **2** and the *trans*-[$RuCl_2(PNN^G)(PPh_3)$] derivative for the solvent-free reduction of methyl benzoate **g** at 40 °C, 27.5 bar of H_2, 50 mol% NaOEt (S/C 50000) Pag. S114

Figure S99. Hydrogen uptake curve of complex **2** and the *trans*-[$RuCl_2(PNN^G)(PPh_3)$] derivative for the solvent-free reduction of methyl benzoate **g** at 40 °C, 27.5 bar of H_2, 50 mol% NaOEt (S/C 80000) Pag. S115

Figure S100. Hydrogen uptake curve of complex **2** and the *trans*-[$RuCl_2(PNN^G)(PPh_3)$] derivative for the solvent-free reduction of methyl benzoate **g** at 40 °C, 27.5 bar of H_2, 50 mol% NaOEt (S/C 100000) Pag. S115

Figure S101. Hydrogen uptake curve of complex **2** and the *trans*-[$RuCl_2(PNN^G)(PPh_3)$] derivative for the solvent-free reduction of methyl benzoate **g** at 40 °C, 27.5 bar of H_2, 50 mol% NaOMe (S/C 100000) Pag. S116

Figure S102. Hydrogen uptake curve of complex **2** and the *trans*-[$RuCl_2(PNN^G)(PPh_3)$] derivative for the solvent-free reduction of ethyl benzoate **h** at 40 °C, 27.5 bar of H_2, 50 mol% NaOEt (S/C 100000) Pag. S116

GC analytical method for methyl and ethyl decanoate hydrogenation Pag. S117

Figure S103. Representative example of GC chromatogram of the catalytic hydrogenation of methyl decanoate **a** using Ru PNN complexes, containing all the species involved in the reaction Pag. S118

Figure S104. GC trace of products of methyl decanoate **a** hydrogenation catalysed by complex **2** (S/C 50000), showing full conversion to 1-decanol Pag. S118

Figure S105. Representative example of GC chromatogram of the catalytic hydrogenation of ethyl decanoate **b** using Ru PNN complexes, containing all the species involved in the reaction Pag. S119

Figure S106. GC trace of products of ethyl decanoate **b** hydrogenation catalysed by complex **2** (S/C 50000), showing full conversion to 1-decanol Pag. S119

GC analytical method for ethyl dodecanoate hydrogenations Pag. S120

Figure S107. Representative example of GC chromatogram of the catalytic hydrogenation of ethyl dodecanoate **c** using Ru PNN complexes, containing all the species involved in the reaction Pag. S121

Figure S108. GC trace of products of ethyl dodecanoate **c** hydrogenation catalysed by complex **2** (S/C 44000), showing full conversion to 1-dodecanol Pag. S121

GC analytical method for ethyl 10-undecenoate hydrogenations Pag. S122

Figure S109. Representative example of GC chromatogram of the catalytic hydrogenation of ethyl 10-undecenoate **d** using Ru PNN complexes, containing all the species involved in the reaction Pag. S123

Figure S110. GC trace of products of ethyl 10-undecenoate **d** hydrogenation catalysed by complex **2** (S/C 10000), showing full conversion to 10-undecen-1-ol Pag. S123

GC analytical method for methyl and ethyl oleate hydrogenations Pag. S124

Figure S111. Representative GC trace of starting material reference for technical grade methyl oleate **e**, showing composition (%) as a ratio of peak areas Pag. S125

Figure S112. Representative GC trace of hydrogenation products of methyl oleate **e** catalysed by complex **2** (S/C 25000) Pag. S125

Figure S113. GC trace of starting material reference for technical grade ethyl oleate **f**, showing composition (%) as a ratio of peak areas Pag. S126

Figure S114. GC trace of hydrogenation products of ethyl oleate **f** catalyzed by complex **2** (S/C 50000) Pag. S126

GC analytical method for methyl and ethyl benzoate hydrogenations Pag. S127

Figure S115. Representative example of GC chromatogram of the catalytic hydrogenation of methyl benzoate **g** using Ru PNN complexes containing all the species involved in the reaction Pag. S128

Figure S116. GC trace of products of methyl benzoate **g** hydrogenation catalysed by complex **2** (S/C 10000), showing full conversion to benzyl alcohol Pag. S128

Figure S117. Representative example of GC chromatogram of the catalytic hydrogenation of ethyl benzoate **h** using Ru PNN complexes containing all the species involved in the reaction Pag. S129

Figure S118. GC trace of products of ethyl benzoate **h** hydrogenation catalysed by complex **2** (S/C 100000), showing full conversion to benzyl alcohol Pag. S129

Single crystal X-ray structure determination of compound 10. General data. Pag. S130

Figure S119. Molecular structure of complex **10** Pag. S131

Single crystal X-ray structure determination of complex 10. Detailed crystallographic data. Pag. S132

References Pag. S134

Experimental section

Materials and methods

All reactions were carried out under an argon atmosphere using standard Schlenk techniques. The solvents were carefully dried by standard methods and distilled under argon before use. The ruthenium complexes [Ru(η^2-OAc)$_2$(PPh$_3$)$_2$],[69] [RuH(η^2-OAc)(PPh$_3$)$_3$],[70] [Ru(η^1-OAc)(η^2-OAc)(CO)(PPh$_3$)$_2$],[71] and the PNN',[52] PNN^G,[33, 36] and PNN(C$_2$)$_{imine}$[54] ligands were prepared according to literature procedures, whereas all other chemicals were purchased from Merck and Strem and used without further purification. NMR measurements were recorded on Bruker Avance III HD NMR 400 spectrometer. Chemical shifts (ppm) are relative to TMS for ^{1}H and ^{13}C{^{1}H}, whereas H$_3$PO$_4$ was used for ^{31}P{^{1}H}. Elemental analyses (C, H, N) were carried out with a Carlo Erba 1106 analyzer, whereas GC analyses of catalytic products were performed with a Varian CP-3900 gas chromatograph system equipped with an Agilent HP-88, 60 m x 0.25 mm x 0.2 µm column using nitrogen (1.2 mL/min.) as the carrier gas and a flame ionization detector (FID).

Synthesis of *trans*-[Ru(η^1-OAc)$_2$(PNN')(PPh$_3$)] (1). ***Method 1.*** [Ru(η^2-OAc)$_2$(PPh$_3$)$_2$] (200 mg, 0.269 mmol) was suspended in acetone (5 mL) and the PNN' ligand (108 mg, 0.282 mmol, 1.05 equiv) was added. The mixture was stirred for 3 h at room temperature. The obtained yellow precipitate was filtered, washed with diethyl ether (2x2 mL), *n*-heptane (4x3 mL), *n*-pentane (2x3 mL) and dried under reduced pressure. Yield: 198 mg (85%).

Anal. Calcd for C$_{47}$H$_{44}$N$_2$O$_4$P$_2$Ru (863.90): C, 65.35; H, 5.13; N, 3.24. Found: C, 65.32; H, 5.14; N, 3.13. ^{1}H NMR (400.1 MHz, toluene-*d*^{8}, 25 °C): δ 10.96 (br t, ^3J(H,H) = 10.9 Hz, 1H; NH), 8.60 (d, ^3J(H,H) = 5.0 Hz, 1H; *ortho*-CH of C$_5$H$_4$N), 8.04-7.30 (br m, 3H; aromatic protons), 7.39-7.30 (m, 1H; aromatic proton), 7.22 (dd, ^3J(H,H) = 6.9 Hz, ^4J(H,H) = 3.6 Hz, 1H; aromatic proton), 7.18-6.97 (m, 17H; aromatic protons), 6.94-6.72 (m, 6H; aromatic protons), 6.68 (d, ^3J(H,H) = 7.6 Hz, 1H; *para*-CH of C$_5$H$_4$N), 6.31 (t, ^3J(H,H) = 8.6 Hz, 2H; aromatic protons), 6.10 (t, ^3J(H,H) = 6.5 Hz, 1H; *meta*-CH of C$_5$H$_4$N), 5.81 (td, ^2J(H,H) = 12.3 Hz, ^4J(H,P) = 2.3 Hz, 1H; NCH$_2$), 5.66 (*pseudo* t, ^2J(H,H) = 13.3 Hz, 1H; NCH$_2$), 3.85 (ddd, ^2J(H,H) = 14.5 Hz, ^4J(H,H) = 5.6 Hz, ^4J(H,P) = 2.9 Hz, 1H; NCH$_2$), 3.65 (ddd, ^2J(H,H) = 11.4 Hz, ^4J(H,H) = 7.3 Hz, ^4J(H,P) = 3.8 Hz, 1H; NCH$_2$), 1.91 (s, 3H; OCOCH$_3$), 1.59 ppm (s, 3H; OCOCH$_3$). ^{13}C{^{1}H} NMR (100.6 MHz, toluene-*d*^{8}, 25 °C): δ 181.1 (s; O*C*OCH$_3$), 177.6 (s; O*C*OCH$_3$), 164.1 (s; N*C*CH$_2$ of C$_5$H$_4$N), 156.3 (d, ^3J(C,P) = 3.7 Hz; N*C*H of C$_5$H$_4$N), 143.0 (d, ^1J(C,P) = 14.7 Hz; *ipso*-Ph), 140.2-123.3 (m; aromatic carbon atoms), 120.9 (s; *meta*-*C*H of C$_5$H$_4$N), 119.8 (s; *para*-*C*H of C$_5$H$_4$N), 60.5 (s; N*C*H$_2$Py), 57.1 (d, ^3J(C,P) = 6.6 Hz;

(C_6H_4)CH_2N), 26.3 (s; OCO*C*H_3), 24.8 ppm (s; OCO*C*H_3). $^{31}P\{^1H\}$ NMR (162.0 MHz, toluene-d^8, 25 °C): δ 46.6 (d, ^2J(P,P) = 29.3 Hz), 38,5 ppm (d, ^2J(P,P) = 29.3 Hz).

Method 2. [Ru(η^2-OAc)$_2$(PPh$_3$)$_2$] (100 mg, 0.134 mmol) and the PNN' ligand (54 mg, 0.141 mmol, 1.05 equiv) were dissolved in toluene (5 mL) and stirred at room temperature for 2 h. The resulting light orange solution was concentrated to about 1 mL removing the solvent under reduced pressure. Addition of diethyl ether (5 mL) afforded the precipitation of the product as a yellow solid that was filtered, washed with *n*-heptane (4x3 mL) and *n*-pentane (4x2 mL) and dried under reduced pressure. Yield: 93 mg (80%).

Synthesis of *trans*-[Ru(η^1-OAc)$_2$(PNN^G)(PPh$_3$)] (2): Complex **2** was prepared following the procedure used for the synthesis of **1** (Method 1), with the PNN^G ligand (90.5 mg, 0.282 mmol, 1.05 equiv) in place of PNN. Yield 174.7 mg (81%). Anal. Calcd for $C_{42}H_{42}N_2O_4P_2Ru$ (801.83): C, 62.91; H, 5.28; N, 3.49. Found: C, 62.86; H, 5.25; N, 3.52. ^{1}H NMR (400.1 MHz, toluene-d^8, 25 °C): δ 11.40 (d, ^3J(H,H) = 11.4 Hz, 1H; NH), 8.53 (d, ^3J(H,H) = 4.8 Hz, 1H; *ortho*-CH of C_5H_4N), 7.62 (t, ^3J(H,H) = 8.4 Hz, 1H; aromatic proton), 7.17-6.83 (m, 25H; aromatic protons), 6.61 (d, ^3J(H,H) = 7.6 Hz, 1H; *para*-CH of C_5H_4N), 6.36 (t, ^3J(H,H) = 6.4 Hz, 1H; *meta*-CH of C_5H_4N), 4.92 (t, ^2J(H,H) = 12.5 Hz, 1H; NCH_2Py), 3.76 (ddd, ^2J(H,H) = 13.1 Hz, ^3J(H,H) = 5.4, ^3J(H,H) = 3.3 Hz, 1H; NCH_2Py), 3.38-3.24 (m, 1H; N*H_2*CH_2P), 3.15-3.02 (m, 1H; NCH_2C*H_2*P), 3.01-2.82 (m, 1H; NC*H_2*CH_2P), 2.52 (td, ^2J(H,H) = 12.7 Hz, ^3J(H,H) = 3.6 Hz, 1H; NCH_2C*H_2*P), 1.88 (s, 3H; OCOCH_3), 1.44 ppm (s, 3H; OCOCH_3). $^{13}C\{^1H\}$ NMR (100.6 MHz, toluene-d^8, 25 °C): δ 182.2 (d, ^3J(C,P) = 2.2 Hz; O*C*OCH_3), 178.0 (s; O*C*OCH_3), 166.0 (s; N*C*CH_2), 155.2 (d, ^3J(C,P) = 2.2 Hz; N*C*H of C_5H_4N), 140.2 (d, ^1J(C,P) = 30.1 Hz; *ipso*-Ph), 137.1-124.5 (m; aromatic carbon atoms), 121.2 (d, ^4J(C,P) = 1.7 Hz; *meta*-*C*H of C_5H_4N), 119.3 (s; *para*-*C*H of C_5H_4N), 58.2 (br s; NCH_2Py), 48.3 (br s; N*C*$H_2$$CH_2$P), 38.9 (d, ^1J(C,P) = 24.2 Hz; NCH_2*C*H_2P), 25.1 (s; OCO*C*H_3), 24.8 ppm (s; OCO*C*H_3). $^{31}P\{^1H\}$ NMR (162.0 MHz, toluene-d^8, 25 °C): δ 53.8 (d, ^2J(P,P) = 29.3 Hz), 47.6 ppm (d. ^2J(P,P) = 29.3 Hz).

Synthesis of *trans*-[RuH(η^1-OAc)(PNN')(PPh$_3$)] (3). [RuH(η^2-OAc)(PPh$_3$)$_3$] (100 mg, 0.106 mmol) and the PNN' ligand (44.4 mg, 0.116 mmol, 1.1 equiv) were suspended in *n*-heptane (10 mL), and the slurry was refluxed for 2 h. The yellow solid was filtered, washed with diethyl ether (2x3 mL), *n*-pentane (3x5 mL), and dried under reduced pressure. Yield: 65 mg, 76%. Anal. Calcd for $C_{45}H_{42}N_2O_2P_2Ru$ (805.86): C, 67.07; H, 5.25; N, 3.48. Found: C, 67.10; H, 5.27; N, 3.41. ^{1}H NMR (400.1 MHz, toluene-d^8, 25 °C): δ 10.12 (t, ^3J(H,H) = 9.3 Hz, 1H; NH), 8.17 (d, ^3J(H,H) = 2.5 Hz, 1H; *ortho*-CH of C_5H_4N), 8.78-6.75 (m, 29H; aromatic protons), 6.69 (t, ^3J(H,H) = 6.8 Hz, 1H; *para*-CH of C_5H_4N), 6.56 (d, ^3J(H,H) = 7.1 Hz, 1H; *meta*-CH of C_5H_4N), 5.93 (t, ^3J(H,H) = 7.1 Hz, 1H; *meta*-CH of C_5H_4N), 4.19 (*pseudo* t, J(H,H) = 12.1 Hz, 1H; NCH_2), 3.95 (pseudo t, J(H,H) = 10.6 Hz, 1H; NCH_2), 3.76 (br d, J(H,H) = 12.2 Hz, 1H; NCH_2), 3.59 (pseudo t, J(H,H) = 5.0 Hz, 1H;

NCH_2), 1.88 (s, 3H; $OCOCH_3$), -18.62 ppm (t, ^2J(H,P) = 27.5 Hz, 1H; Ru-H). $^{13}C\{^1H\}$ NMR (100.6 MHz, toluene-d^8, 25 °C): δ 179.4 (s; O*C*OCH$_3$), 160.4 (s; N*C*CH$_2$ of C_5H_4N), 155.3 (d, ^3J(C,P) = 2.2 Hz; N*C*H of C_5H_4N), 140.7 (d, ^1J(C,P) = 14.7 Hz; *ipso*-Ph), 138.7-124.5 (m; aromatic carbon atoms), 121.6 (s; *meta*-*C*H of C_5H_4N), 118.6 (s; *para*-*C*H of C_5H_4N), 62.5 (s; N*C*H$_2$Py), 59.1 (d, ^3J(C,P) = 5.1 Hz; (C_6H_4)*C*H$_2$N), 25.8 ppm (s; OCO*C*H$_3$). $^{31}P\{^1H\}$ NMR (162.0 MHz, toluene-d^8, 25 °C): δ 63.3 (d, ^2J(P,P) = 33.6 Hz), 63.1 ppm (d, ^2J(P,P) = 33.6 Hz).

Synthesis of *trans*-[RuH(η^1-OAc)(PNN^G)(PPh_3)] (4). Complex **4** was prepared following the procedure used for the synthesis of **3**, with the PNN^G ligand (35.6 mg, 0.111 mmol, 1.05 equiv) in place of PNN. Yield: 56.0 mg (71%). Anal. Calcd for $C_{40}H_{40}N_2O_2P_2Ru$ (743.79): C, 64.59; H, 5.42; N, 3.77. Found: C, 64.63; H, 5.44; N, 3.76. ^1H NMR (400.1 MHz, toluene-d^8, 25 °C): δ 10.62 (t, ^3J(H,H) = 8.7 Hz, 1H; NH), 8.26 (br d, ^3J(H,H) = 4.9 Hz, 1H; *ortho*-CH of C_5H_4N), 7.91-7.65 (m, 10H; aromatic protons), 7.23-6.87 (m, 15H; aromatic protons), 6.72 (td, ^3J(H,H) = 7,5 Hz, ^4J(H,H) = 1.2 Hz,1H; *para*-CH of C_5H_4N), 6.48 (d, ^3J(H,H) = 7.8 Hz, 1H; *meta*-CH of C_5H_4N), 6.09 (t, ^3J(H,H) = 6.4 Hz, 1H; *meta*-CH of C_5H_4N), 3.97-3.77 (m, 2H; NCH_2Py), 3.17-2.92 (m, 2H; NCH_2CH_2P), 2.44-2.32 (m, 1H; NC*H$_2$*CH$_2$P), 2.27 (td, ^3J(H,H) = 11.8 Hz, ^4J(H,H) = 2.6 Hz,1H; NCH$_2$C*H$_2$*P), 1.89 (s, 3H; $OCOCH_3$), -18.40 ppm (dd, ^2J(H,P) = 26.8 Hz, ^2J(H,P) = 24.3 Hz, 1H; Ru-H). $^{13}C\{^1H\}$ NMR (100.6 MHz, toluene-d^8, 25 °C): δ 180.1 (br d, ^2J(C,P) = 3.9 Hz; O*C*OCH$_3$), 163.1 (s; N*C*CH$_2$), 154.9 (d, ^3J(C,P) = 2.4 Hz; N*C*H of C_5H_4N), 140.1 (d, ^1J(C,P) = 34.5 Hz; *ipso*-Ph), 137.1-124.5 (m; aromatic carbon atoms), 121.8 (s; *meta*-*C*H of C_5H_4N), 119.1 (s; *para*-*C*H of C_5H_4N), 60.5 (br s; NCH_2Py), 51.3 (d, ^3J(C,P) = 2.7 Hz; N*C*H$_2$CH$_2$P), 40.0 (d; ^1J(C,P) = 24.9 Hz; NCH$_2$*C*H$_2$P), 25.8 ppm (s; OCO*C*H$_3$). $^{31}P\{^1H\}$ NMR (162.0 MHz, toluene-d^8, 25 °C): δ 78.0 (d, ^2J(P,P) = 29.3 Hz), 64.5 ppm (d, ^2J(P,P) = 29.3 Hz).

Synthesis of *trans*-[Ru(η^1-OAc)$_2$(PNN')(PPh_2Me)] (5). Complex **1** (200 mg, 0.231 mmol) was dissolved in degassed toluene (5 mL), added of PPh_2Me (47.3 μL, 51 mg, 0.255 mmol, 1.1 equiv), and the mixture was stirred at 40 °C for 2 h. The resulting orange solution was concentrated to about 1 mL removing the solvent under reduced pressure. Addition of *n*-heptane (10 mL) afforded the precipitation of the product as a dark-yellow solid that was filtered, washed with diethyl ether (2x2 mL), *n*-heptane (4x3 mL) and *n*-pentane (4x2 mL) and dried under reduced pressure. Yield: 161.1 mg (87%). Anal. Calcd for $C_{42}H_{42}N_2O_4P_2Ru$ (801.83): C, 62.91; H, 5.28; N, 3.49. Found: C, 62.92; H, 5.30; N, 3.51. ^1H NMR (400.1 MHz, toluene-d^8, 25 °C): δ 10.92 (br t, ^3J(H,H) = 9.5 Hz, 1H; NH), 8.64 (m, 1H; *ortho*-CH of C_5H_4N), 7.62 (t, ^3J(H,H) = 8.0 Hz, 1H; aromatic proton), 7.49 (t, ^3J(H,H) = 8.1 Hz, 1H; aromatic proton), 7.44-7.31 (m, 8H; aromatic protons), 7.25-6.85 (m, 15H; aromatic protons), 6.70 (d, ^3J(H,H) = 7.6 Hz, 1H; *para*-CH of C_5H_4N), 6.28 (t, ^3J(H,H) = 6.4 Hz, 1H; *meta*-CH of C_5H_4N), 5.25-5.00 (m, 2H; NCH_2), 3.79 (dq, ^2J(H,H) = 13.8 Hz, ^4J(H,P) = 3.1 Hz, 1H;

NCH_2Py), 3.60 (ddd, ^2J(H,H) = 10.9 Hz, ^4J(H,H) = 6.4 Hz, ^4J(H,P) = 4.0 Hz, 1H; (C_6H_4)CH_2N), 2.13 (br s, 3H; PCH_3), 1.91 (s, 3H; OCOCH_3), 1.61 ppm (s, 3H; OCOCH_3). ^{13}C{^{1}H} NMR (100.6 MHz, toluene-d^8, 25 °C): δ 181.1 (s; O*C*OCH_3), 177.8 (s; O*C*OCH_3), 163.9 (s; N*C*CH_2 of C_5H_4N), 155.0 (d, ^3J(C,P) = 2.9 Hz; N*C*H of C_5H_4N), 142.5 (d, ^1J(C,P) = 15.4 Hz; *ipso*-Ph), 141.2 (d, ^1J(C,P) = 32.3 Hz; *ipso*-Ph), 138.3 (d, ^1J(C,P) = 35.2 Hz; *ipso*-Ph), 137.3-124.4 (m; aromatic carbon atoms), 121.3 (s; *meta*-*C*H of C_5H_4N), 119.6 (s; *para*-*C*H of C_5H_4N), 59.8 (s; NCH_2Py), 56.3 (d, ^3J(C,P) = 7.3 Hz; (C_6H_4)CH_2N), 25.5 (s; OCOCH_3), 24.7 (s; OCOCH_3), 15.8 ppm (d, ^1J(C,P) = 24.9 Hz; PCH_3). ^{31}P{^{1}H} NMR (162.0 MHz, toluene-d^8, 25 °C): δ 48.8 (d, ^2J(P,P) = 29.3 Hz), 28.1 ppm (d, ^2J(P,P) = 29.3 Hz).

Synthesis of *trans*-[Ru(η^1-OAc)$_2$(PNN^G)(PPh$_2$Me)] (6) and [Ru(η^2-OAc)(PNN^G)(PPh$_2$Me)]OAc (7) mixture. The mixture of complexes **6/7** was prepared by following the procedure used for the synthesis of **5**, employing *trans*-[Ru(η^1-OAc)$_2$(PNN^G)(PPh$_3$)] (**3**) (200 mg, 0.249 mmol) in place of **1** and heating the mixture of reagents at 50 °C for 2 h. The dark-yellow product was obtained as a mixture of **6** / **7** in a 1:1 molar ratio. Yield: 155.0 mg (84%). Anal. Calcd for $C_{37}H_{40}N_2O_4P_2Ru$ (739.75): C, 60.07; H, 5.45; N, 3.79. Found: C, 60.15; H, 5.38; N, 3.71. ^{1}H NMR (400.1 MHz, toluene-d^8, 25 °C): δ 11.14 (br s, 1H; NH compound **6**), 10.00 (br s, 1H; NH compound **7**), 8.50-8.40 (m, 1H; *ortho*-CH of C_5H_4N compound **7**), 8.15-6.69 (m, 19H; aromatic protons), 6.57 (d, ^3J(H,H) = 7.8 Hz, 1H; *para*-CH of C_5H_4N compound **6**), 6.51 (td, ^3J(H,H) = 7.6 Hz, ^4J(H,H) = 1.2 Hz, 1H; *meta*-CH of C_5H_4N compound **6**), 6.42-6.32 (m, 2H; aromatic protons), 6.28 (d, ^3J(H,H) = 7.3 Hz, 1H; *para*-CH of C_5H_4N compound **7**), 5.65 (t, ^3J(H,H) = 6.5 Hz, 1H; *meta*-CH of C_5H_4N compound **7**), 5.37 (dd, ^3J(H,H) = 16.3 Hz, ^4J(H,H) = 6.6 Hz, 1H; NCH_2Py compound **7**), 4.72 (t, ^2J(H,H) = 12.5 Hz, 1H; NCH_2Py compound **6**), 3.82-3.72 (ddd, ^2J(H,H) = 13.8 Hz, ^3J(H,H) = 5.4, ^3J(H,H) = 4.0 Hz, 1H; NCH_2Py compound **6**), 3.51-3.32 (m, 1H; NCH_2CH_2P compound **7**), 3.36-3.28 (m, 1H; NCH_2Py compound **7**), 3.28-3.17 (m, 1H; NCH_2CH_2P compounds **6** and **7**), 3.06-2.96 (m, 1H; NCH_2CH_2P compound **6**), 2.96-2.87 (m, 1H; NCH_2CH_2P compound **6**), 2.79-2.66 (m, 1H; NCH_2CH_2P compound **7**), 2.62-2.50 (m, 1H; NCH_2CH_2P compounds **6** and **7**), 2.22 (br s, 3H; OCOCH_3 compound **7**), 2.17 (s, 3H; OCOCH_3 compound **7**), 2.04 (d, ^2J(H,P) = 8.3 Hz, 3H; PCH_3 compound **6**), 1.88 (s, 3H; OCOCH_3 compound **6**), 1.67 (s, 3H; OCOCH_3 compound **6**), 1.58 ppm (d, ^2J(H,P) = 8.1 Hz, 3H; PCH_3 compound **7**). ^{13}C{^{1}H} NMR (100.6 MHz, toluene-d^8, 25 °C): δ 182.1 (s; O*C*OCH_3 compound **7**), 180.5 (d, ^3J(C,P) = 2.9 Hz; O*C*OCH_3 compound **6**), 177.7 (s; O*C*OCH_3 compound **6**), 177.4 (s; O*C*OCH_3 compound **6**), 165.9 (s; N*C*CH_2 compound **6**), 165.3 (s; N*C*CH_2 compound **7**), 158.2 (s; N*C*H of C_5H_4N compound **7**), 154.4 (s; N*C*H of C_5H_4N compound **6**), 137.3-124.7 (m; aromatic carbon atoms), 121.4 (s; *meta*-*C*H of C_5H_4N compound **6**), 120.1 (s; *meta*-*C*H of C_5H_4N compound **7**), 119.6 (s; *para*-*C*H of C_5H_4N compound **7**), 119.4 (s; *para*-*C*H of C_5H_4N

compound **6**), 59.0 (br s; NCH$_2$Py compound **6**), 57.3 (br s; NCH$_2$Py compound **7**), 50.4 (br s; N*C*H$_2$CH$_2$P compound **7**), 48.1 (br s; N*C*H$_2$CH$_2$P compound **6**), 38.7 (d, ^1J(C,P) = 24.2 Hz; NCH$_2$*C*H$_2$P compound **6**), 35.5 (d, ^1J(C,P) = 24.9 Hz; NCH$_2$*C*H$_2$P compound **7**), 25.6 (s; OCO*C*H$_3$ compound **7**), 25.3 (s; OCO*C*H$_3$ compound **6**), 25.0 (s; OCO*C*H$_3$ compound **7**), 24.8 (s; OCO*C*H$_3$ compound **6**), 16.9 (d, ^1J(C,P) = 26.4 Hz; P*C*H$_3$ compound **6**), 14.8 ppm (d, ^1J(C,P) = 28.6 Hz; P*C*H$_3$ compound **7**). ^{31}P{^{1}H} NMR (162.0 MHz, toluene-d^8, 25 °C): δ 67.0 (d, ^2J(P,P) = 29.3 Hz; compound **7**), 62.0 (d, ^2J(P,P) = 31.3 Hz; compound **6**), 30.9 (d, ^2J(P,P) = 29.3 Hz; compound **7**), 29.9 ppm (d, ^2J(P,P) = 31.3 Hz; compound **6**).

Synthesis of [Ru(η^2-OAc)(CO)(PNN')]OAc (8) and *cis*-[Ru(η^1-OAc)$_2$(CO)(PNN')] (9) mixture. [Ru(η^1-OAc)(η^2-OAc)(CO)(PPh$_3$)$_2$] (200 mg, 0.259 mmol) was suspended in toluene (5 mL) and then the PNN' ligand (104 mg, 0.272 mmol, 1.05 equiv) was added. The mixture was refluxed for 14 h. The resulting dark grey suspension was concentrated to about 1 mL, removing the solvent under reduced pressure. Addition of diethyl ether (10 mL) afforded the precipitation of the product as a grey solid that was filtered, washed with diethyl ether (3x3 mL), *n*-heptane (4x3 mL) and *n*-pentane (2x2 mL) and dried under reduced pressure. The grey product was isolated as a mixture of **8** / **9** in a 3:1 molar ratio. Yield: 114.1 mg (70%). Anal. Calcd for $C_{30}H_{29}N_2O_5PRu$ (629.61): C, 57.23; H, 4.64; N, 4.45. Found: C, 57.33; H, 4.65; N, 4.51. ^{1}H NMR (400.1 MHz, CD$_3$OD, 25 °C): δ 9.35 (br s, 1H; *ortho*-CH of C$_5$H$_4$N compound **9**), 8.71 (br s, 1H; *ortho*-CH of C$_5$H$_4$N compound **8**), 8.08-7.09 (m, 16H; aromatic protons), 6.82 (t, ^3J(H,H) = 9.2 Hz, 1H; *meta*-CH of C$_5$H$_4$N compound **8**), 6.76 (t, ^3J(H,H) = 8.6 Hz, 1H; *meta*-CH of C$_5$H$_4$N compound **8**), 4.65-4.49 (m, 1H; NC*H*$_2$Py compound **8**), 4.47-4.37 (m, 1H; NC*H*$_2$Py compound **9**), 4.31-4.12 (m, 2H; NC*H*$_2$Py and (C$_6$H$_4$)C*H*$_2$N compounds **8** and **9**), 3.88-3.78 (m, 1H; (C$_6$H$_4$)C*H*$_2$N compound **8**), 3.56-3.46 (m, 1H; (C$_6$H$_4$)C*H*$_2$N compound **9**), 1.96 (s, 3H; OCOCH$_3$ compound **9**), 1.88 (s, 3H; OCOCH$_3$ compound **9**), 1.55 (s, 3H; OCOCH$_3$ compound **8**), 1.53 ppm (s, 3H; OCOCH$_3$ compound **9**). ^{1}H NMR (400.1 MHz, CD$_2$Cl$_2$, 25 °C): δ 10.16 (td, ^3J(H,H) = 10.7 Hz, ^4J(H,H) = 2.0 Hz, 1H; NH of **8**), 9.21 (m, 1H; *ortho*-CH of C$_5$H$_4$N compound **9**), 8.95 (br s, 1H; NH compound **9**), 8.83 (m, 1H; *ortho*-CH of C$_5$H$_4$N compound **8**), 7.95-6.83 (m, 16H; aromatic protons), 6.79 (ddd, ^3J(H,H) = 9.4 Hz, ^4J(H,H) = 1.6 Hz, ^5J(H,H) = 1.1 Hz, 1H; *meta*-CH of C$_5$H$_4$N compound **8**), 6.66-6.58 (m, 1H; *meta*-CH of C$_5$H$_4$N compound **9**), 4.59-4.49 (m, 1H; NC*H*$_2$Py compound **9**), 4.48 (dd, ^3J(H,H) = 6.5 Hz, ^4J(H,H) = 1.7 Hz, 1H; NC*H*$_2$Py compound **8**), 4.20-4.06 (m, 2H; NC*H*$_2$Py and (C$_6$H$_4$)C*H*$_2$N compound **8**), 4.04 (dd, ^3J(H,H) = 11.8 Hz, ^4J(H,H) = 4.5 Hz, 1H; (C$_6$H$_4$)C*H*$_2$N compound **9**), 3.89 (t, ^3J(H,H) = 11.3 Hz, 1H; (C$_6$H$_4$)C*H*$_2$N compound **8**), 2.05 (s, 3H; OCOCH$_3$ compound **9**), 1.89 (s, 3H; OCOCH$_3$ compound **8**), 1.48 (s, 3H; OCOCH$_3$ compound **8**), 1.36 ppm (s, 3H; OCOCH$_3$ compound **9**). ^{13}C{^{1}H} NMR (100.6 MHz, CD$_2$Cl$_2$, 25 °C): δ 207.2 (d, ^2J(C,P) = 14.3 Hz; CO compound **9**), 205.5 (d, ^2J(C,P) = 17.6 Hz; CO

compound **8**), 181.7 (s; O*C*OCH$_3$ compound **8**), 181.3 (s; O*C*OCH$_3$ compound **9**), 177.3 (s; O*C*OCH$_3$ compound **9**), 176.4 (s; O*C*OCH$_3$ compound **8**), 160.9 (s; N*C*CH$_2$ of C$_5$H$_4$N compound **9**), 159.1 (d, ^3J(C,P) = 1.5 Hz; N*C*CH$_2$ of C$_5$H$_4$N compound **8**), 149.9 (s; N*C*H of C$_5$H$_4$N compound **9**), 149.6 (s; N*C*H of C$_5$H$_4$N compound **8**), 140.0 (d, ^1J(C,P) = 15.4 Hz; *ipso*-Ph), 138.5-123.7 (m; aromatic carbon atoms), 124.3 (d, ^3J(C,P) = 2.9 Hz; *meta-C*H of C$_5$H$_4$N compound **8**), 123.7 (d, ^3J(C,P) = 2.6 Hz; *meta-C*H of C$_5$H$_4$N compound **9**), 120.9 (d, ^3J(C,P) = 1.5 Hz; *meta-C*H of C$_5$H$_4$N compound **8**), 120.6 (d, ^3J(C,P) = 1.5 Hz; *meta-C*H of C$_5$H$_4$N compound **9**), 61.7 (s; N*C*H$_2$Py compound **8**), 60.1 (d, ^3J(C,P) = 5.1 Hz; (C$_6$H$_4$)*C*H$_2$N compound **8**), 57.1 (s; N*C*H$_2$Py compound **9**), 54.8 (d, ^3J(C,P) = 5.6 Hz; (C$_6$H$_4$)*C*H$_2$N compound **9**), 24.7 (s; OCO*C*H$_3$ compound **9**), 24.5 (s; OCO*C*H$_3$ compound **8**), 23.9 (s; OCO*C*H$_3$ compound **9**), 23.3 ppm (s; OCO*C*H$_3$ compound **8**). ^{31}P{^{1}H} NMR (162.0 MHz, CD$_3$OD, 25 °C): δ 55.1 (s; 25% compound **9**), 42.5 ppm (s; 75% compound **8**). ^{31}P{^{1}H} NMR (162.0 MHz, CD$_2$Cl$_2$, 25 °C): δ 54.9 (s; 25% compound **9**), 43.5 ppm (s; 75% compound **8**).

Synthesis of *cis*-[Ru(η^1-OAc)$_2$(CO)(PNN(C$_2$)$_{imine}$)] (10). [Ru(η^1-OAc)(η^2-OAc)(CO)(PPh$_3$)$_2$] (200 mg, 0.259 mmol) was suspended in toluene (5 mL) and added of the PNN(C$_2$)$_{imine}$ ligand (107.4 mg, 0.272 mmol, 1.05 equiv). The mixture was refluxed for 2 h. The resulting yellow solution was concentrated to about 1 mL, removing the solvent under reduced pressure. Addition of *n*-heptane (10 mL) afforded the precipitation of the product as a yellow solid that was filtered, washed with *n*-heptane (3x4 mL), diethyl ether (3x3 mL), and *n*-pentane (2x2 mL) and dried under reduced pressure. Yield: 141.4 mg (87%). Anal. Calcd for C$_{31}$H$_{29}$N$_2$O$_5$PRu (641.63): C, 58.03; H, 4.56; N, 4.37. Found: C, 58.10; H, 4.51; N, 4.40. ^{1}H NMR (400.1 MHz, CD$_2$Cl$_2$, 25 °C): δ 9.04-8.97 (m, 1H; RuNC*H* of C$_4$H$_3$N$_2$), 8.40 (br s, 1H; CH$_2$N=C*H*), 7.89 (ddd, ^3J(H,H) = 8.0 Hz, ^4J(H,H) = 1.4 Hz, ^3J(H,H) = 1.2 Hz, 2H; aromatic protons), 7.78 (td, ^3J(H,H) = 7.6 Hz, ^4J(H,H) = 1.5 Hz, 1H; aromatic proton), 7.73-7.59 (m, 3H; aromatic protons), 7.56-7.41 (m, 6H; aromatic protons), 7.29 (m, 2H; aromatic protons), 7.24-7.14 (m, 1H; aromatic proton), 6.92 (t, ^3J(H,H) = 8.7 Hz, 1H; *meta*-CH of C$_5$H$_4$N), 4.21-4.09 (m, 2H; NCH$_2$), 3.78 (ddd, ^3J(H,H) = 16.2 Hz, ^3J(H,H) = 7.9 Hz, ^3J(H,H) = 3.3 Hz, 1H; NCH$_2$C*H*$_2$Py), 3.48 (ddt, ^3J(H,H) = 13.3 Hz, ^4J(H,H) = 6.6 Hz, ^5J(H,H) = 3.2 Hz, 1H; NCH$_2$C*H*$_2$Py), 1.58 (s, 3H; OCOCH$_3$), 1.29 ppm (s, 3H; OCOCH$_3$). ^{13}C{^{1}H} NMR (100.6 MHz, CD$_2$Cl$_2$, 25 °C): δ 204.6 (d, ^2J(CP) = 16.9 Hz; CO), 176.0 (s; O*C*OCH$_3$), 175.7 (s; O*C*OCH$_3$), 168.0 (d, ^3J(C,P) = 5.9 Hz; CH$_2$N=*C*H), 160.9 (s; N*C*CH$_2$ of C$_5$H$_4$N), 151.2 (s; N*C*H of C$_5$H$_4$N), 137.7 (d, ^3J(C,P) = 14.7 Hz; *ipso*-Ph), 137.9-126.3 (m; aromatic carbon atoms), 124.7 (d, ^4J(C,P) = 2.9 Hz; *meta-C*H of C$_5$H$_4$N), 122.4 (d, ^4J(C,P) = 2.2 Hz; *meta-C*H of C$_5$H$_4$N), 63.1 (NCH$_2$*C*H$_2$), 36.7 (N*C*H$_2$CH$_2$), 23.9 (s; OCO*C*H$_3$), 23.2 ppm (s; OCO*C*H$_3$). ^{31}P{^{1}H} NMR (162.0 MHz, CD$_2$Cl$_2$, 25 °C): δ 48.4 ppm (s).

Typical procedure for hydrogenation of methyl decanoate. The hydrogenation reactions for the reduction of methyl decanoate were performed in an 8-vessel Biotage® Endeavor™

apparatus employing ruthenium PNN acetates **1-10**, *trans*-[$RuCl_2$(PNN')(PPh_3)] and *trans*-[$RuCl_2$(PNN^G)(PPh_3)]. The catalysts were added to the reactor from appropriate volumes of stock solutions of the complexes prepared by dissolving the required mass of the catalyst in CH_2Cl_2 (1.0-4.0 mL), aliquots of which were dispensed into 8 different vials. The solvent was then removed under the flow of N_2. To each of these containers, NaOMe or NaOEt (50 mol% respect to the substrate) was added, followed by the substrate (10-20 mmol, 2.1-4.3 mL). For the experiments carried out with solvent, toluene was added (one volume respect to the substrate, 2.1-4.3 mL). The vials were then sealed inside of the instrument, where the vessels were purged 5 times with N_2 before running an automated purging cycle with H_2. Subsequently, the vessels were charged with H_2 (27.5 bar), heated to 40 or 90 °C and stirred (600 rpm) for the required time (16 h). The reactions were then cooled to room temperature, depressurized, and purged with nitrogen five times. Two work-up procedures were used, prior to analysis: a) adding EtOH (4 mL), followed by 1.0 M HCl (3 mL) or b) adding of MTBE (3 mL), followed by 1.0 M HCl (3 mL) and extraction of the product in the organic phase. The solutions obtained were sampled, diluted with EtOH or MTBE (1 mL), depending on the work-up used, and analyzed by GC.

Typical procedure for hydrogenation of methyl and ethyl esters. The hydrogenation reductions of a series of methyl and ethyl esters were performed in an 8-vessel Biotage® Endeavor™ apparatus employing the catalyst **3** and *trans*-[$RuCl_2$(PNN^G)(PPh_3)], following the procedure used for methyl decanoate.

Typical procedure for base-free hydrogenation of aldehydes. The hydrogenation reductions of aldehydes (2 mmol) were performed in an 8-vessel Biotage® Endeavor™ apparatus following a procedure similar to that used for the ester HY, with the catalysts **1**-**4**, **8**/**9** (2.0 µmol) and employing MeOH (4 mL) as solvent (EtOH, 2-propanol and Me-THF were also used). The catalytic runs were conducted at 90 °C for 16 h with a H_2 pressure of 27.5 bar by stirring the mixtures at 600 rpm without the use of bases. The substrate solution was 0.5 M and the S/C molar ratio was 1000. A similar method was applied for the reactions with S/C of 5000, using the appropriate amount of catalysts. The reaction vessels were then cooled to room temperature vented and purged three times with N_2. A drop of the reaction mixture was diluted with 1 mL of methanol and analyzed by GC.

Single-Crystal X-ray Crystallography Structure Determination of Compound 10. Single crystals of complex **10** were obtained by slow evaporation of a concentrated solution of the species in CH_2Cl_2. X-ray diffraction data were collected at 100 K on an X-ray single crystal Bruker D8 VENTURE Duo three-angle diffractometer with an IMS microsource with MoKα radiation (λ = 0.71073 Å) using the APEX4 software. For additional details for collection and refining of data, see pages S130-S133.

Accession Codes. CCDC 2553530 contains the supplementary crystallographic data for this paper. These data can be obtained free of charge via www.ccdc.cam.ac.uk/data_request/cif, or by emailing data_request@ccdc.cam.ac.uk, or by contacting The Cambridge Crystallographic Data Centre, 12 Union Road, Cambridge CB2 1EZ, UK; fax: +44 1223 336033.

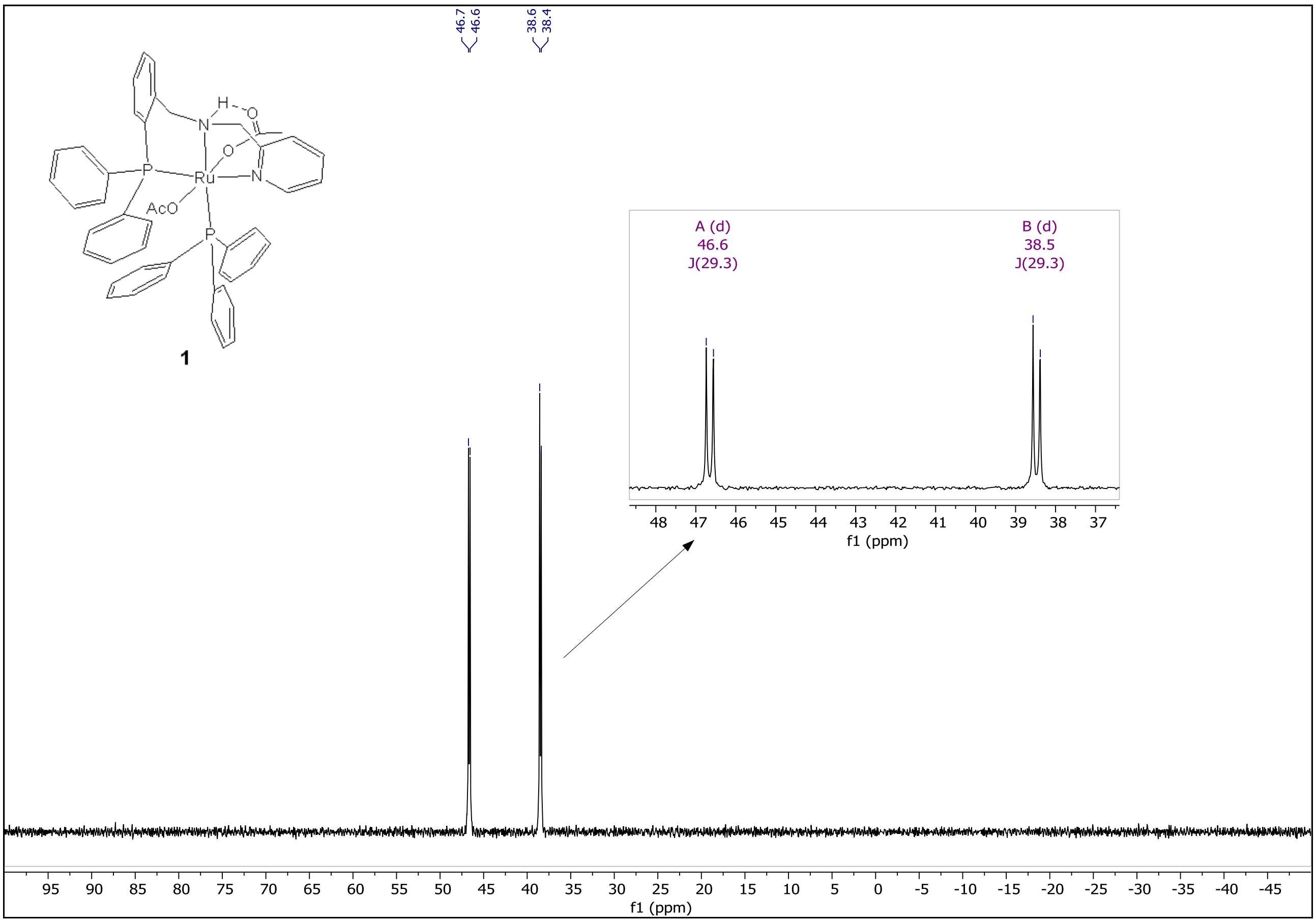

Figure S1. $^{31}P\{^1H\}$ NMR spectrum (162.0 MHz) of *trans*-[Ru(η^1-OAc)$_2$(PNN)(PPh$_3$)] (**1**) in toluene-d^8 at 25 °C.

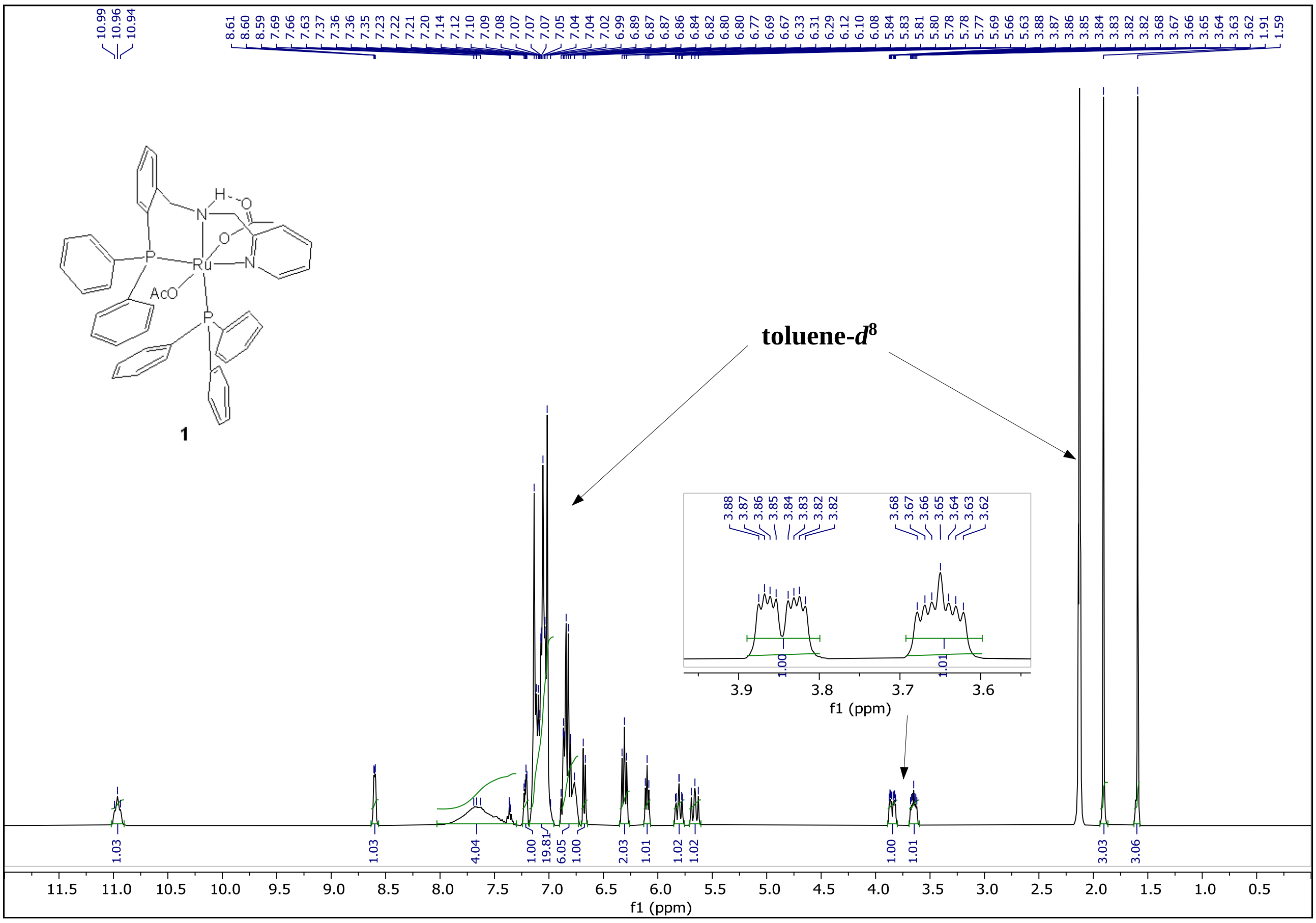

Figure S2. ^1H NMR spectrum (400.1 MHz) of *trans*-[Ru(η^1-OAc)$_2$(PNN)(PPh$_3$)] (**1**) in toluene-d^8 at 25 °C.

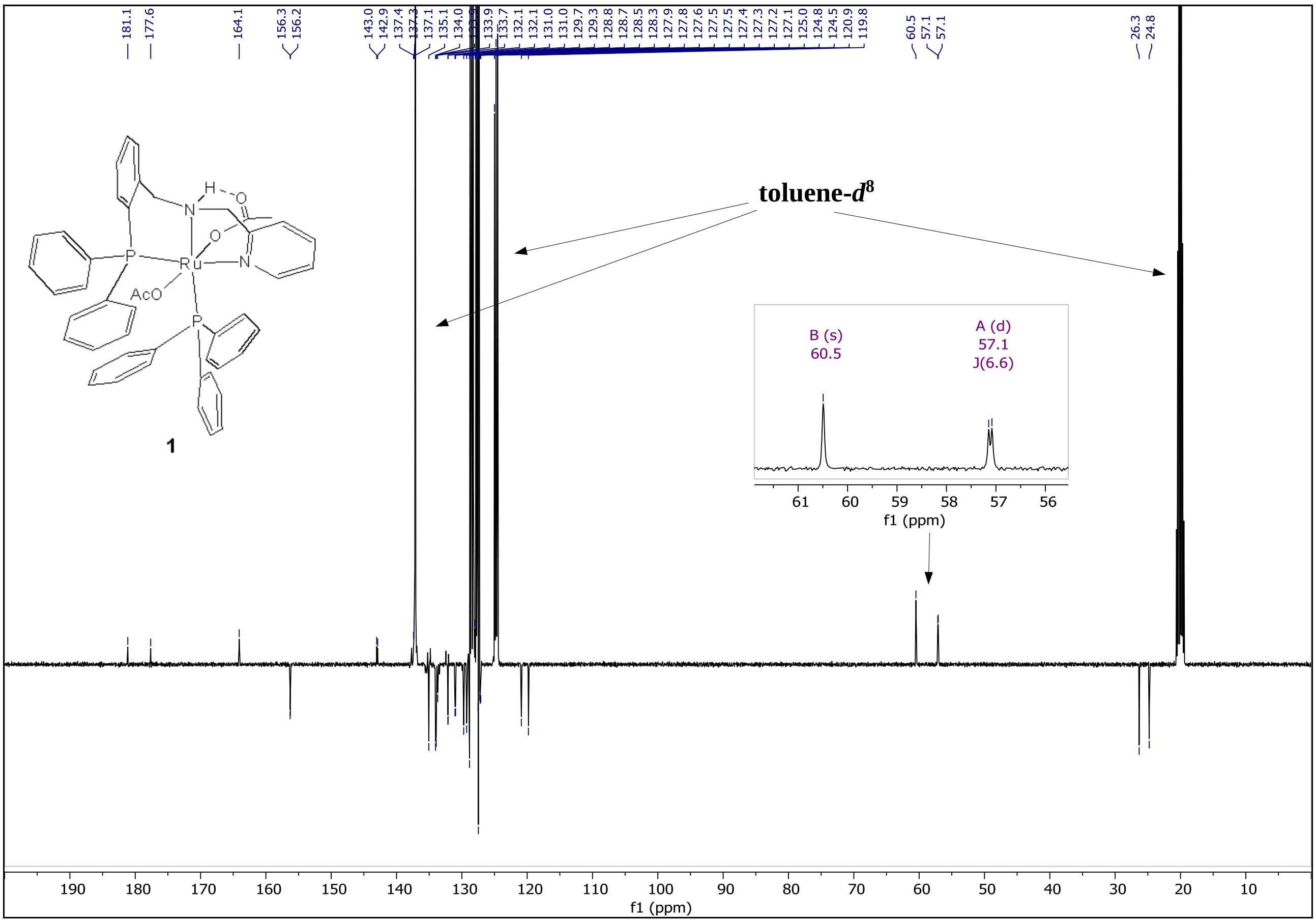

Figure S3. $^{13}C\{^{1}H\}$ DEPTQ NMR spectrum (100.6 MHz) of *trans*-[Ru(η^1-OAc)$_2$(PNN)(PPh$_3$)] (**1**) in toluene-d^8 at 25 °C.

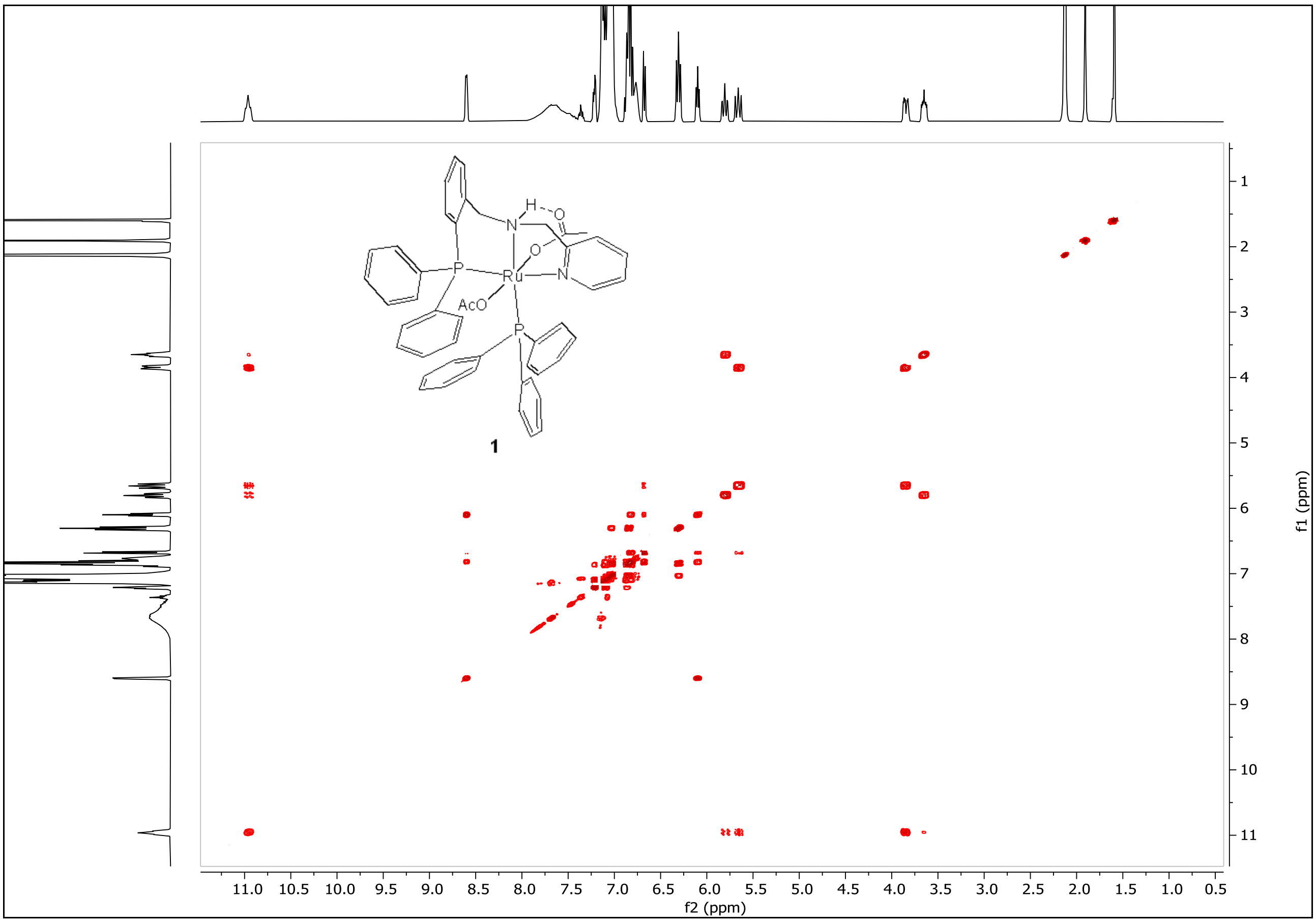

Figure S4. ^1H-^1H COSY 2D NMR spectrum of *trans*-[Ru(η^1-OAc)$_2$(PNN)(PPh$_3$)] (**1**) in toluene-d^8 at 25 °C.

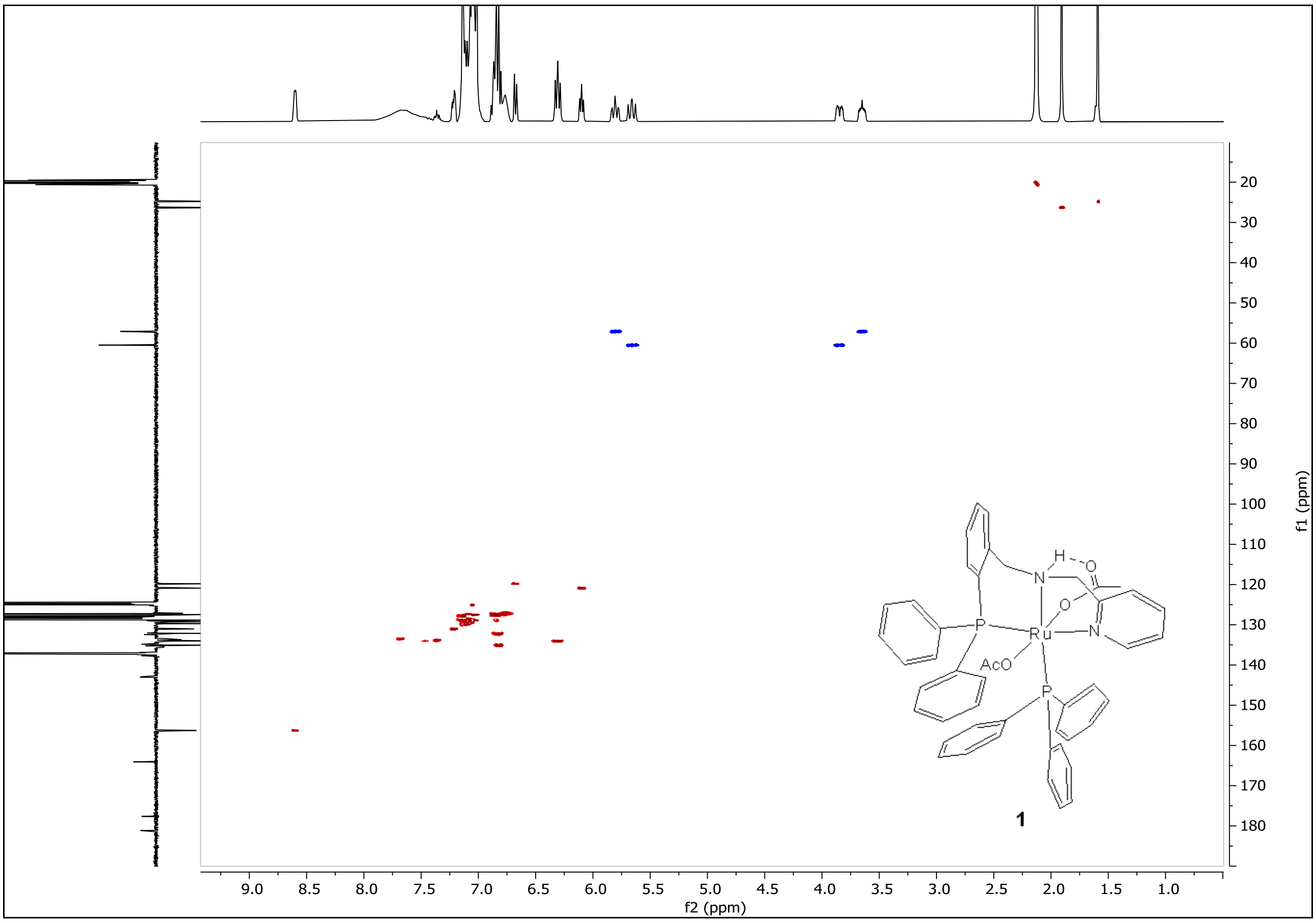

Figure S5. ^{1}H-^{13}H HSQC 2D NMR spectrum of *trans*-[Ru(η^1-OAc)$_2$(PNN)(PPh_3)] (**1**) in toluene-d^8 at 25 °C.

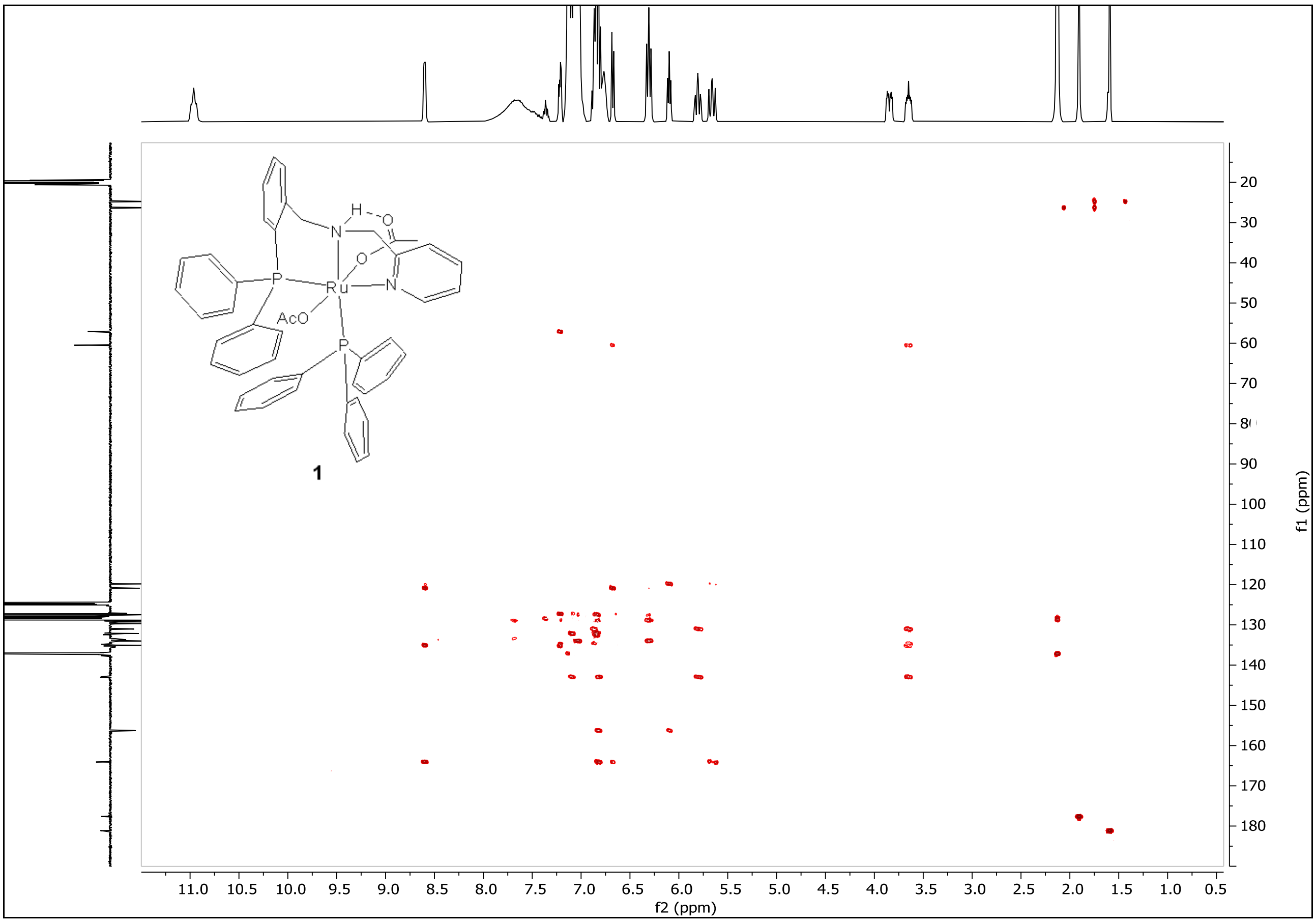

Figure S6. ^{1}H-^{13}H HMBC 2D NMR spectrum of *trans*-[Ru(η^1-OAc)$_2$(PNN)(PPh$_3$)] (**1**) in toluene-d^8 at 25 °C.

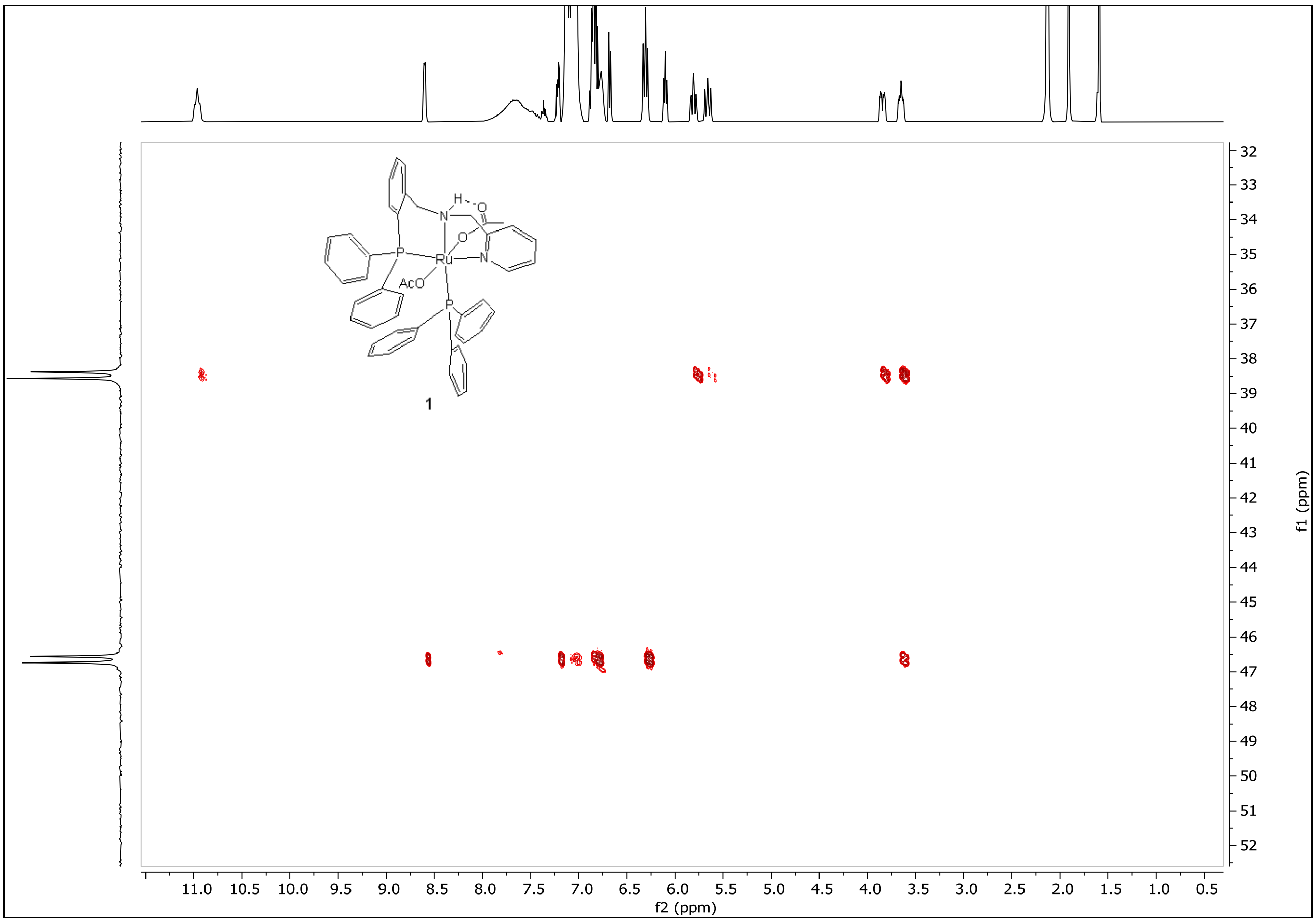

Figure S7. ^{1}H-^{31}P HMBC 2D NMR spectrum of *trans*-[Ru(η^1-OAc)$_2$(PNN)(PPh$_3$)] (**1**) in toluene-d^8 at 25 °C.

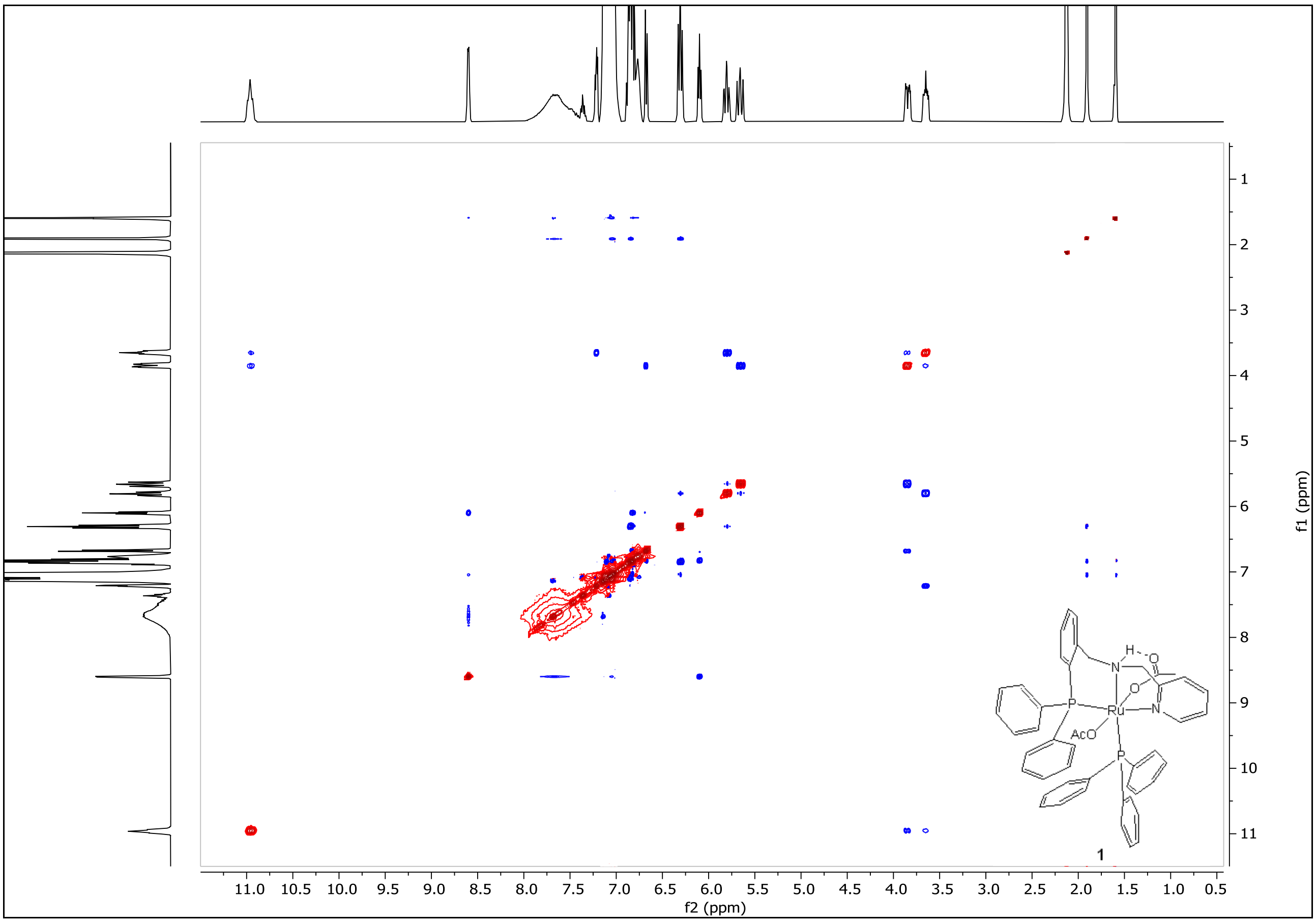

1

Figure S8. ^1H-^1H NOESY 2D NMR spectrum of *trans*-[Ru(η^1-OAc)$_2$(PNN)(PPh_3)] (**1**) in toluene-*d*^{8} at 25 °C.

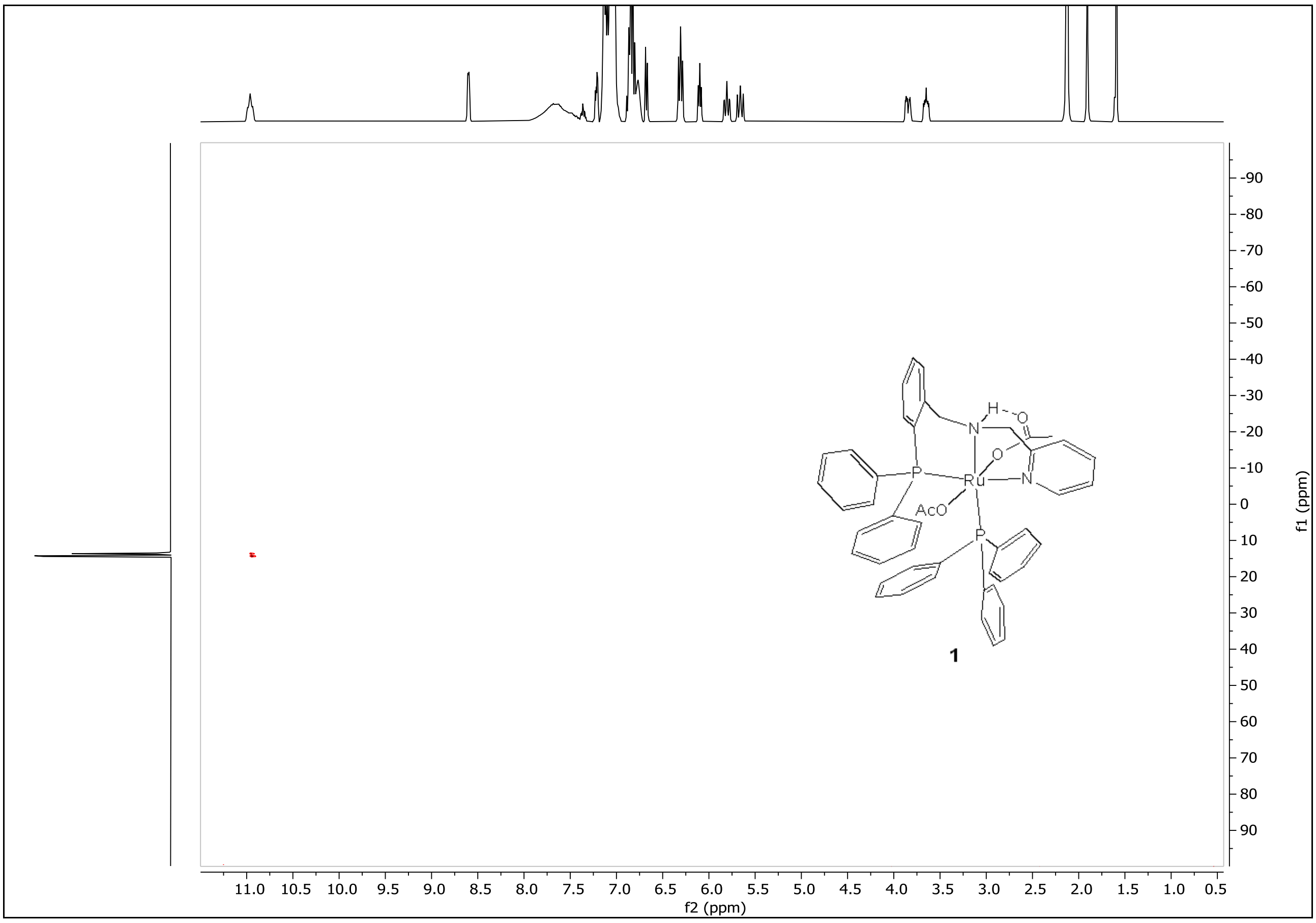

Figure S9. ^{1}H-^{15}N HSQC 2D NMR spectrum of *trans*-[Ru(η^1-OAc)$_2$(PNN)(PPh$_3$)] (**1**) in toluene-*d*^{8} at 25 °C.

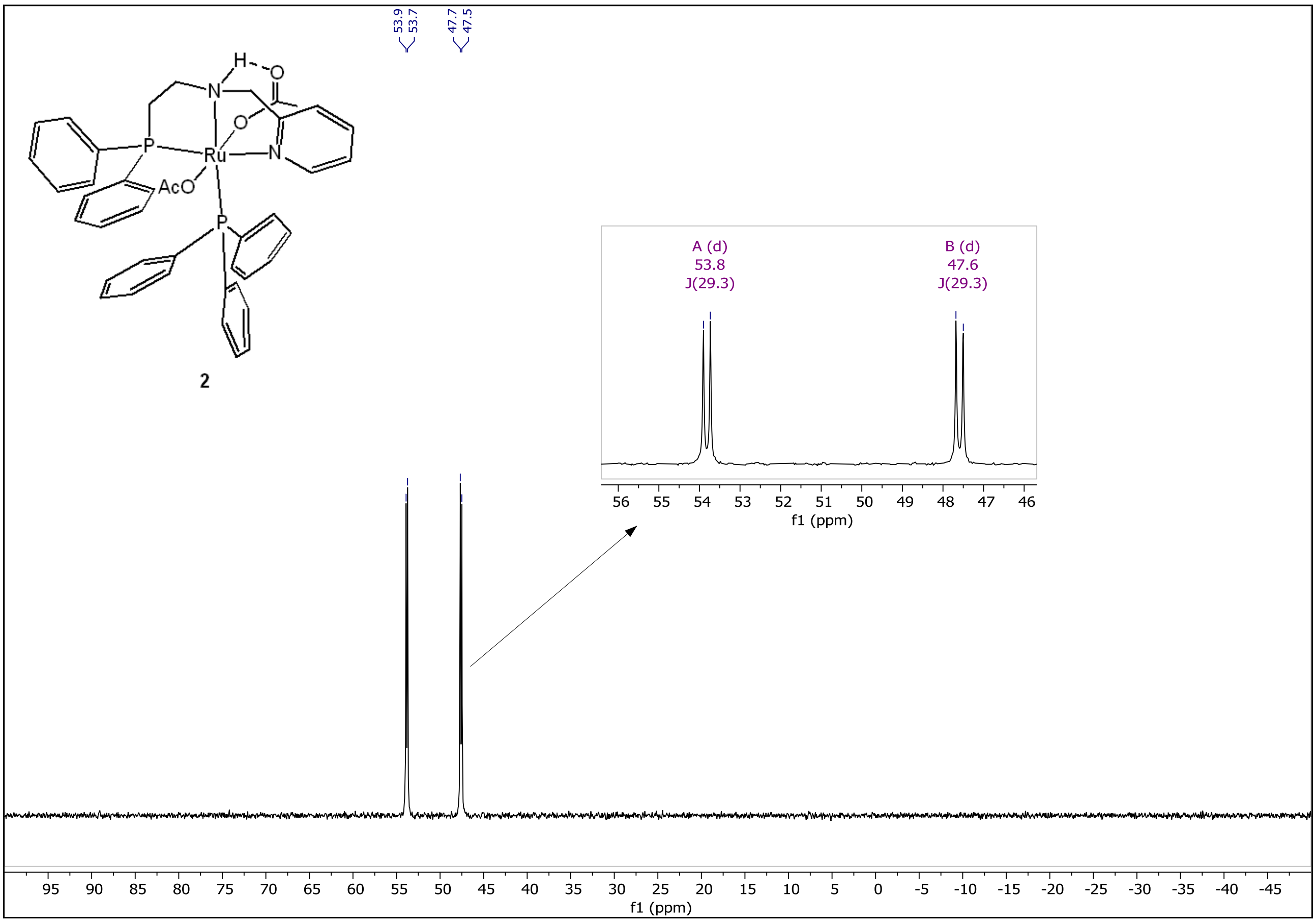

Figure S10. $^{31}P\{^1H\}$ NMR spectrum (162.0 MHz) of *trans*-[Ru(η^1-OAc)$_2$(PNN^G)(PPh$_3$)] (**2**) in toluene-d^8 at 25 °C.

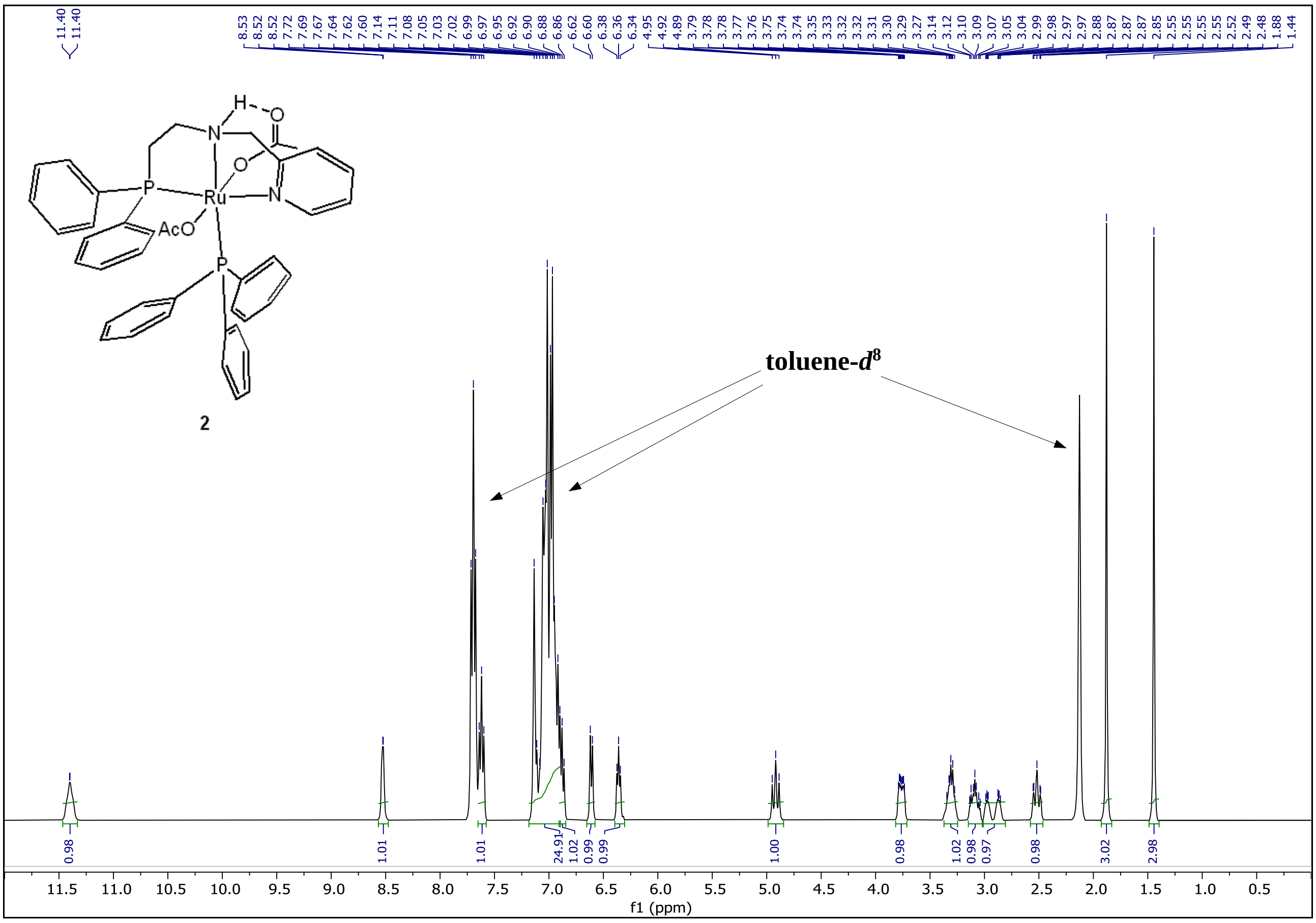

Figure S11. ^{1}H NMR spectrum (400.1 MHz) of *trans*-[Ru(η^1-OAc)$_2$(PNN^G)(PPh$_3$)] (**2**) in toluene-d^8 at 25 °C.

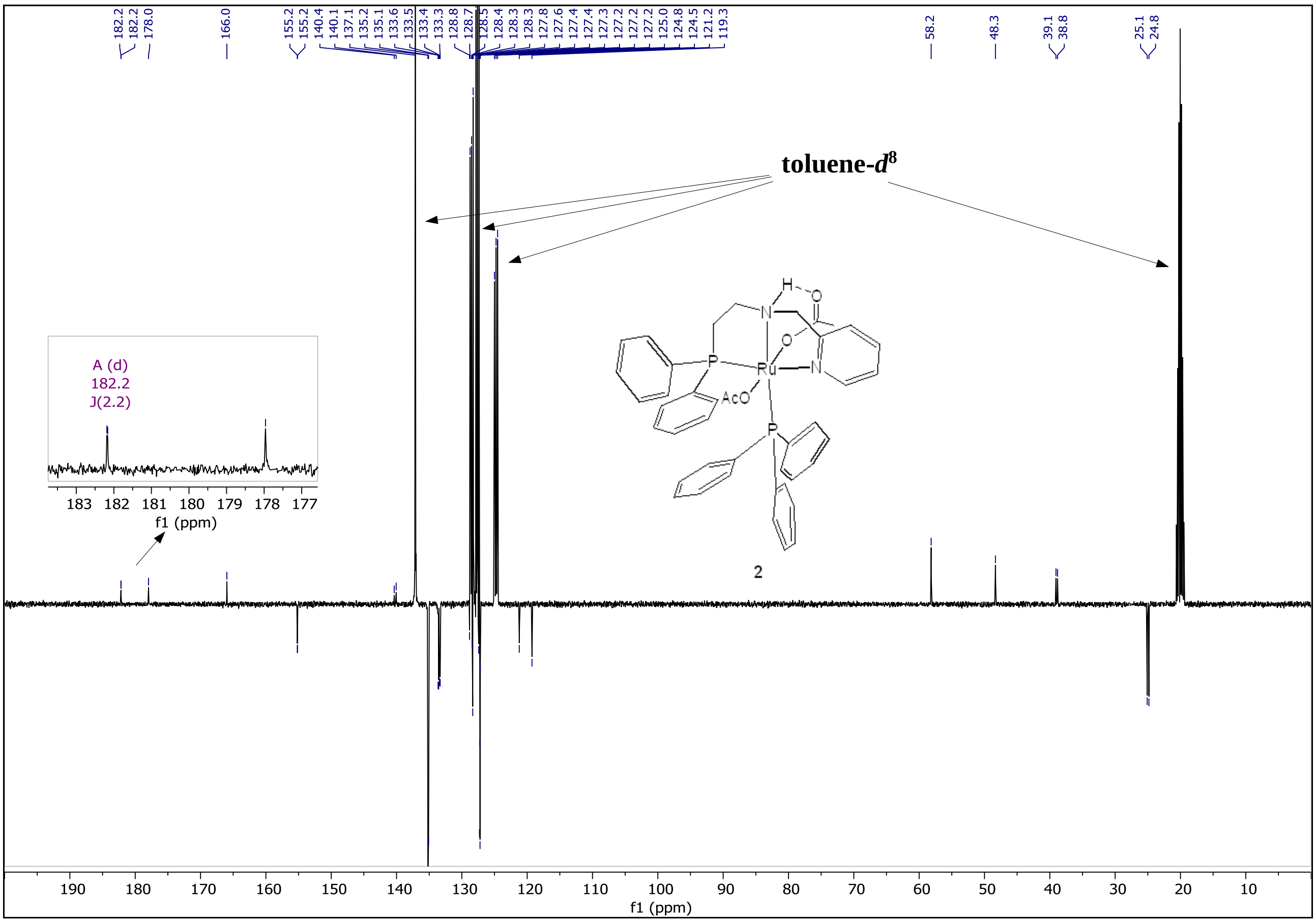

Figure S12. $^{13}C\{^1H\}$ DEPTQ NMR spectrum (100.6 MHz) of *trans*-[Ru(η^1-OAc)$_2$(PNN^G)(PPh$_3$)] (**2**) in toluene-d^8 at 25 °C.

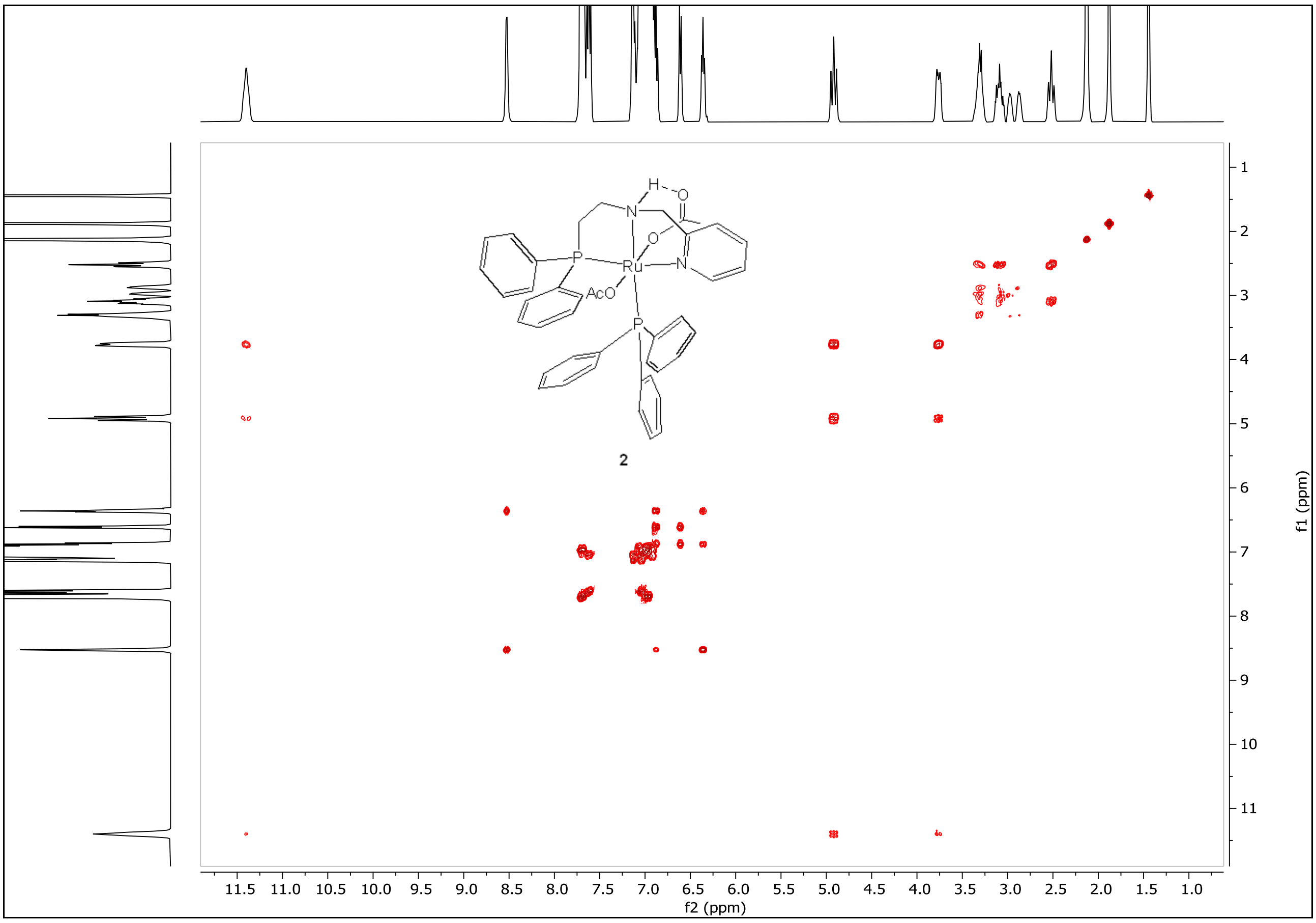

Figure S13. ^{1}H-^{1}H COSY 2D NMR spectrum of *trans*-[Ru(η^{1}-OAc)$_2$(PNN^{G})(PPh$_3$)] (**2**) in toluene-*d*^{8} at 25 °C.

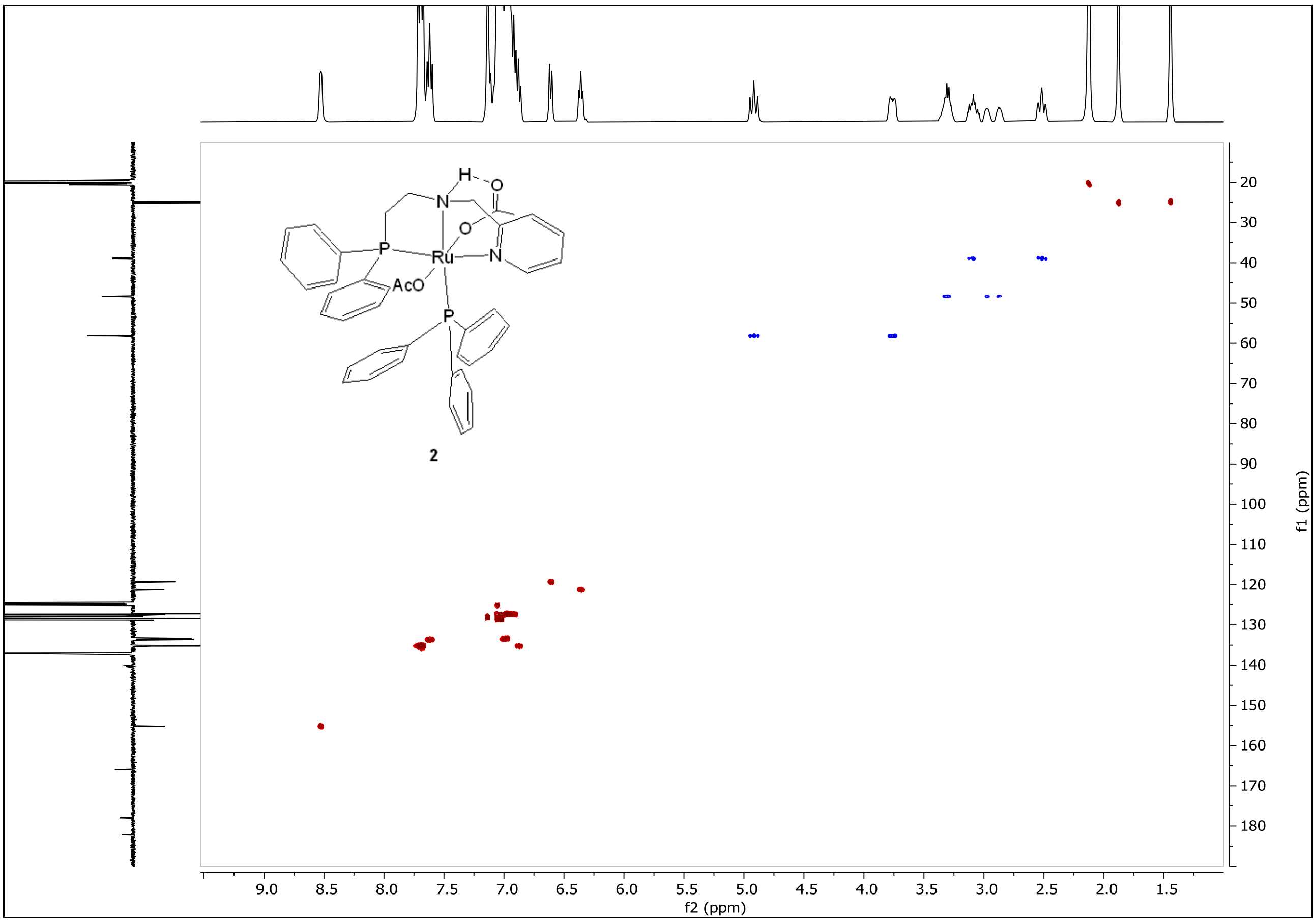

Figure S14. ^{1}H-^{13}C HSQC 2D NMR spectrum of *trans*-[Ru(η^1-OAc)$_2$(PNN^G)(PPh$_3$)] (**2**) in toluene-d^8 at 25 °C.

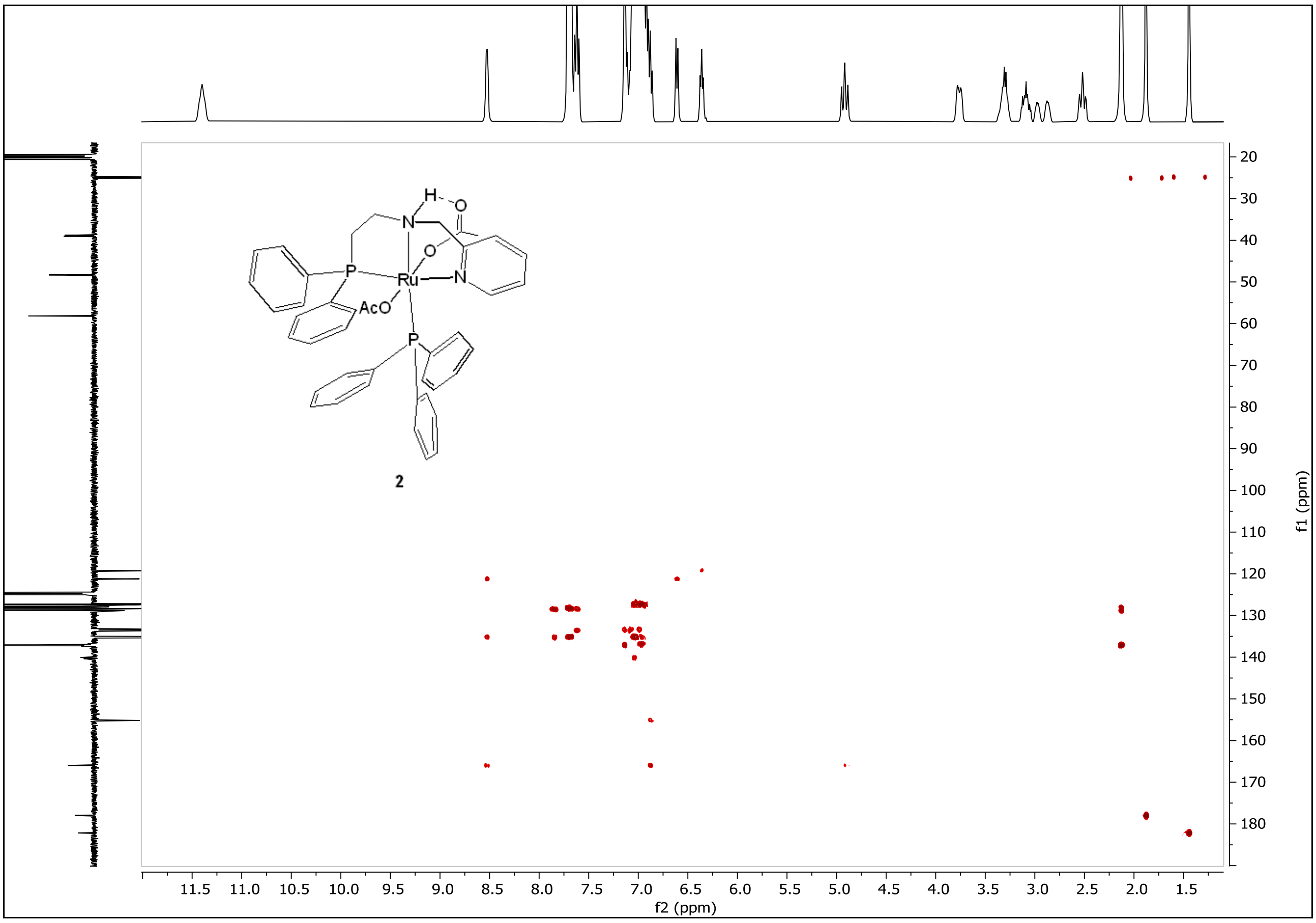

Figure S15. ^{1}H-^{13}C HMBC 2D NMR spectrum of *trans*-[Ru(η^{1}-OAc)$_2$(PNN^{G})(PPh$_3$)] (**2**) in toluene-*d*^{8} at 25 °C.

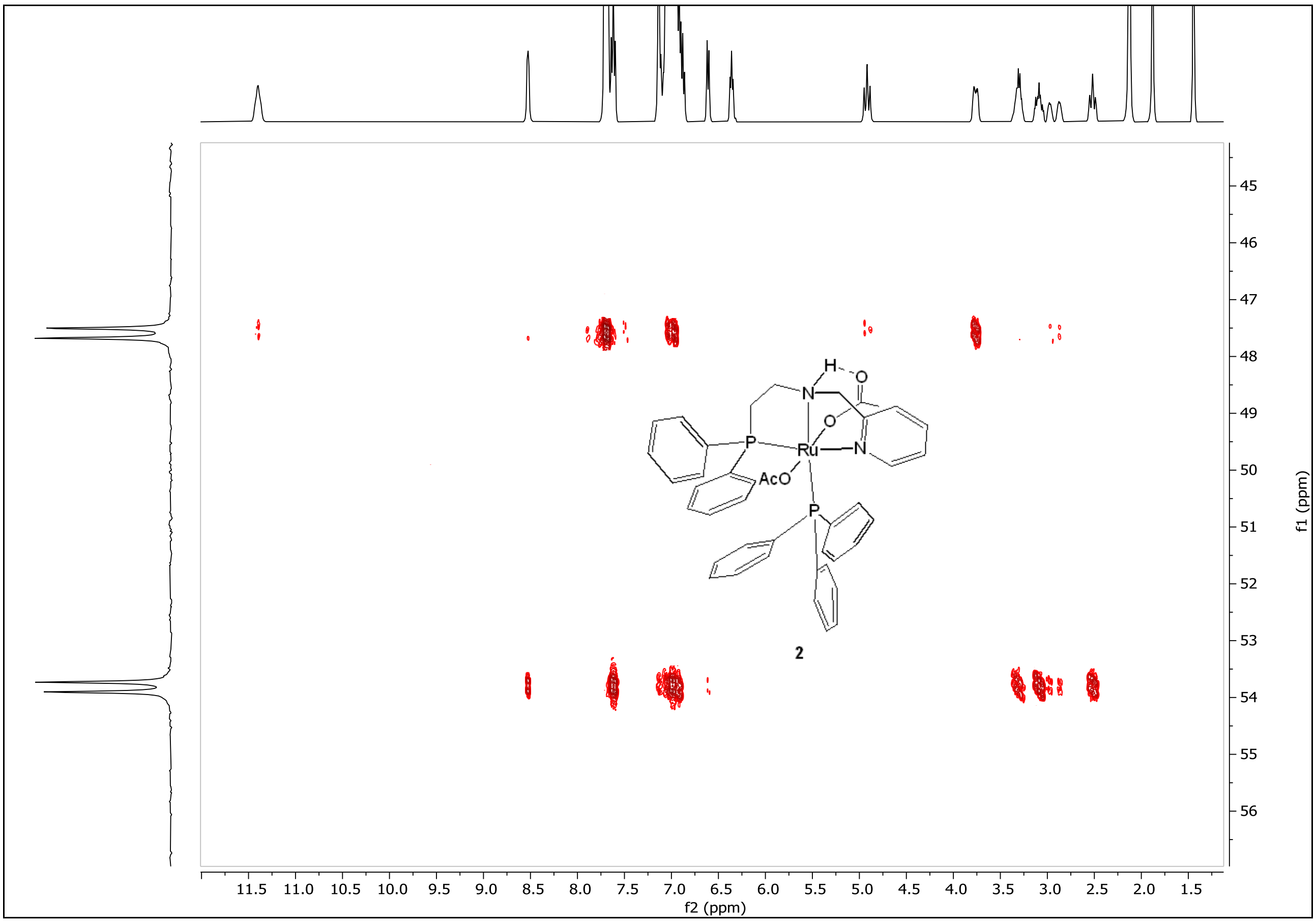

Figure S16. ^1H-^{31}P HMBC 2D NMR spectrum of *trans*-[Ru(η^1-OAc)$_2$(PNN^G)(PPh$_3$)] (**2**) in toluene-*d*^{8} at 25 °C.

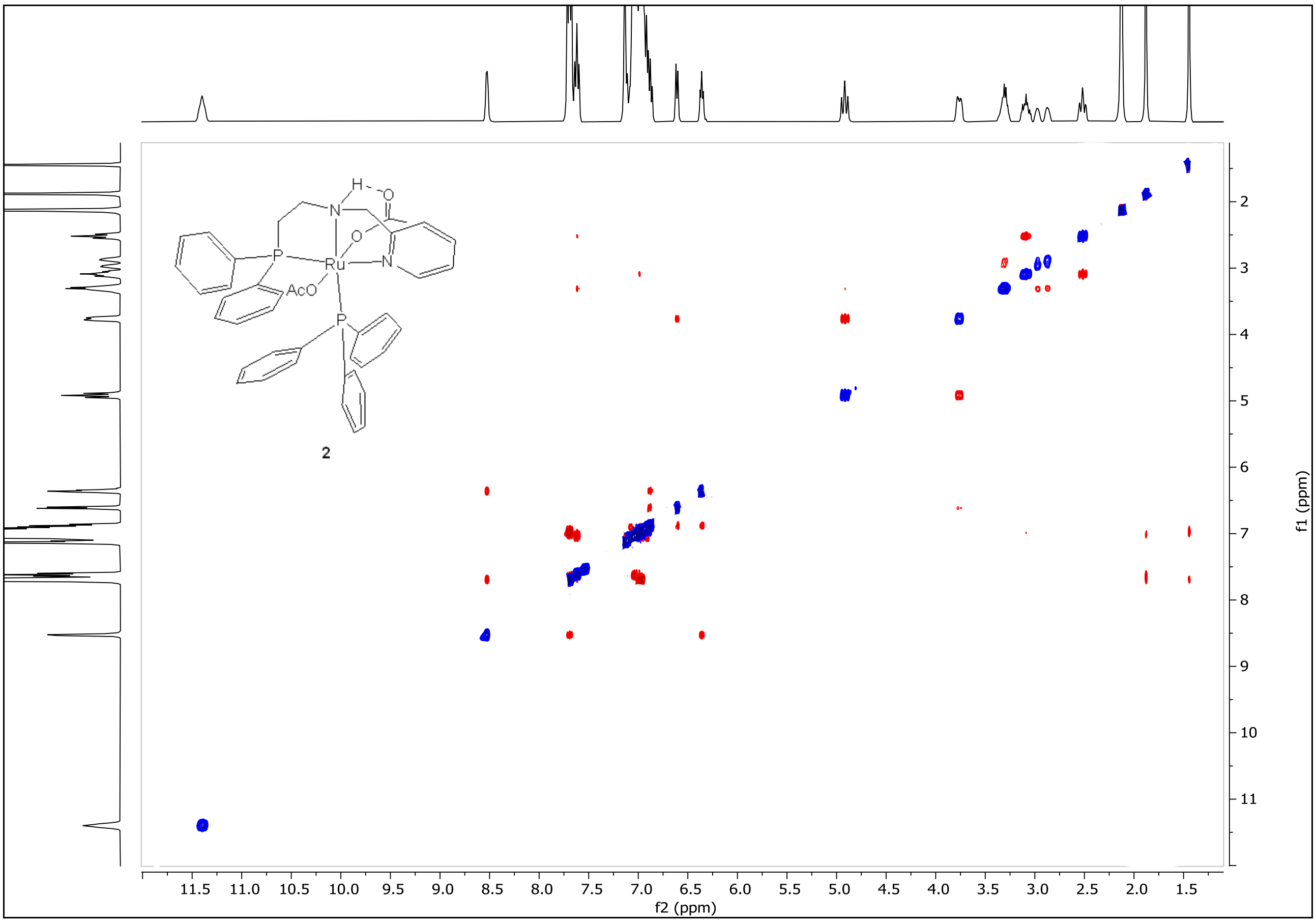

Figure S17. ^{1}H-^{1}H NOESY 2D NMR spectrum of *trans*-[Ru(η^1-OAc)$_2$(PNN^G)(PPh$_3$)] (**2**) in toluene-d^8 at 25 °C.

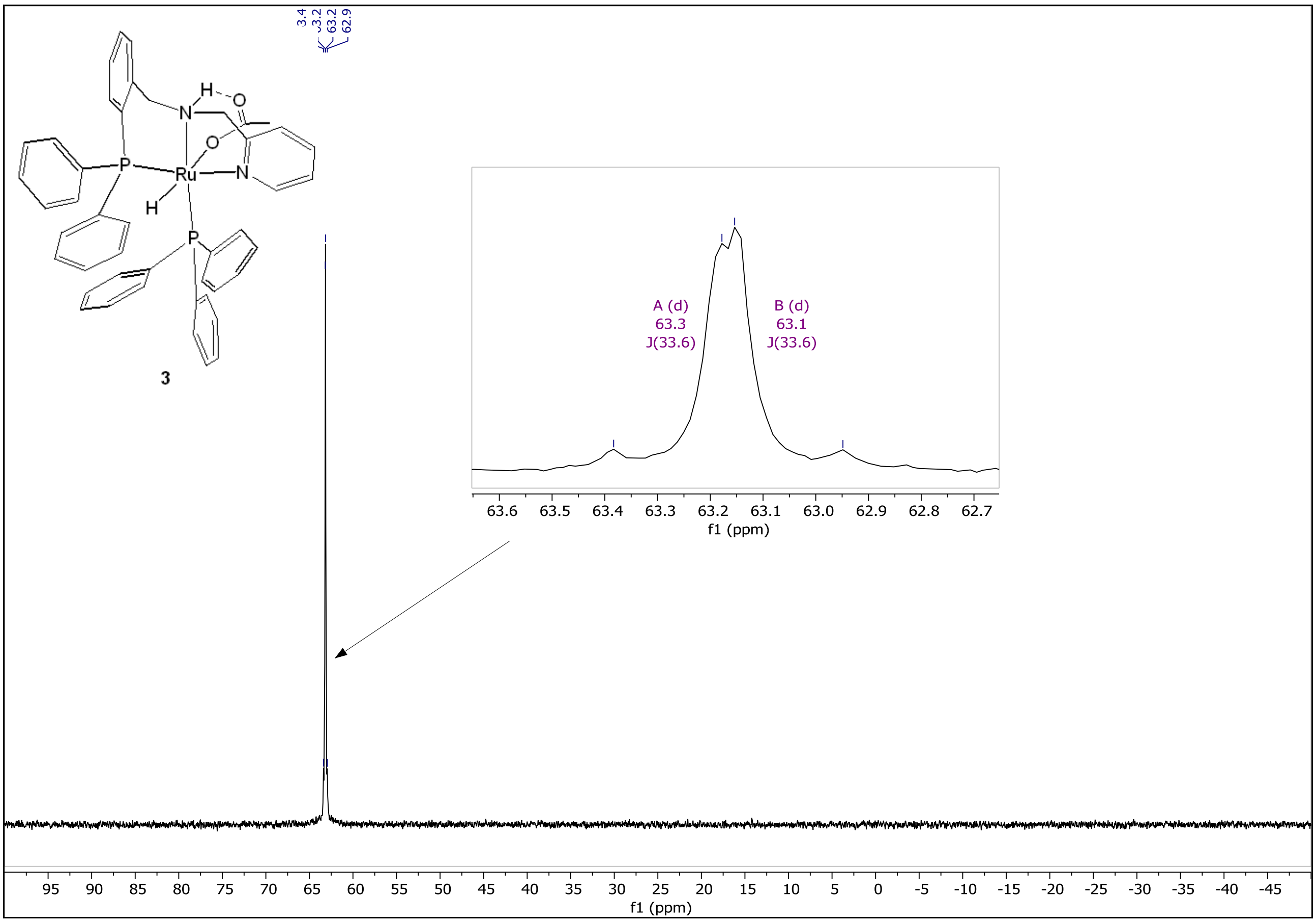

Figure S18. $^{31}P\{^{1}H\}$ NMR spectrum (162.0 MHz) of *trans*-[RuH(η^1-OAc)(PNN)(PPh_3)] (**3**) in toluene-d^8 at 25 °C.

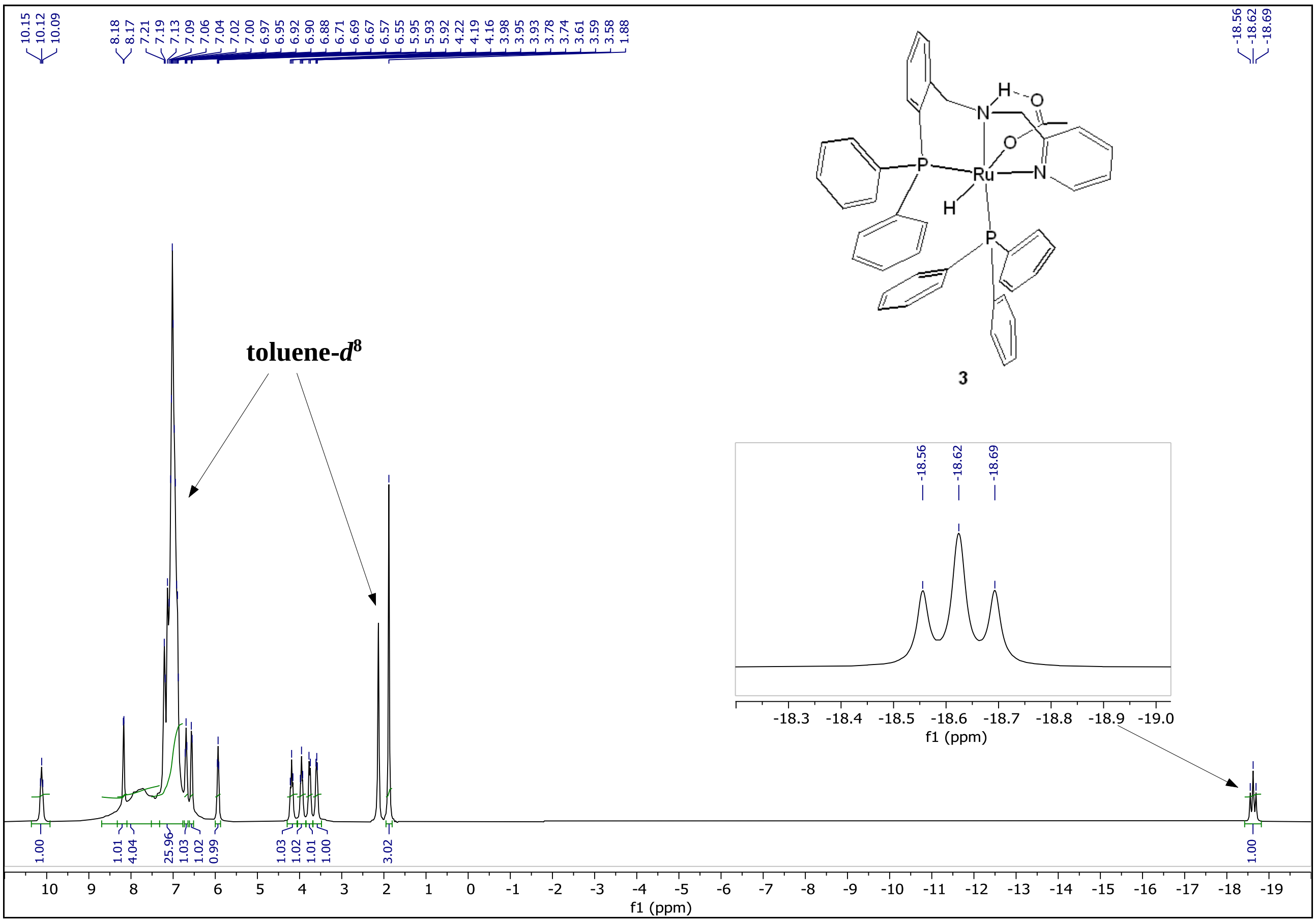

Figure S19. ^{1}H NMR spectrum (400.1 MHz) of *trans*-[RuH(η^1-OAc)(PNN)(PPh_3)] (**3**) in toluene-d^8 at 25 °C.

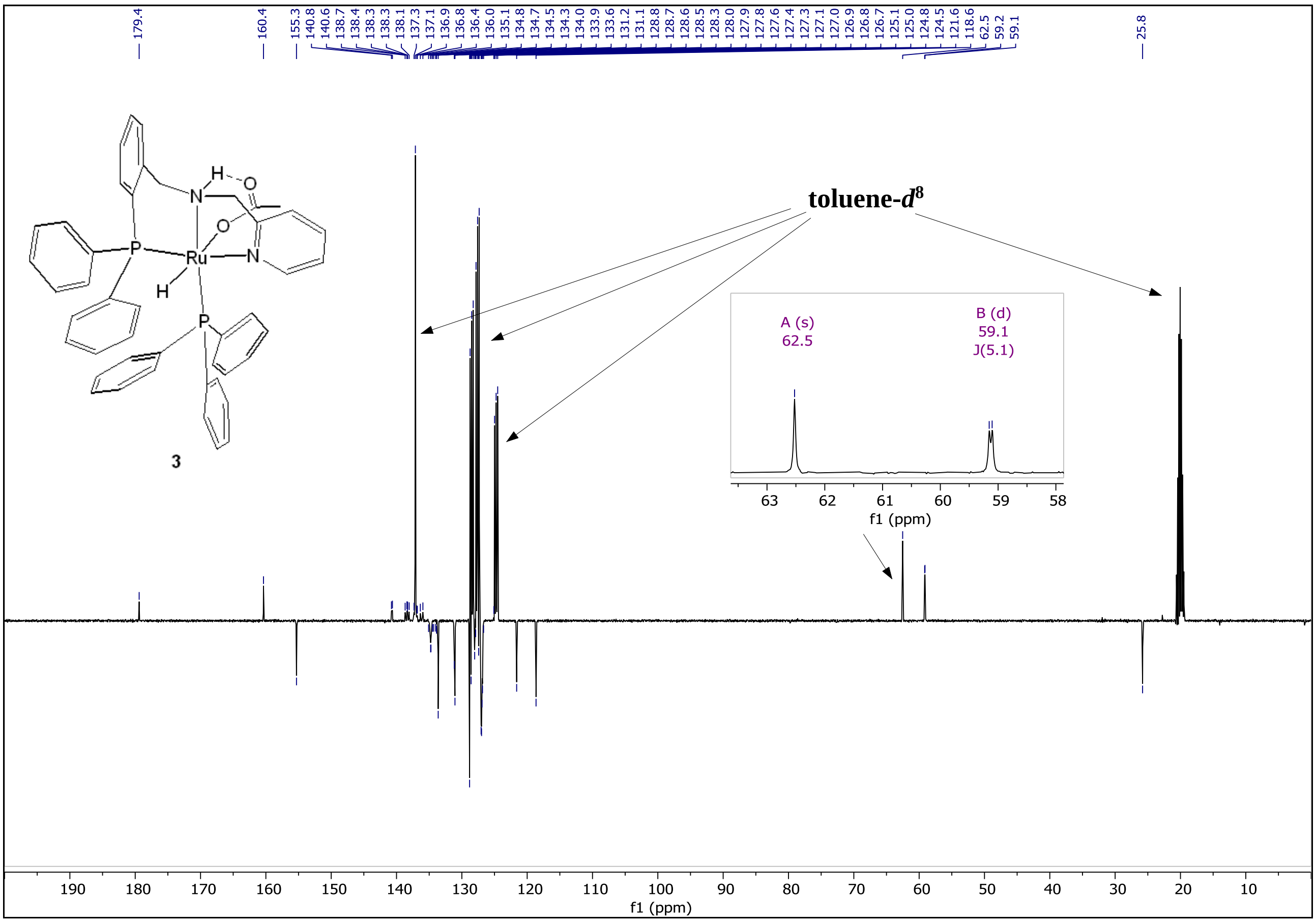

Figure S20. $^{13}C\{^{1}H\}$ DEPTQ NMR spectrum (100.6 MHz) of *trans*-[RuH(η^1-OAc)(PNN)(PPh_3)] (**3**) in toluene-d^8 at 25 °C.

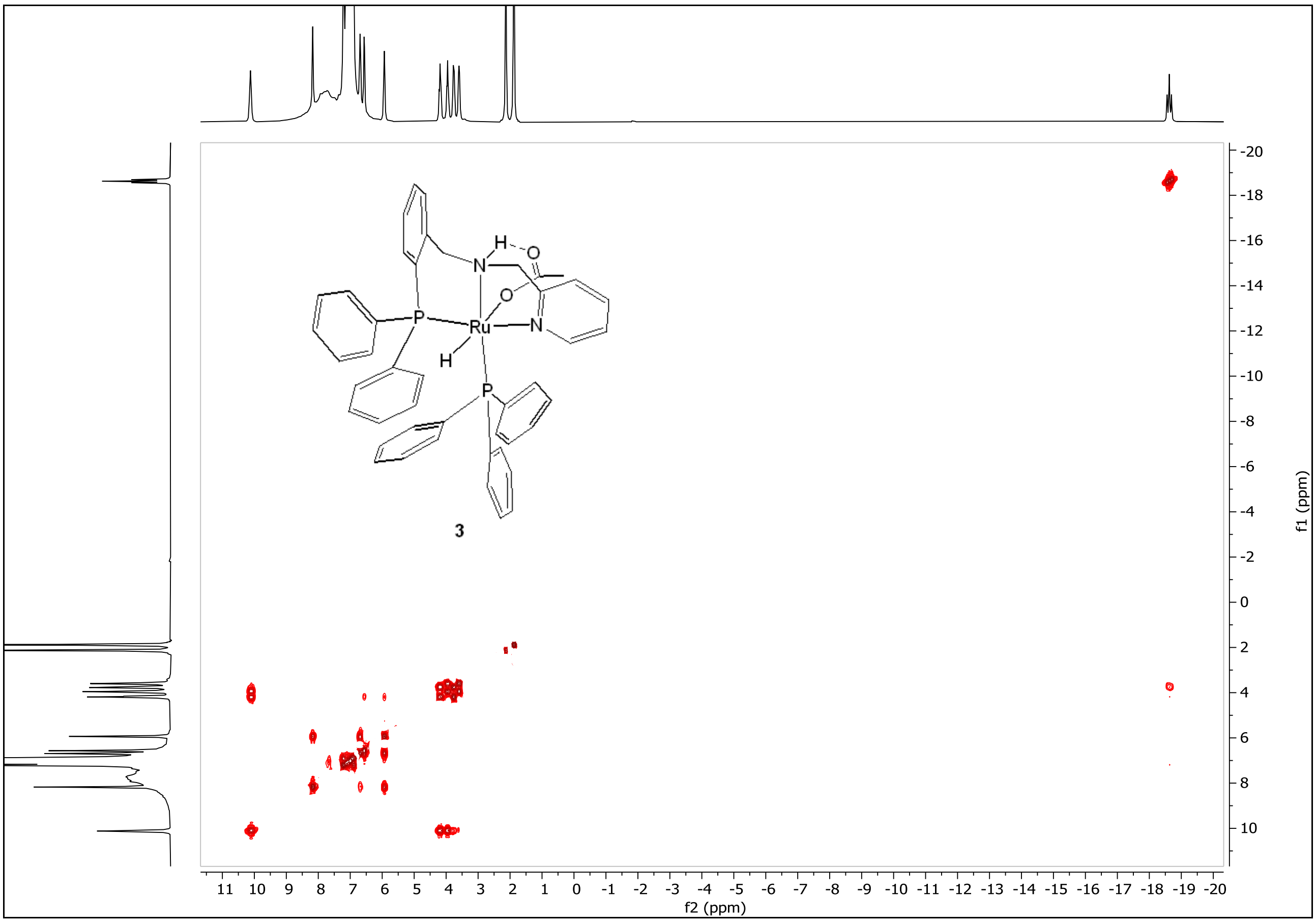

Figure S21. ^{1}H-^{1}H COSY 2D NMR spectrum of *trans*-[RuH(η^{1}-OAc)(PNN)(PPh_3)] (**3**) in toluene-d^8 at 25 °C.

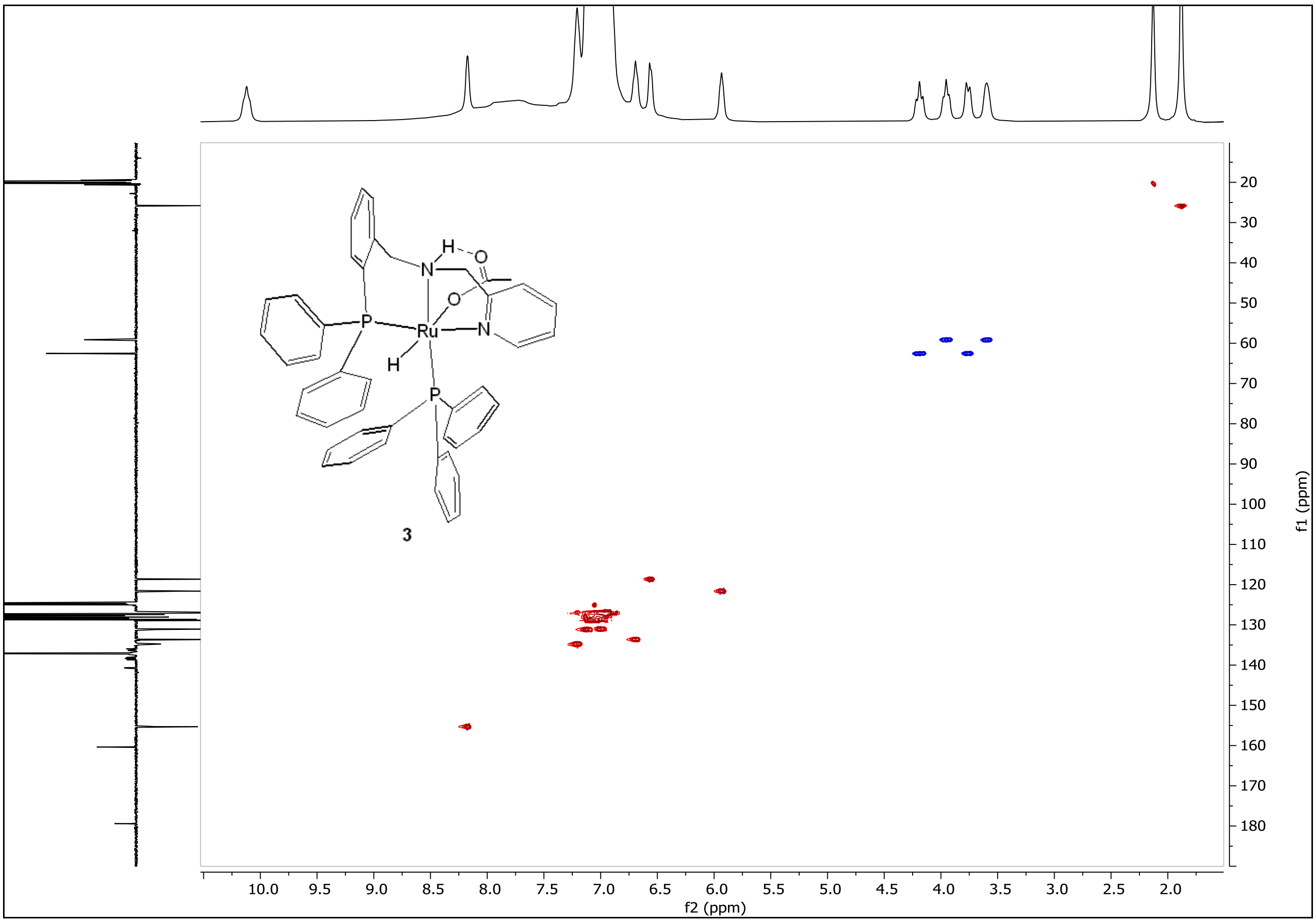

Figure S22. ^{1}H-^{13}C HSQC 2D NMR spectrum of *trans*-[RuH(η^1-OAc)(PNN)(PPh_3)] (**3**) in toluene-d^8 at 25 °C.

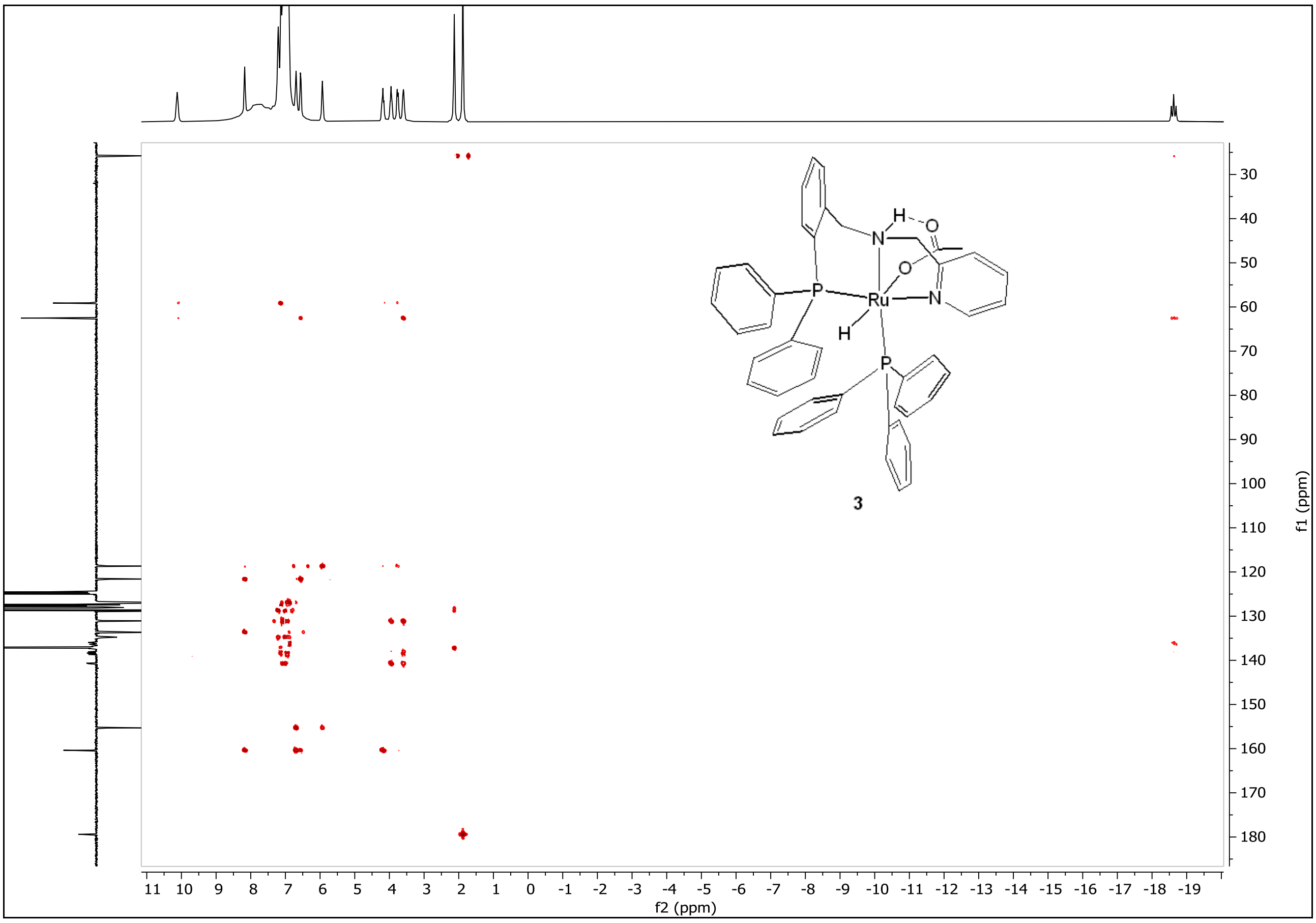

Figure S23. ^{1}H-^{13}C HMBC 2D NMR spectrum of *trans*-[RuH(η^1-OAc)(PNN)(PPh_3)] (**3**) in toluene-d^8 at 25 °C.

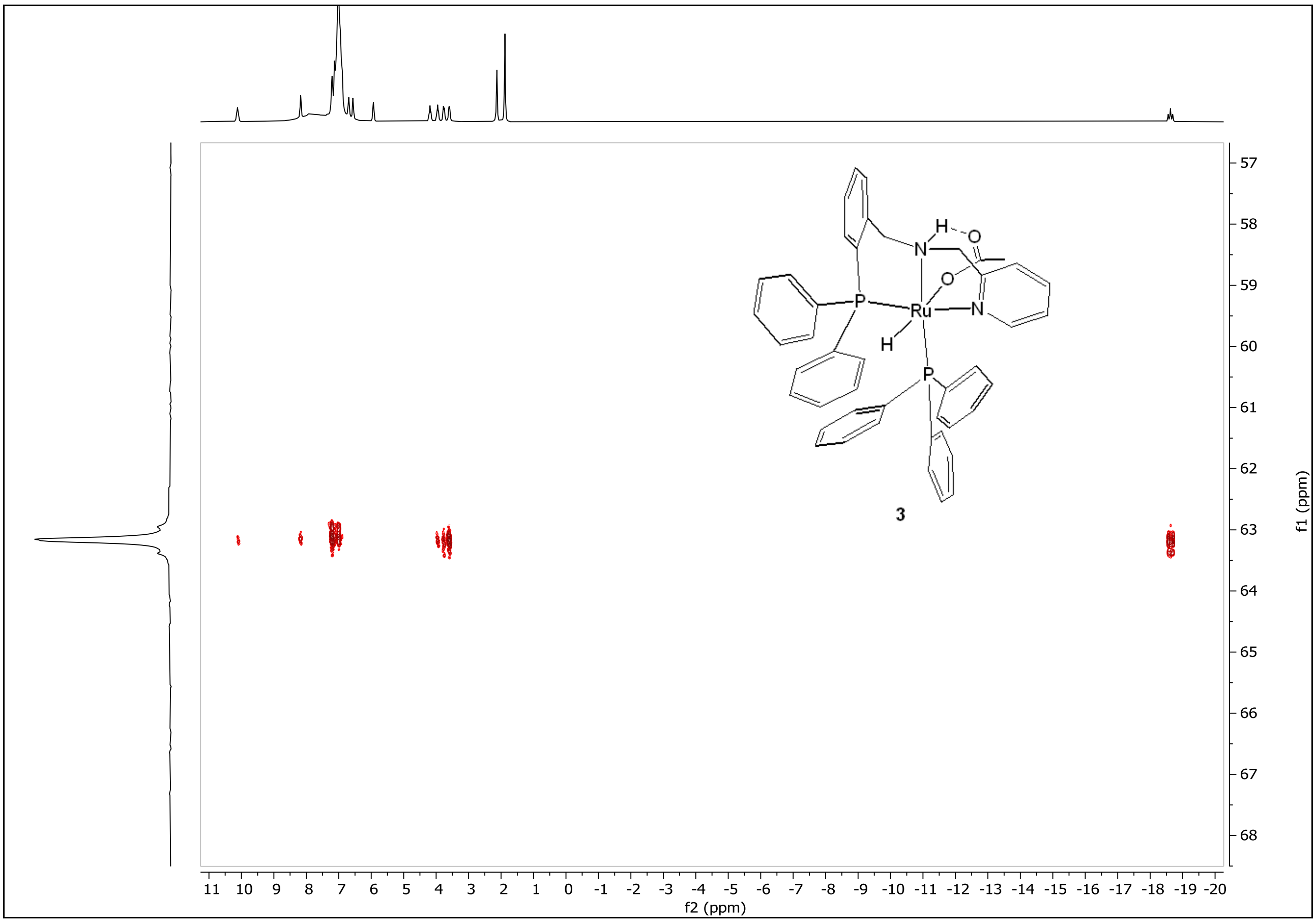

Figure S24. ^{1}H-^{31}P HMBC 2D NMR spectrum of *trans*-[RuH(η^1-OAc)(PNN)(PPh_3)] (**3**) in toluene-*d*^{8} at 25 °C.

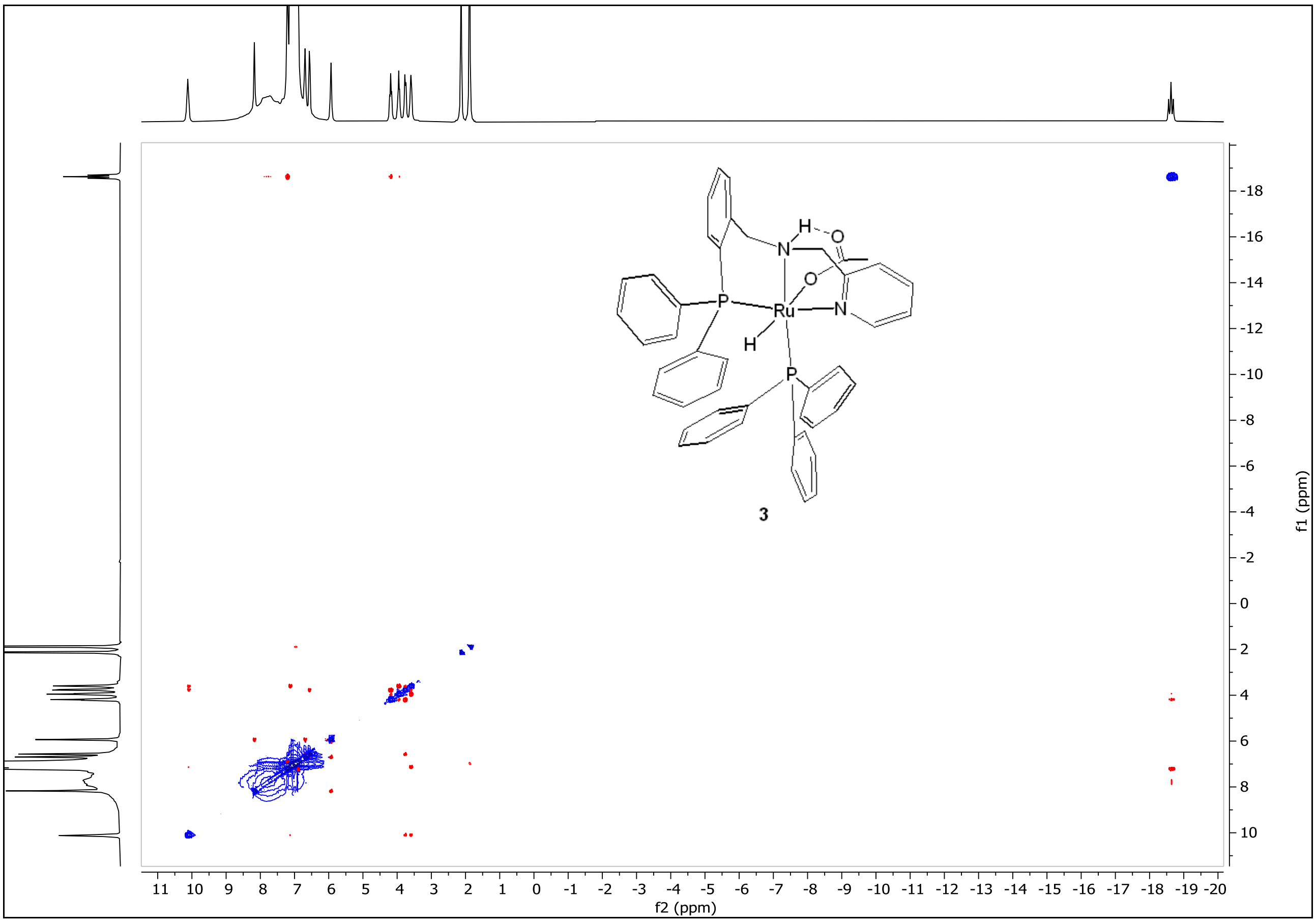

Figure S25. ^{1}H-^{1}H NOESY 2D NMR spectrum of *trans*-[RuH(η[1]-OAc)(PNN)(PPh_3)] (**3**) in toluene-*d*[8] at 25 °C.

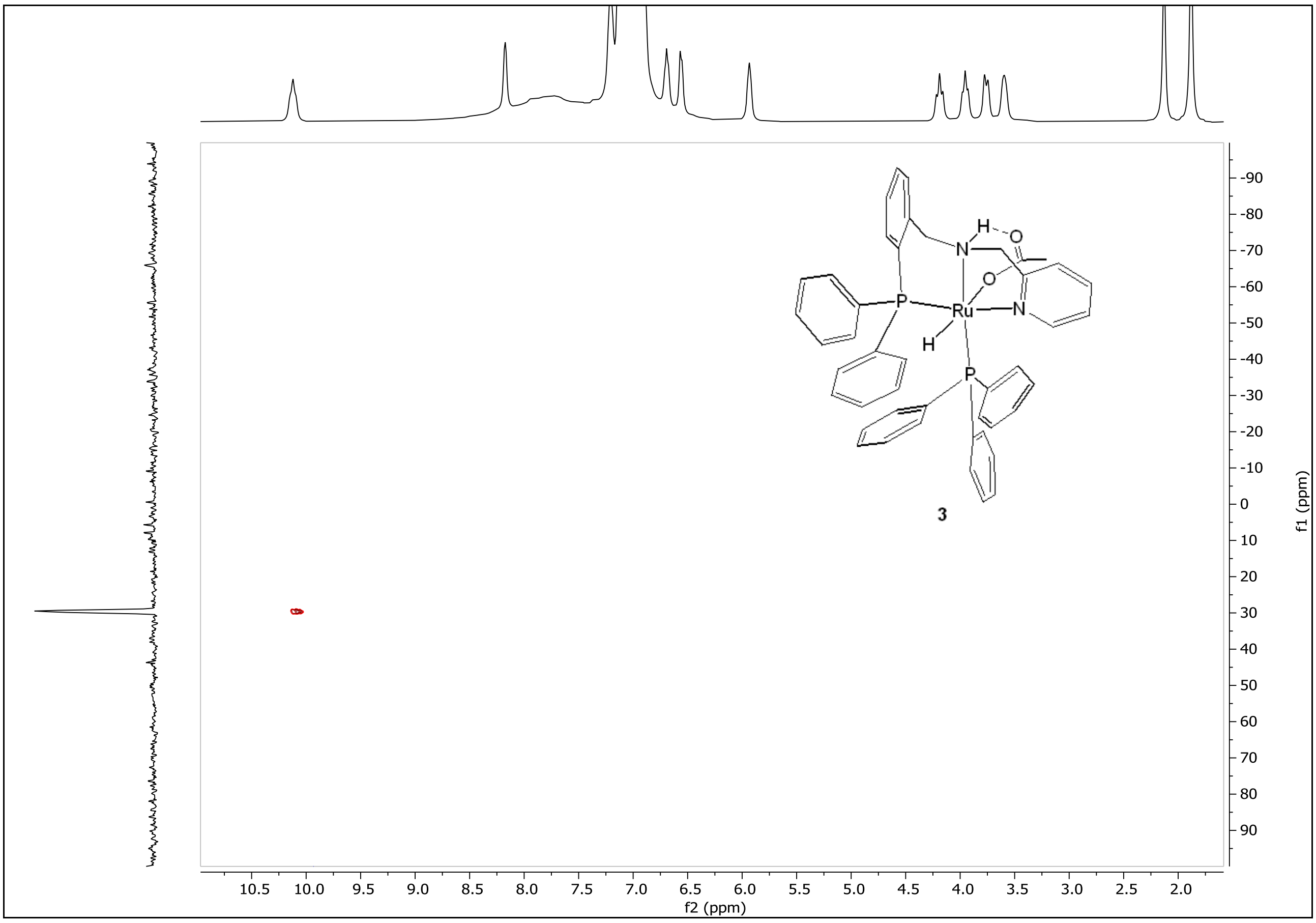

Figure S26. ^{1}H-^{15}N HSQC 2D NMR spectrum of *trans*-[RuH(η^1-OAc)(PNN)(PPh_3)] (**3**) in toluene-d^8 at 25 °C.

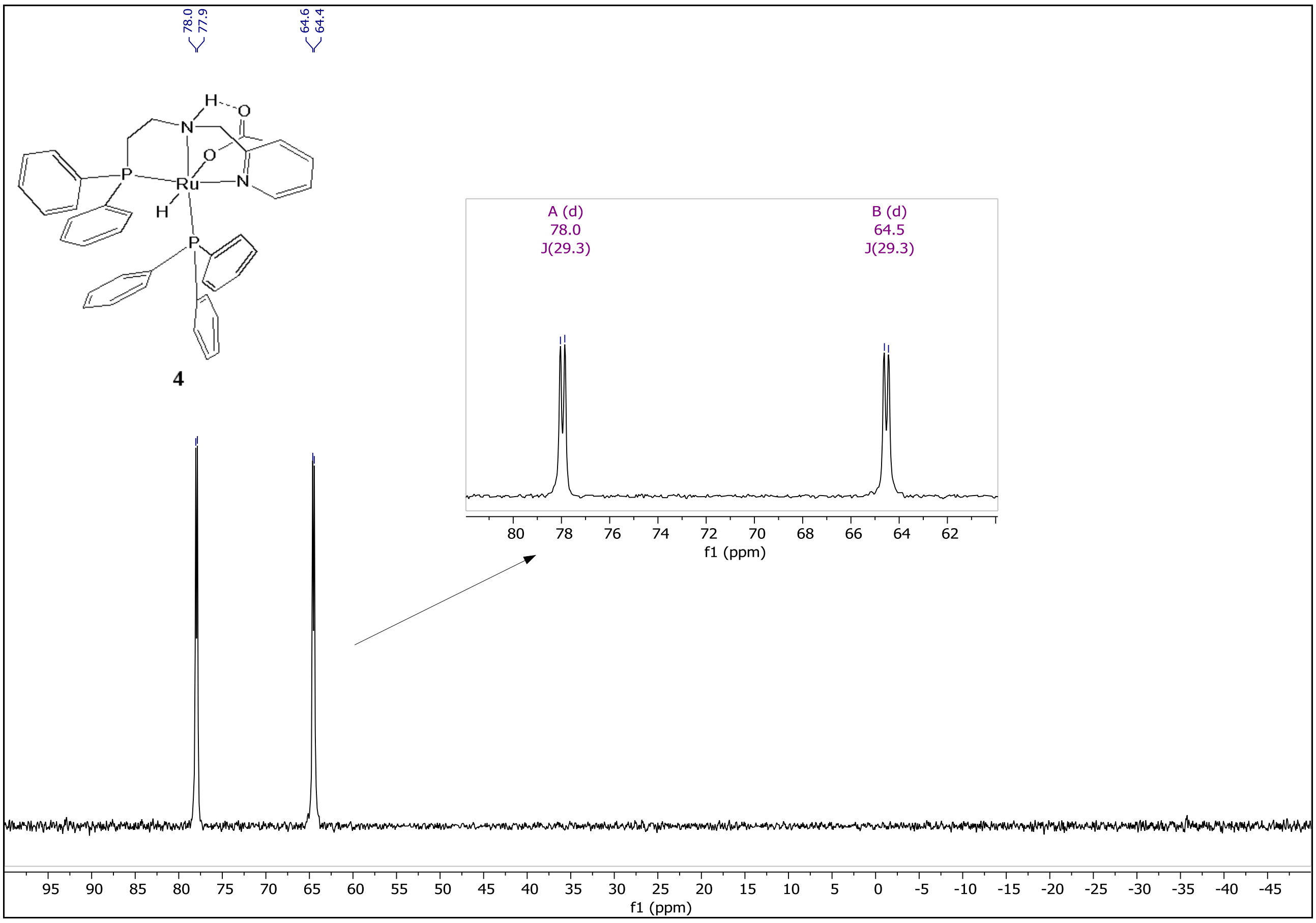

Figure S27. $^{31}P\{^{1}H\}$ NMR spectrum (162.0 MHz) of *trans*-[RuH(η^1-OAc)(PNN^G)(PPh_3)] (**4**) in toluene-d^8 at 25 °C.

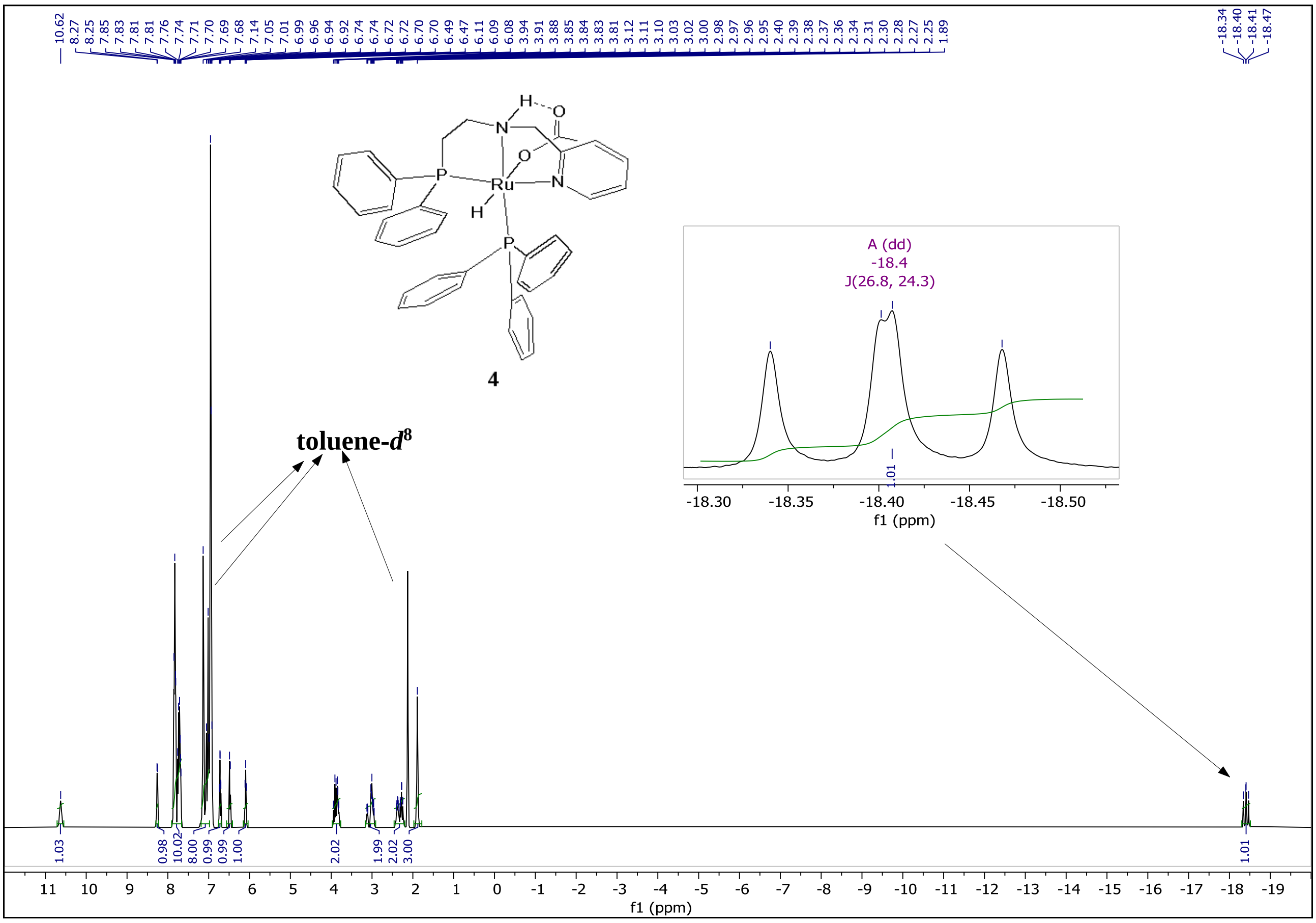

Figure S28. ^{1}H NMR spectrum (400.1 MHz) of *trans*-[RuH(η^1-OAc)(PNN^G)(PPh_3)] (**4**) in toluene-d^8 at 25 °C.

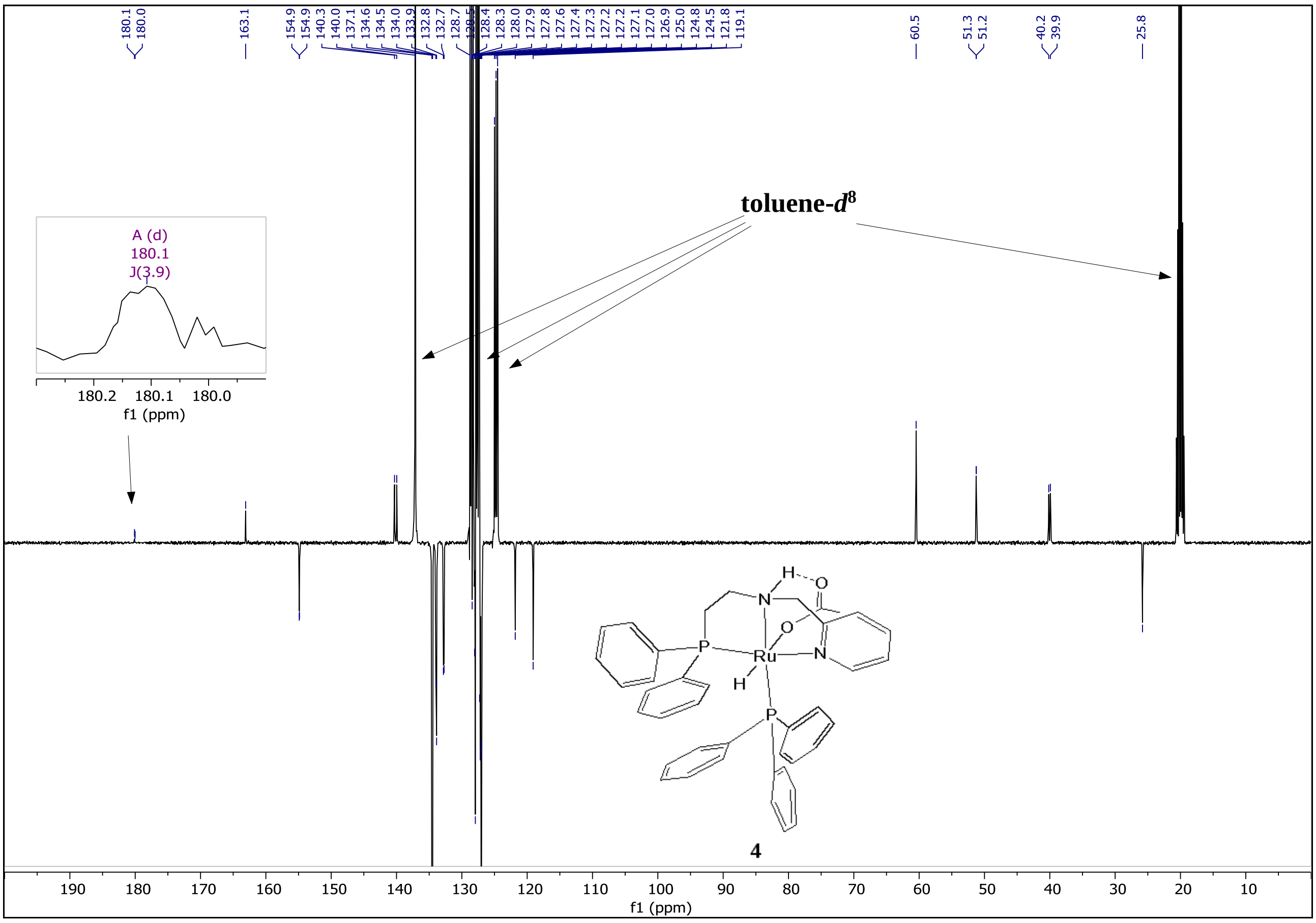

Figure S29. $^{13}C\{^{1}H\}$ DEPTQ NMR spectrum (100.6 MHz) of *trans*-[RuH(η^1-OAc)(PNN^G)(PPh_3)] (**4**) in toluene-d^8 at 25 °C.

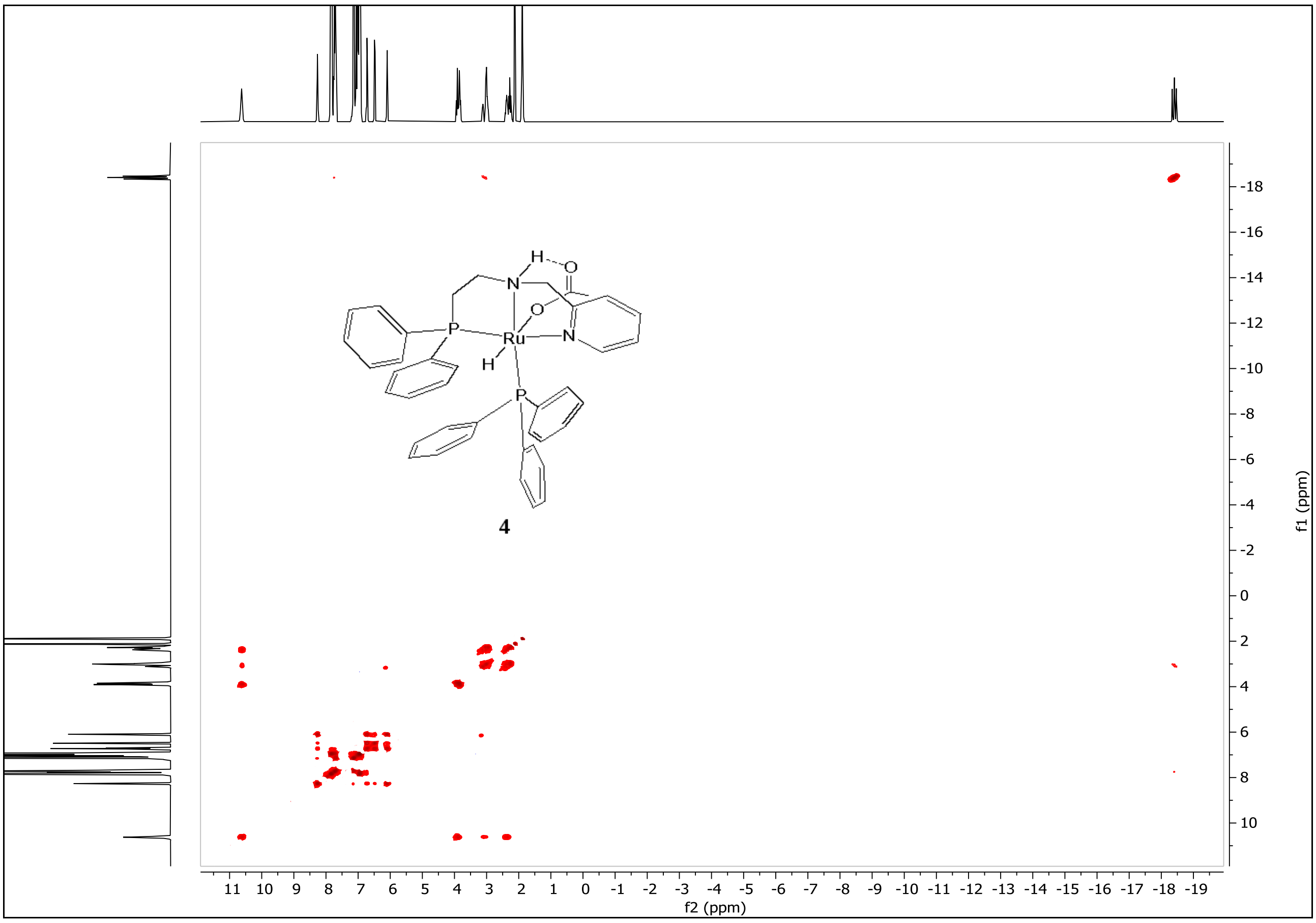

Figure S30. ^{1}H-^{1}H COSY 2D NMR spectrum of *trans*-[RuH(η^{1}-OAc)(PNN^{G})(PPh_3)] (**4**) in toluene-d^{8} at 25 °C.

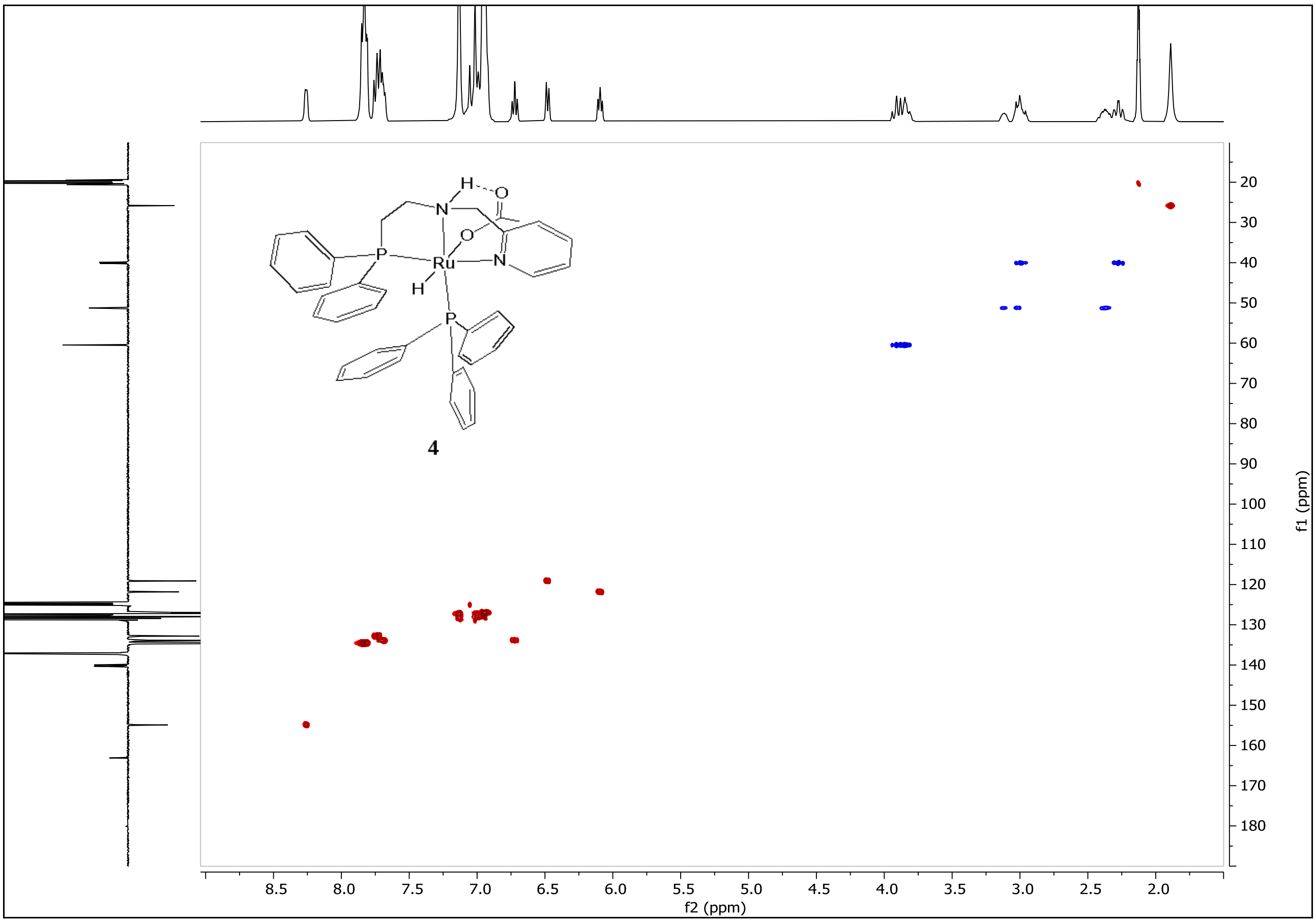

Figure S31. ^{1}H-^{13}C HSQC 2D NMR spectrum of *trans*-[RuH(η^{1}-OAc)(PNN^{G})(PPh_3)] (**4**) in toluene-d^{8} at 25 °C.

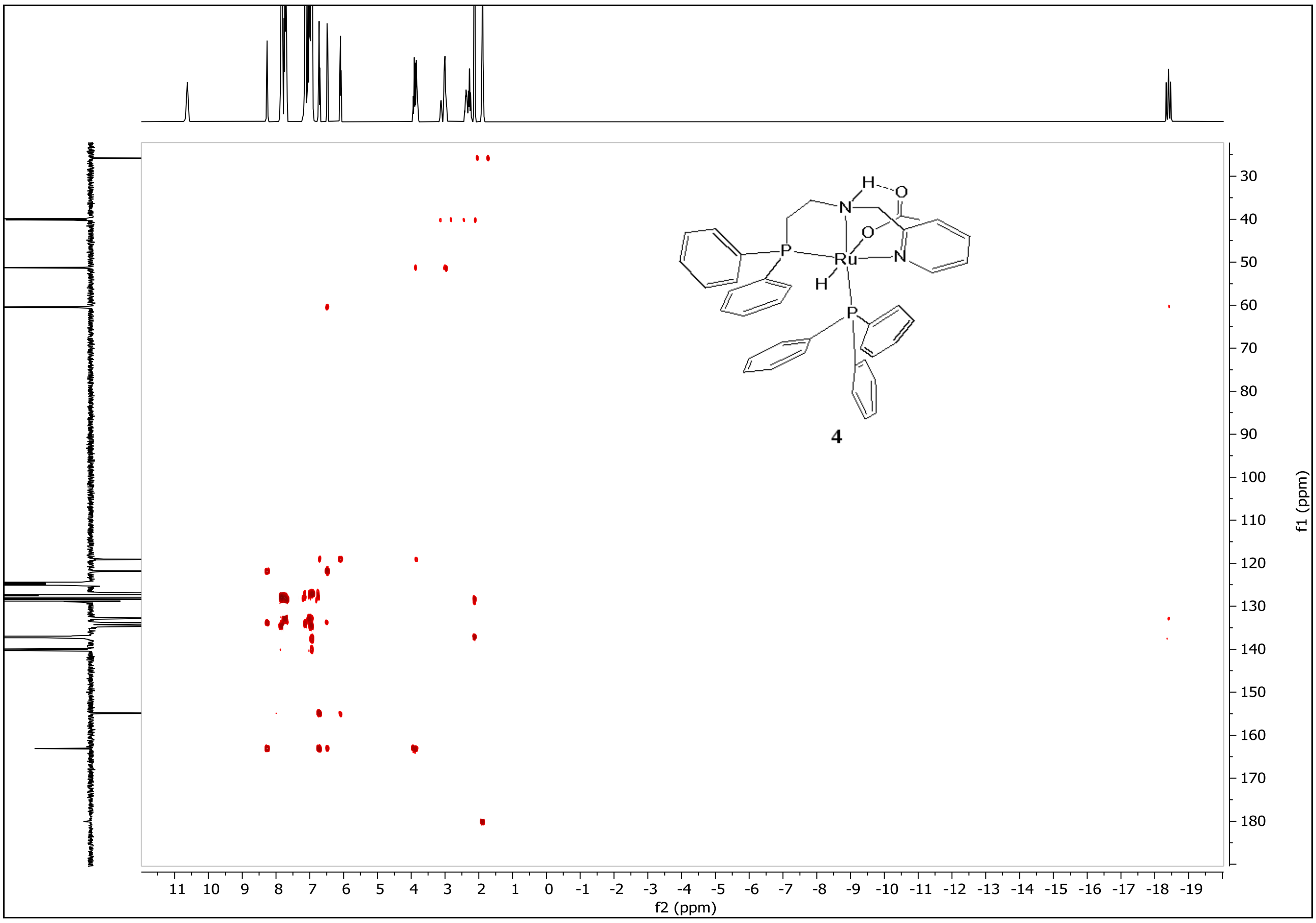

Figure S32. ^{1}H-^{13}C HMBC 2D NMR spectrum of *trans*-[RuH(η^{1}-OAc)(PNN^{G})(PPh_3)] (**4**) in toluene-d^{8} at 25 °C.

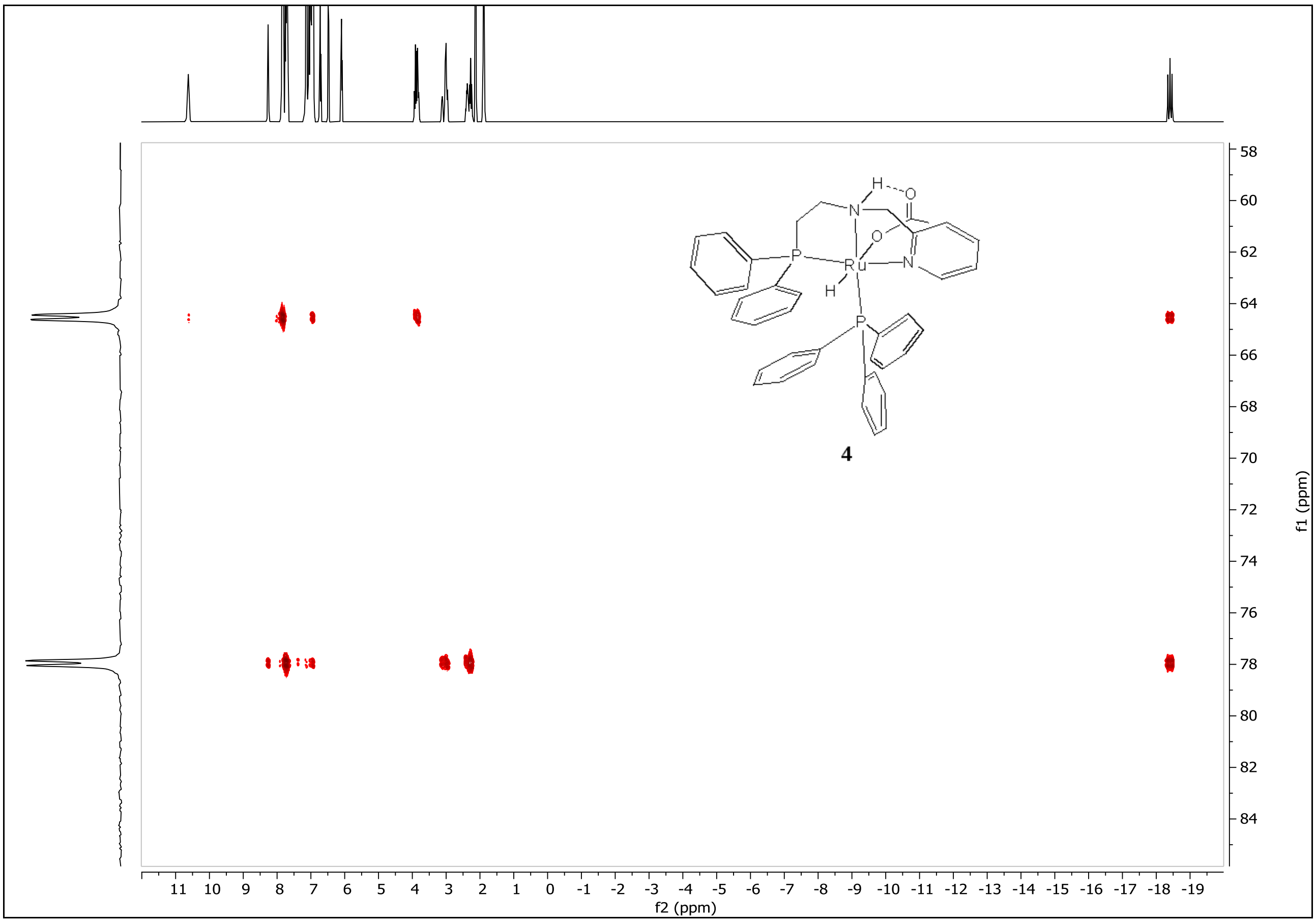

Figure S33. ^{1}H-^{31}P HMBC 2D NMR spectrum of *trans*-[RuH(η^1-OAc)(PNN^G)(PPh_3)] (**4**) in toluene-d^8 at 25 °C.

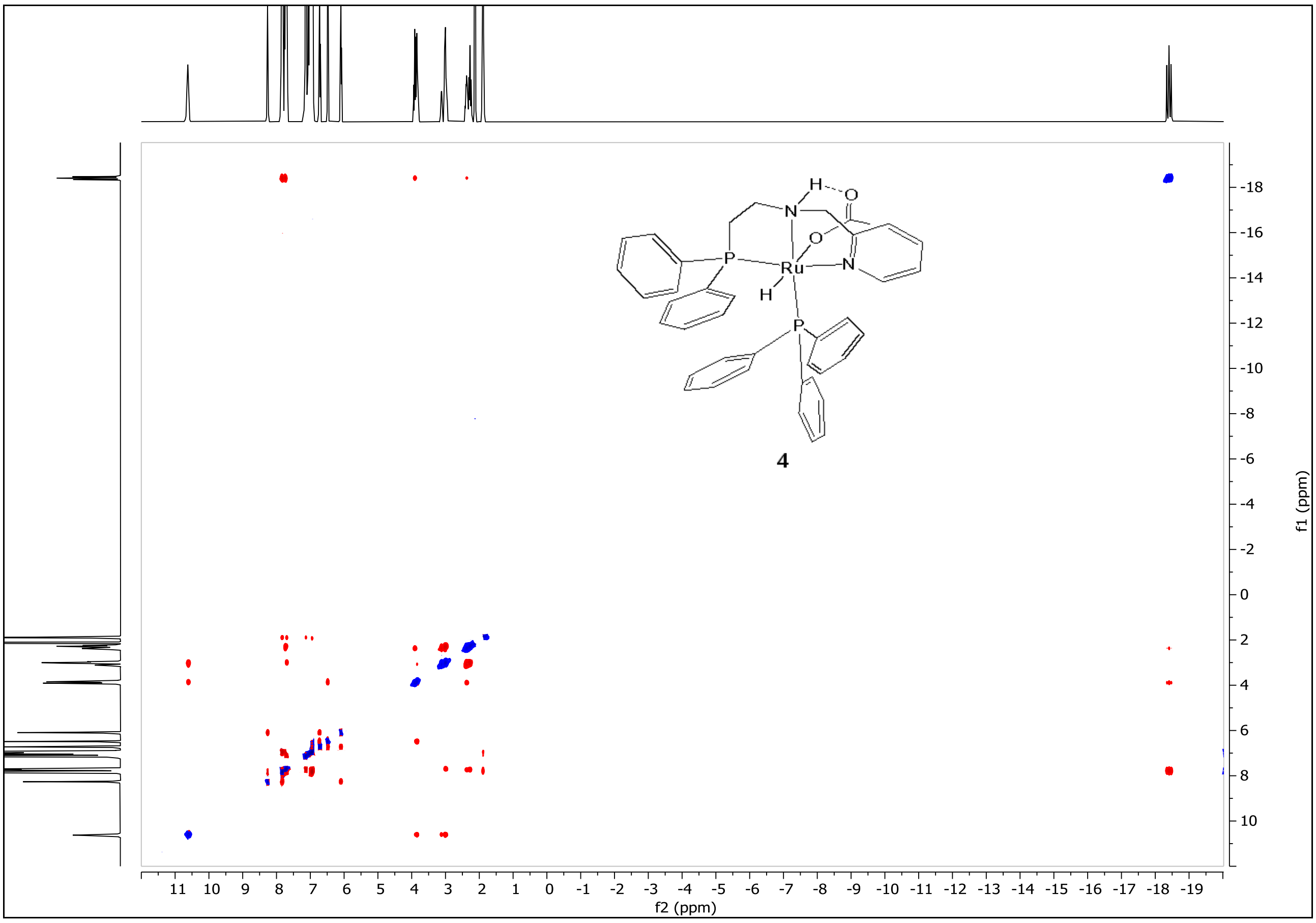

Figure S34. ^{1}H-^{1}H NOESY 2D NMR spectrum of *trans*-[RuH(η^1-OAc)(PNN^G)(PPh_3)] (**4**) in toluene-d^8 at 25 °C.

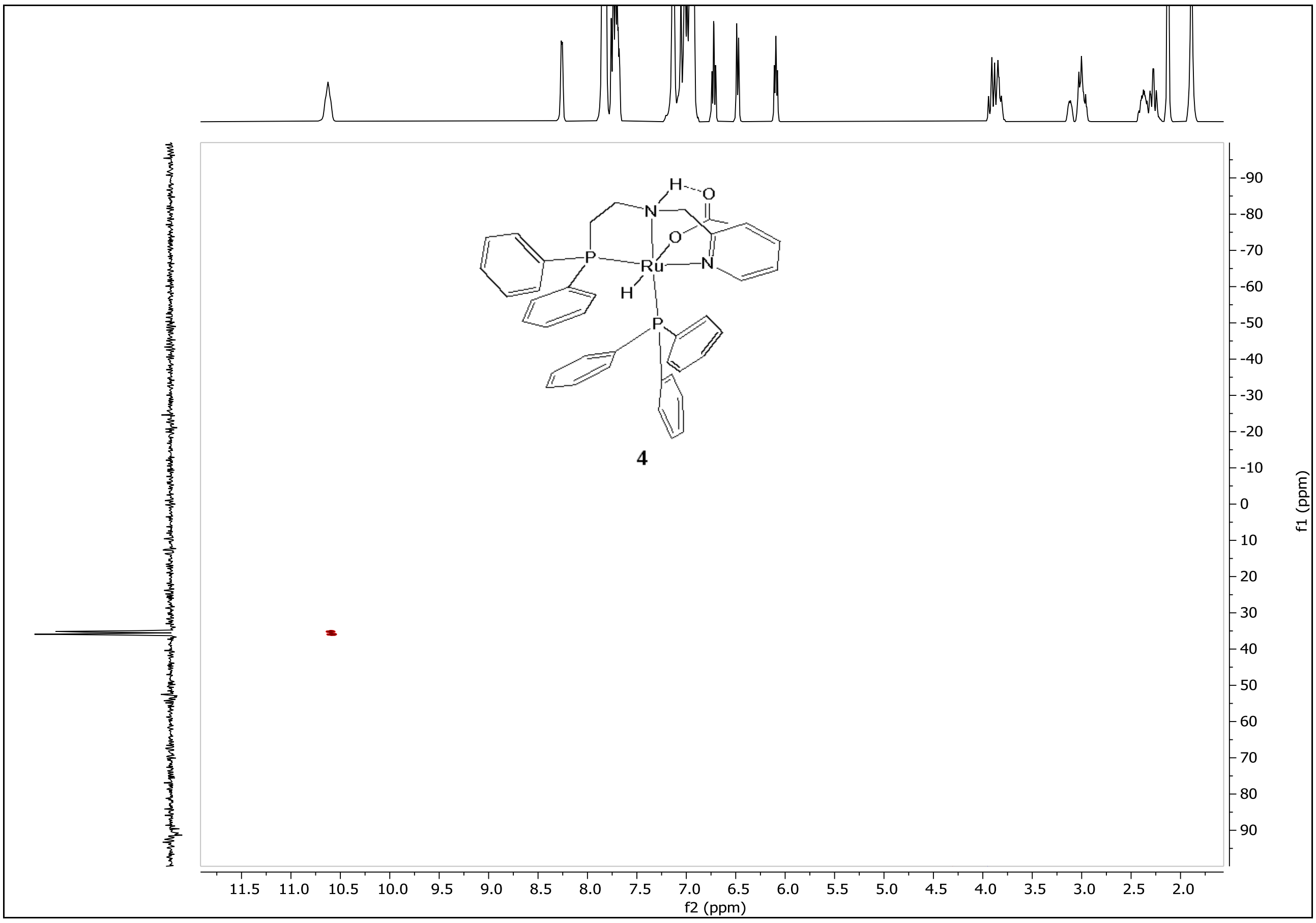

Figure S35. ^{1}H-^{15}N HSQC 2D NMR spectrum of *trans*-[RuH(η^1-OAc)(PNN^G)(PPh_3)] (**4**) in toluene-*d*^{8} at 25 °C.

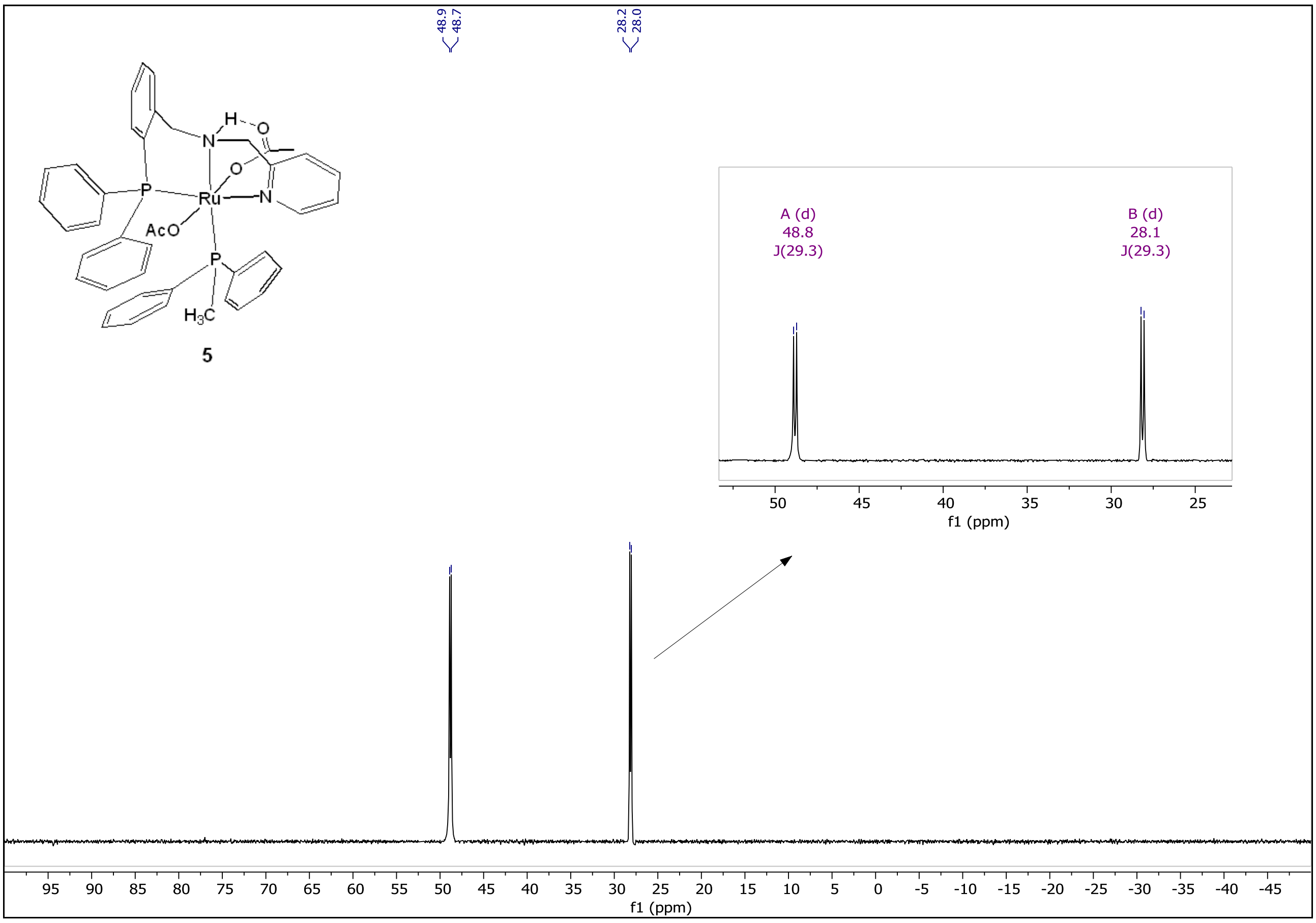

Figure S36. $^{31}P\{^{1}H\}$ NMR spectrum (162.0 MHz) of *trans*-[Ru(η^1-OAc)$_2$(PNN')(PPh$_2$Me)] (**5**) in toluene-d^8 at 25 °C.

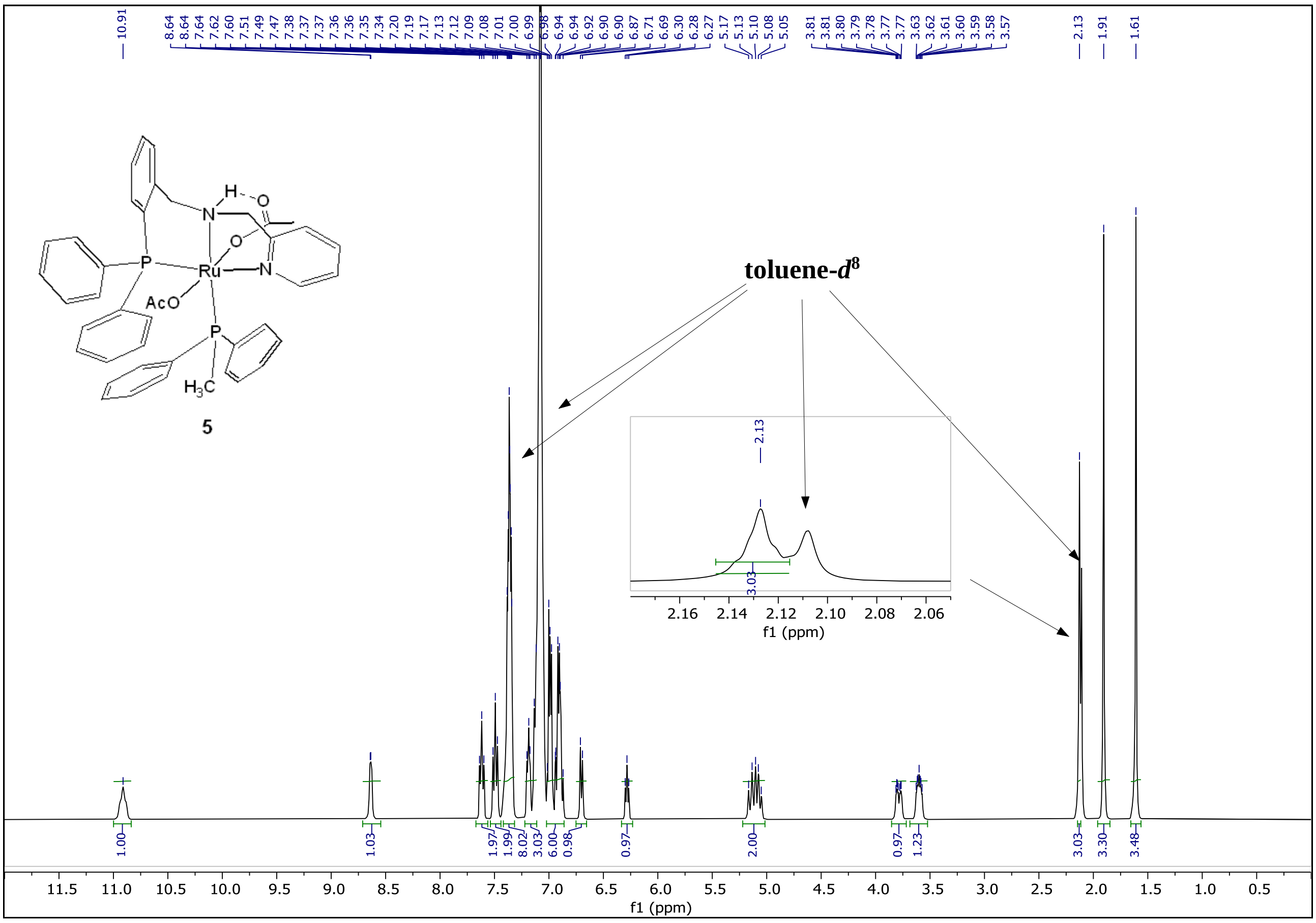

Figure S37. ^{1}H NMR spectrum (400.1 MHz) of *trans*-[Ru(η^1-OAc)$_2$(PNN')(PPh$_2$Me)] (**5**) in toluene-d^8 at 25 °C.

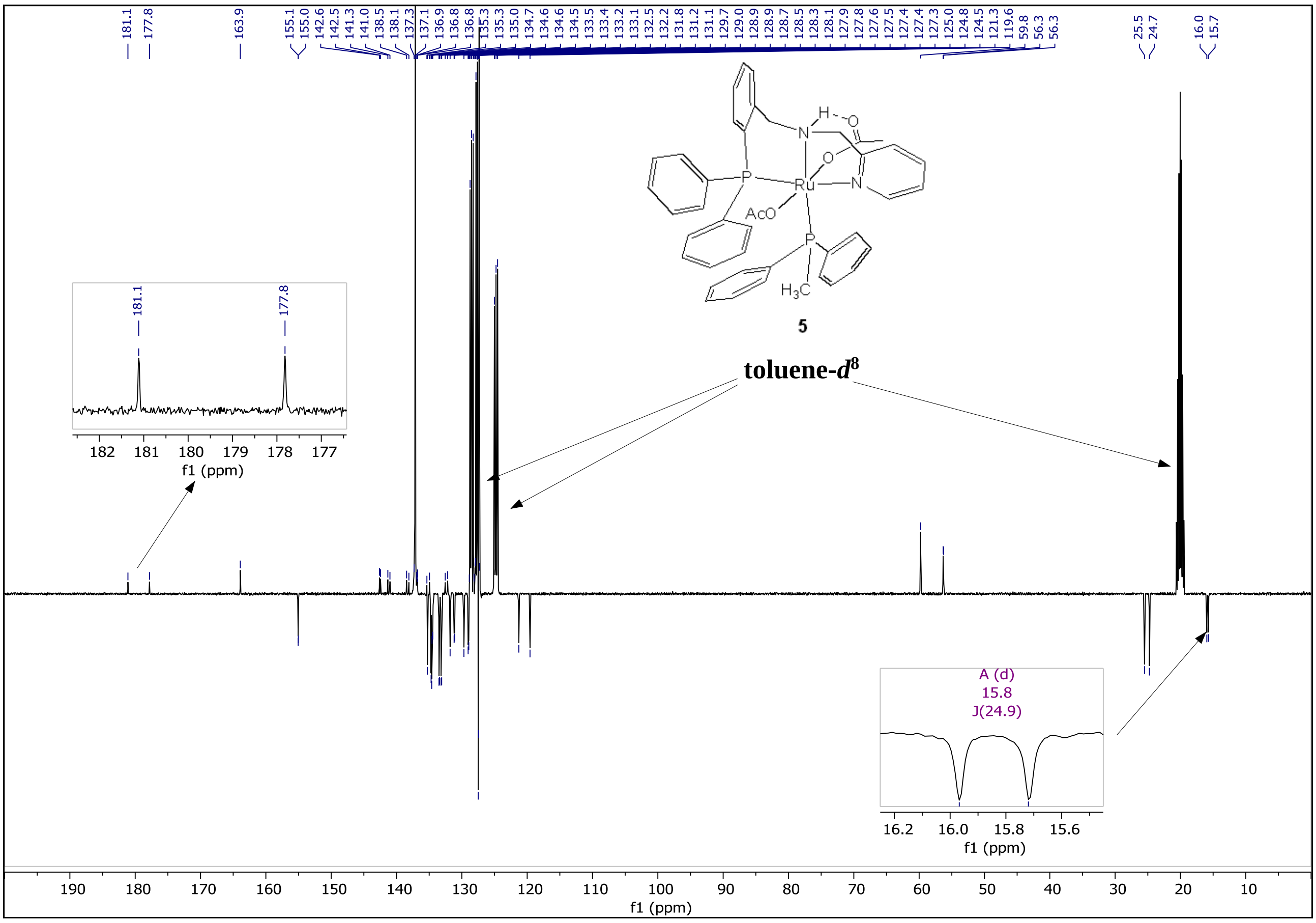

Figure S38. $^{13}C\{^{1}H\}$ DEPTQ NMR spectrum (100.6 MHz) of *trans*-[Ru(η[1]-OAc)$_2$(PNN')(PPh$_2$Me)] (**5**) in toluene-*d*[8] at 25 °C.

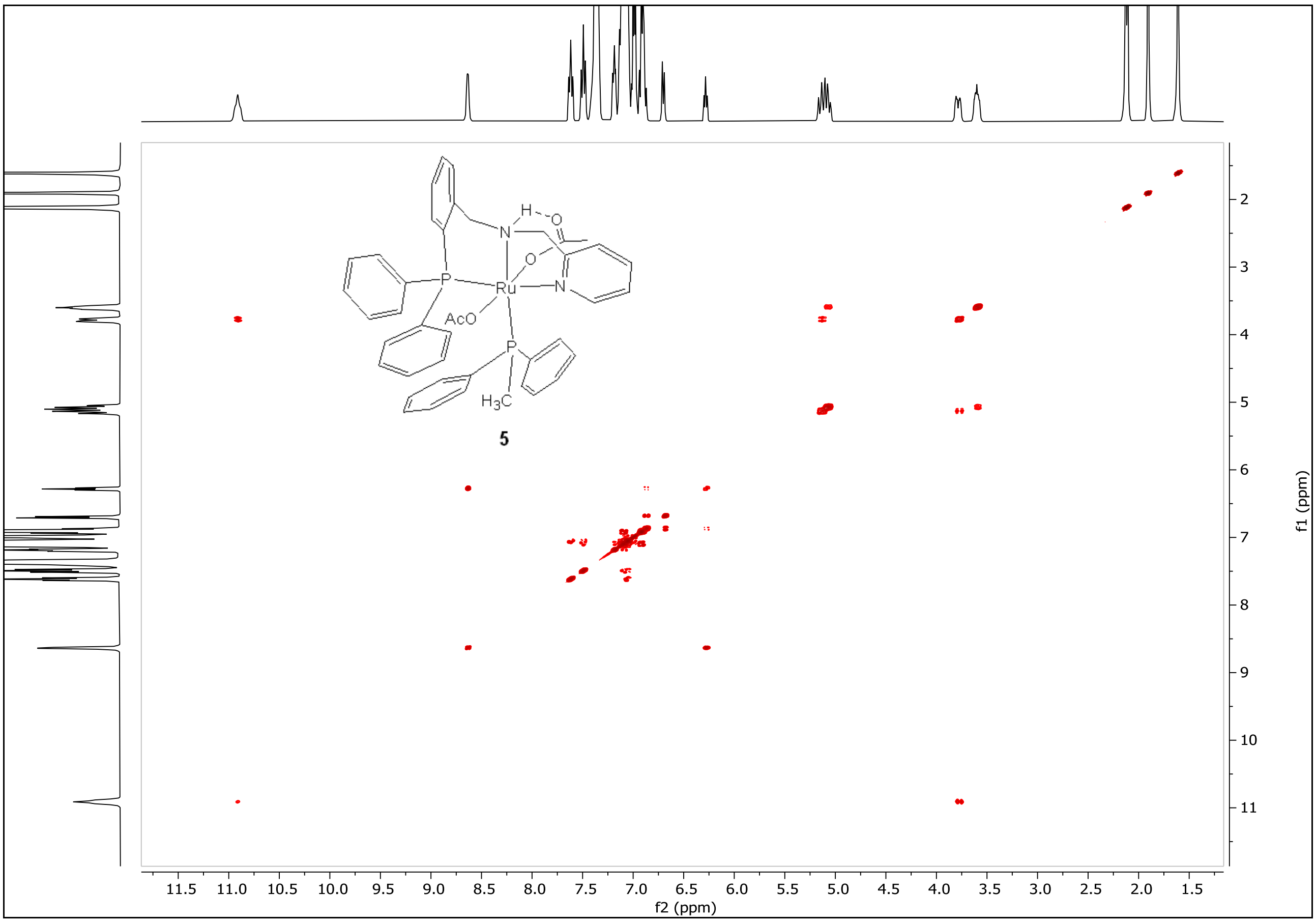

Figure S39. ^{1}H-^{1}H COSY 2D NMR spectrum of *trans*-[Ru(η^1-OAc)$_2$(PNN')(PPh$_2$Me)] (**5**) in toluene-*d*[8] at 25 °C.

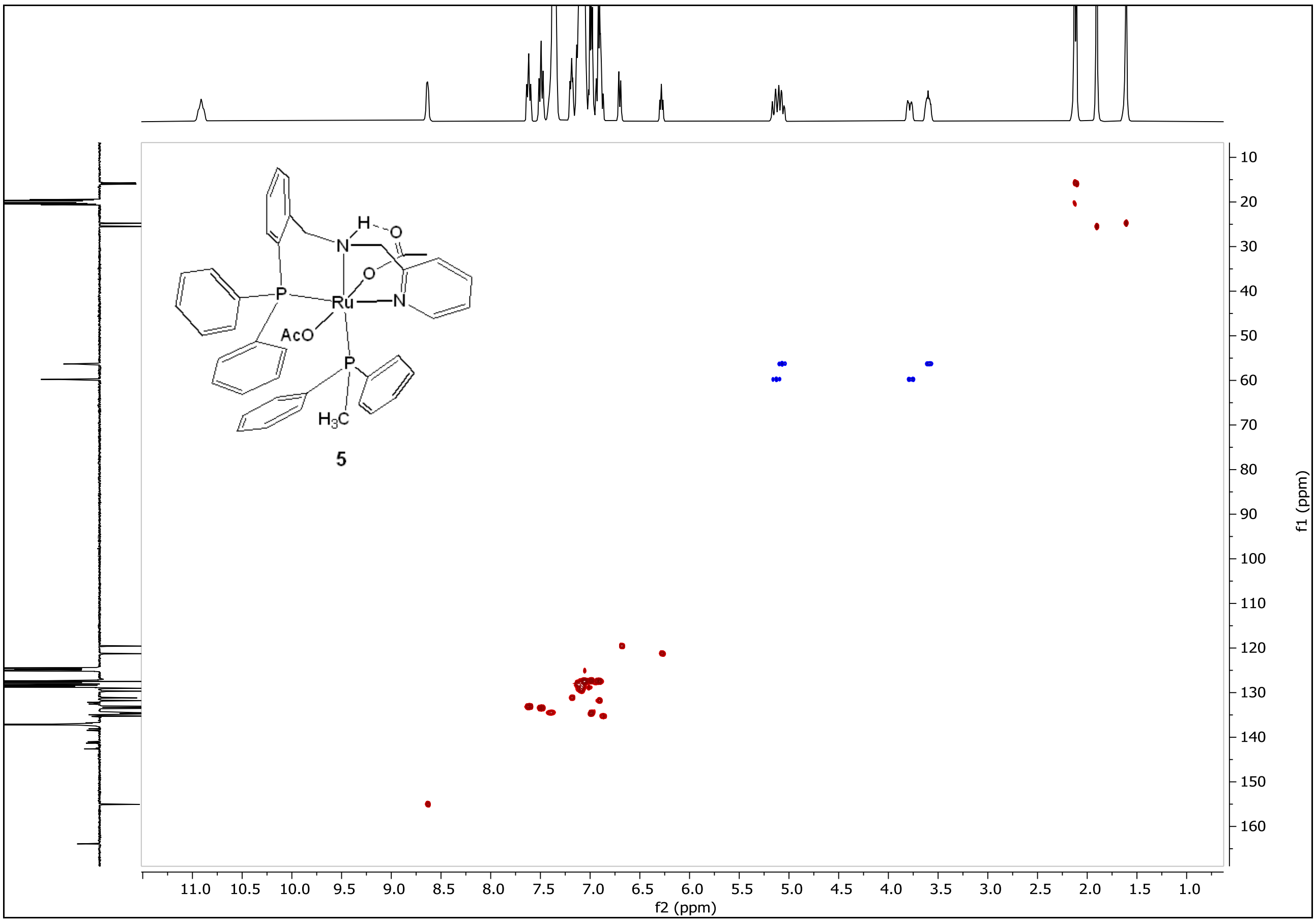

Figure S40. ^{1}H-^{13}C HSQC 2D NMR spectrum of *trans*-[Ru(η^1-OAc)$_2$(PNN')(PPh$_2$Me)] (**5**) in toluene-d^8 at 25 °C.

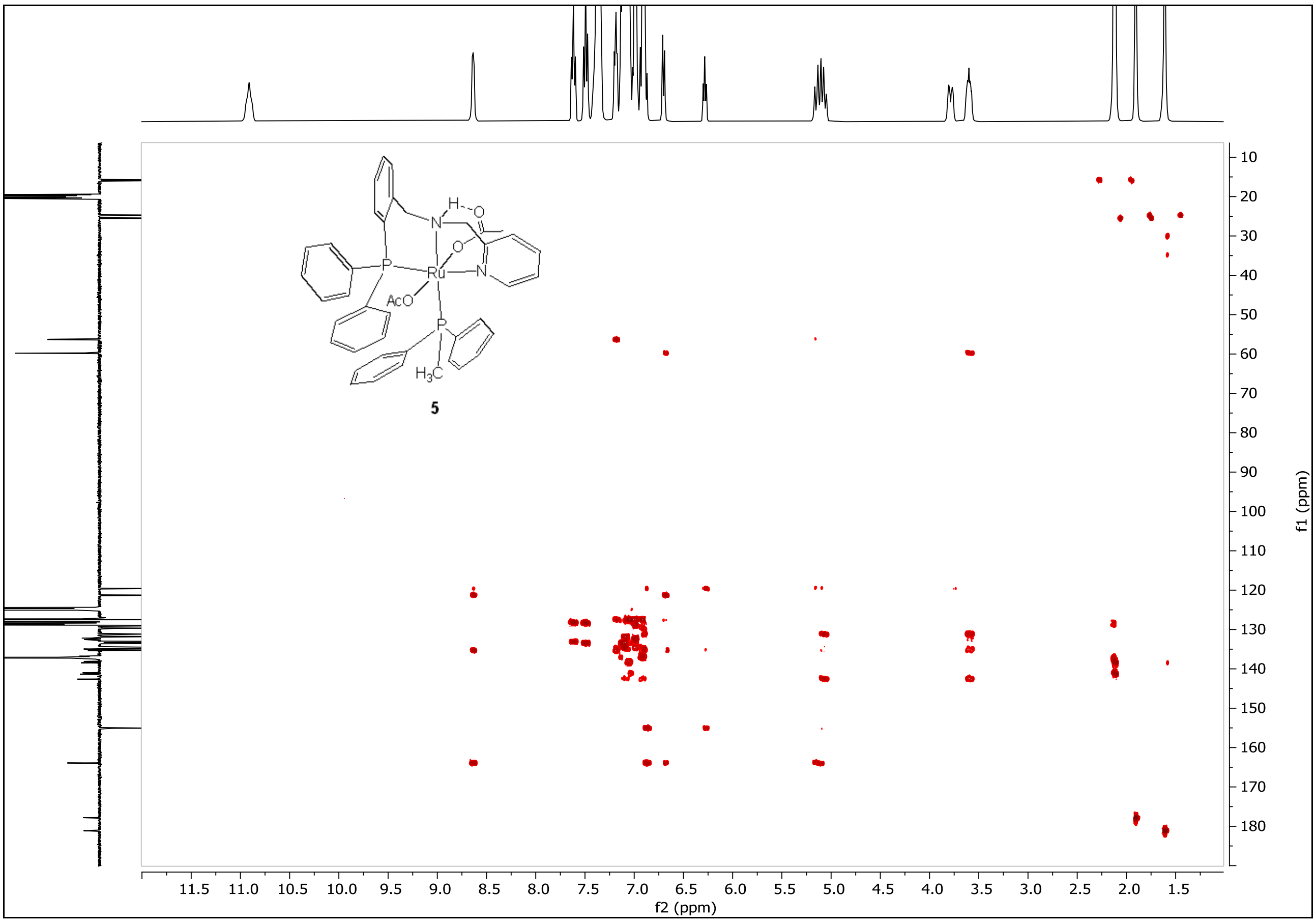

Figure S41. ^{1}H-^{13}C HMBC 2D NMR spectrum of *trans*-[Ru(η[1]-OAc)$_2$(PNN')(PPh$_2$Me)] (**5**) in toluene-d^8 at 25 °C.

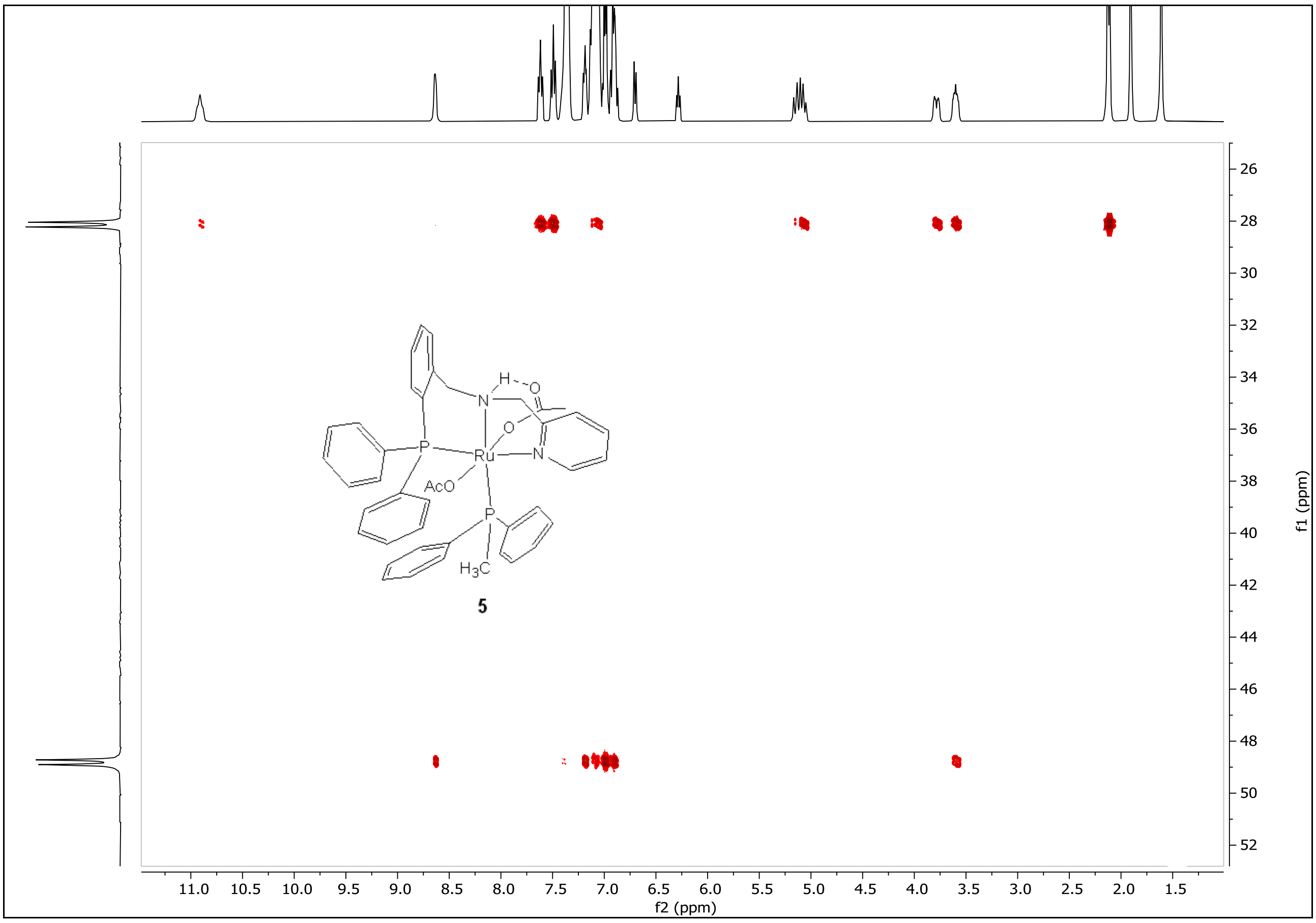

Figure S42. ^{1}H-^{31}P HMBC 2D NMR spectrum of *trans*-[Ru(η^{1}-OAc)$_2$(PNN')(PPh$_2$Me)] (**5**) in toluene-d^8 at 25 °C.

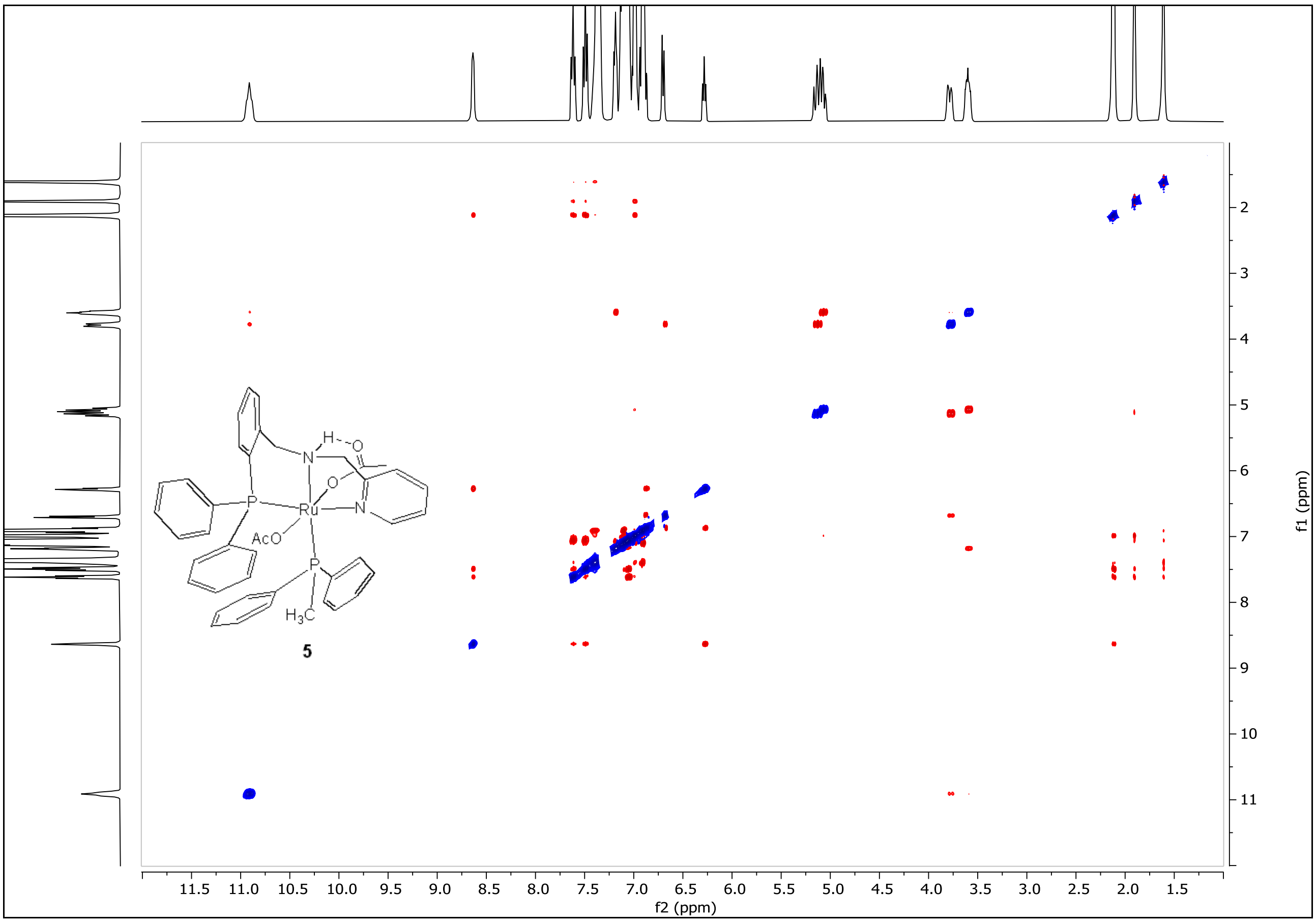

Figure S43. ^1H-^1H NOESY 2D NMR spectrum of *trans*-[Ru(η^1-OAc)$_2$(PNN')(PPh$_2$Me)] (**5**) in toluene-d^8 at 25 °C.

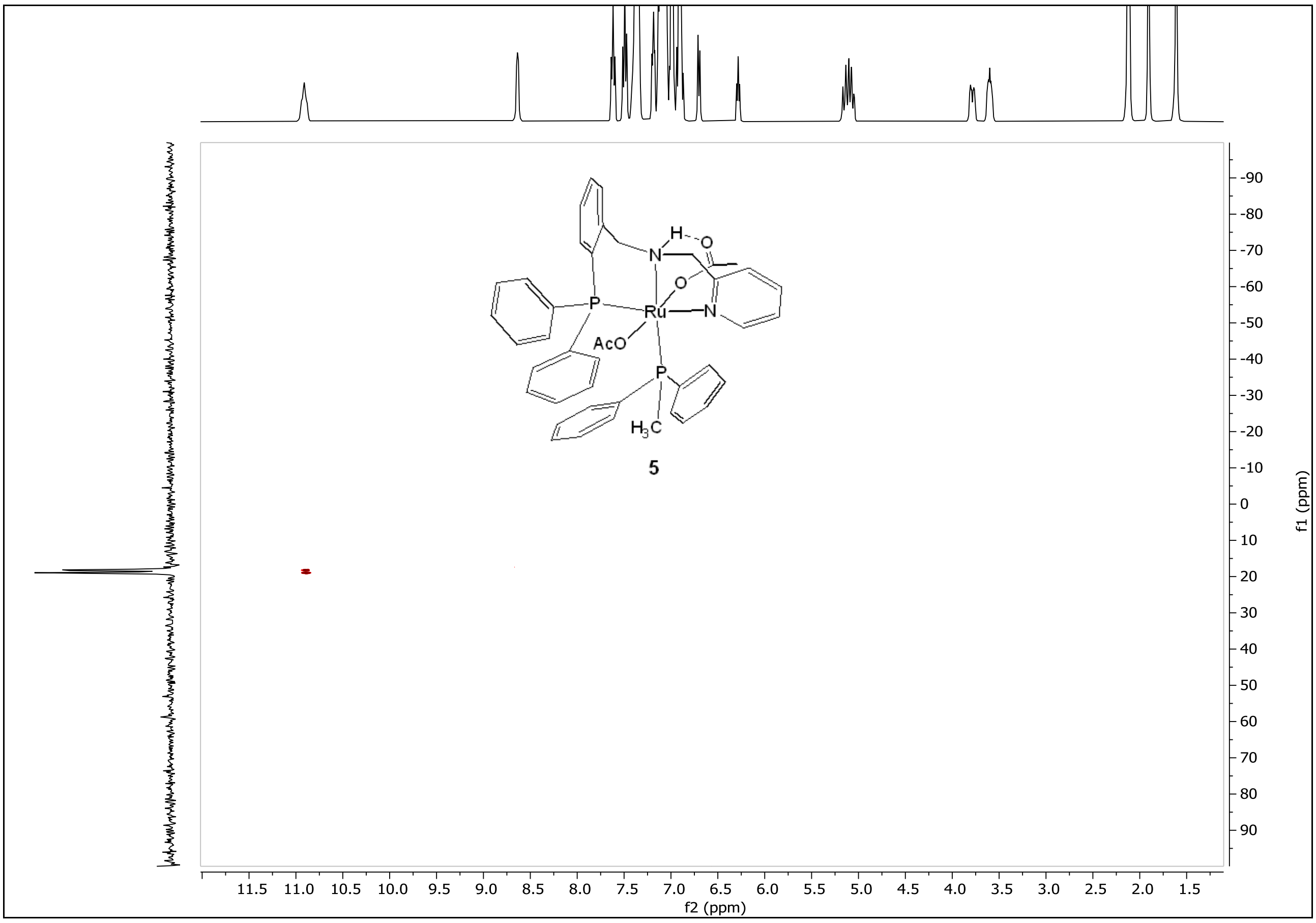

Figure S44. ^{1}H-^{15}N HSQC 2D NMR spectrum of *trans*-[Ru(η^1-OAc)$_2$(PNN')(PPh$_2$Me)] (**5**) in toluene-d^8 at 25 °C.

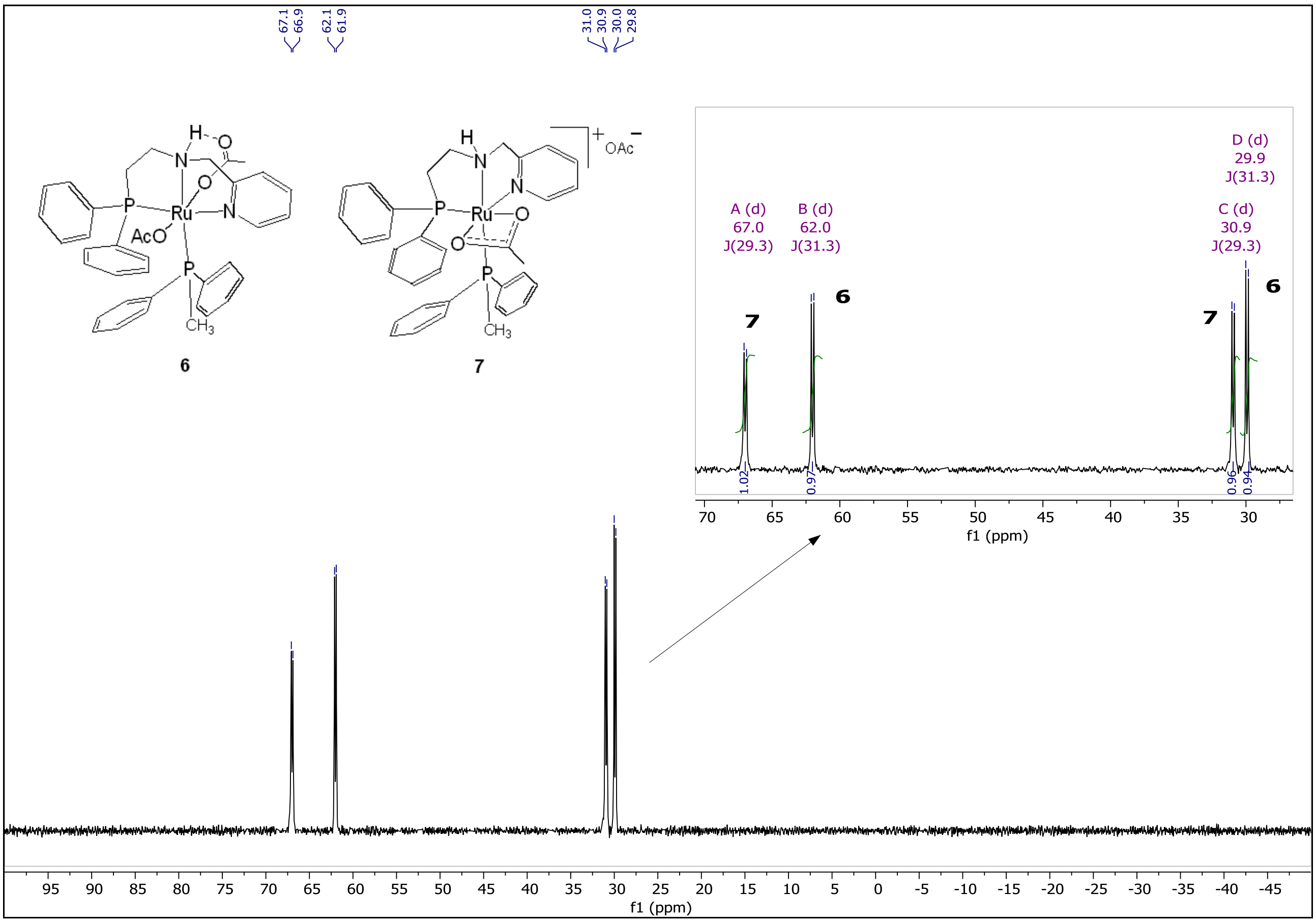

Figure S45. $^{31}P\{^{1}H\}$ NMR spectrum (162.0 MHz) of the 1:1 mixture of *trans*-[Ru(η^1-OAc)$_2$(PNN^G)(PPh$_2$Me)] (**6**) and [Ru(η^2-OAc)(PNN^G)(PPh$_2$Me)]OAc (**7**) in toluene-d^8 at 25 °C.

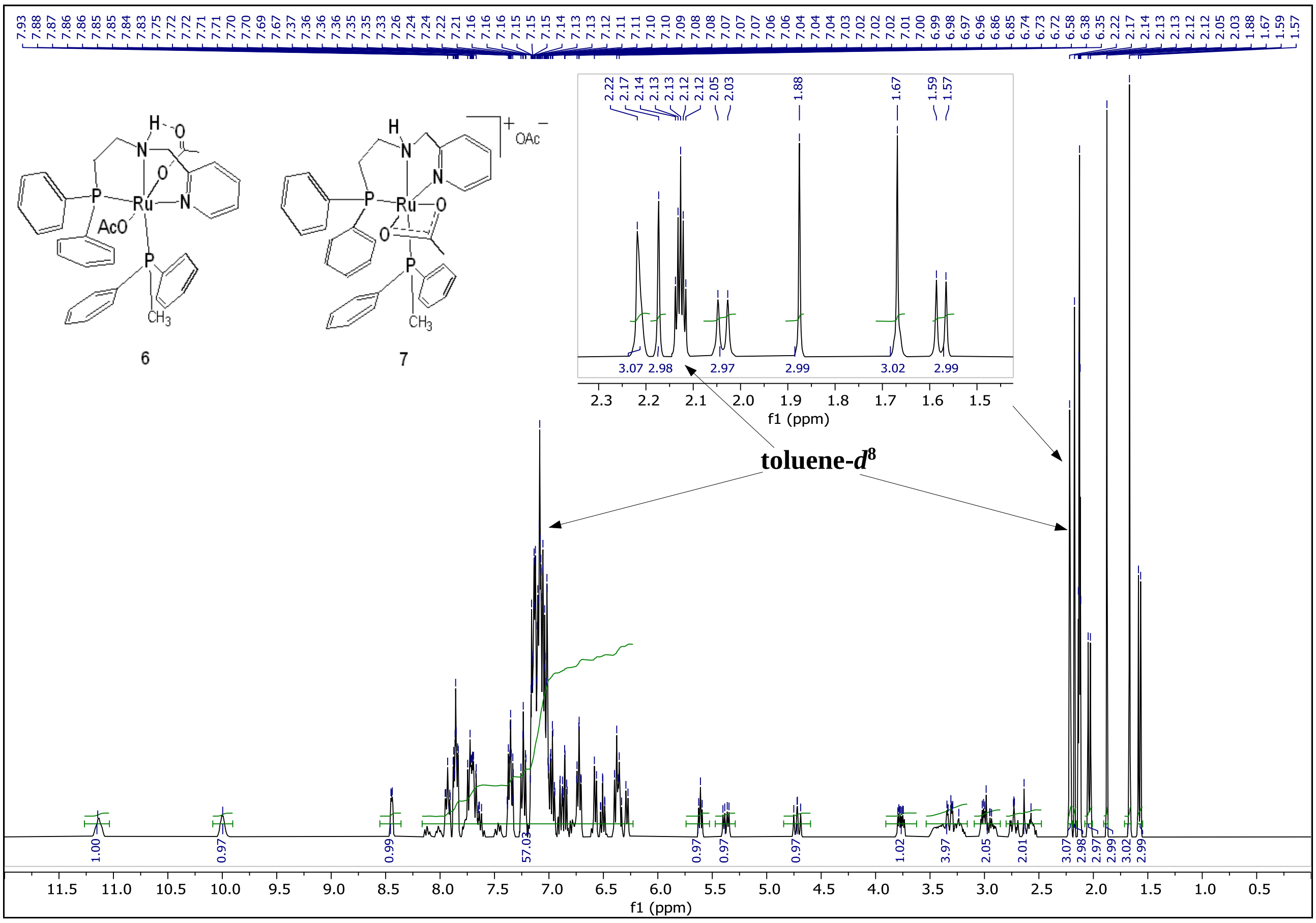

Figure S46. ^{1}H NMR spectrum (400.1 MHz) of the 1:1 mixture of *trans*-[Ru(η^1-OAc)$_2$(PNN^G)(PPh$_2$Me)] (**6**) and [Ru(η^2-OAc)(PNN^G)(PPh$_2$Me)]OAc (**7**) in toluene-d^8 at 25 °C.

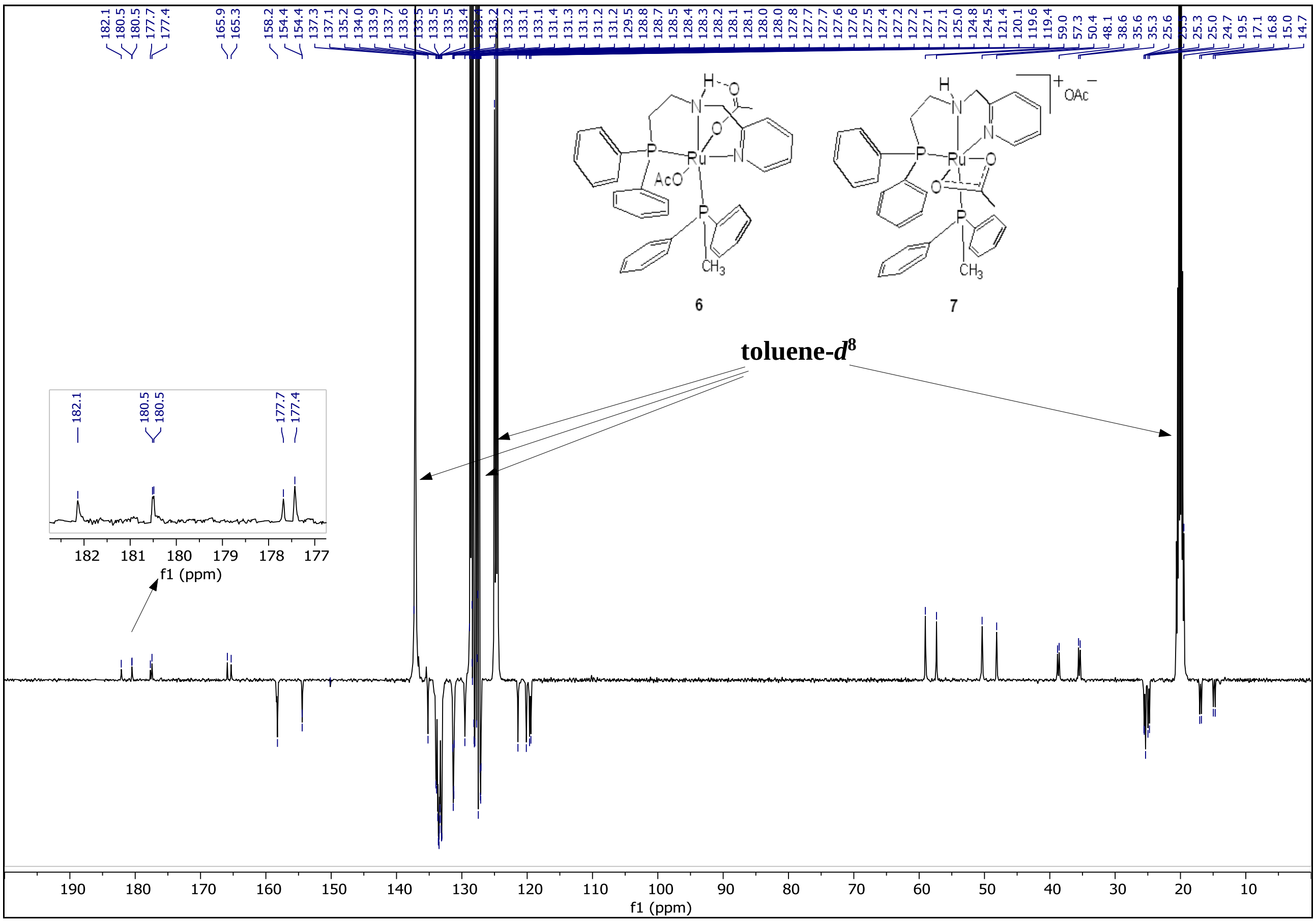

Figure S47. $^{13}C\{^1H\}$ NMR spectrum (100.6 MHz) of the 1:1 mixture of *trans*-[Ru(η^1-OAc)$_2$(PNN^G)(PPh$_2$Me)] (**6**) and [Ru(η^2-OAc)(PNN^G)(PPh$_2$Me)]OAc (**7**) in toluene-d^8 at 25 °C.

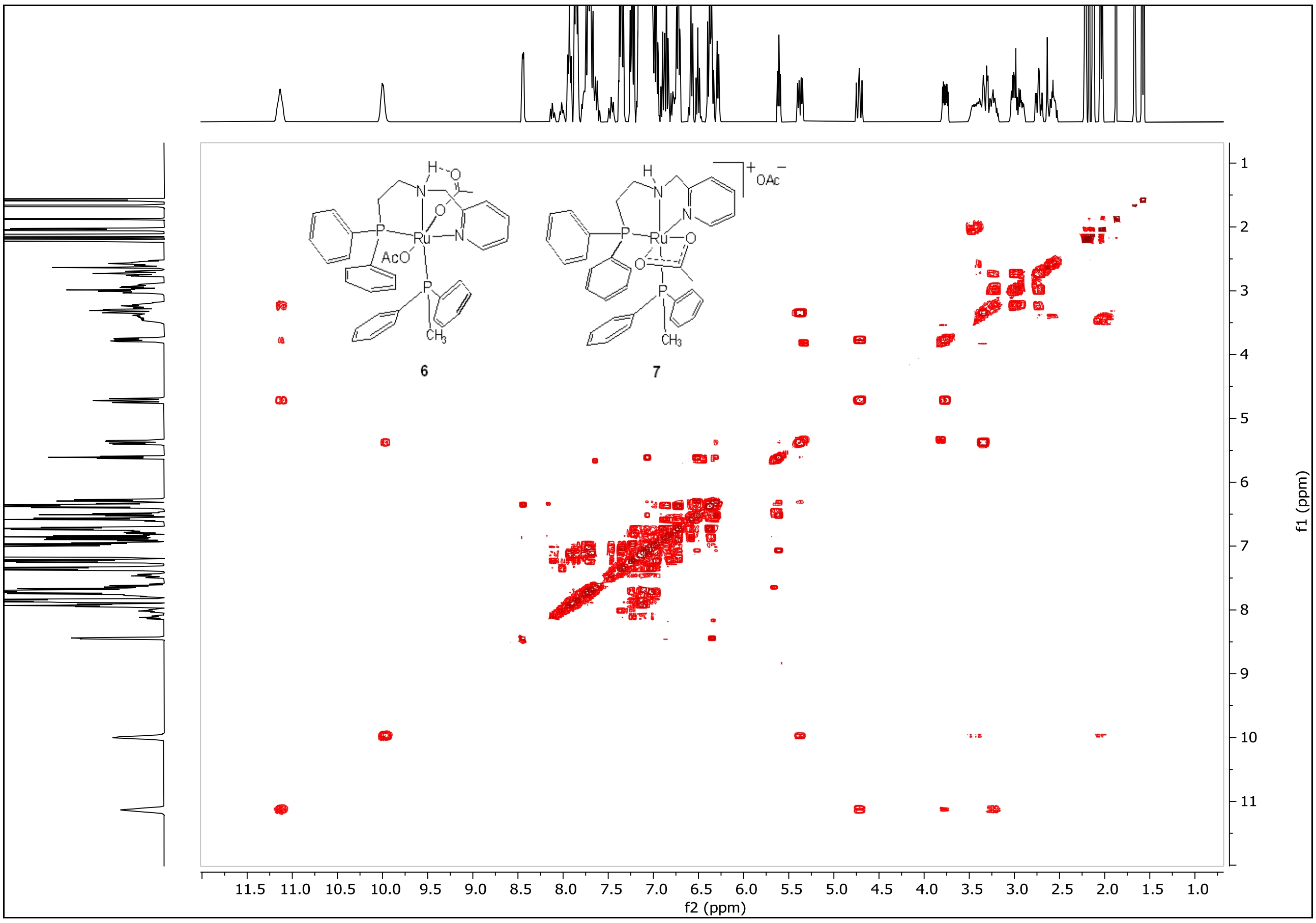

Figure S48. ^{1}H-^{1}H COSY 2D NMR spectrum of the 1:1 mixture of *trans*-[Ru(η^1-OAc)$_2$(PNN^G)(PPh$_2$Me)] (**6**) and [Ru(η^2-OAc)(PNN^G)(PPh$_2$Me)]OAc (**7**) in toluene-d^8 at 25 °C.

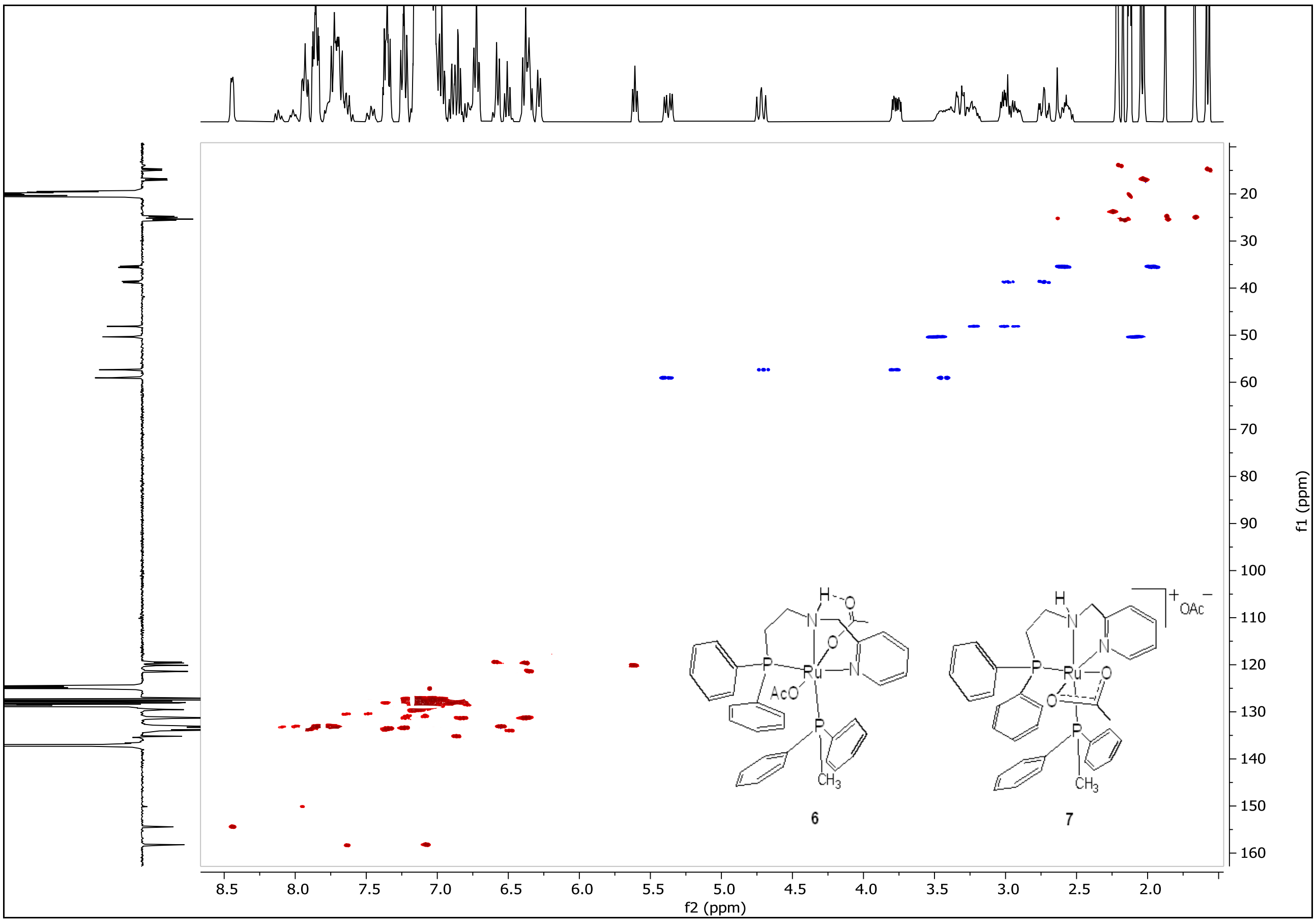

Figure S49. ^{1}H-^{13}C HSQC 2D NMR spectrum of the 1:1 mixture of *trans*-[Ru(η^1-OAc)$_2$(PNN^G)(PPh$_2$Me)] (**6**) and [Ru(η^2-OAc)(PNN^G)(PPh$_2$Me)]OAc (**7**) in toluene-d^8 at 25 °C.

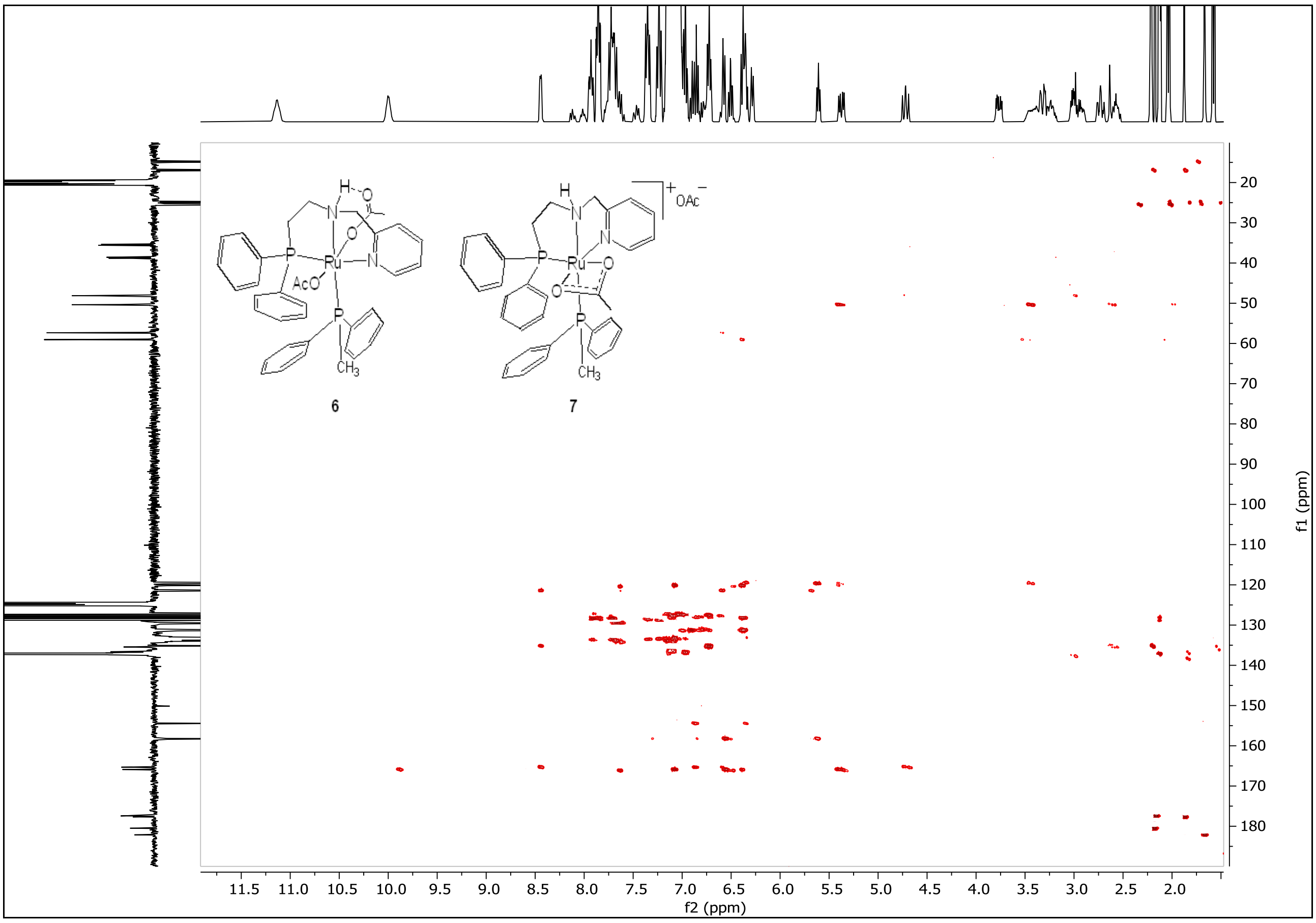

Figure S50. ${}^{1}H$-${}^{13}C$ HMBC 2D NMR spectrum of the 1:1 mixture of *trans*-[Ru(η^{1}-OAc)$_2$(PNN^{G})(PPh$_2$Me)] (**6**) and [Ru(η^{2}-OAc)(PNN^{G})(PPh$_2$Me)]OAc (**7**) in toluene-d^{8} at 25 °C.

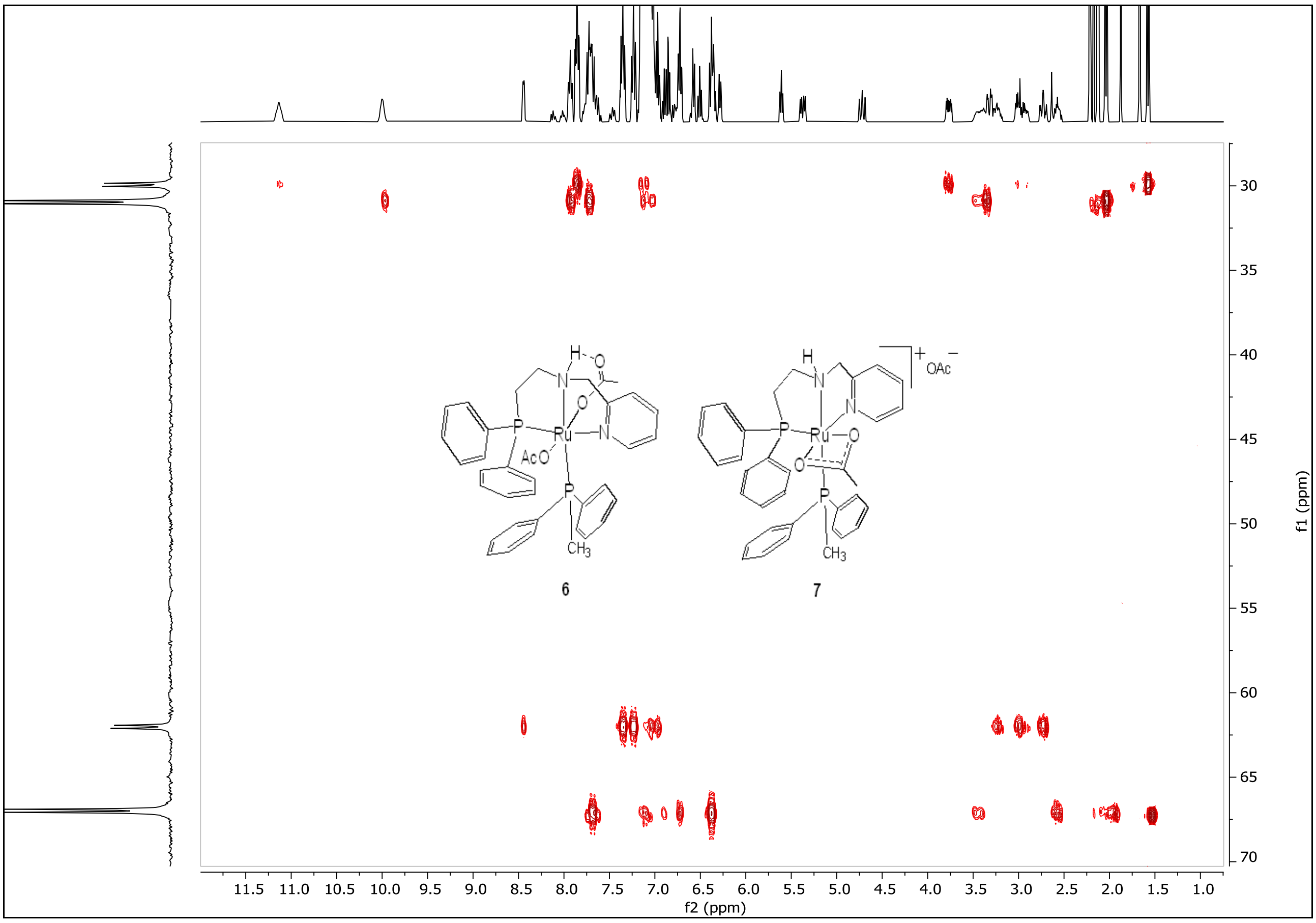

Figure S51. ^{1}H-^{31}P HMBC 2D NMR spectrum of the 1:1 mixture of *trans*-[Ru(η^1-OAc)$_2$(PNN^G)(PPh$_2$Me)] (**6**) and [Ru(η^2-OAc)(PNN^G)(PPh$_2$Me)]OAc (**7**) in toluene-d^8 at 25 °C.

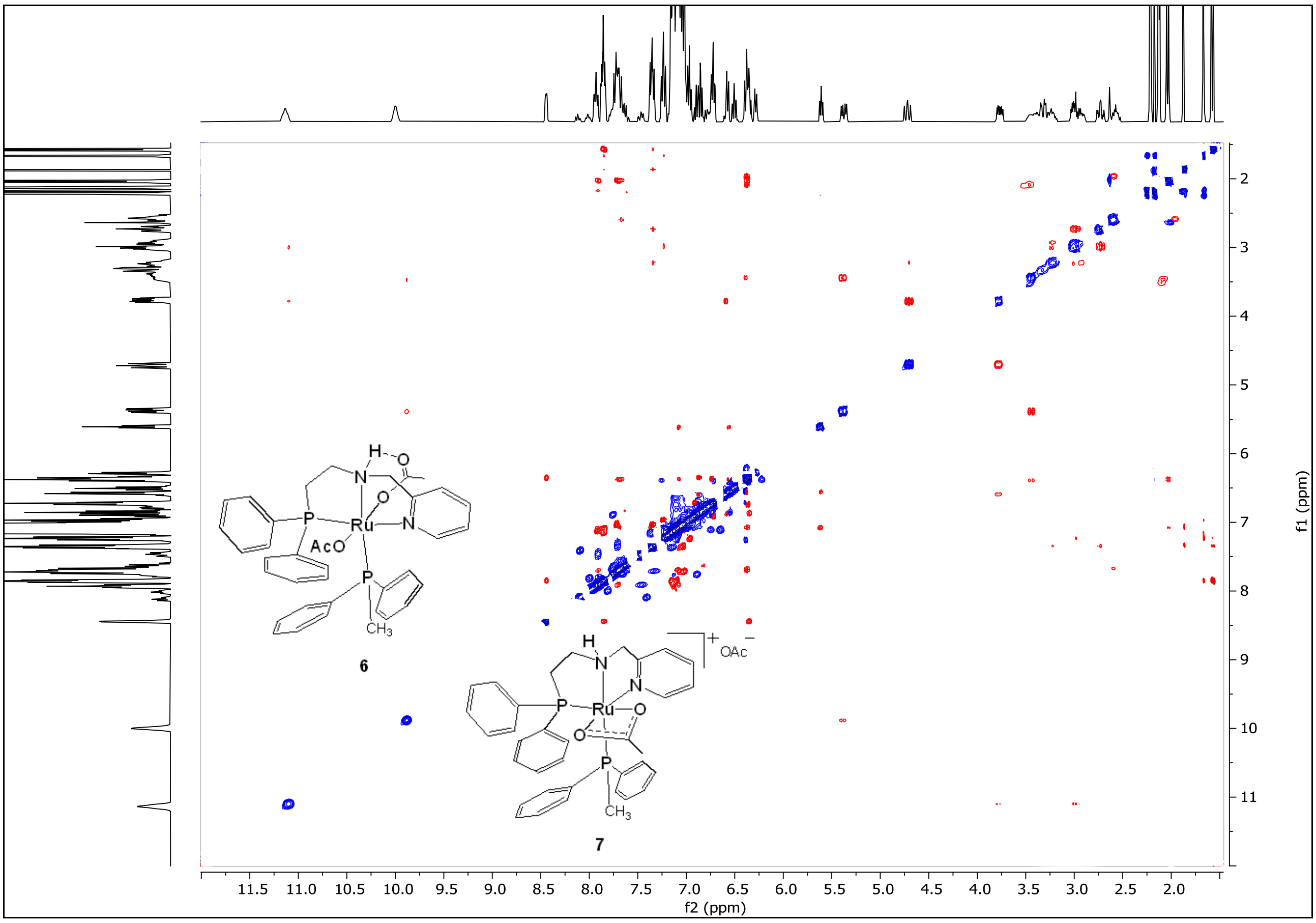

Figure S52. ^{1}H-^{1}H NOESY 2D NMR spectrum of the 1:1 mixture of *trans*-[Ru(η^1-OAc)$_2$(PNN^G)(PPh$_2$Me)] (**6**) and [Ru(η^2-OAc)(PNN^G)(PPh$_2$Me)]OAc (**7**) in toluene-*d*^{8} at 25 °C.

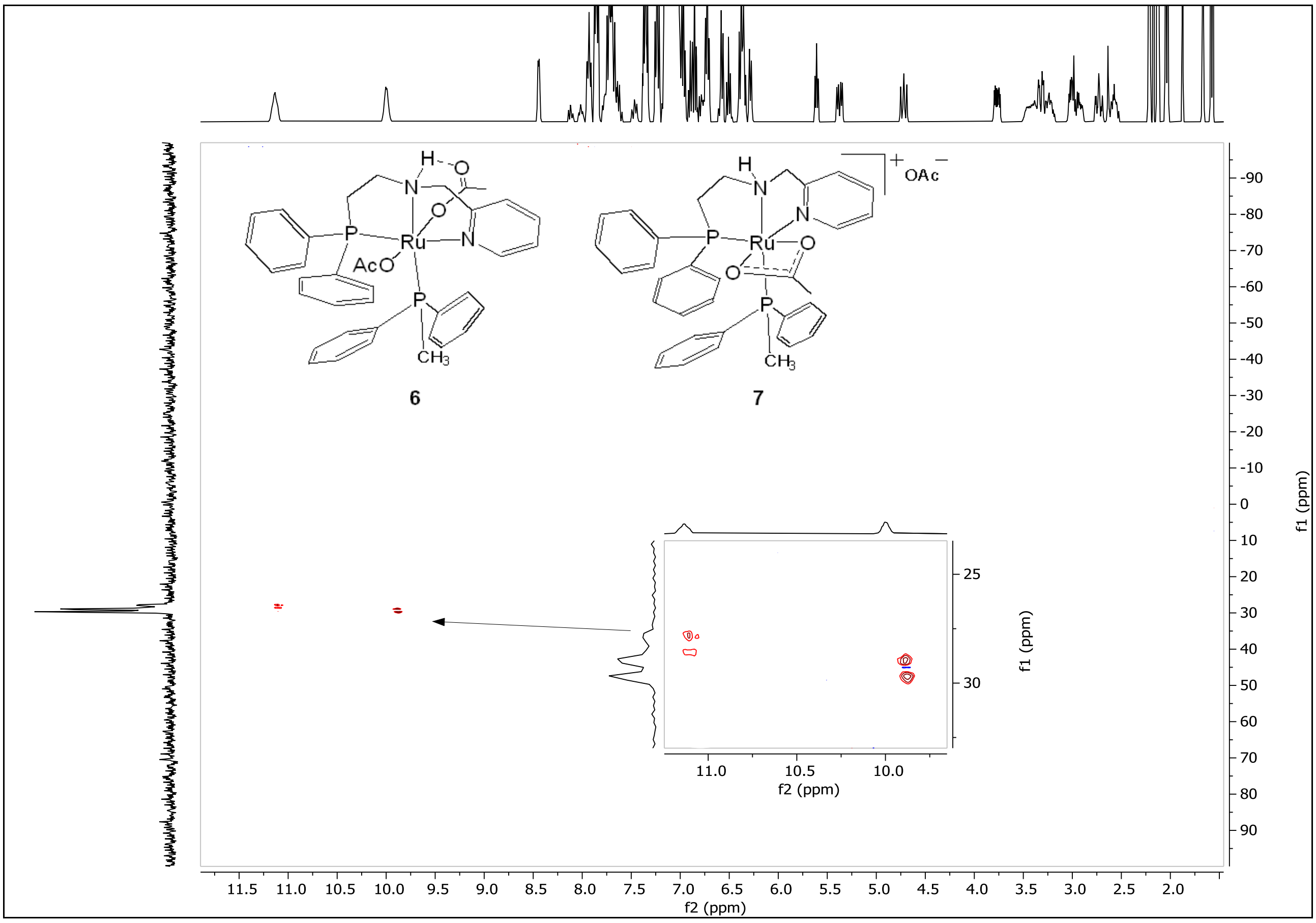

Figure S53. ^{1}H-^{15}N HSQC 2D NMR spectrum of the 1:1 mixture of *trans*-[Ru(η^1-OAc)$_2$(PNN^G)(PPh$_2$Me)] (**6**) and [Ru(η^2-OAc)(PNN^G)(PPh$_2$Me)]OAc (**7**) in toluene-d^8 at 25 °C.

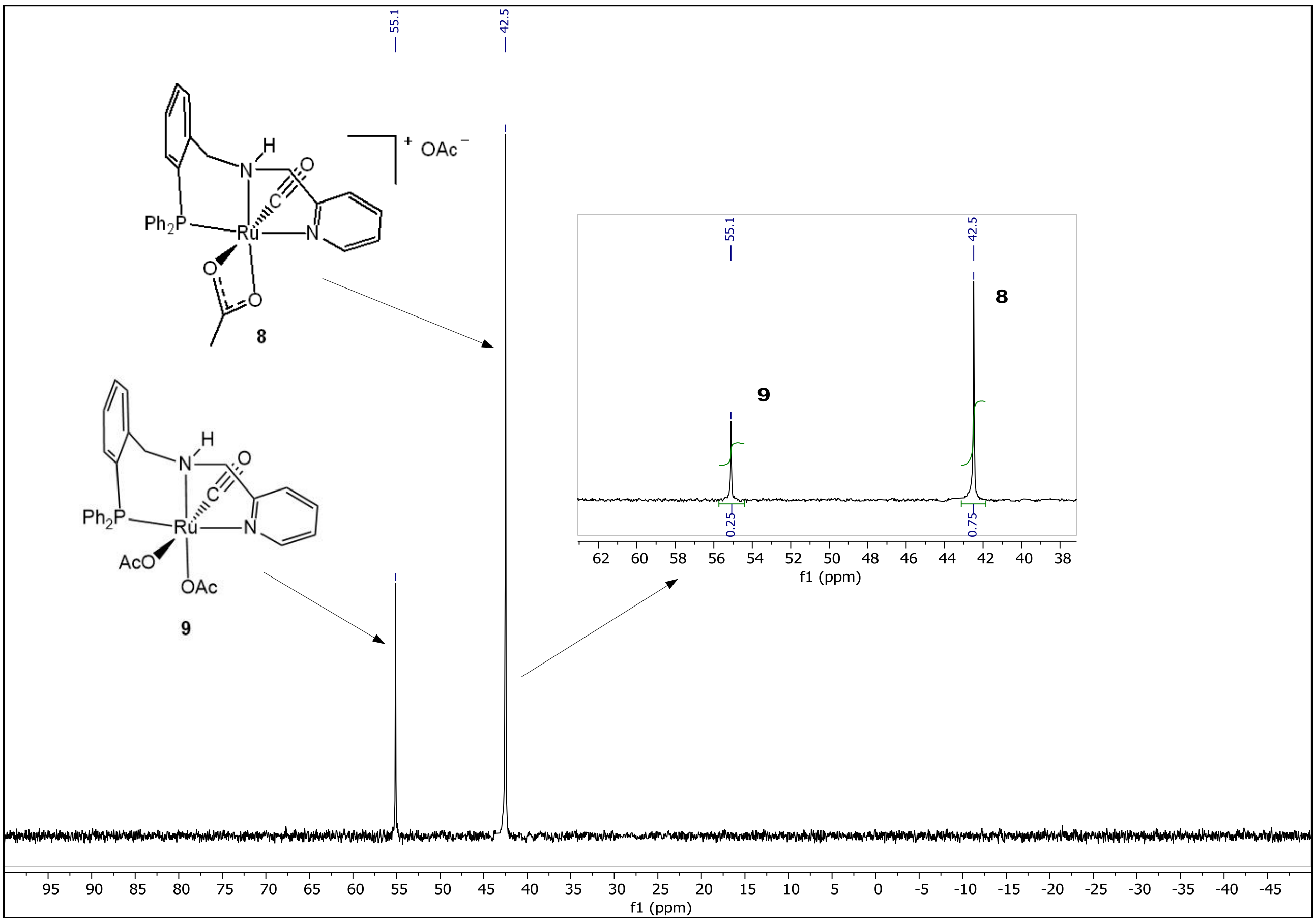

Figure S54. $^{31}P\{^{1}H\}$ NMR spectrum (162.0 MHz) of [Ru(η^2-OAc)(CO)(PNN')]OAc (**8**) and *cis*-[Ru(η^1-OAc)$_2$(CO)(PNN')] (**9**) in CD_3OD at 25 °C.

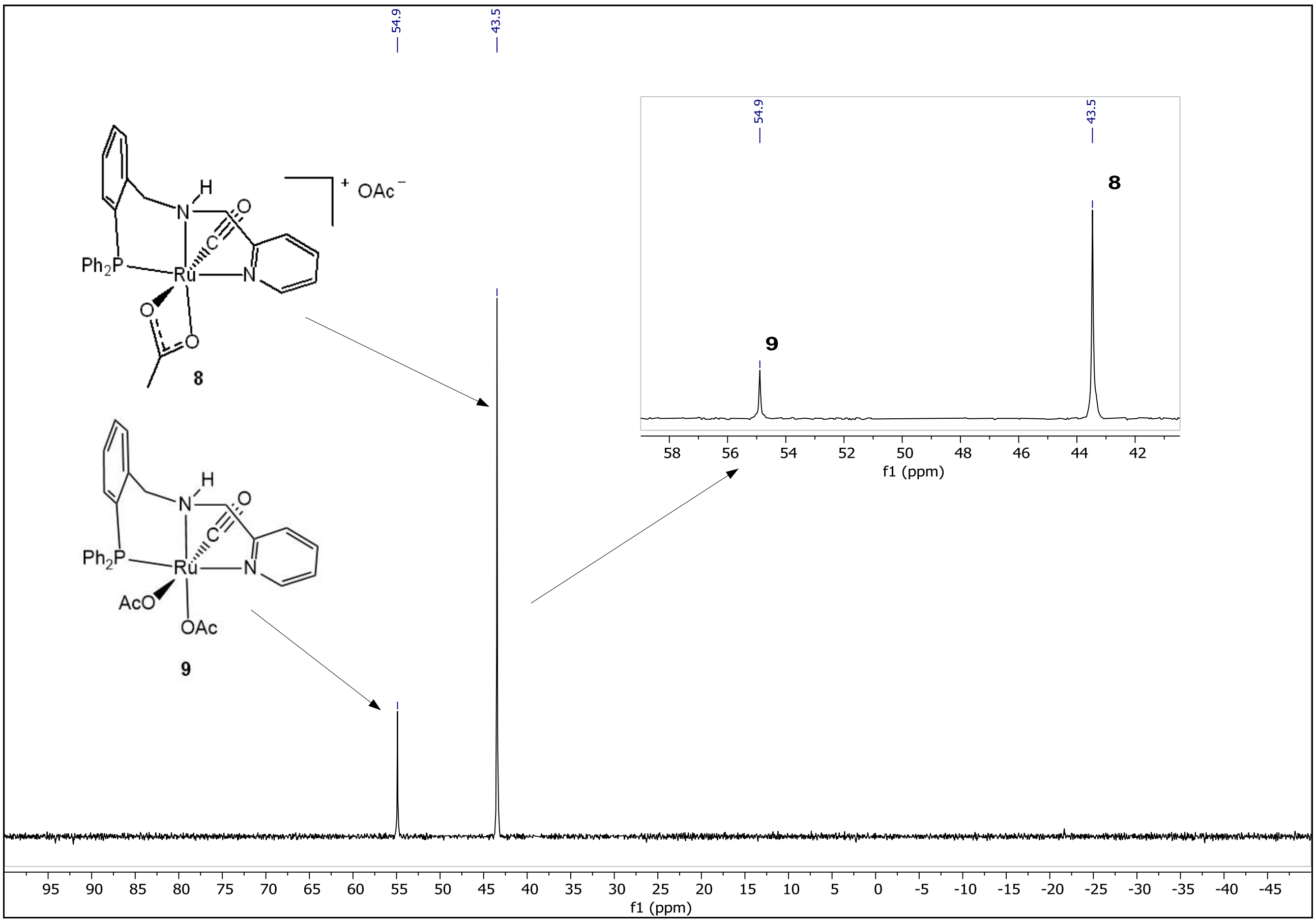

Figure S55. $^{31}P\{^{1}H\}$ NMR spectrum (162.0 MHz) of [Ru(η^2-OAc)(CO)(PNN')]OAc (**8**) and *cis*-[Ru(η^1-OAc)$_2$(CO)(PNN')] (**9**) in CD_2Cl_2 at 25 °C.

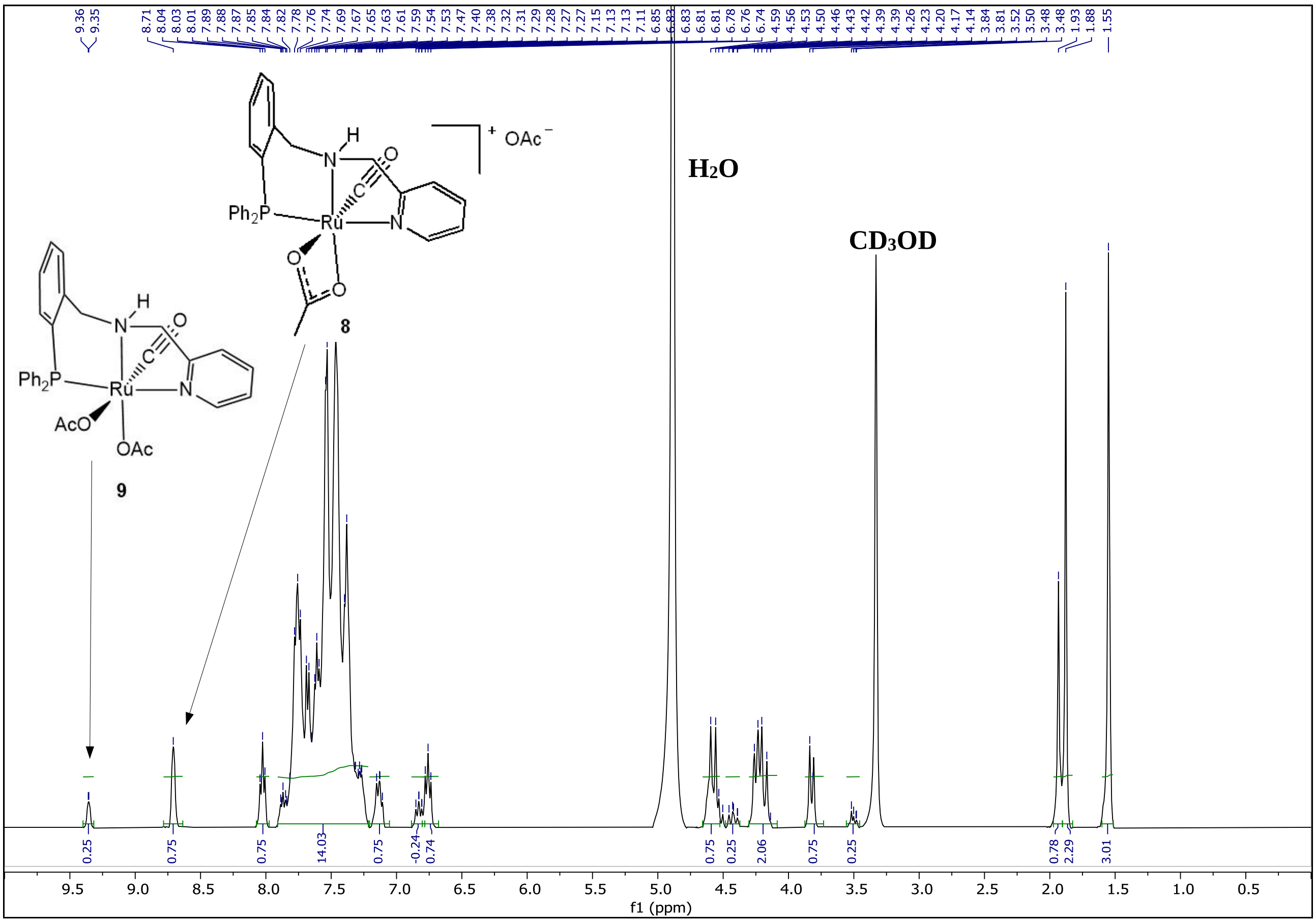

Figure S56. ^{1}H NMR spectrum (400.1 MHz) of [Ru(η^2-OAc)(CO)(PNN')]OAc (**8**) and *cis*-[Ru(η^1-OAc)$_2$(CO)(PNN')] (**9**) in CD_3OD at 25 °C.

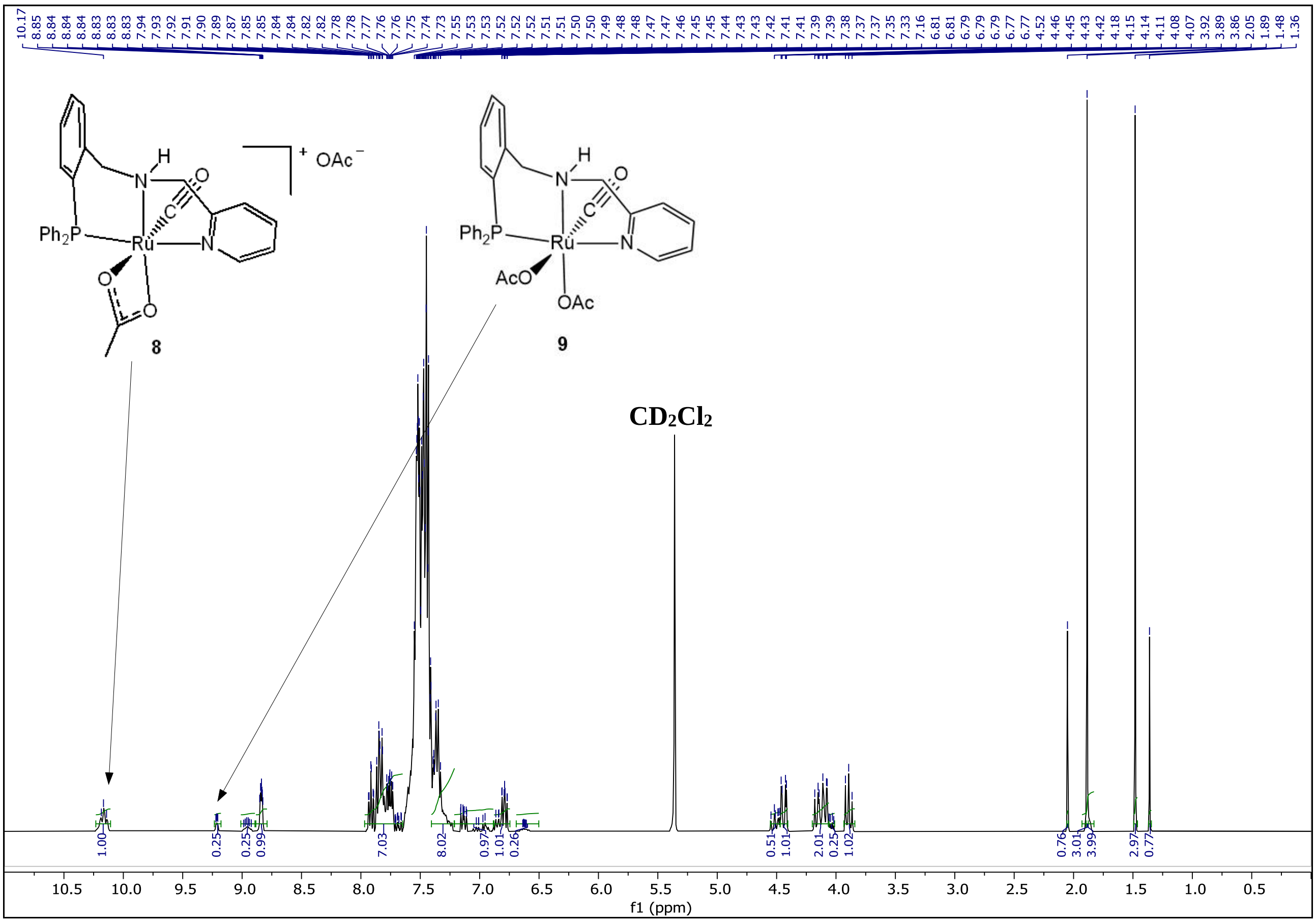

Figure S57. ^{1}H NMR spectrum (400.1 MHz) of [Ru(η^2-OAc)(CO)(PNN')]OAc (**8**) and *cis*-[Ru(η^1-OAc)$_2$(CO)(PNN')] (**9**) in CD_2Cl_2 at 25 °C.

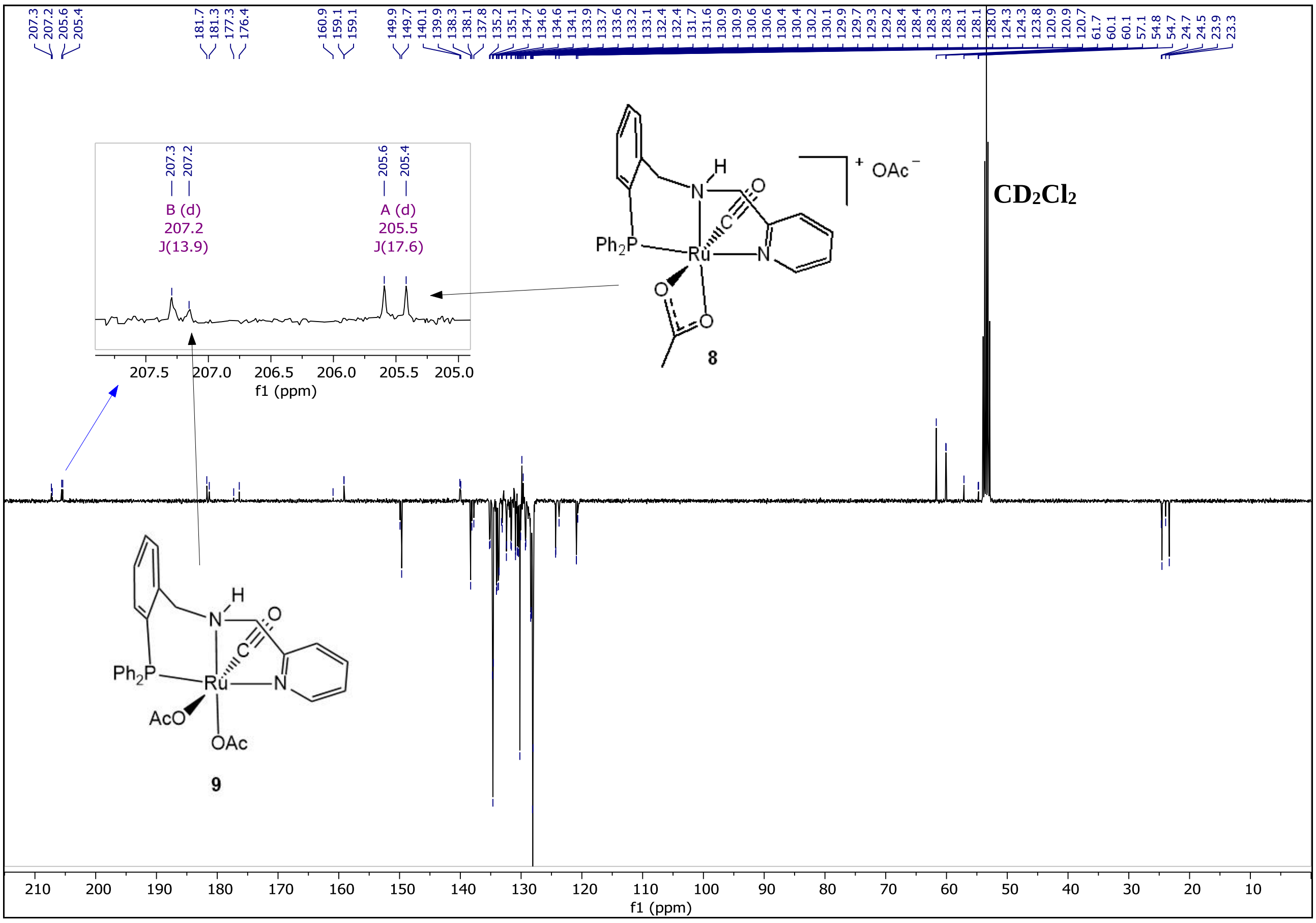

Figure S58. $^{13}C\{^{1}H\}$ NMR spectrum (100.6 MHz) of [Ru(η^2-OAc)(CO)(PNN')]OAc (**8**) and *cis*-[Ru(η^1-OAc)$_2$(CO)(PNN')] (**9**) in CD_2Cl_2 at 25 °C.

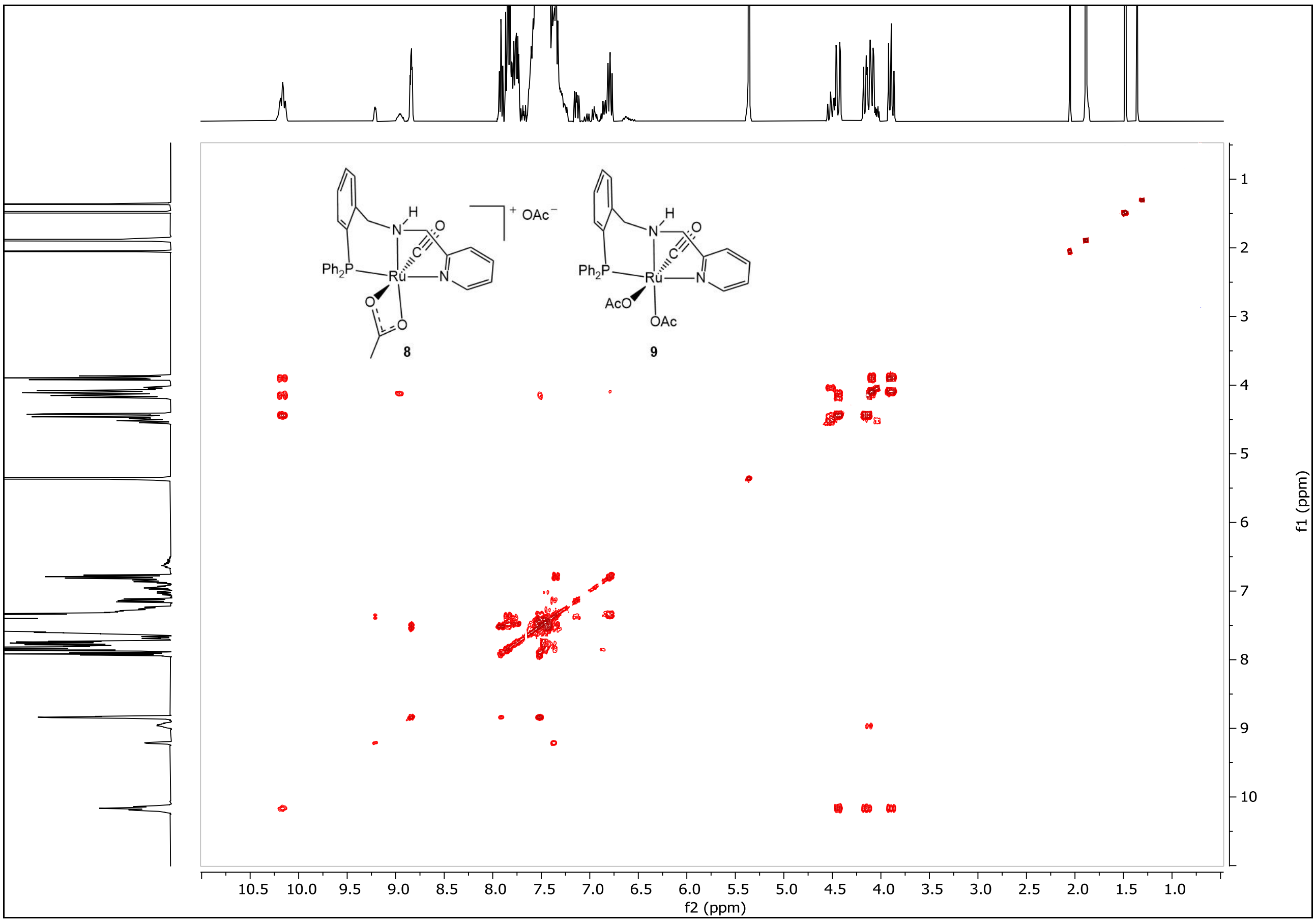

Figure S59. ^1H-^1H COSY 2D NMR spectrum of [Ru(η^2-OAc)(CO)(PNN')]OAc (**8**) and *cis*-[Ru(η^1-OAc)$_2$(CO)(PNN')] (**9**) in CD_2Cl_2 at 25 °C.

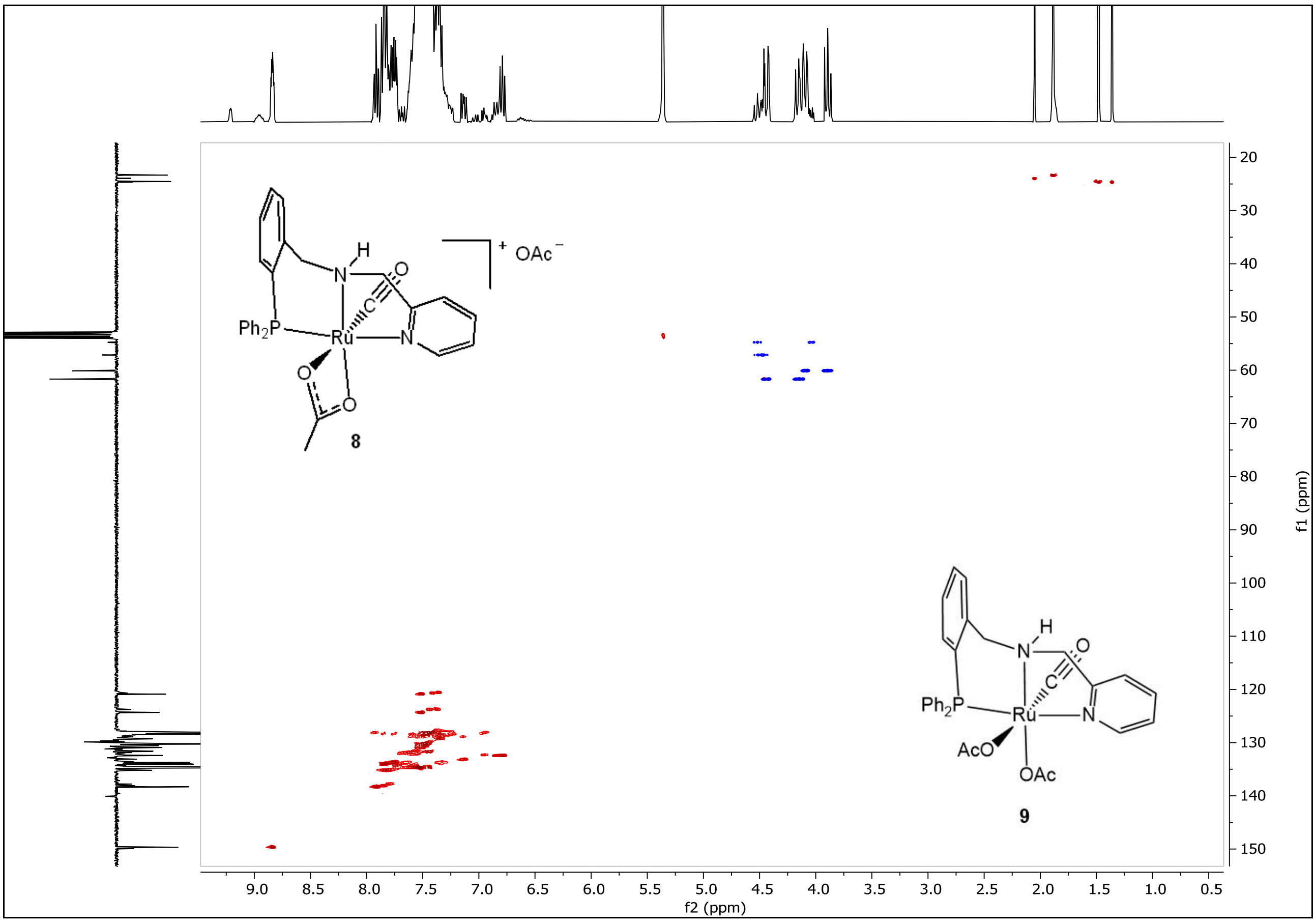

Figure S60. ^{1}H-^{13}C HSQC 2D NMR spectrum of [Ru(η^2-OAc)(CO)(PNN')]OAc (**8**) and *cis*-[Ru(η^1-OAc)$_2$(CO)(PNN')] (**9**) in CD_2Cl_2 at 25 °C.

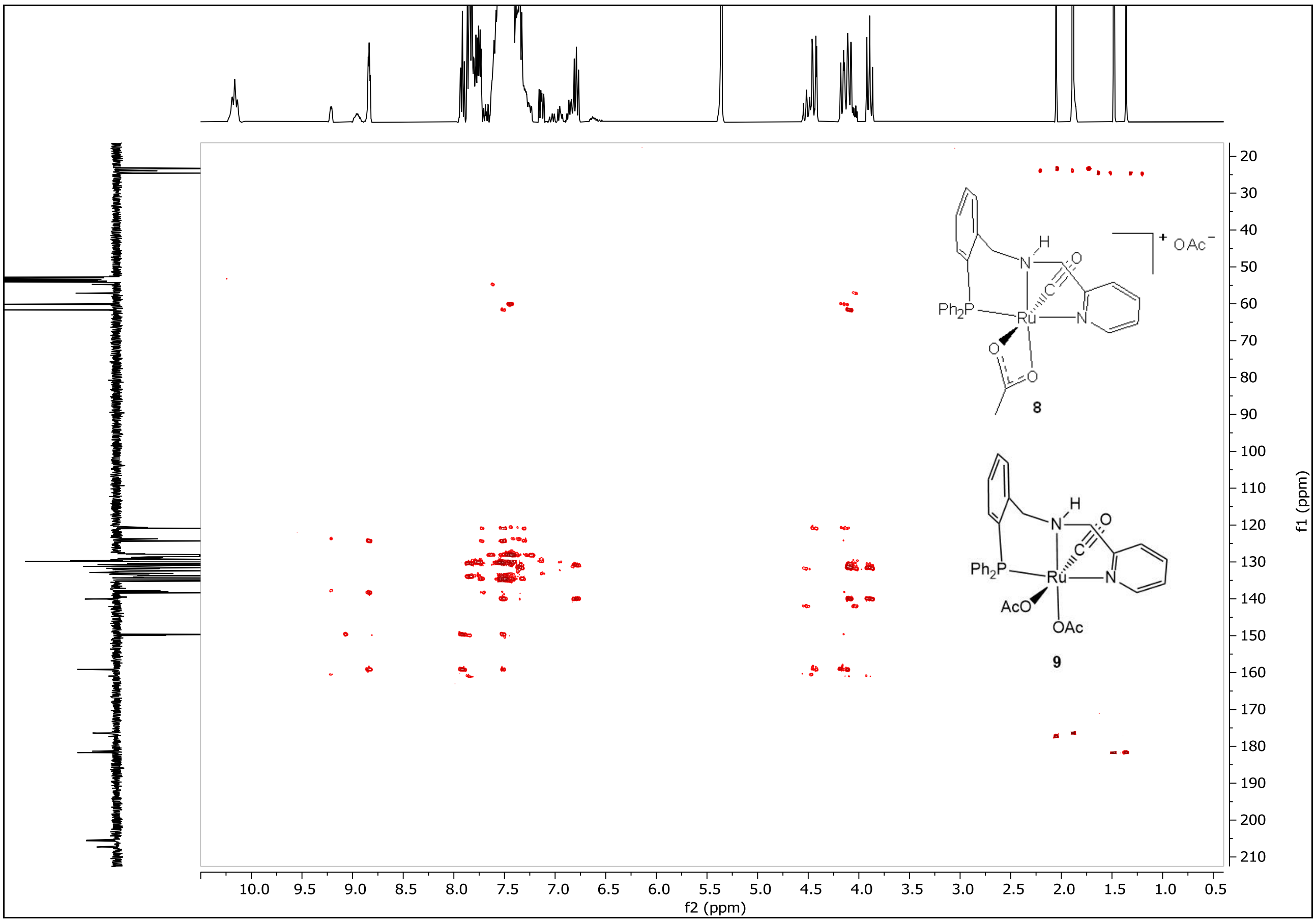

Figure S61. ^{1}H-^{13}C HMBC 2D NMR spectrum of [Ru(η^2-OAc)(CO)(PNN')]OAc (**8**) and *cis*-[Ru(η^1-OAc)$_2$(CO)(PNN')] (**9**) in CD_2Cl_2 at 25 °C.

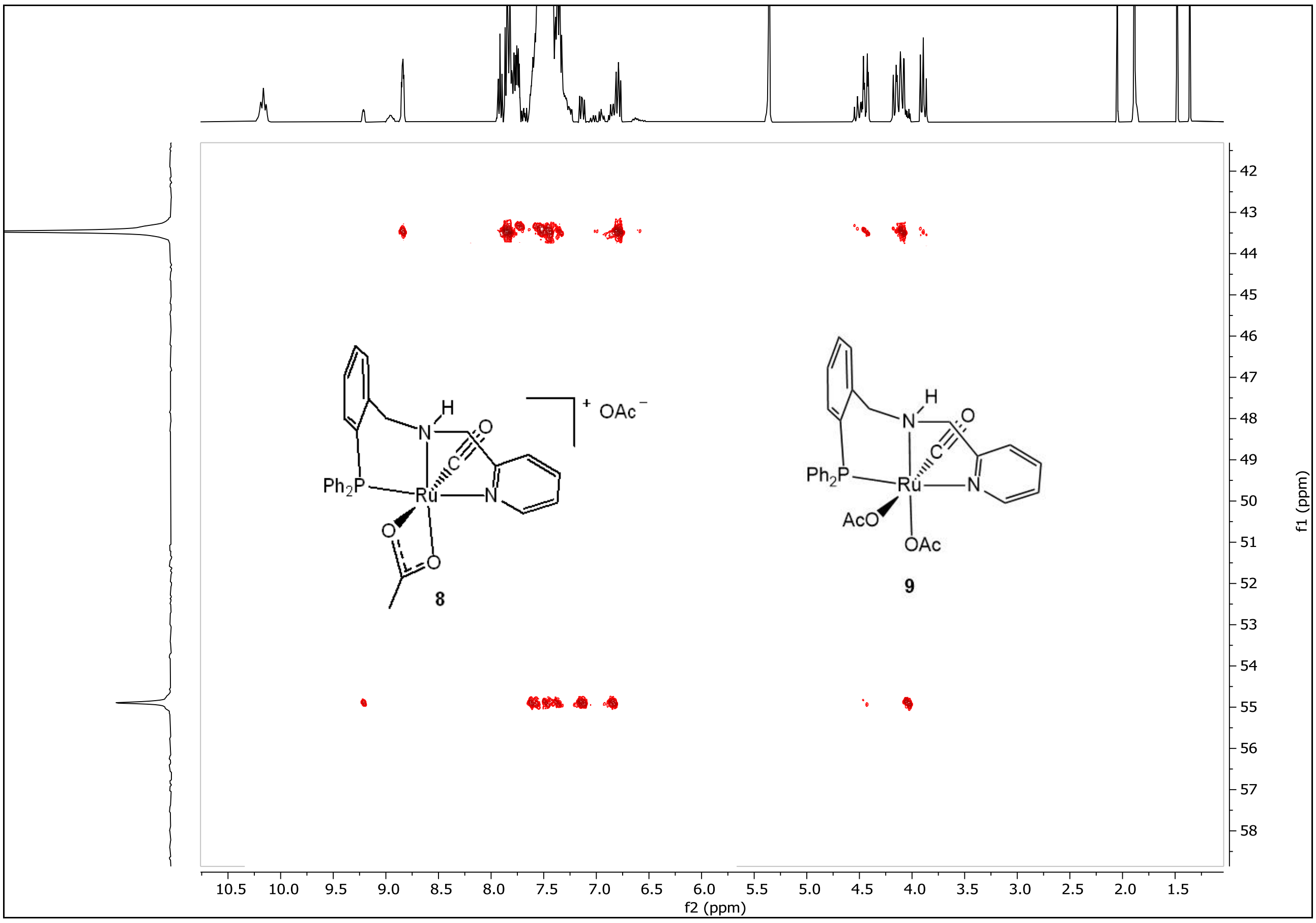

Figure S62. ^{1}H-^{31}P HMBC 2D NMR spectrum of [Ru(η^2-OAc)(CO)(PNN')]OAc (**8**) and *cis*-[Ru(η^1-OAc)$_2$(CO)(PNN')] (**9**) in CD_2Cl_2 at 25 °C.

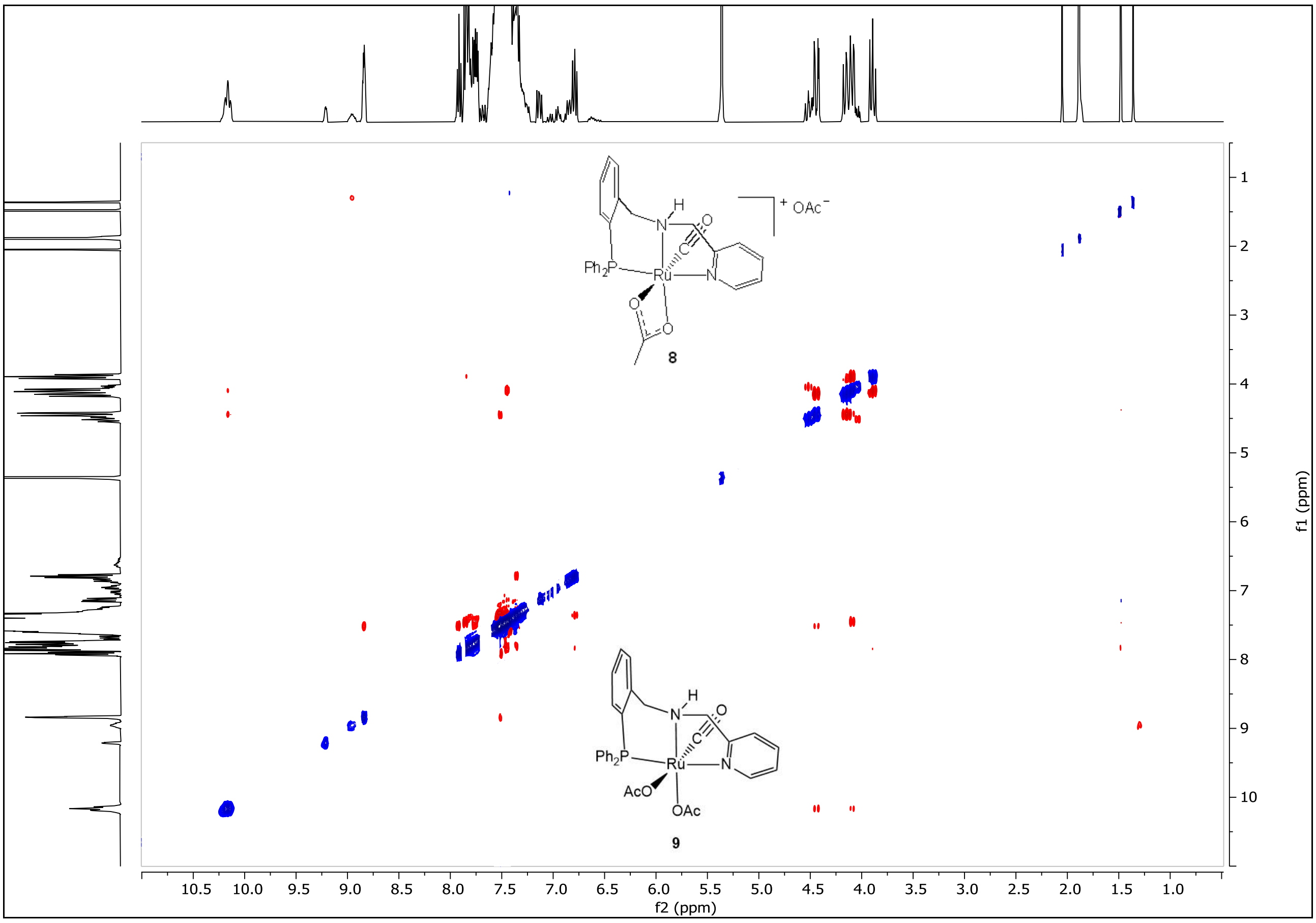

Figure S63. ^{1}H-^{1}H NOESY 2D NMR spectrum of [Ru(η^2-OAc)(CO)(PNN')]OAc (**8**) and *cis*-[Ru(η^1-OAc)$_2$(CO)(PNN')] (**9**) in CD_2Cl_2 at 25 °C.

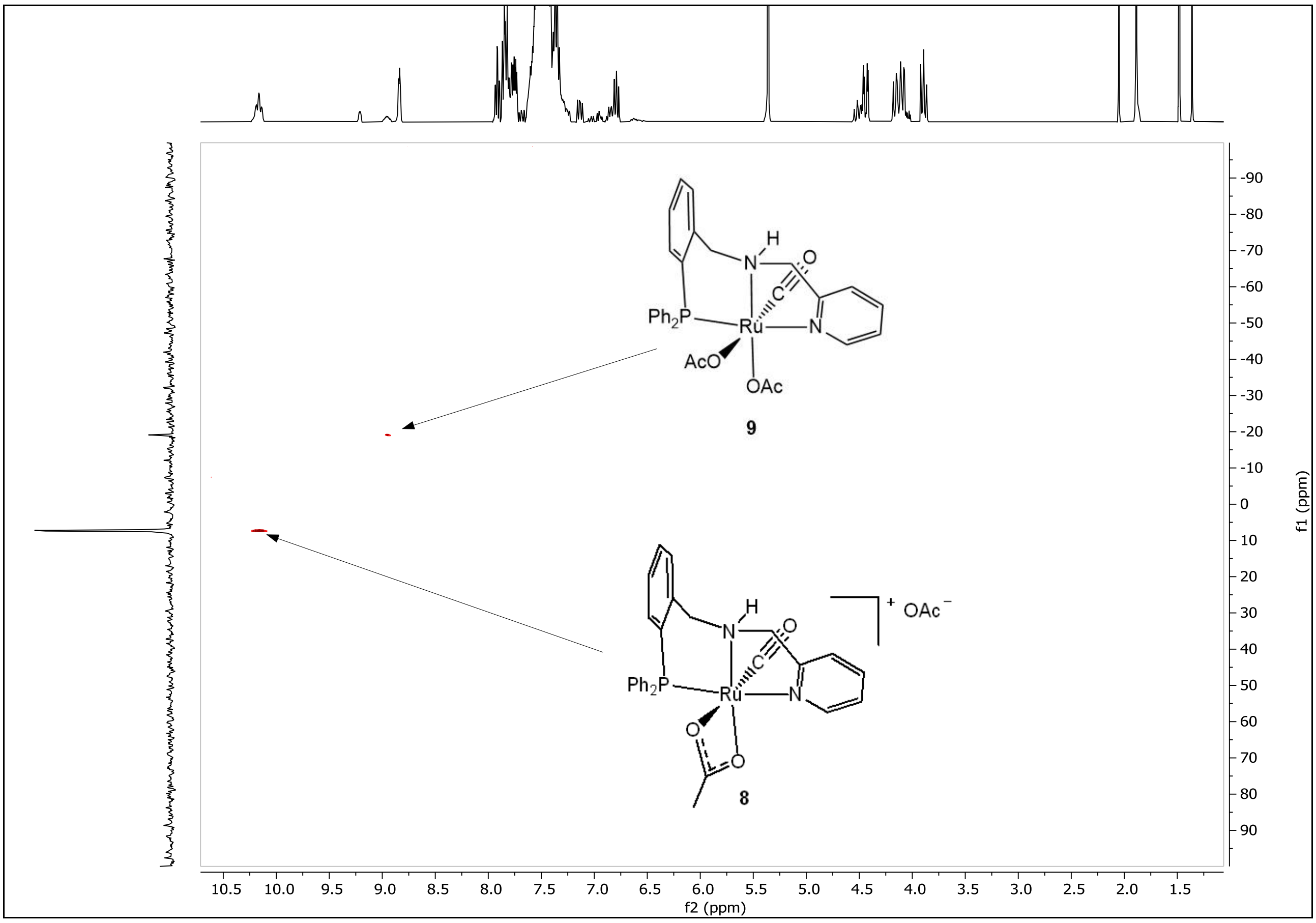

Figure S64. ^{1}H-^{15}N HSQC 2D NMR spectrum of [Ru(η^2-OAc)(CO)(PNN')]OAc (**8**) and *cis*-[Ru(η^1-OAc)$_2$(CO)(PNN')] (**9**) in CD_2Cl_2 at 25 °C.

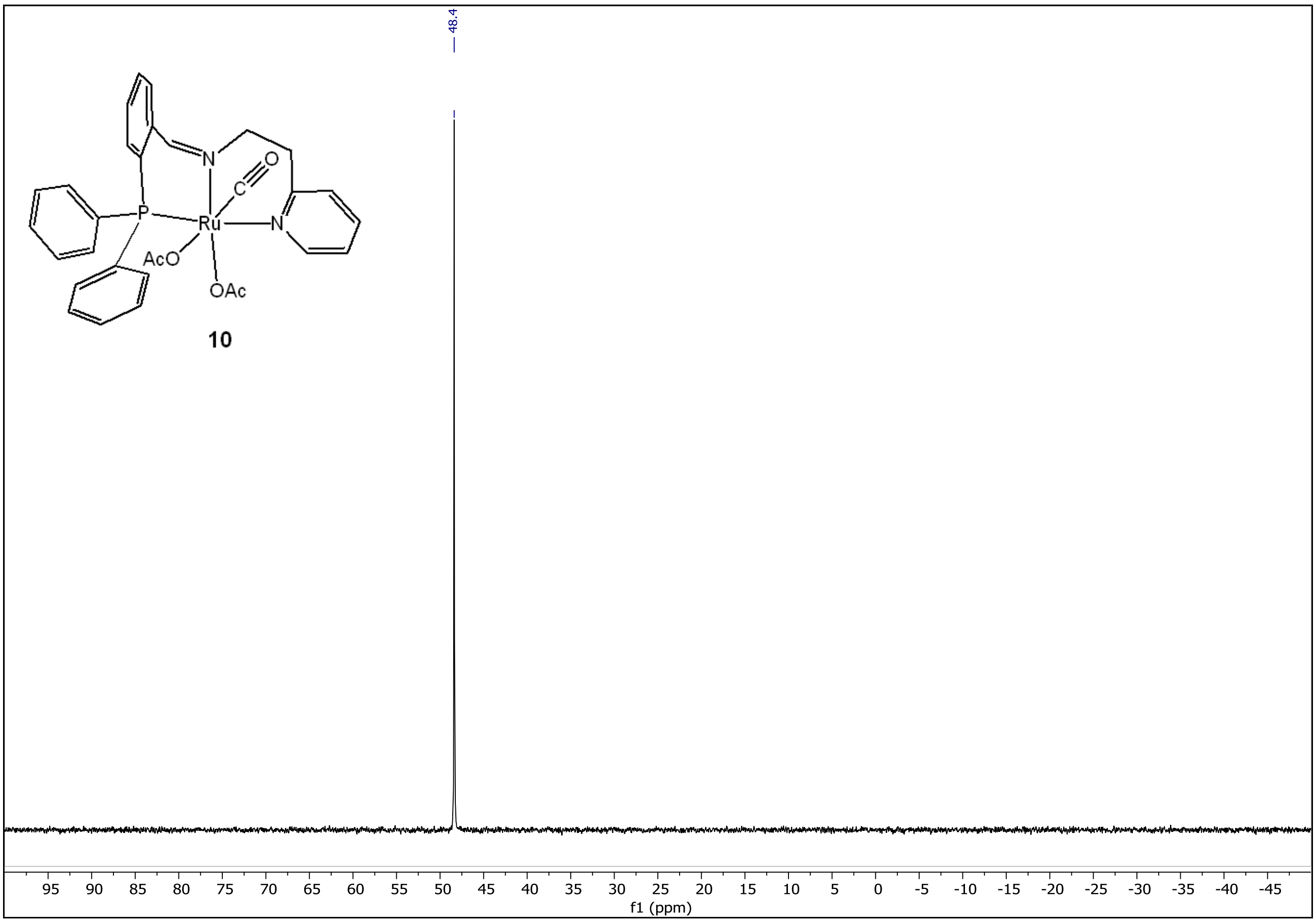

Figure S65. $^{31}P\{^{1}H\}$ NMR spectrum (162.0 MHz) of *cis*-[Ru(η^1-OAc)$_2$(CO)(PNN(C_2)$_{imine}$)] (**10**) in CD_2Cl_2 at 25 °C.

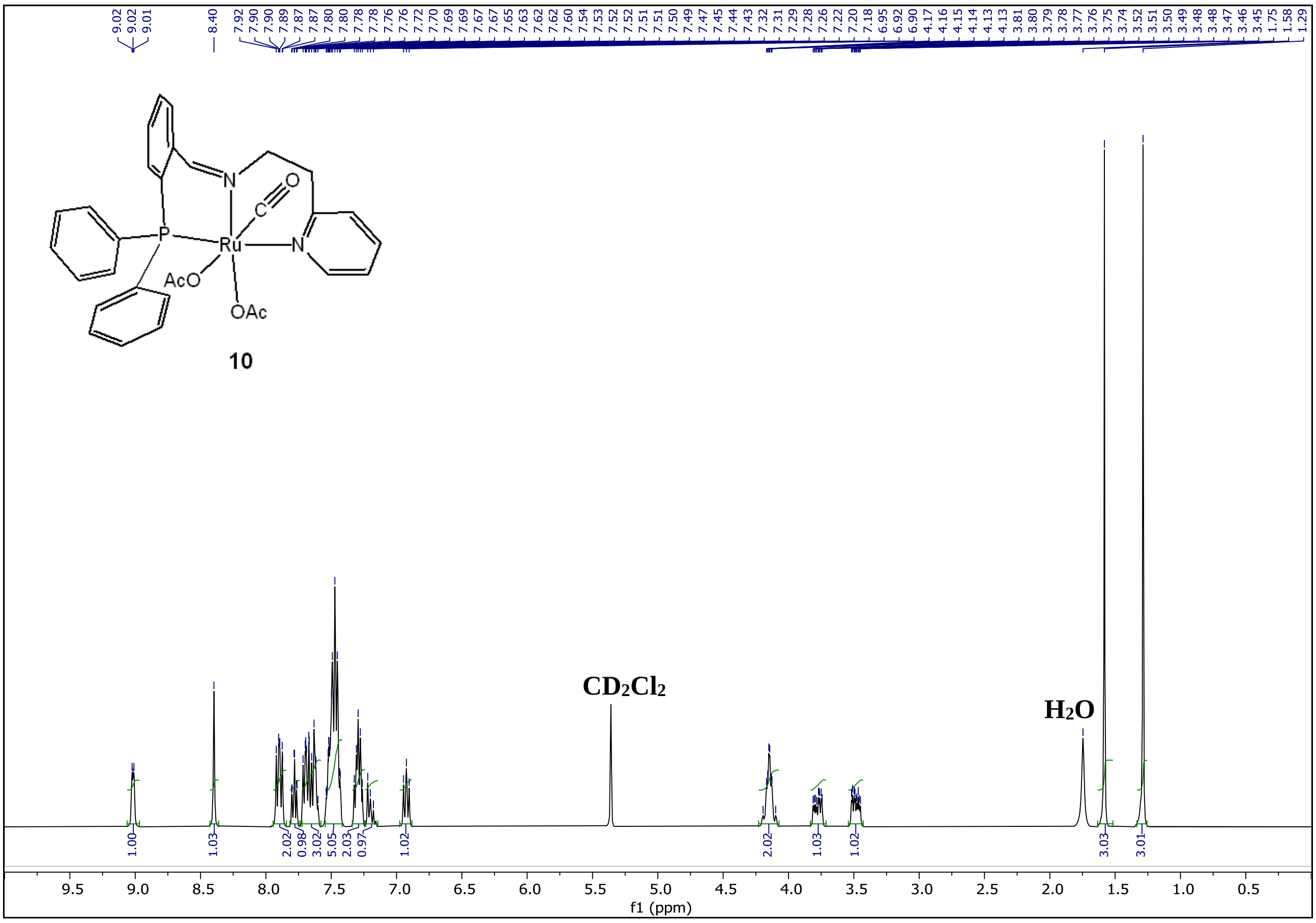

Figure S66. ^{1}H NMR spectrum (400.1 MHz) of *cis*-[Ru(η^1-OAc)$_2$(CO)(PNN(C_2)$_{imine}$)] (**10**) in CD_2Cl_2 at 25 °C.

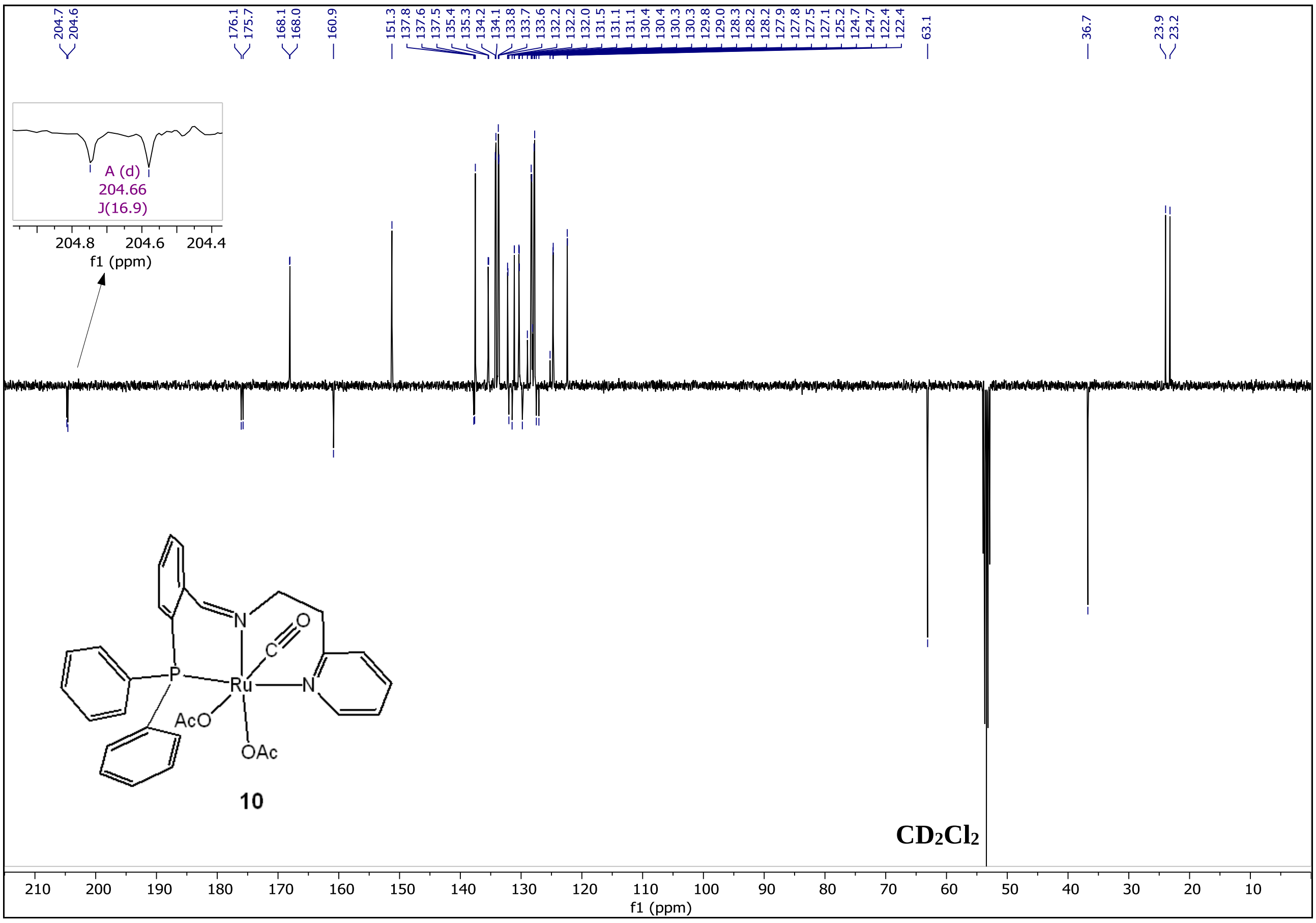

Figure S67. $^{13}C\{^{1}H\}$ DEPTQ NMR spectrum (100.6 MHz) of *cis*-[Ru(η^1-OAc)$_2$(CO)(PNN(C_2)$_{imine}$)] (**10**) in CD_2Cl_2 at 25 °C.

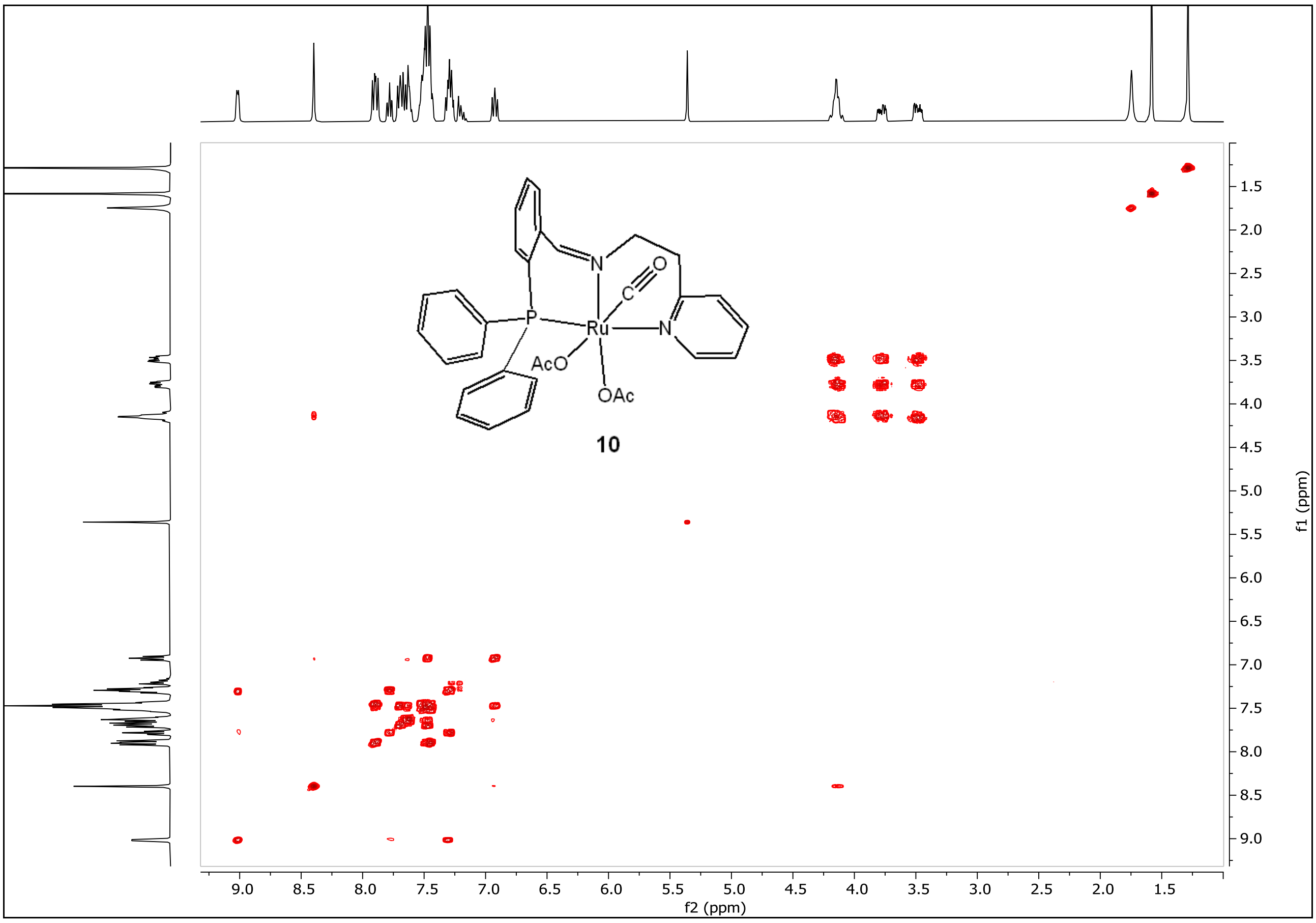

Figure S68. ^{1}H-^{1}H COSY 2D NMR spectrum of *cis*-[Ru(η^1-OAc)$_2$(CO)(PNN(C$_2$)$_{imine}$)] (**10**) in CD_2Cl_2 at 25 °C.

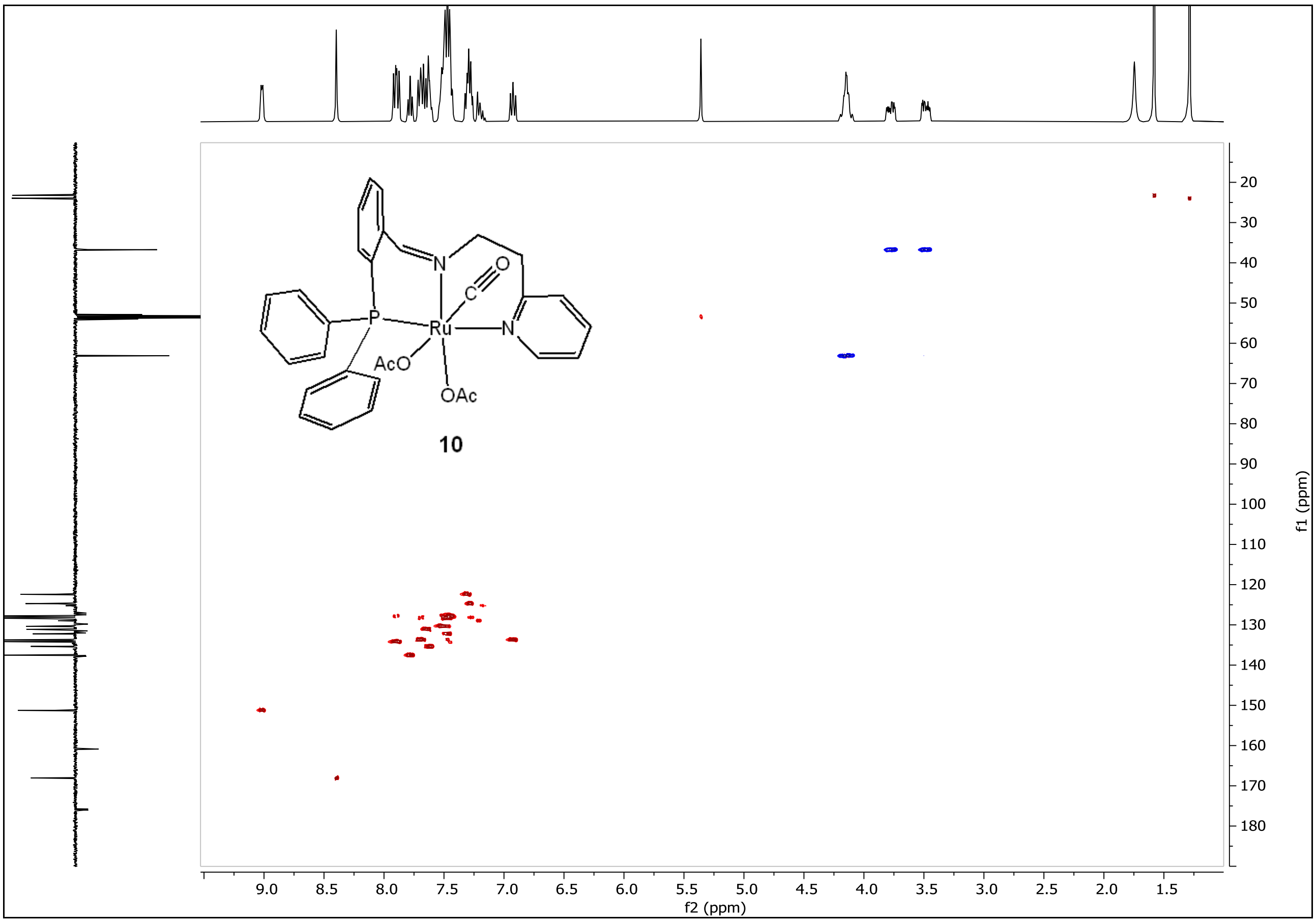

Figure S69. ^{1}H-^{13}C HSQC 2D NMR spectrum of *cis*-[Ru(η^1-OAc)$_2$(CO)(PNN(C_2)$_{imine}$)] (**10**) in CD_2Cl_2 at 25 °C.

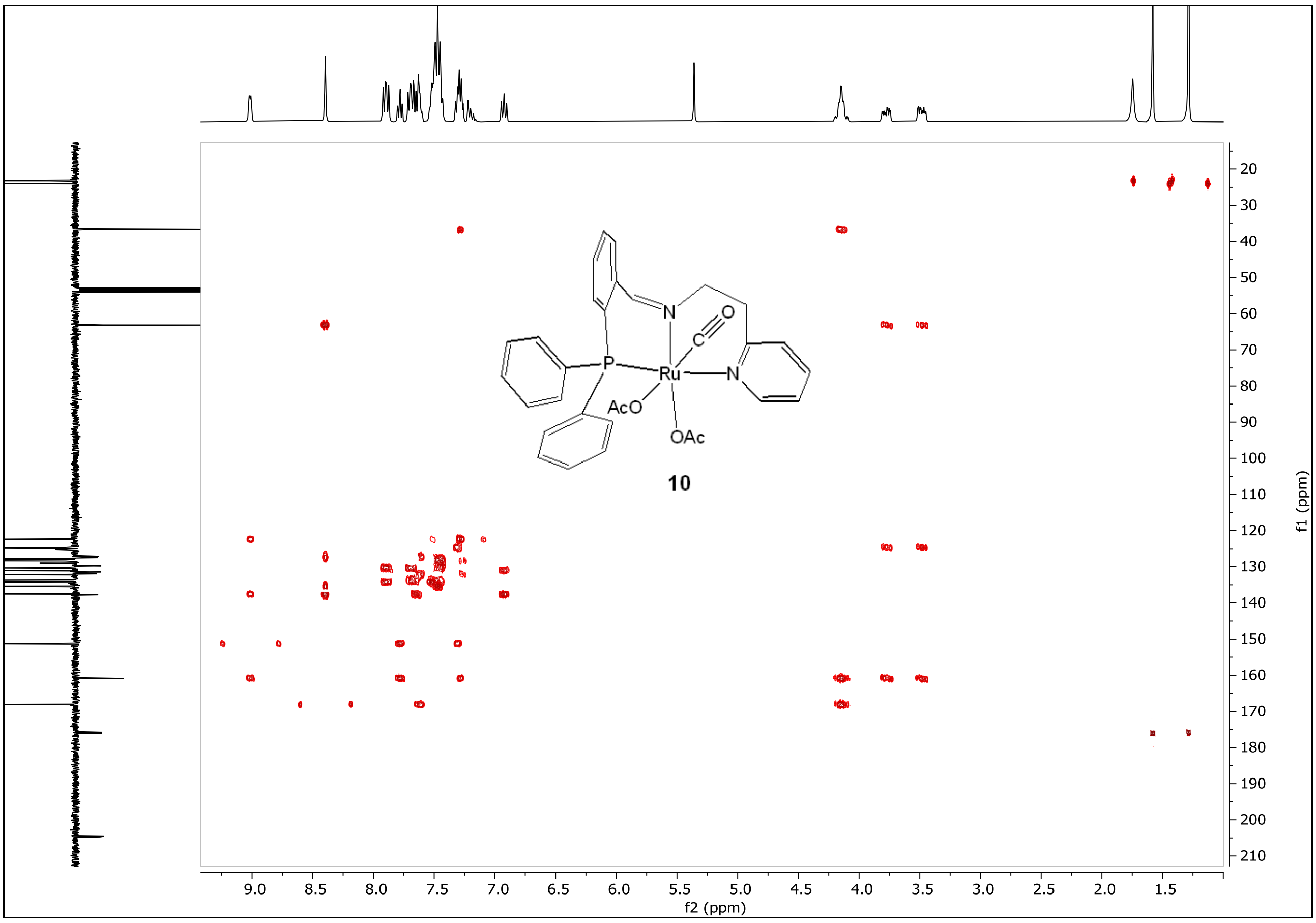

Figure S70. ^{1}H-^{13}C HMBC 2D NMR spectrum of *cis*-[Ru(η^1-OAc)$_2$(CO)(PNN(C_2)$_{imine}$)] (**10**) in CD_2Cl_2 at 25 °C.

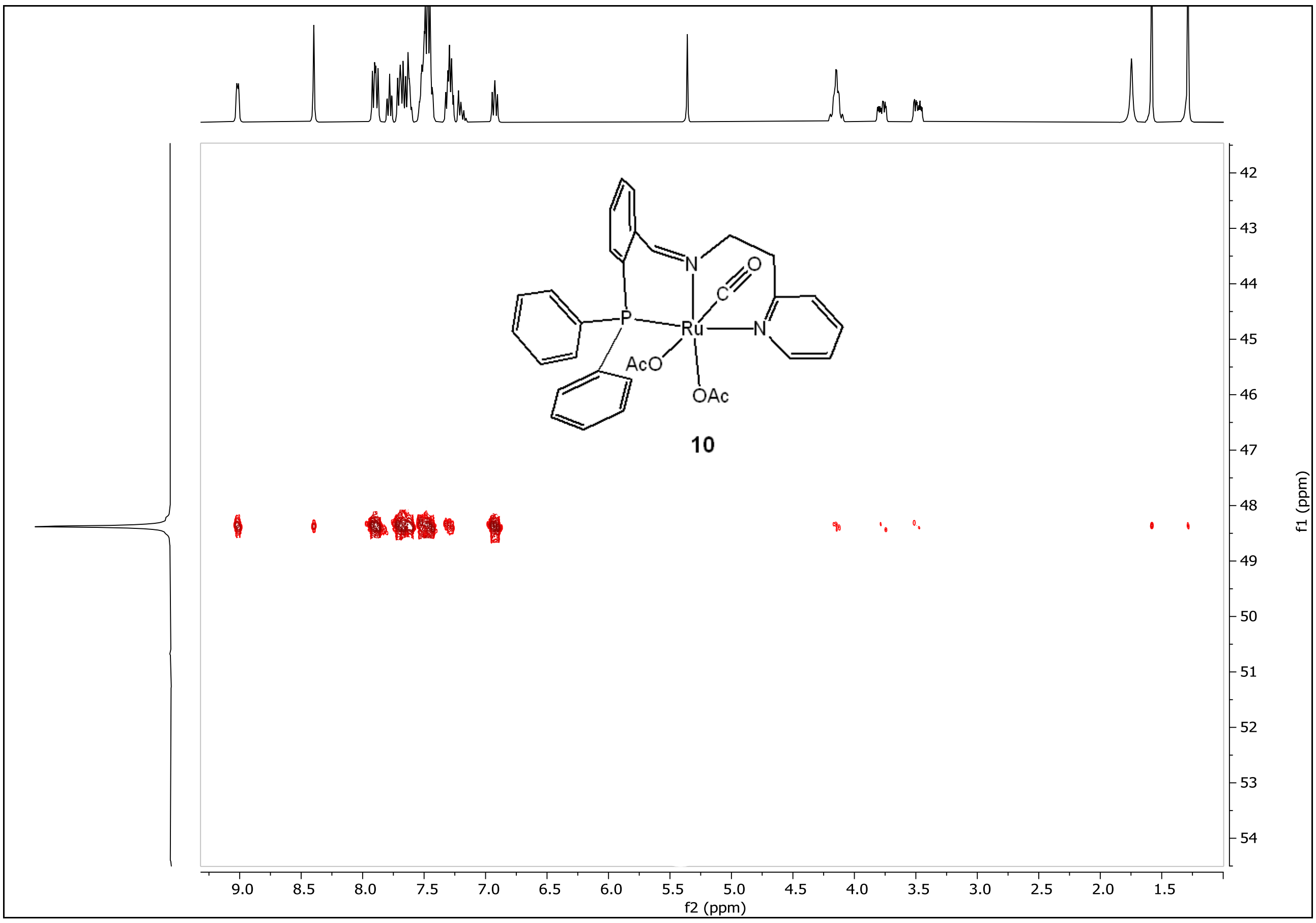

Figure S71. ^{1}H-^{31}P HMBC 2D NMR spectrum of *cis*-[Ru(η^1-OAc)$_2$(CO)(PNN(C_2)$_{imine}$)] (**10**) in CD_2Cl_2 at 25 °C.

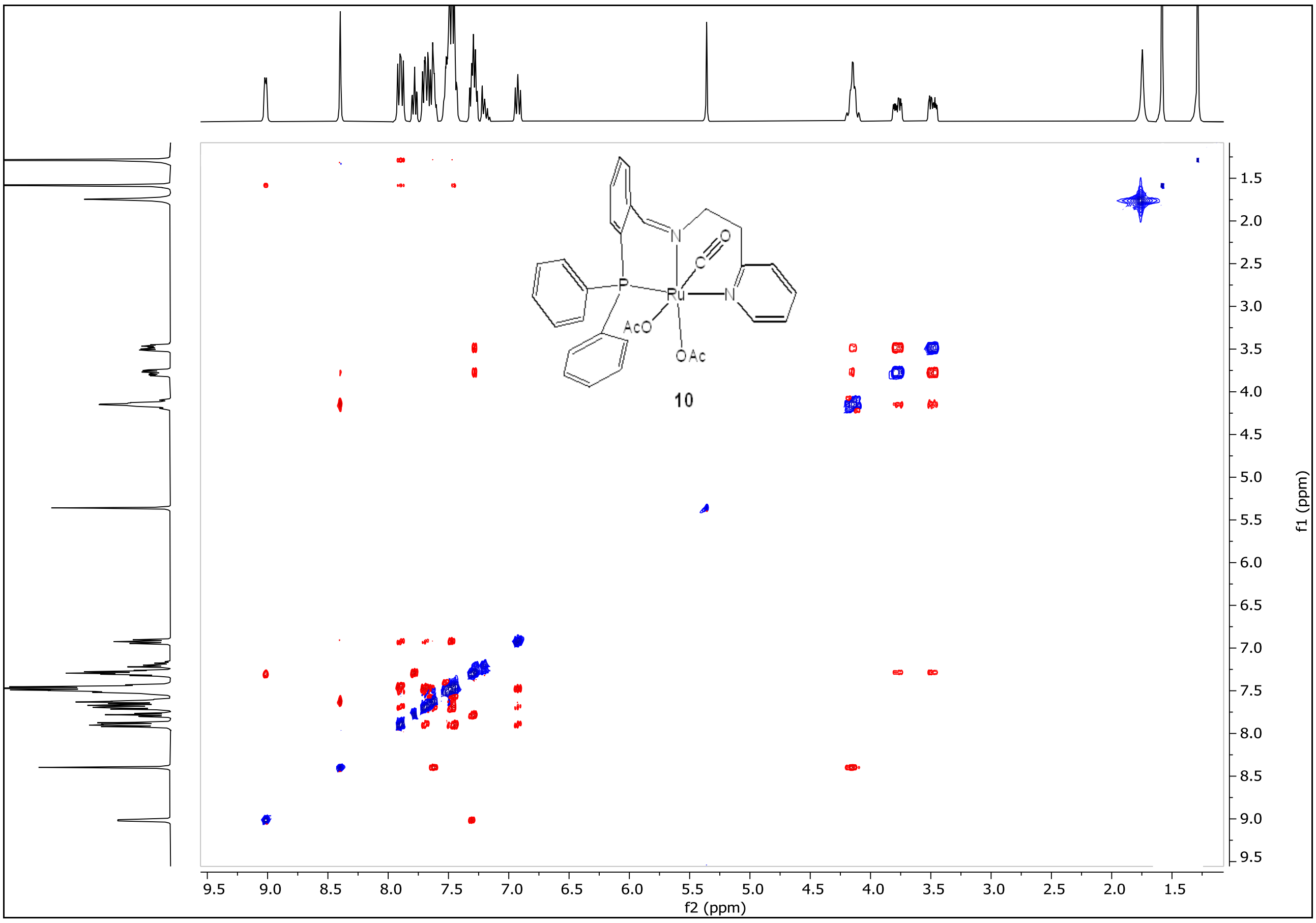

Figure S72. ^{1}H-^{1}H NOESY 2D NMR spectrum of *cis*-[Ru(η^1-OAc)$_2$(CO)(PNN(C$_2$)$_{imine}$)] (**10**) in CD_2Cl_2 at 25 °C.

Table S1. Further data regarding the catalytic hydrogenation of methyl decanoate **a** with complexes **1-10**, *trans*-[$RuCl_2$(PNN')(PPh_3)] and *trans*-[$RuCl_2$(PNN^G)(PPh_3)] (S/C = 1000-100000), NaOMe 50 mol% at 27.5 bar of H_2 at 40-90 °C, with or without solvent, 16 h

Entry	Complex	S/C	T [°C]	Conv.[a] [%]
1	**1**	50000	40	20.7
2	**1**	50000	90	5.3
3	**2**	50000	90	18.5
4	**2**	100000	40	25.9
5	**3**	50000	40	1.5
6	**3**	50000	90	1.5
7	**4**	50000	40	19.3
8	**4**	50000	90	4.5
9	**5**[b]	1000	40	61.1
10	**5**[b]	1000	90	100
11	**5**	50000	40	47.2
12	**5**	50000	90	16.6
13	**6/7**	50000	40	3.7
14	**6/7**	50000	90	14.9
15	**8/9**[c]	1000	90	91.4
16	**8/9**	10000	40	1.7
17	**8/9**	10000	90	8.0
18	**8/9**	50000	40	0.0
19	**8/9**	50000	90	1.1
20	**10**	10000	40	2.5
21	**10**	10000	90	5.0
22	[$RuCl_2$(PNN')(PPh_3)]	10000	40	7.2
23	[$RuCl_2$(PNN')(PPh_3)]	50000	40	0.0
24	[$RuCl_2$(PNN')(PPh_3)]	50000	90	5.8
25	[$RuCl_2$(PNN^G)(PPh_3)]	10000	90	92.5
26	[$RuCl_2$(PNN^G)(PPh_3)]	50000	40	91,9
27	[$RuCl_2$(PNN^G)(PPh_3)]	50000	90	24.8
28	[$RuCl_2$(PNN^G)(PPh_3)]	100000	40	62.1

[a] Determined by GC analyses. [b] Using MeTHF as solvent. [c] Using MeTHF as solvent and K_3PO_4 as base.

Table S2. Further data regarding the catalytic hydrogenation of methyl and ethyl esters with **2** and *trans*-[$RuCl_2$(PNN[G])(PPh_3)], with base 50 mol% at 27.5 bar of H_2 and in absence of solvent after 16 h.

Entry	Complex	Substrate	S/C	base	T [°C)]	Conv.[a] [%]
1	[$RuCl_2$(PNN[G])(PPh_3)]	**b**	100000	NaOEt	40	87.2
2	[$RuCl_2$(PNN[G])(PPh_3)]		10000	NaOEt	30	80.1
3	[$RuCl_2$(PNN[G])(PPh_3)]	**d**	50000	NaOEt	40	94.1
4	[$RuCl_2$(PNN[G])(PPh_3)]		100000	NaOEt	40	81.5
5	**2**		100000	NaOMe	50	9.9
6	[$RuCl_2$(PNN[G])(PPh_3)]	**e**[b]	25000	NaOMe	60	100
7	[$RuCl_2$(PNN[G])(PPh_3)]		50000	NaOMe	50	89.8
8	[$RuCl_2$(PNN[G])(PPh_3)]		100000	NaOMe	50	30.6
9	[$RuCl_2$(PNN[G])(PPh_3)]	**f**[b]	50000	NaOEt	40	96.8
10	[$RuCl_2$(PNN[G])(PPh_3)]		100000	NaOEt	40	96.7
11	**2**		80000	NaOEt	40	67.6
12	**2**		100000	NaOMe	40	35.1
13	[$RuCl_2$(PNN[G])(PPh_3)]		10000	NaOEt	40	96.6
14	[$RuCl_2$(PNN[G])(PPh_3)]		25000	NaOEt	40	83.9
15	[$RuCl_2$(PNN[G])(PPh_3)]	**g**	50000	NaOEt	40	76.2
16	[$RuCl_2$(PNN[G])(PPh_3)]		80000	NaOEt	40	68.6
17	[$RuCl_2$(PNN[G])(PPh_3)]		100000	NaOEt	40	67.4
18	[$RuCl_2$(PNN[G])(PPh_3)]		100000	NaOMe	40	49.4
19	**2**	**h**	50000	NaOEt	40	82.8
20	[$RuCl_2$(PNN[G])(PPh_3)]		100000	NaOEt	40	100

[a] Determined by GC analyses. [b] Substrates **e** and **f** were of technical grade and contains methyl or ethyl oleate (> 76-80%) but also other polyunsaturated (methyl or ethyl linoleate and linolenate, etc.) and saturated esters (methyl or ethyl palmitate and stearate): the reported conversions are referred to the HY of the real methyl or ethyl oleate present in the starting material, although the reduction of the other esters to the respective alcohols (linoleyl alcohol, palmityl alcohol, etc.) has been also observed (see supp. info).

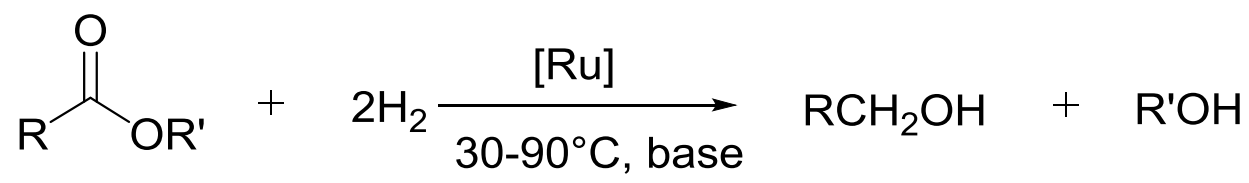

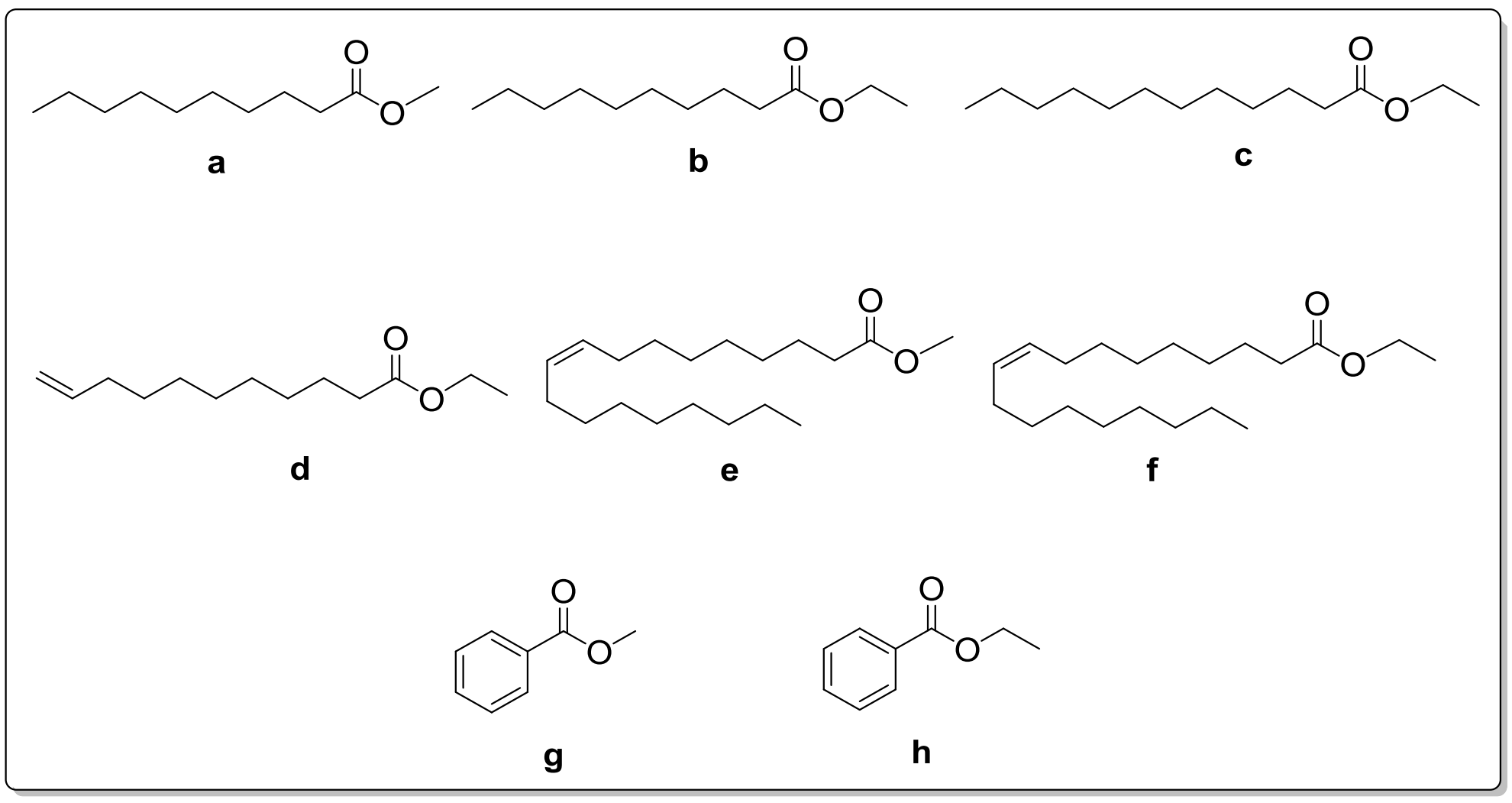

Table S3. Further data regarding the base-free catalytic hydrogenation of 1-decanal with complexes **1-4**, **8/9**, *trans*-[$RuCl_2$(PNN')(PPh_3)] and *trans*-[$RuCl_2$(PNN^G)(PPh_3)], at 27.5 bar of H_2, at 90 °C and in absence of base after 16 h.

**Entry**	**Complex**	**S/C**	**Solvent**	**Conv.[a] [%]**
1	**1**	5000	MeOH	50.4
2	**1**	5000	MeOH	69.9[b]
3	**1**	5000	EtOH	28.7
4	**2**	1000	MeOH	72.2
5	**2**	5000	MeOH	27.9
6	**2**	1000	2-propanol	100
7	**2**	5000	2-propanol	9.9
8	**2**	5000	EtOH	19.5
9	**3**	1000	MeOH	88.4
10	**3**	5000	MeOH	34.3
11	**3**	5000	EtOH	32.0
12	**4**	1000	MeOH	97.2
13	**4**	5000	MeOH	46.2
14	**4**	1000	2-propanol	100
15	**4**	5000	2-propanol	20.7
16	**4**	5000	EtOH	28.9
17	**8/9**	1000	MeOH	95.9
18	**8/9**	5000	MeOH	26.9
19	[$RuCl_2$(PNN')(PPh_3)]	1000	2-propanol	85.0
20	[$RuCl_2$(PNN')(PPh_3)]	1000	EtOH	42.1
21	[$RuCl_2$(PNN^G)(PPh_3)]	1000	2-propanol	51.2

[a] Determined by GC analyses. [b] reaction time of 32 h.

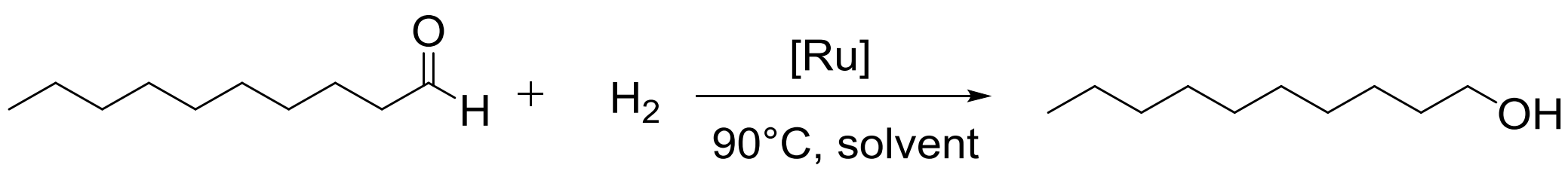

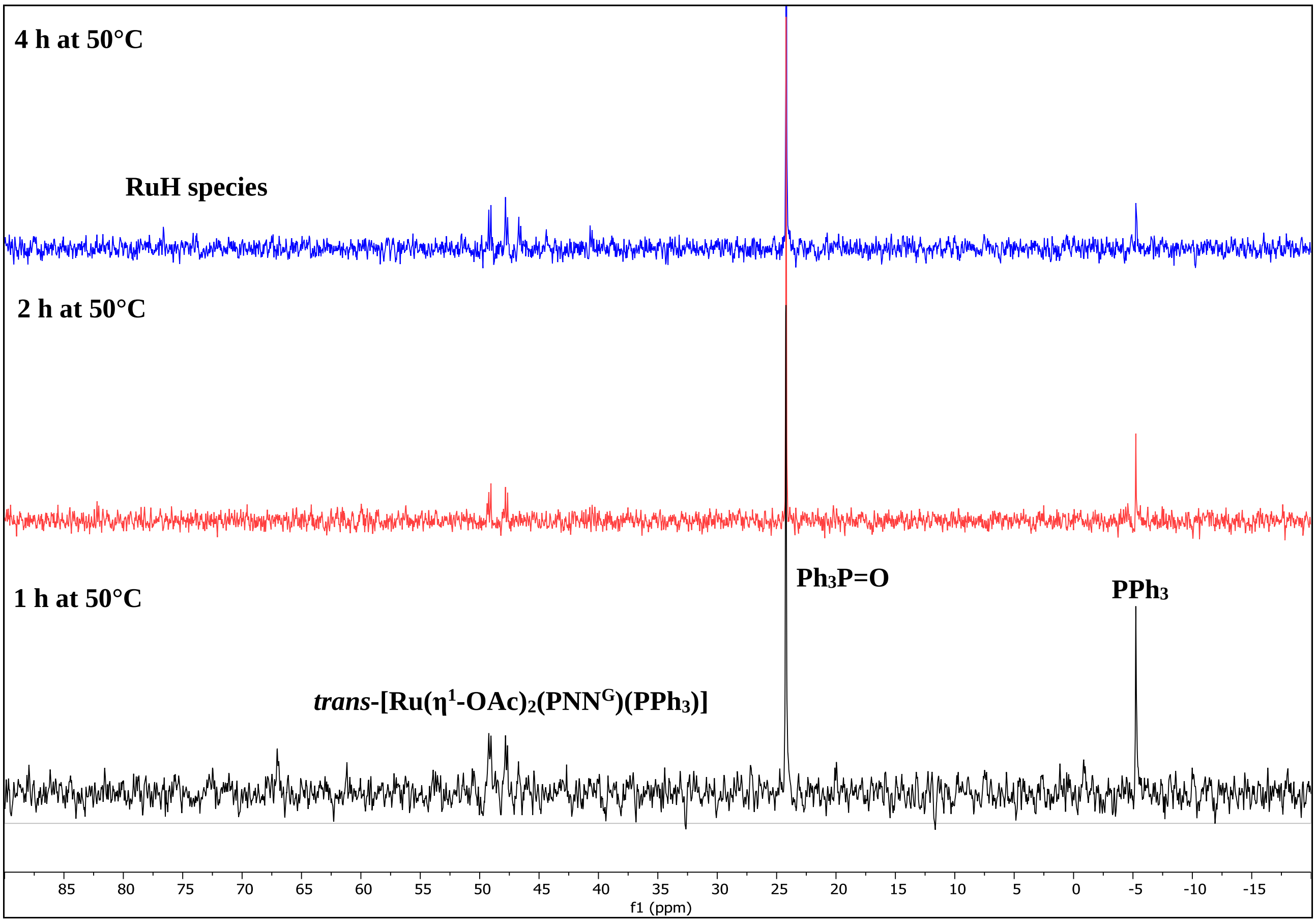

Figure S73. Effect of heating on the formation of ruthenium hydrides by reaction of *trans*-[Ru(η^1-OAc)$_2$(PNN^G)(PPh$_3$)] (**2**) with H_2 (5 bar) and KO^{t}Bu (3 equiv) in the ^{31}P{^{1}H} NMR spectra (162.0 MHz), recorded in toluene-d^8 at 25 °C.

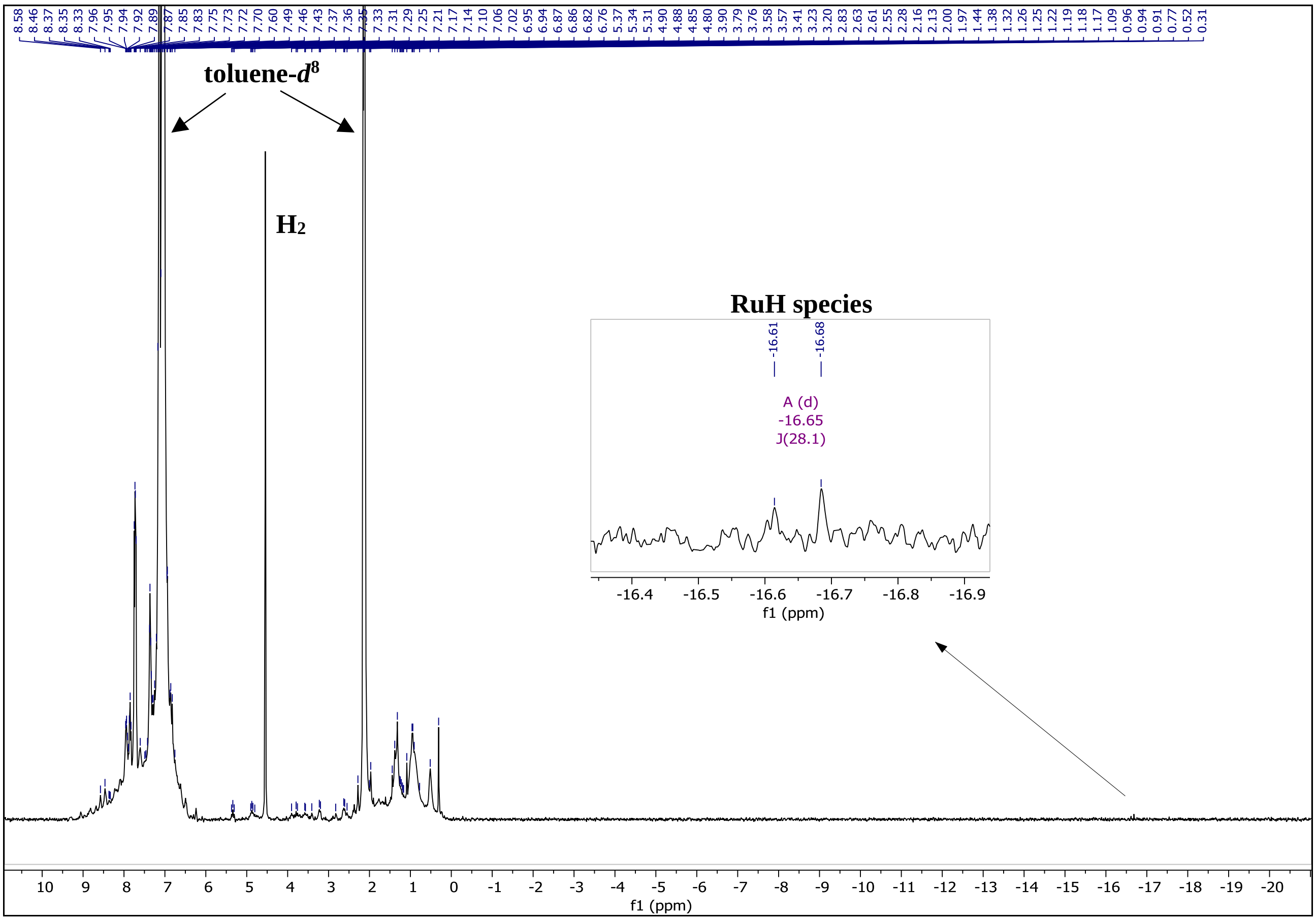

Figure S74. ^{1}H NMR spectrum (400.1 MHz) of the ruthenium hydride obtained by reaction of *trans*-[Ru(η^1-OAc)$_2$(PNN^G)(PPh$_3$)] (**2**) with H_2 (5 bar) and KO^{t}Bu (3 equiv) after heating at 50 °C for 4 h, recorded in toluene-d^8 at 25 °C.

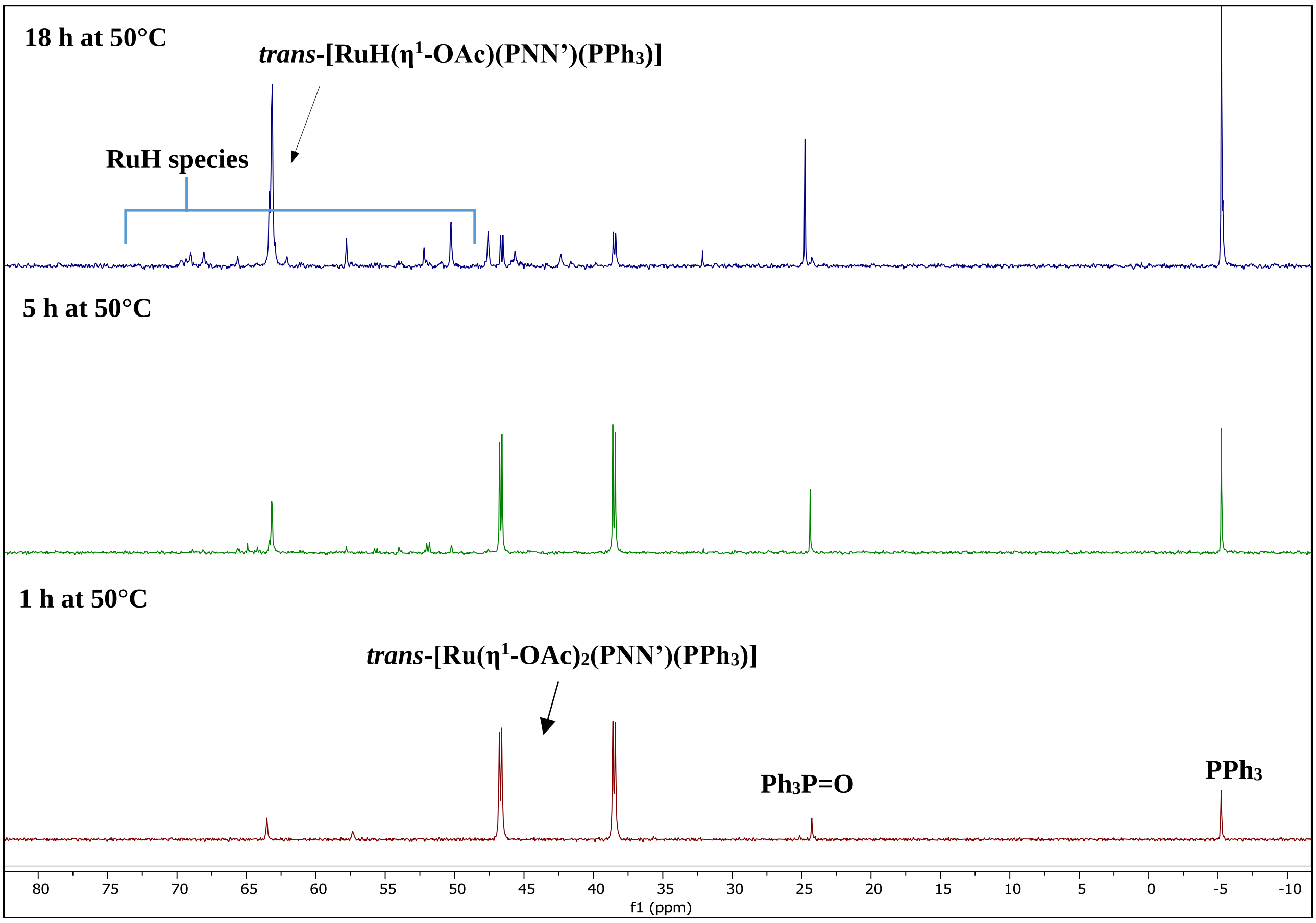

Figure S75. Effect of heating on the formation of ruthenium hydrides by reaction of *trans*-[Ru(η^1-OAc)$_2$(PNN')(PPh$_3$)] (**1**) with H_2 (5 bar) and KO^{t}Bu (3 equiv) in the ^{31}P{^{1}H} NMR spectra (162.0 MHz), recorded in toluene-d^8 at 25 °C.

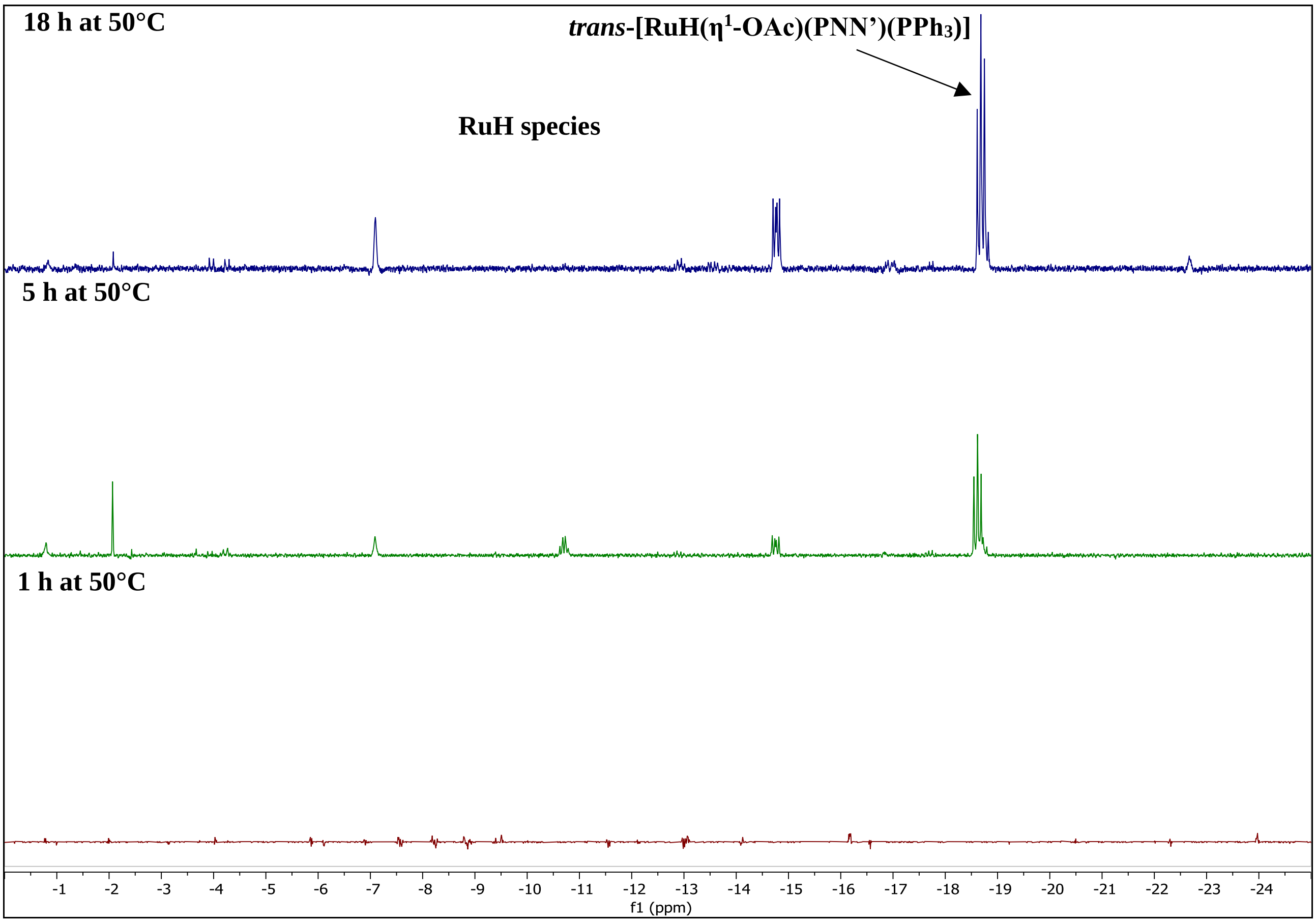

Figure S76. Effect of heating on the formation of ruthenium hydrides by reaction of *trans*-[Ru(η^1-OAc)$_2$(PNN')(PPh_3)] (**1**) with H_2 (5 bar) and KO^{t}Bu (3 equiv) in the hydride region of the ^{1}H NMR spectra (400.1 MHz), recorded in toluene-d^8 at 25 °C.

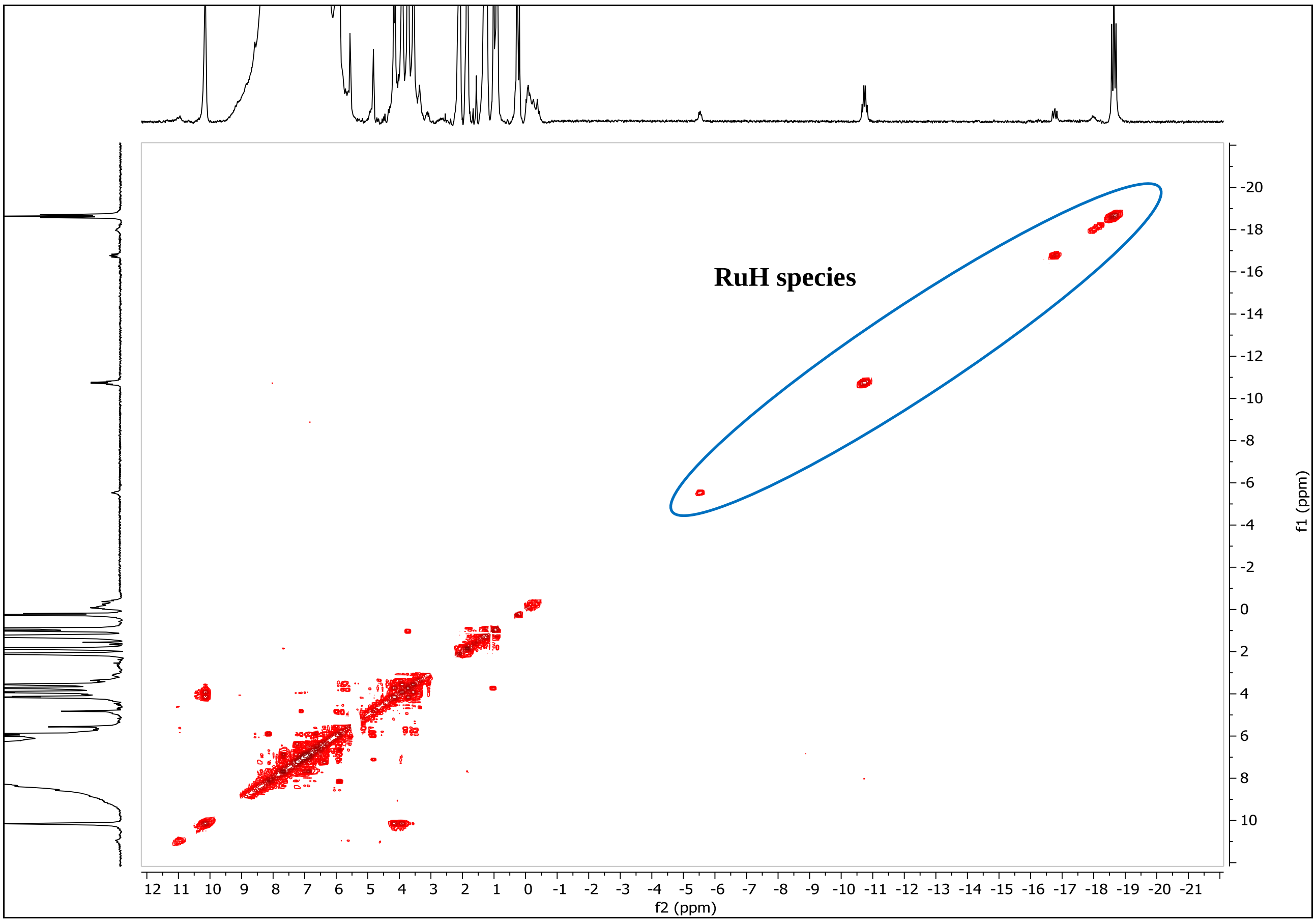

Figure S77. ^{1}H-^{1}H COSY 2D NMR spectrum of the mixture of ruthenium monohydrides obtained by reaction of *trans*-[Ru(η^1-OAc)$_2$(PNN')(PPh$_3$)] (**1**) with H_2 (5 bar) and KO^{t}Bu (3 equiv) after heating at 50 °C for 18 h, recorded in toluene-d^8 at 25 °C (range 12.0/-22.0 ppm).

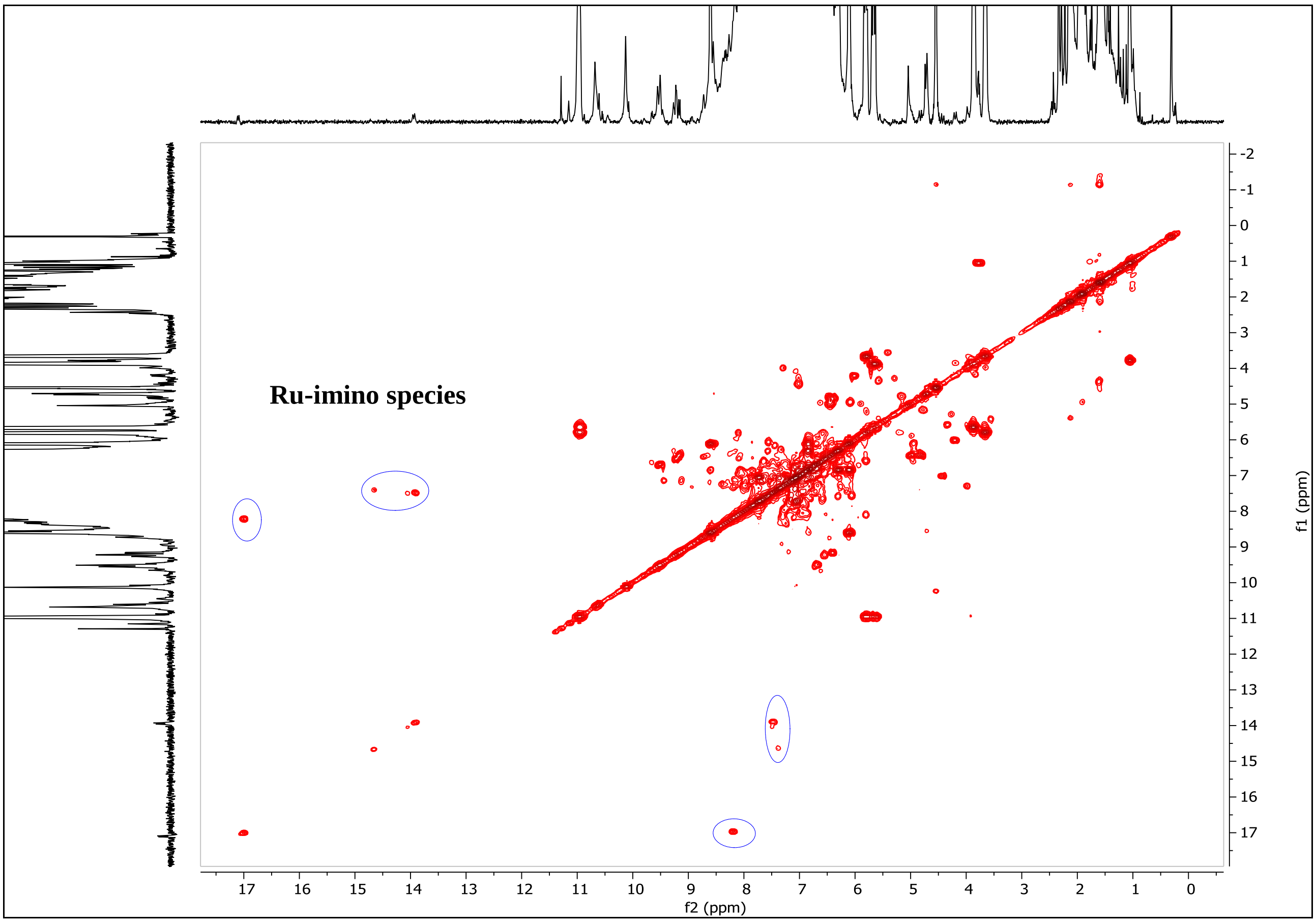

Figure S78. ^{1}H-^{1}H COSY 2D NMR spectrum of the mixture of ruthenium monohydrides obtained by reaction of *trans*-[Ru(η^{1}-OAc)$_2$(PNN')(PPh_3)] (**1**) with H_2 (5 bar) and KO^{t}Bu (3 equiv) after heating at 50 °C for 18 h, recorded in toluene-d^{8} at 25 °C (range 18.0/0.0 ppm).

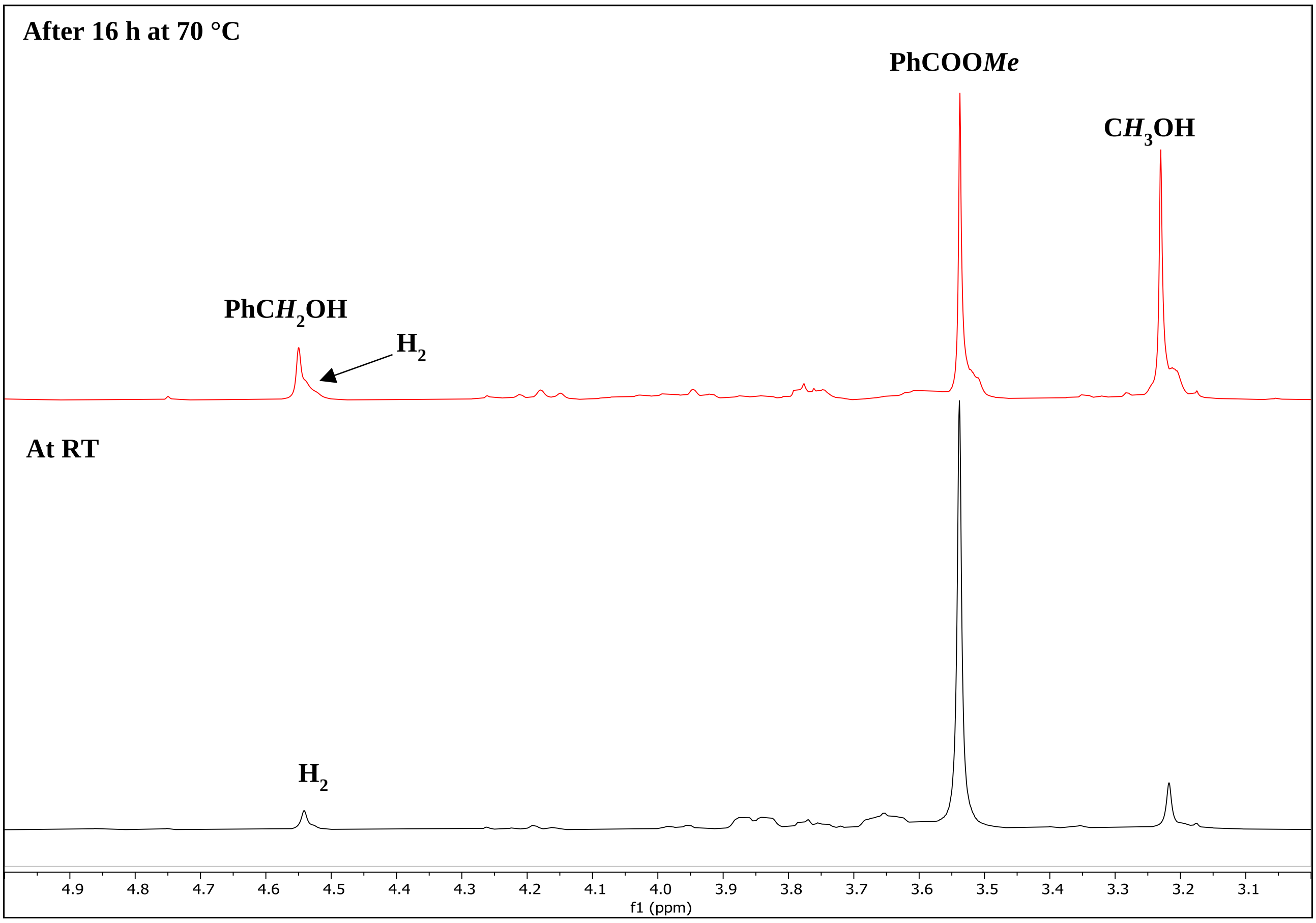

Figure S79. Effect of heating (70 °C for 16 h) on the ^{1}H NMR spectrum (400.1 MHz) of the mixture of ruthenium hydrides (obtained by reaction of *trans*-[Ru(η^1-OAc)$_2$(PNN')(PPh$_3$)] (**1**) with H_2 and KO^{t}Bu) after the addition of PhCOOMe (5 equiv) in H_2 (5 bar) recorded in toluene-*d*^{8} at 25 °C.

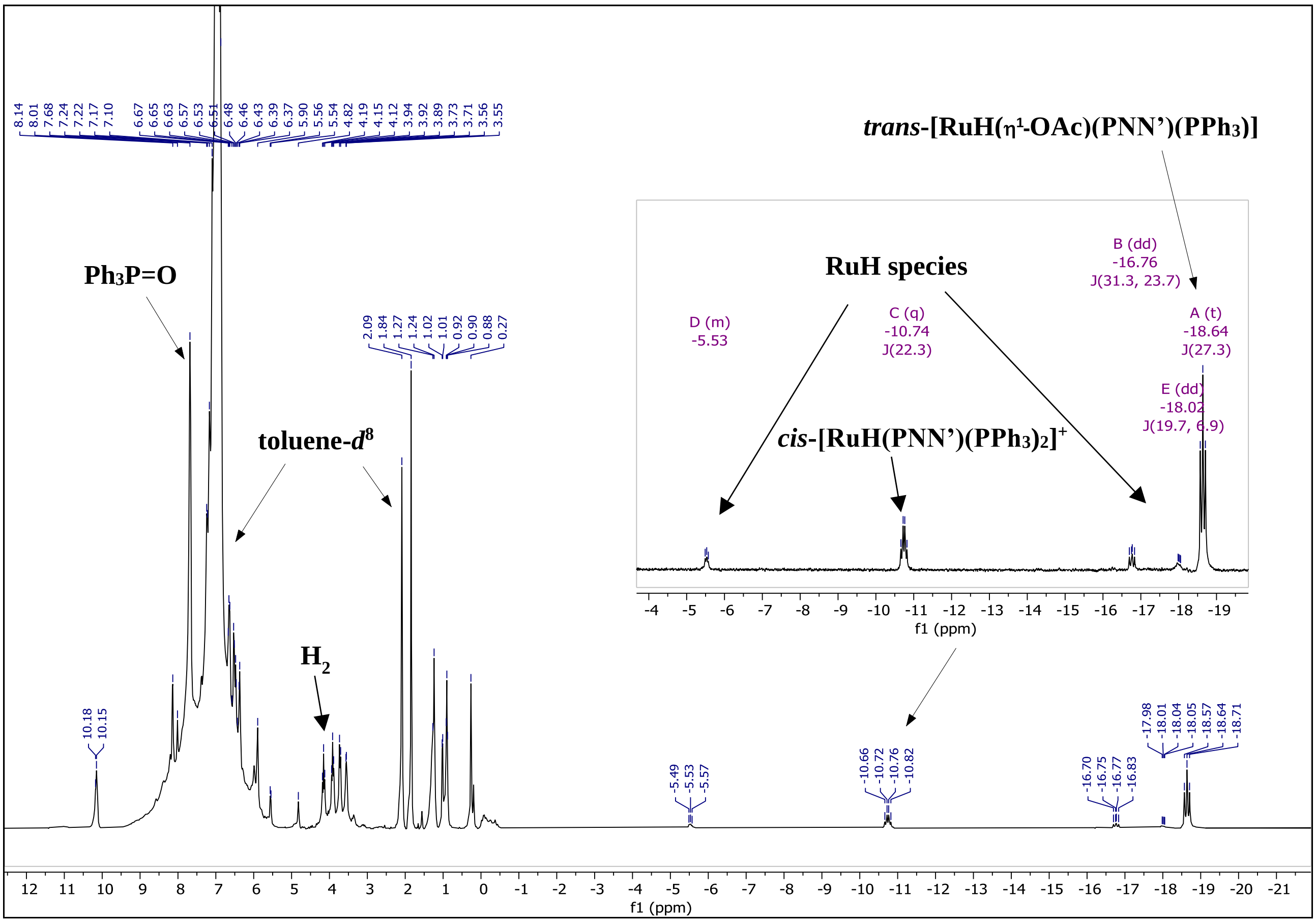

Figure S80. ^{1}H NMR spectrum (400.1 MHz) of the ruthenium hydride mixture obtained by reaction of *trans*-[RuH(η^1-OAc)(PNN')(PPh_3)] (**3**) with H_2 (5 bar) and KO^{t}Bu (3 equiv) after heating at 50 °C for 16 h, recorded in toluene-d^8 at 25 °C.

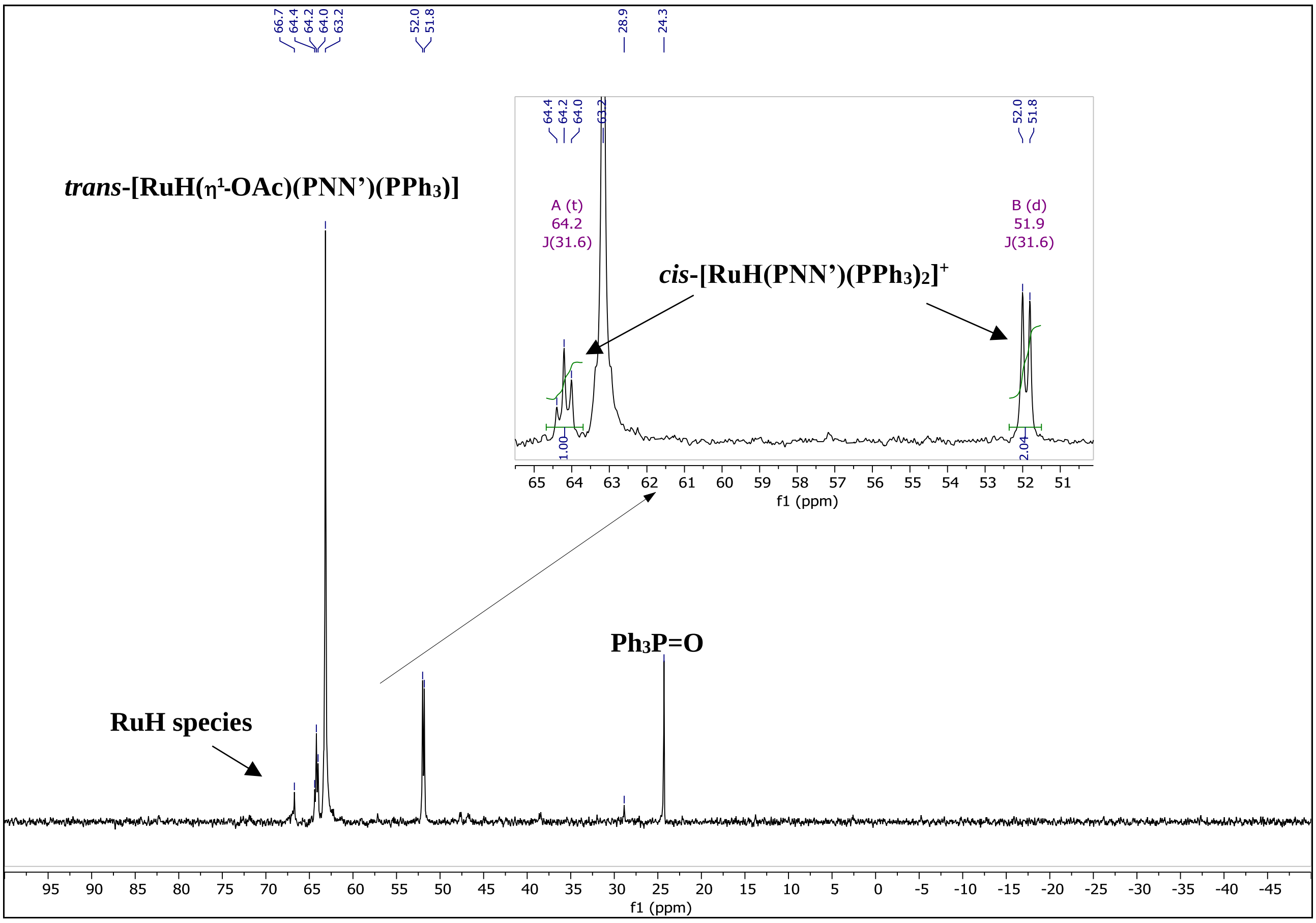

Figure S81. $^{31}P\{^1H\}$ NMR spectrum (162.0 MHz) of the mixture of ruthenium hydrides obtained by reaction of *trans*-[Ru(η^1-OAc)$_2$(PNN')(PPh$_3$)] (**1**) with H_2 (5 bar) and KO^{t}Bu (3 equiv) after heating at 50 °C for 16 h, recorded in toluene-d^8 at 25 °C.

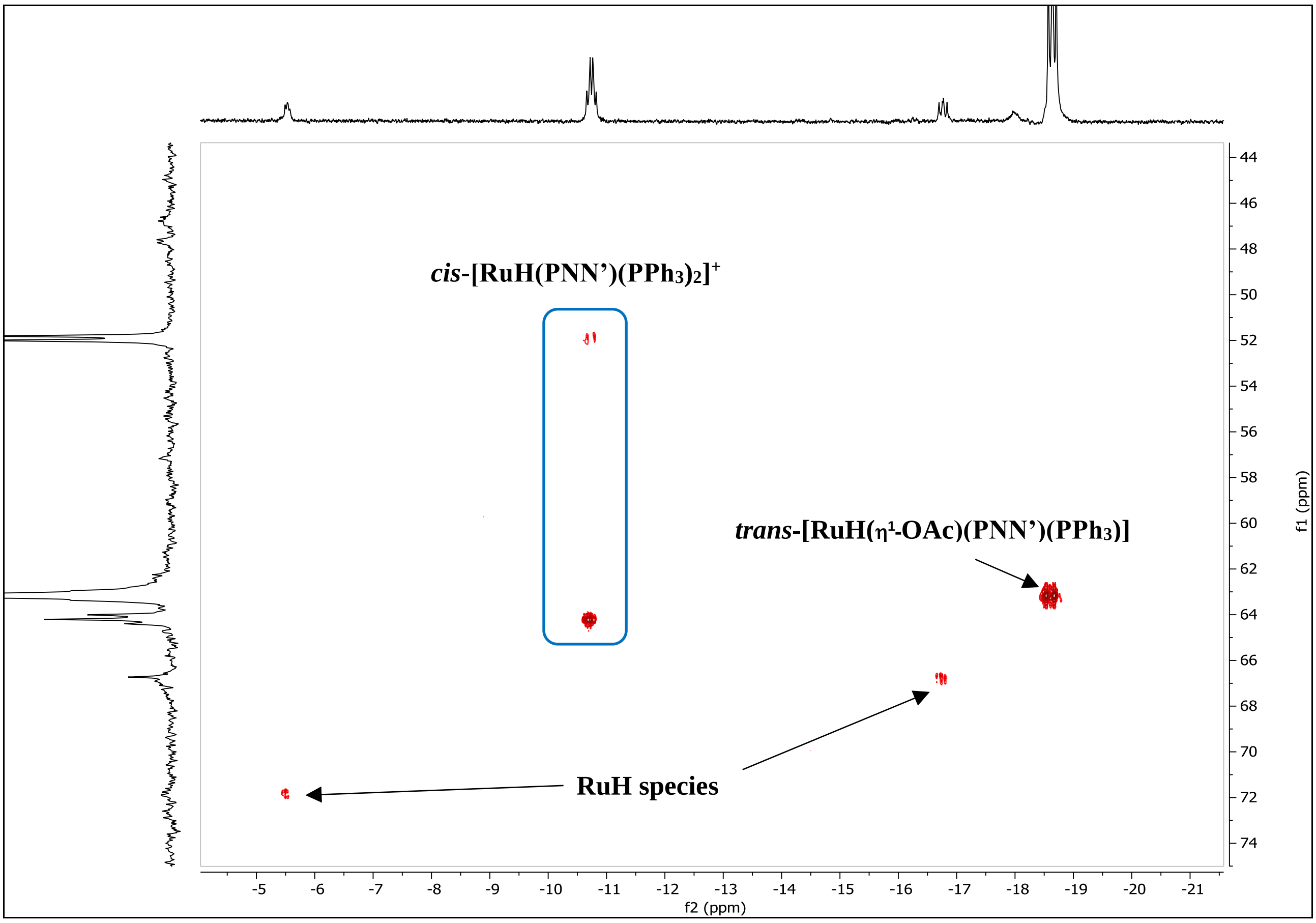

Figure S82. ^{1}H-^{31}P HMBC 2D NMR spectrum of the mixture of ruthenium hydrides obtained by reaction of *trans*-[Ru(η^1-OAc)$_2$(PNN')(PPh$_3$)] (**1**) with H_2 (5 bar) and KO^{t}Bu (3 equiv) after heating at 50 °C for 16 h, recorded in toluene-d^8 at 25 °C.

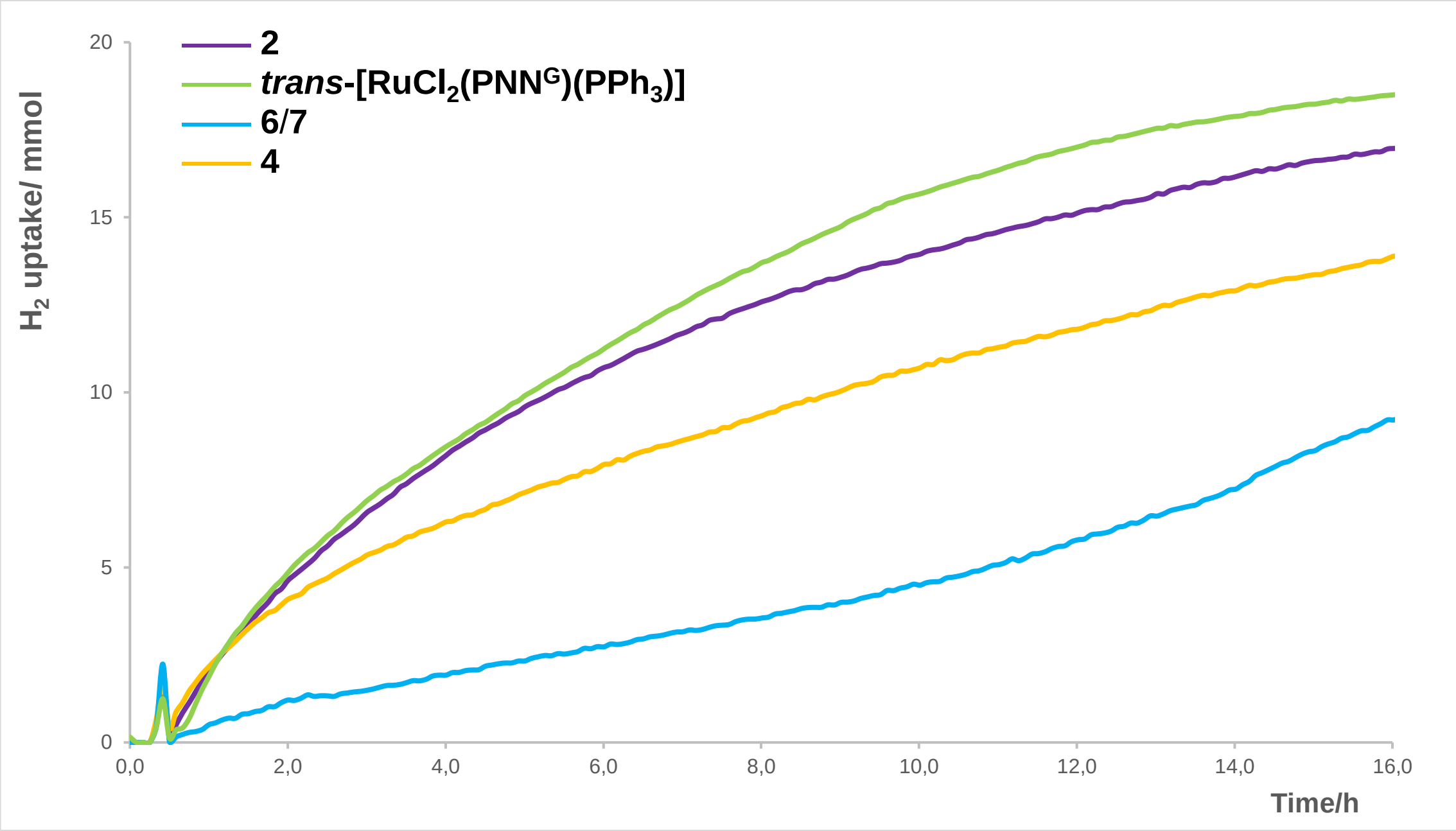

Figure S83. Hydrogen uptake curve of complexes **2**,**4** and **6**/**7** and the *trans*-[$RuCl_2(PNN^G)(PPh_3)$] derivative for the solvent-free reduction of methyl decanoate **a** at 90 °C, 27.5 bar of H_2, 50 mol% NaOMe (S/C 10000).

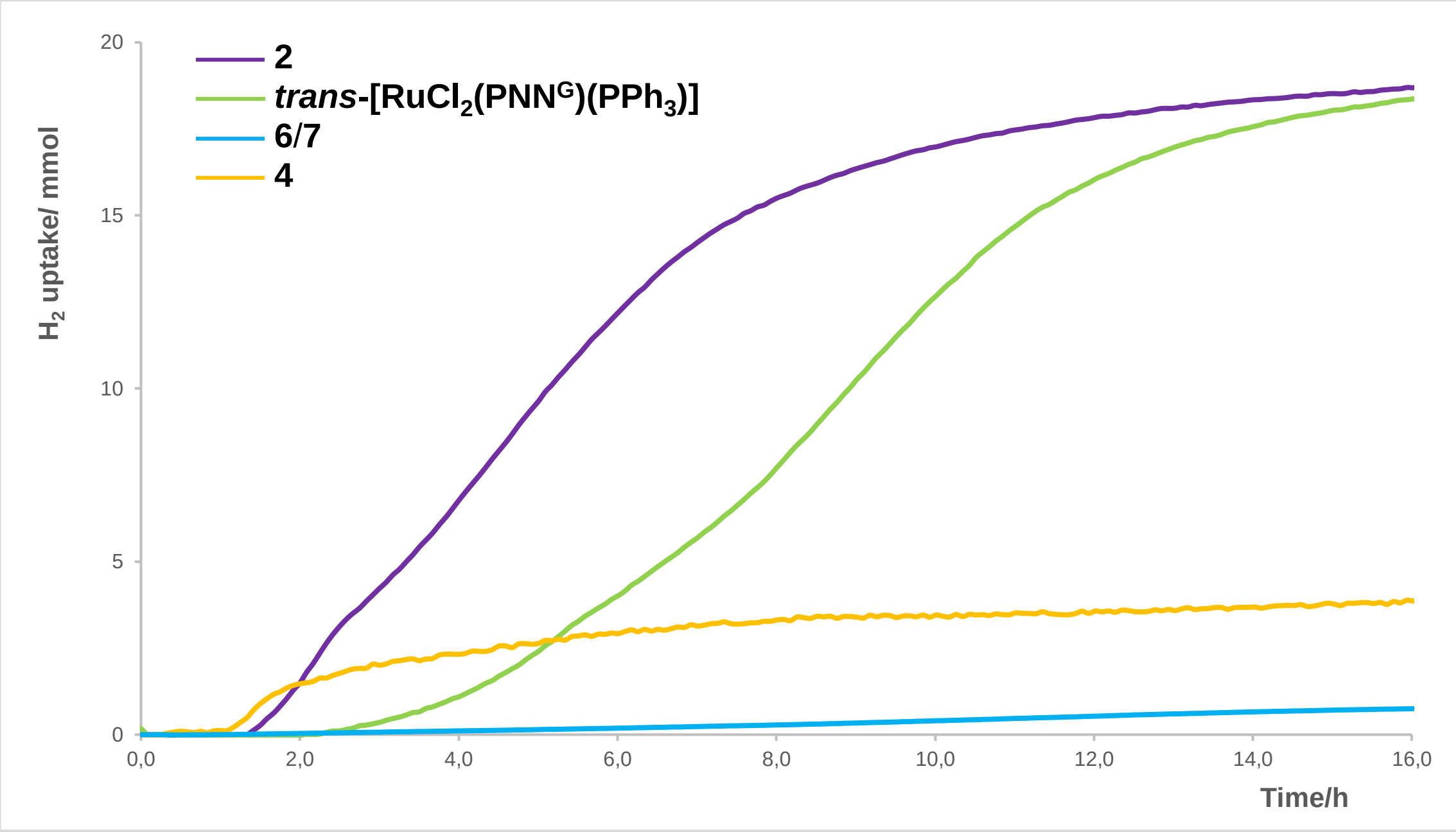

Figure S84. Hydrogen uptake curve of complexes **2**,**4** and **6**/**7** and the *trans*-[$RuCl_2(PNN^G)(PPh_3)$] derivative for the solvent-free reduction of methyl decanoate **a** at 40 °C, 27.5 bar of H_2, 50 mol% NaOMe (S/C 50000).

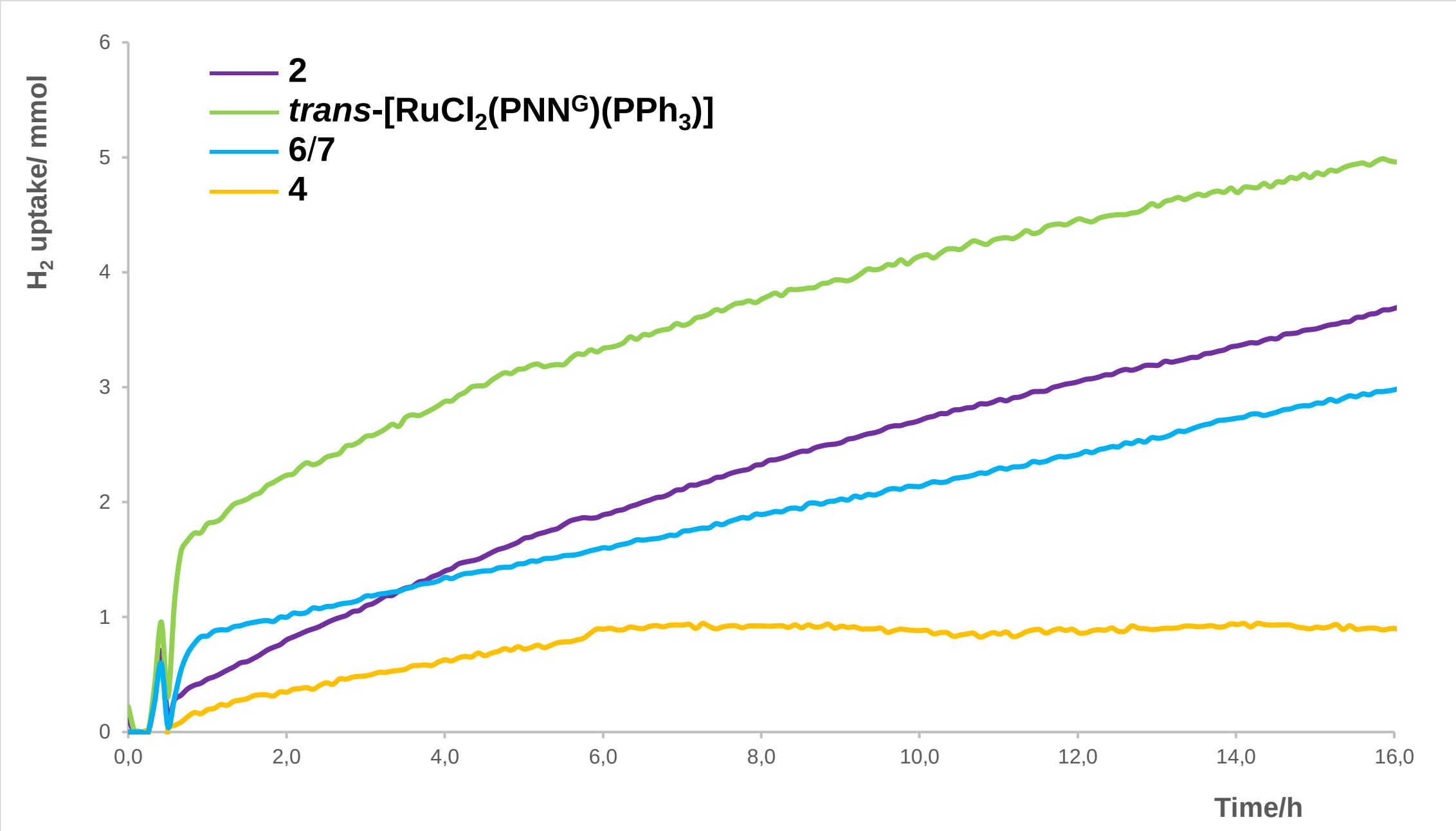

Figure S85. Hydrogen uptake curve of complexes **2**,**4** and **6**/**7** and the *trans*-[RuCl$_2$(PNN^G)(PPh$_3$)] derivative for the solvent-free reduction of methyl decanoate **a** at 90 °C, 27.5 bar of H_2, 50 mol% NaOMe (S/C 50000).

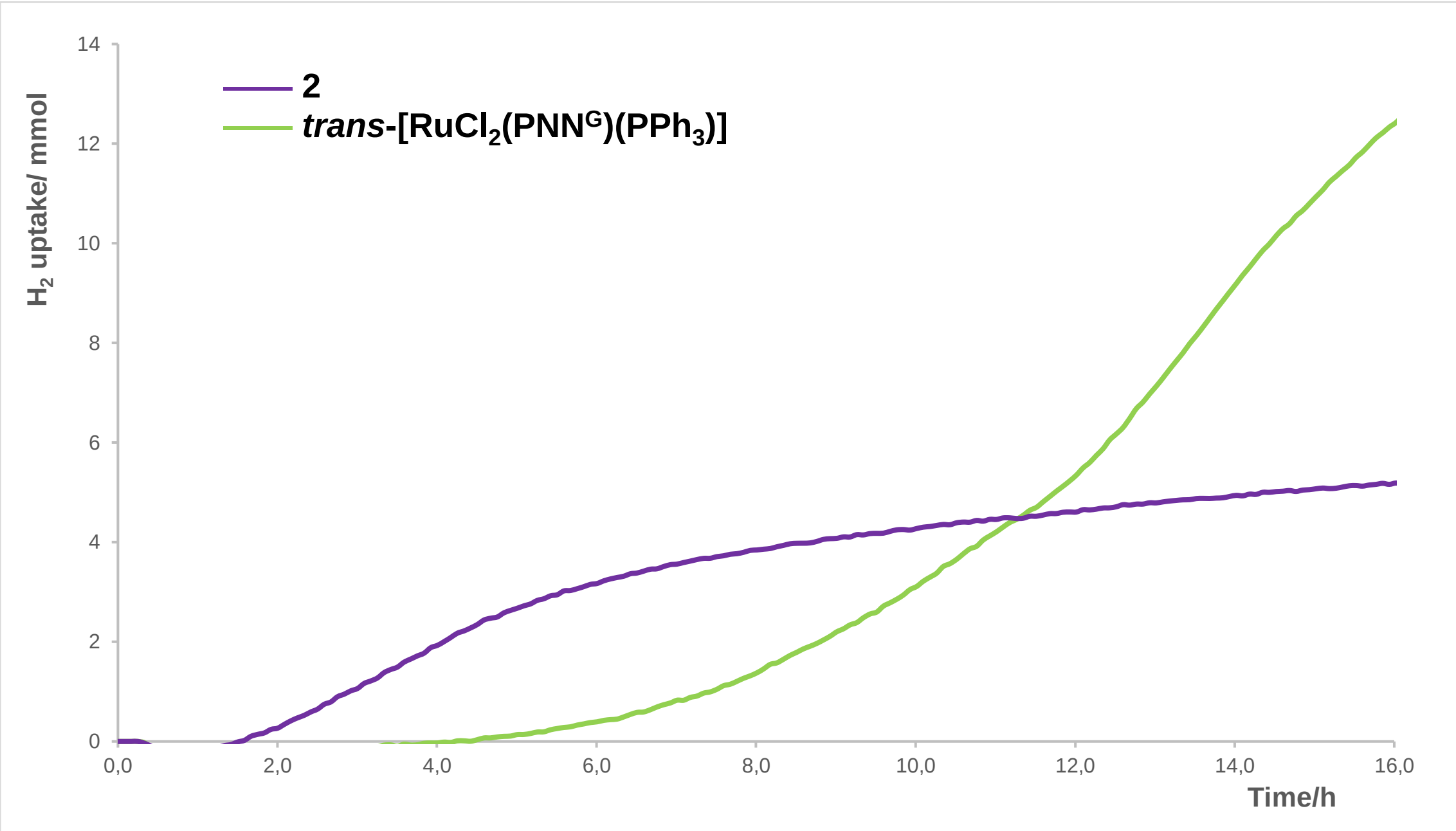

Figure S86. Hydrogen uptake curve of complex **2** and the *trans*-[RuCl$_2$(PNN^G)(PPh$_3$)] derivative for the reduction of methyl decanoate **a** at 40 °C, 27.5 bar of H_2, 50 mol% NaOMe (S/C 100000).

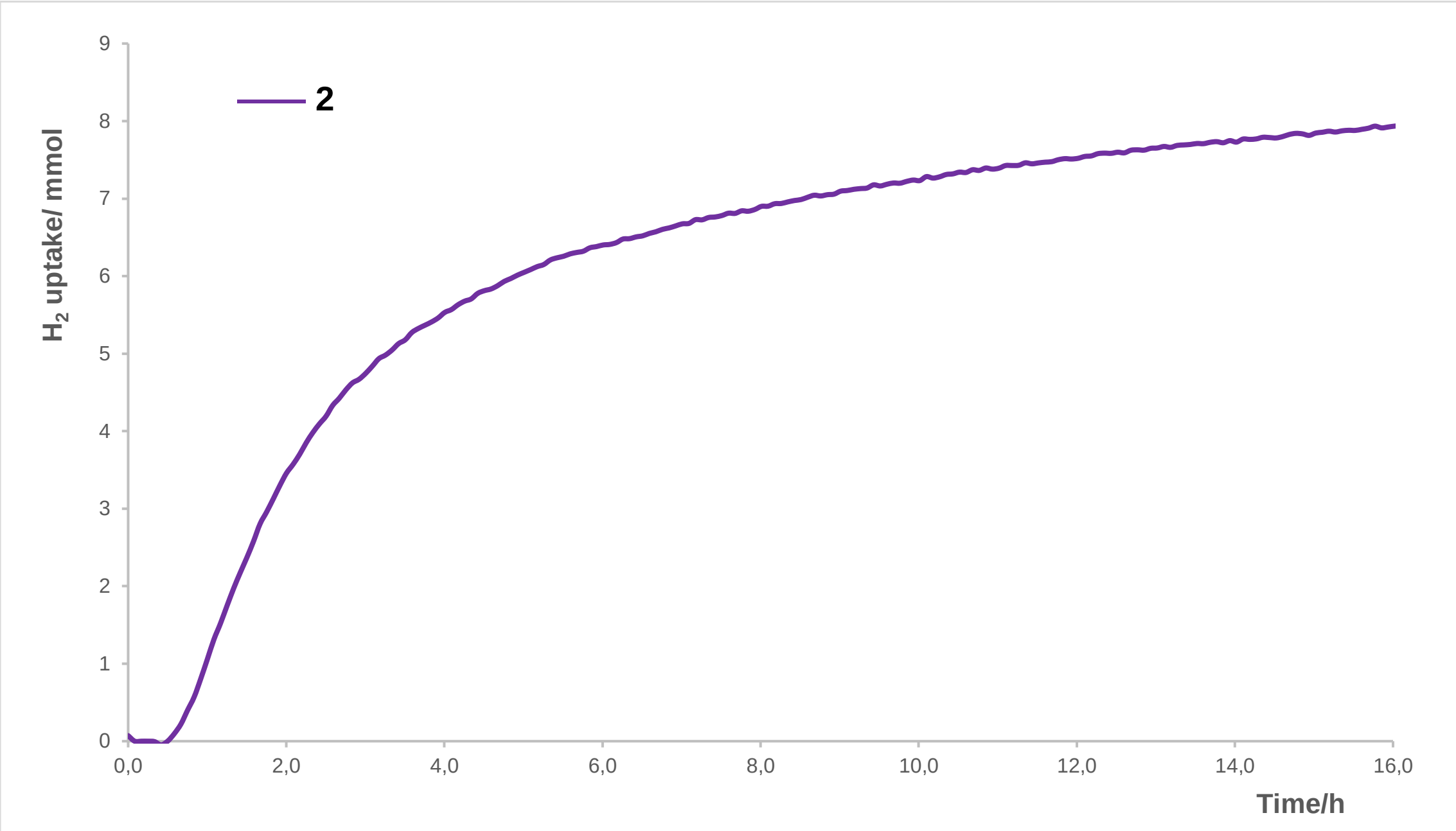

Figure S87. Hydrogen uptake curve of complex **2** for the reduction of methyl decanoate **a** at 40 °C, 27.5 bar of H_2, 50 mol% NaOMe (S/C 50000) using toluene as solvent.

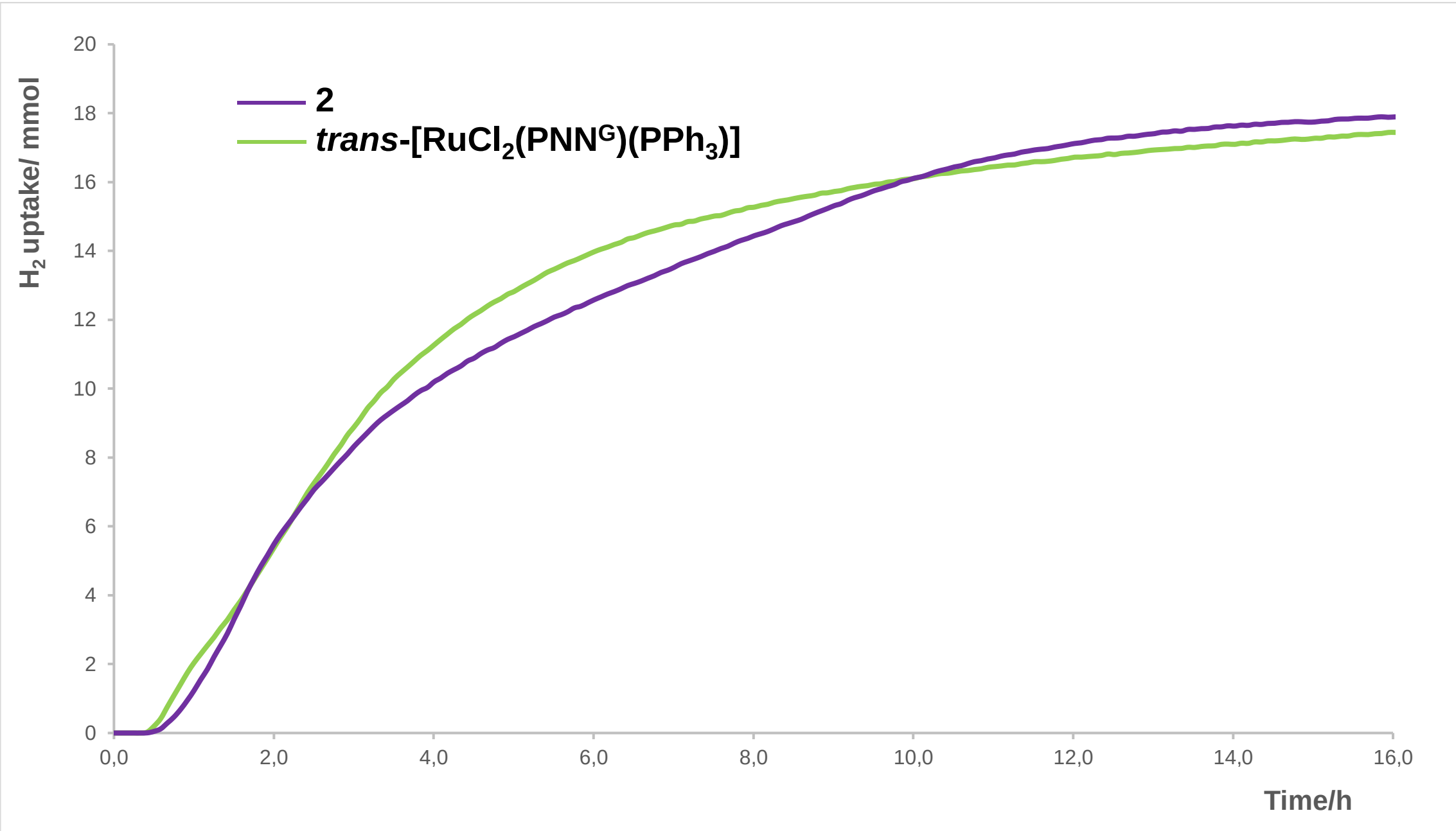

Figure S88. Hydrogen uptake curve of complex **2** and the *trans*-[$RuCl_2(PNN^G)(PPh_3)$] derivative for the solvent-free reduction of ethyl decanoate **b** at 40 °C, 27.5 bar of H_2, 50 mol% NaOEt (S/C 100000).

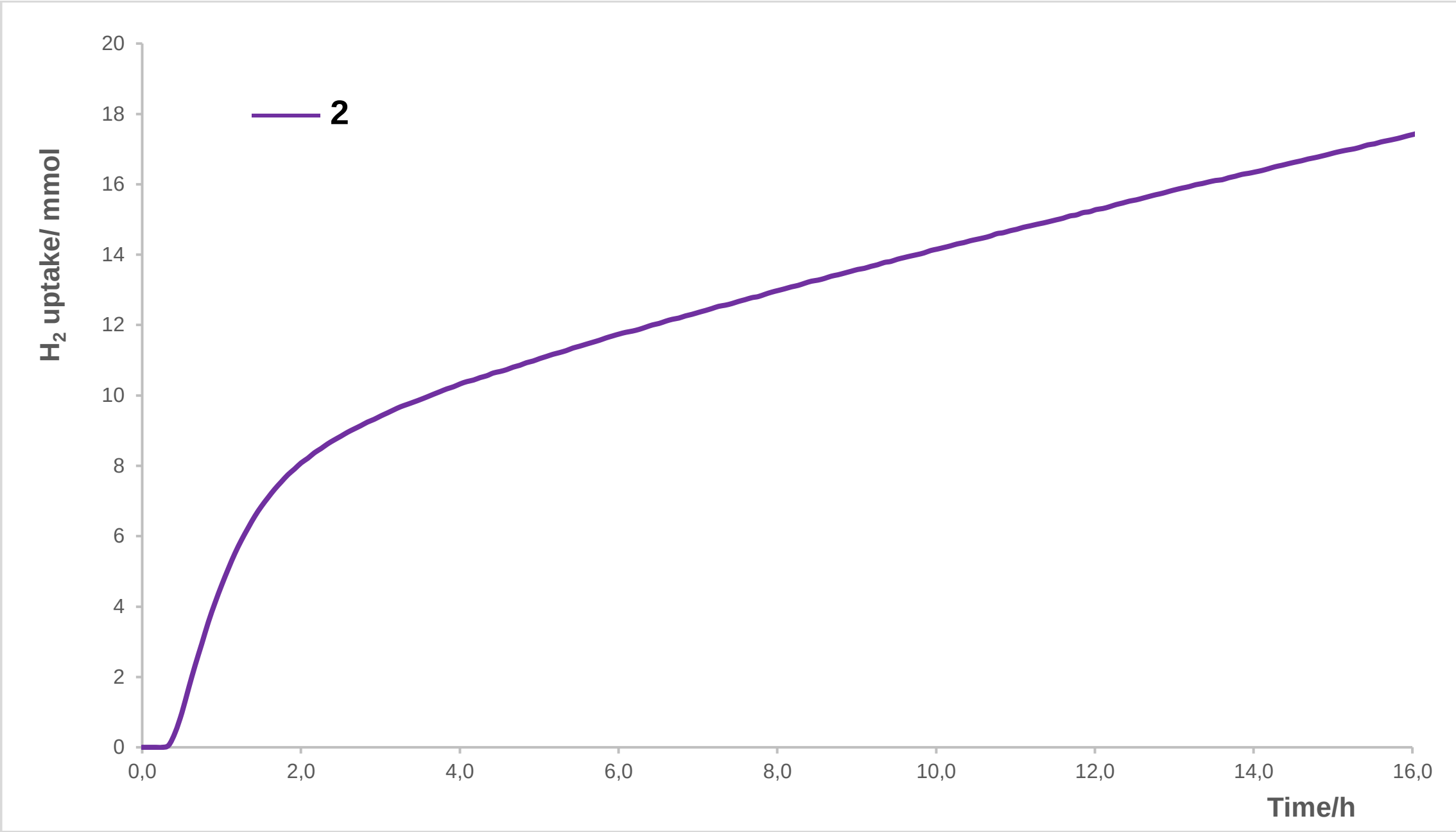

Figure S89. Hydrogen uptake curve of complex **2** for the solvent-free reduction of ethyl dodecanoate **c** at 40 °C, 27.5 bar of H_2, 50 mol% NaOEt (S/C 44000).

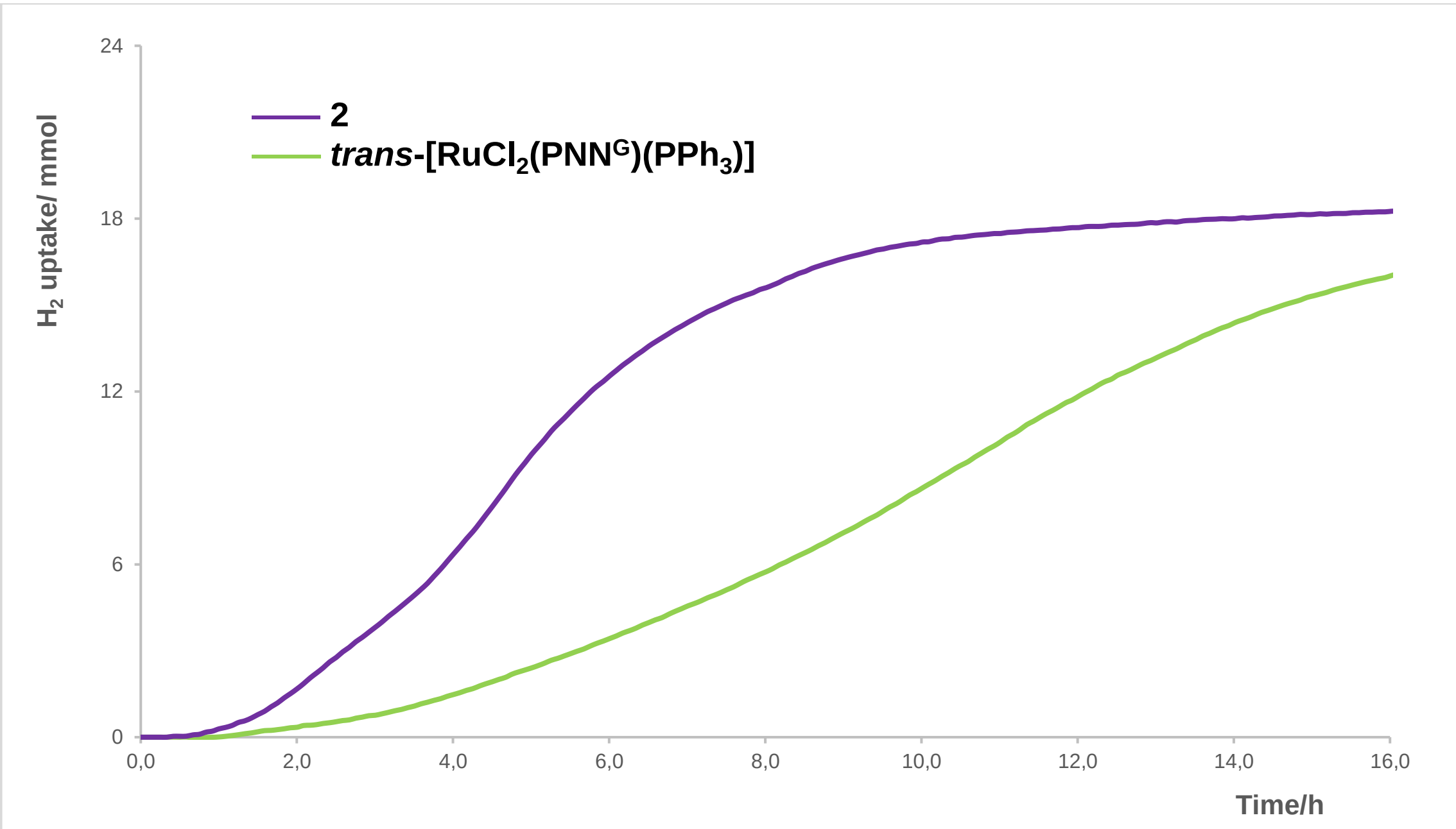

Figure S90. Hydrogen uptake curve of complex **2** and the *trans*-[$RuCl_2$(PNN^G)(PPh_3)] derivative for the solvent-free reduction of ethyl 10-undecenoate **d** at 30 °C, 27.5 bar of H_2, 50 mol% NaOEt (S/C 10000).

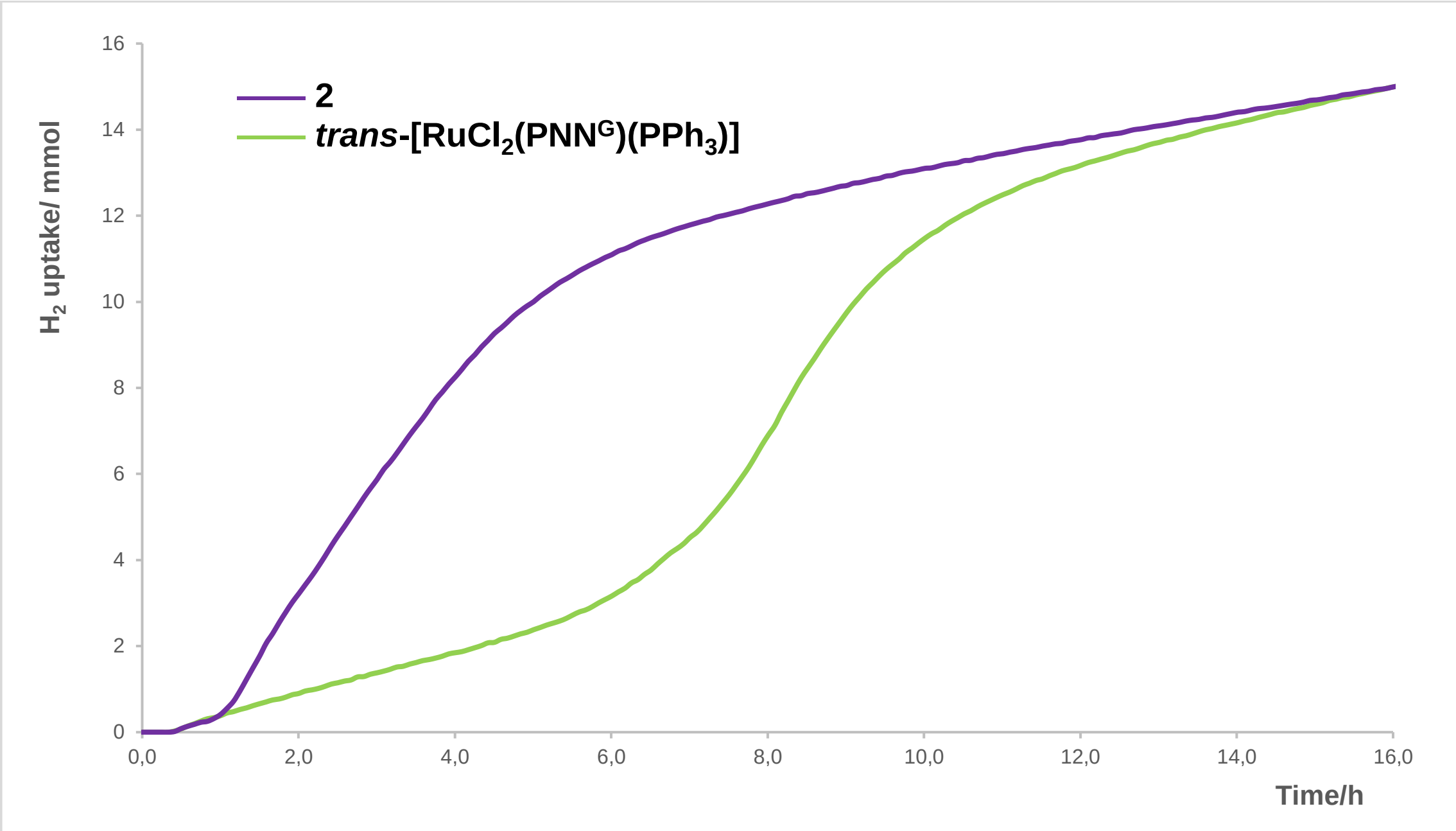

Figure S91. Hydrogen uptake curve of complex **2** and the *trans*-[RuCl$_2$(PNN^G)(PPh$_3$)] derivative for the solvent-free reduction of methyl oleate **e** at 60 °C, 27.5 bar of H_2, 50 mol% NaOMe (S/C 25000).

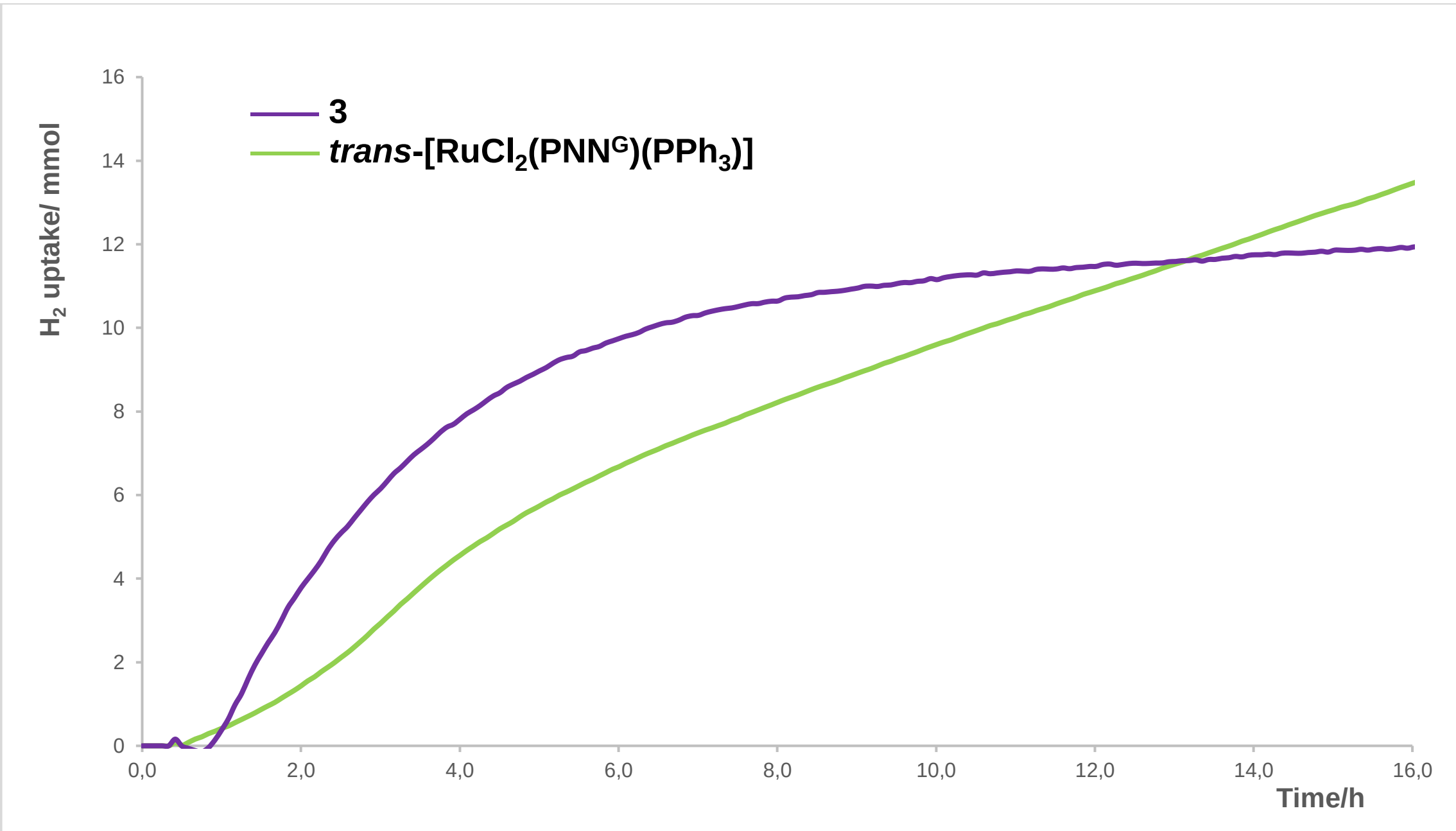

Figure S92. Hydrogen uptake curve of complex **2** and the *trans*-[RuCl$_2$(PNN^G)(PPh$_3$)] derivative for the solvent-free reduction of methyl oleate **e** at 50 °C, 27.5 bar of H_2, 50 mol% NaOMe (S/C 50000).

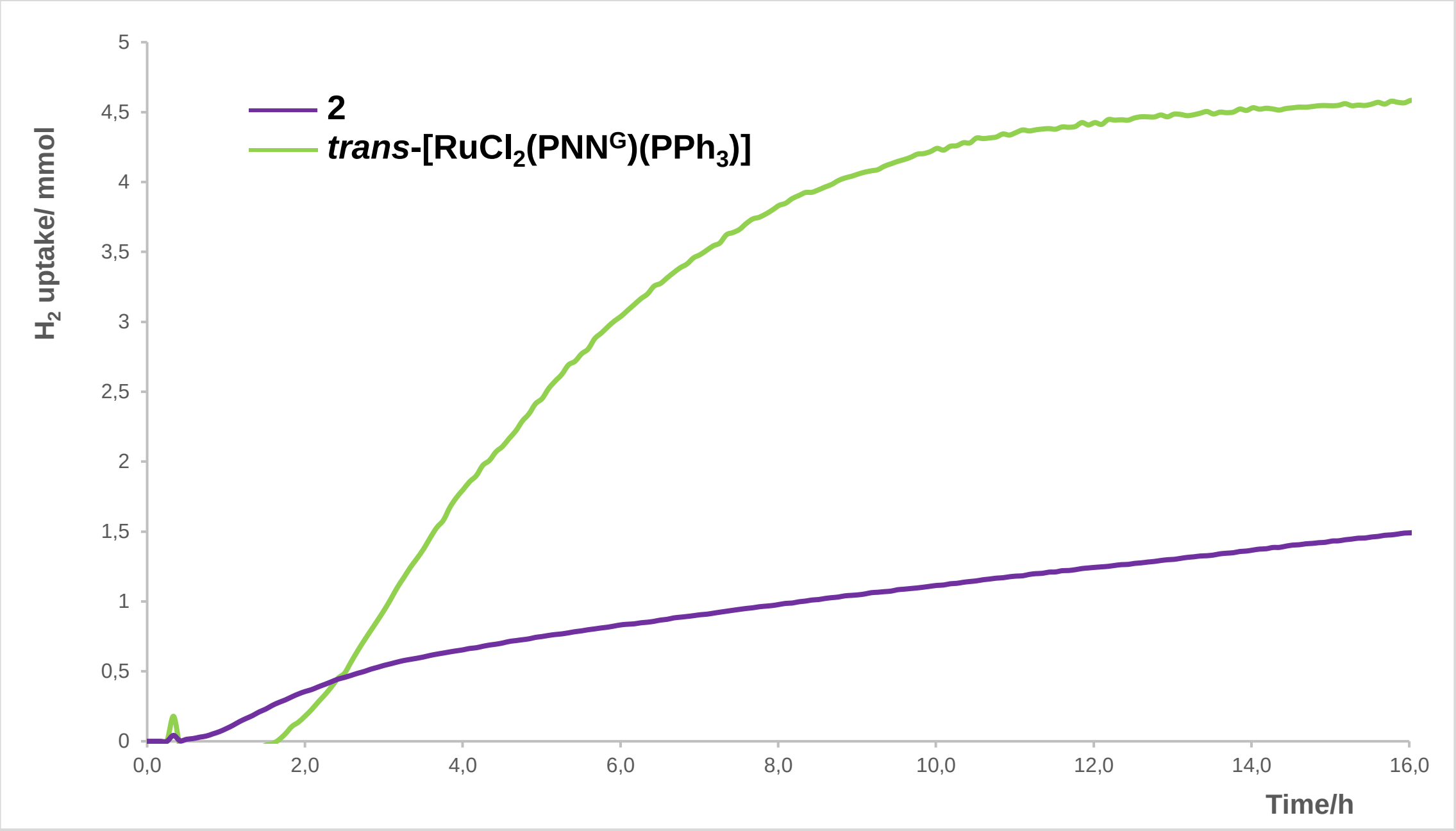

Figure S93. Hydrogen uptake curve of complex **2** and the *trans*-[$RuCl_2(PNN^G)(PPh_3)$] derivative for the solvent-free reduction of methyl oleate **e** at 50 °C, 27.5 bar of H_2, 50 mol% NaOMe (S/C 100000).

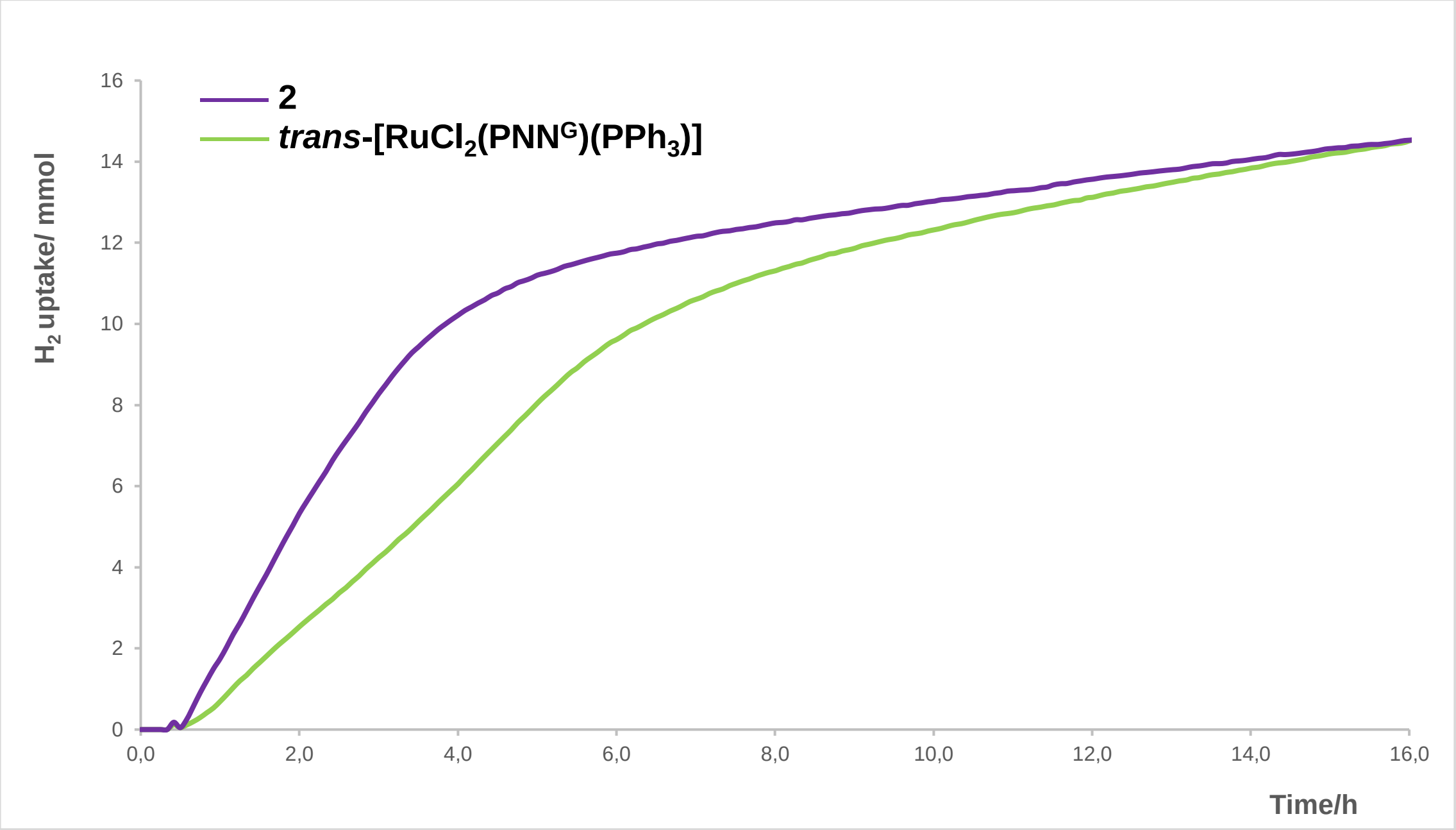

Figure S94. Hydrogen uptake curve of complex **2** and the *trans*-[$RuCl_2(PNN^G)(PPh_3)$] derivative for the solvent-free reduction of ethyl oleate **f** at 40 °C, 27.5 bar of H_2, 50 mol% NaOEt (S/C 50000).

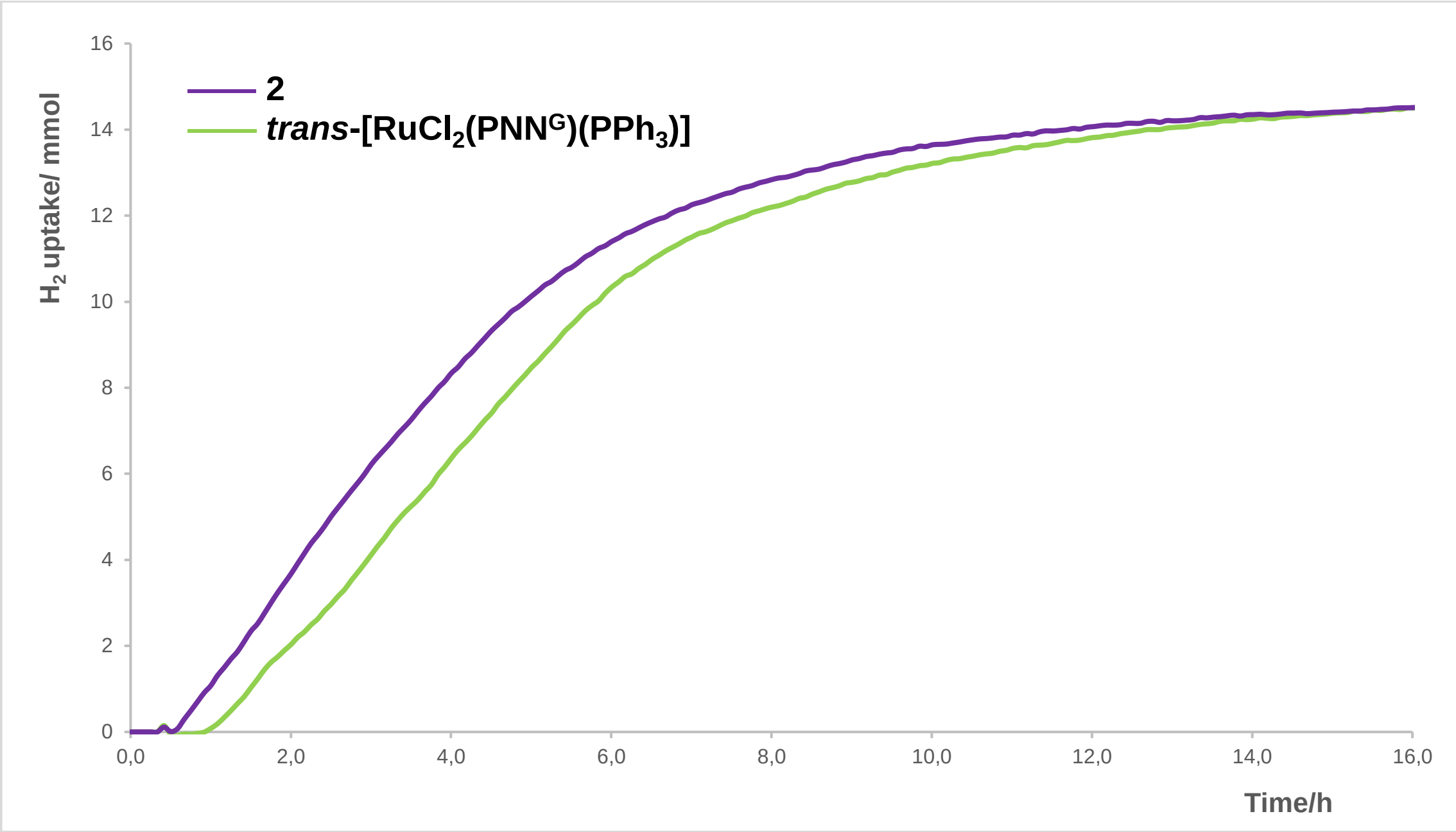

Figure S95. Hydrogen uptake curve of complex **2** and the *trans*-[RuCl$_2$(PNN^G)(PPh$_3$)] derivative for the solvent-free reduction of ethyl oleate **f** at 40 °C, 27.5 bar of H_2, 50 mol% NaOEt (S/C 100000).

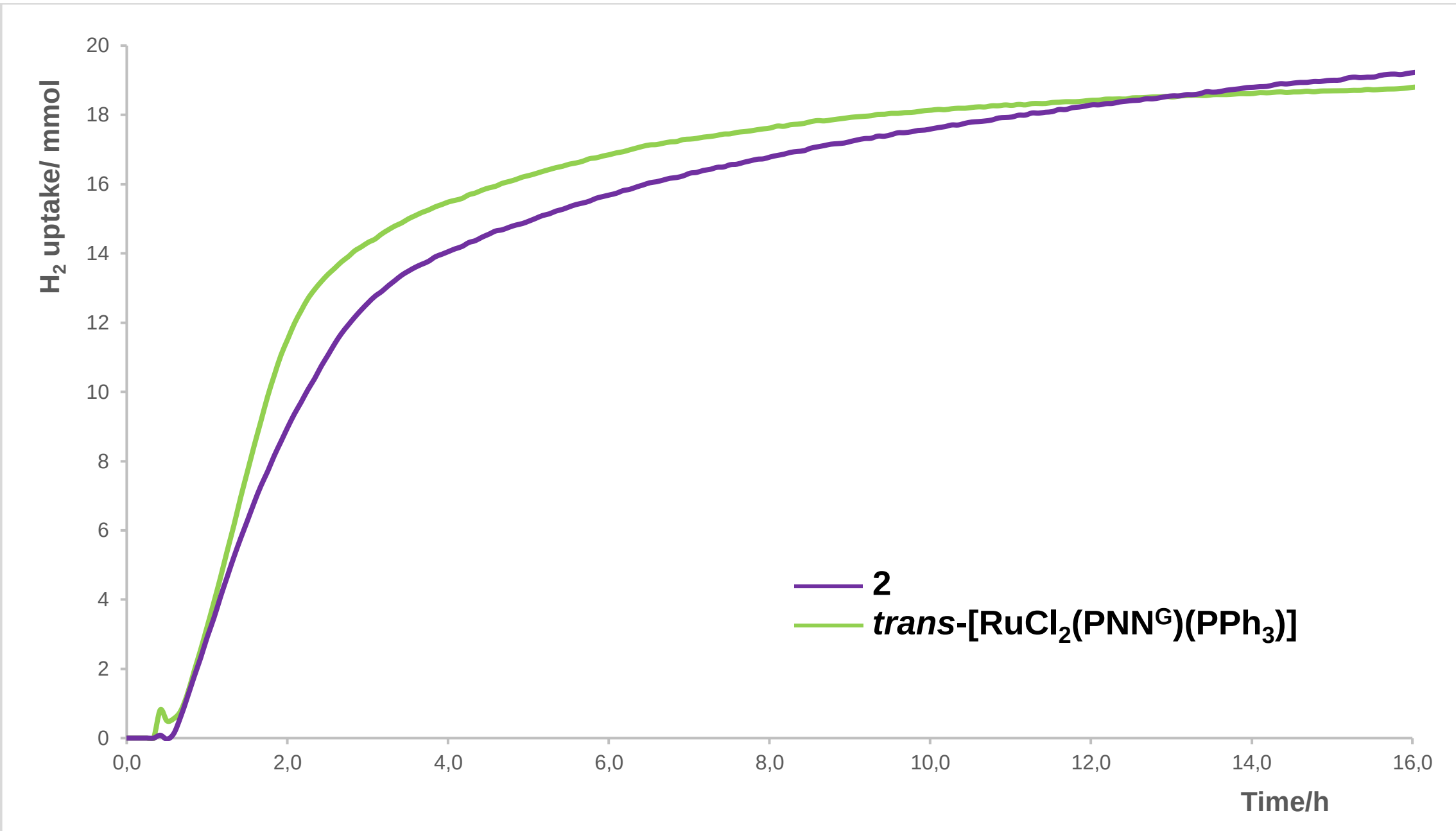

Figure S96. Hydrogen uptake curve of complex **2** and the *trans*-[RuCl$_2$(PNN^G)(PPh$_3$)] derivative for the solvent-free reduction of methyl benzoate **g** at 40 °C, 27.5 bar of H_2, 50 mol% NaOEt (S/C 10000).

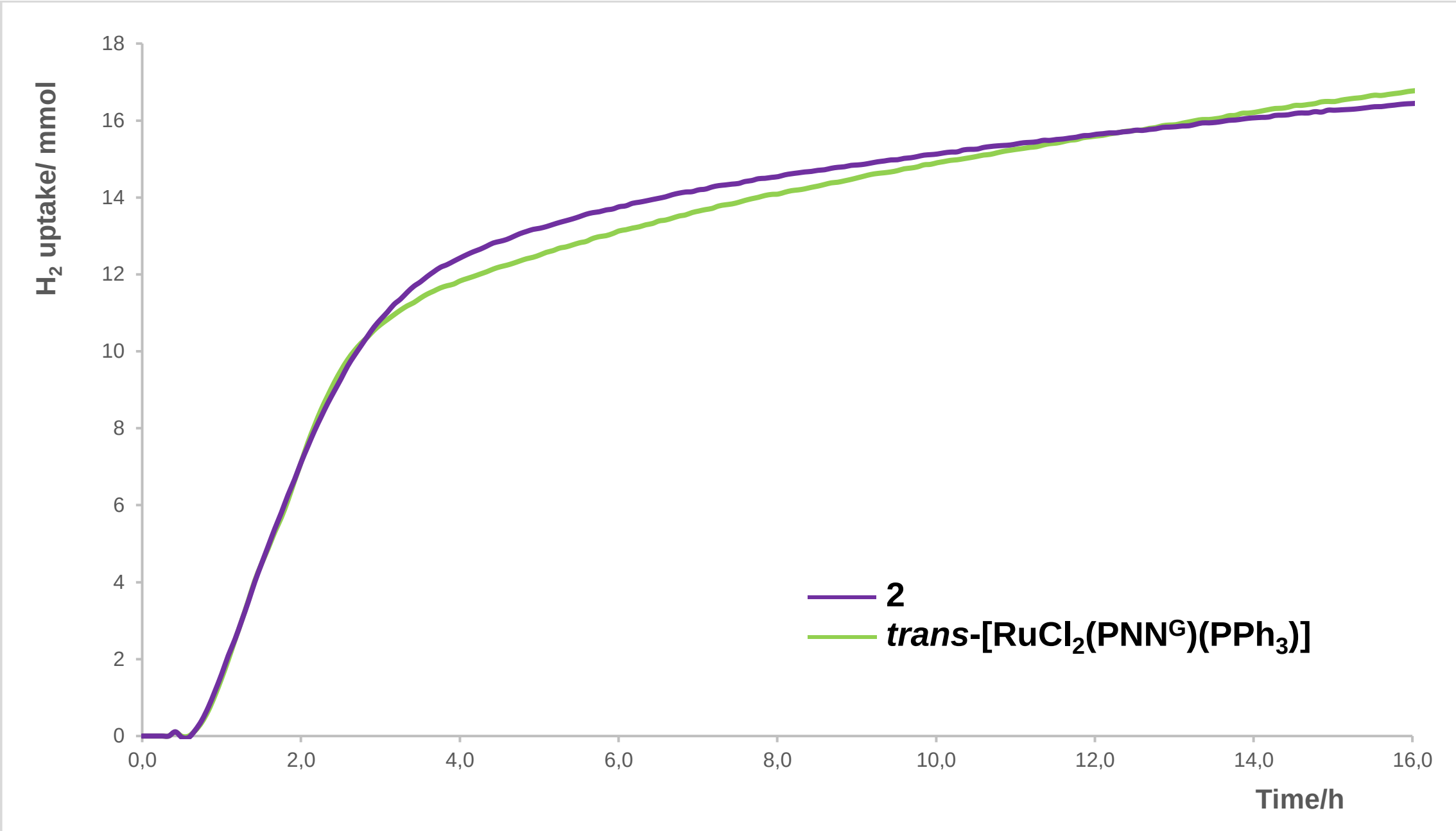

Figure S97. Hydrogen uptake curve of complex **2** and the *trans*-[RuCl$_2$(PNN^G)(PPh$_3$)] derivative for the solvent-free reduction of methyl benzoate **g** at 40 °C, 27.5 bar of H_2, 50 mol% NaOEt (S/C 25000).

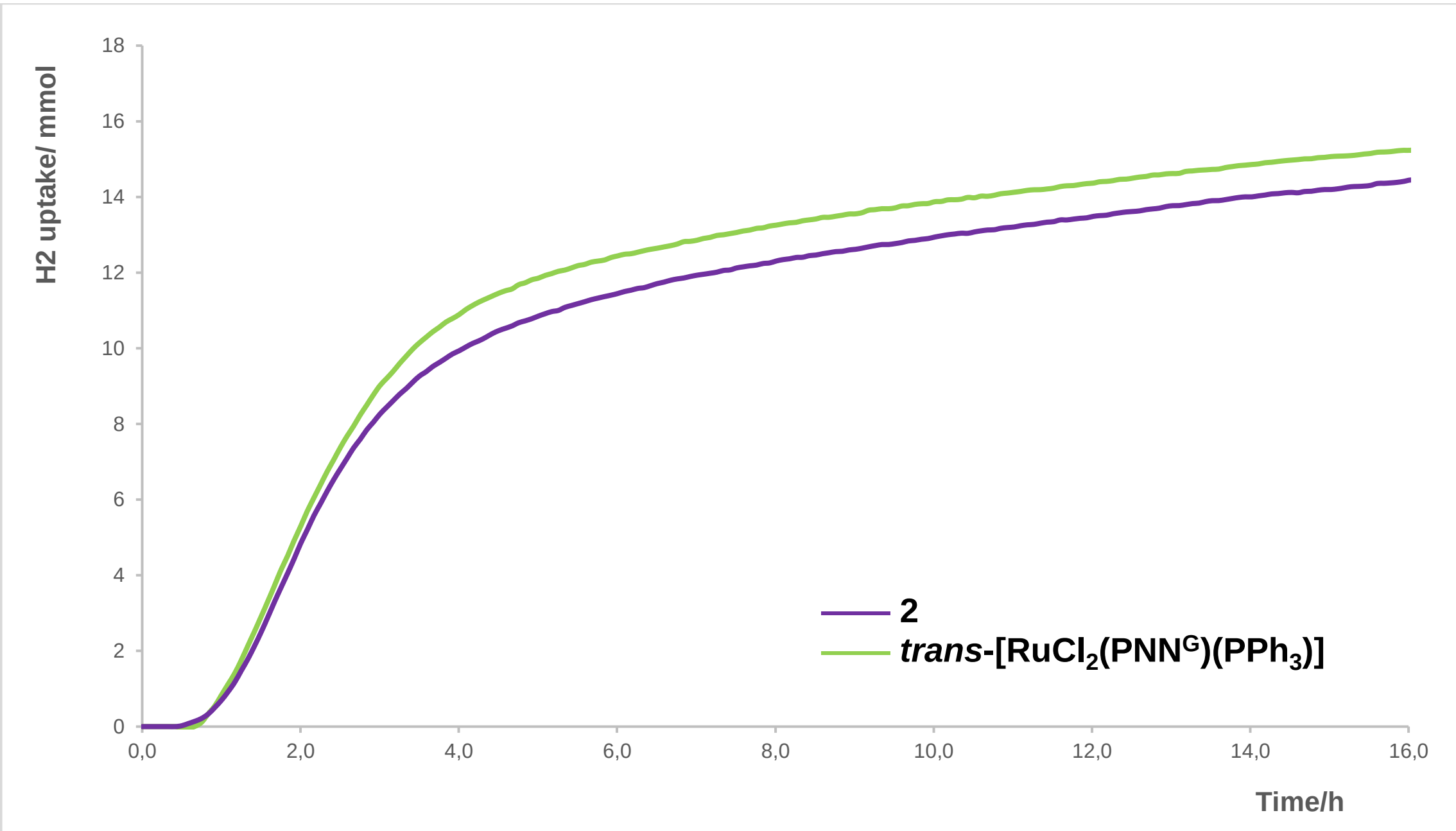

Figure S98. Hydrogen uptake curve of complex **2** and the *trans*-[RuCl$_2$(PNN^G)(PPh$_3$)] derivative for the solvent-free reduction of methyl benzoate **g** at 40 °C, 27.5 bar of H_2, 50 mol% NaOEt (S/C 50000).

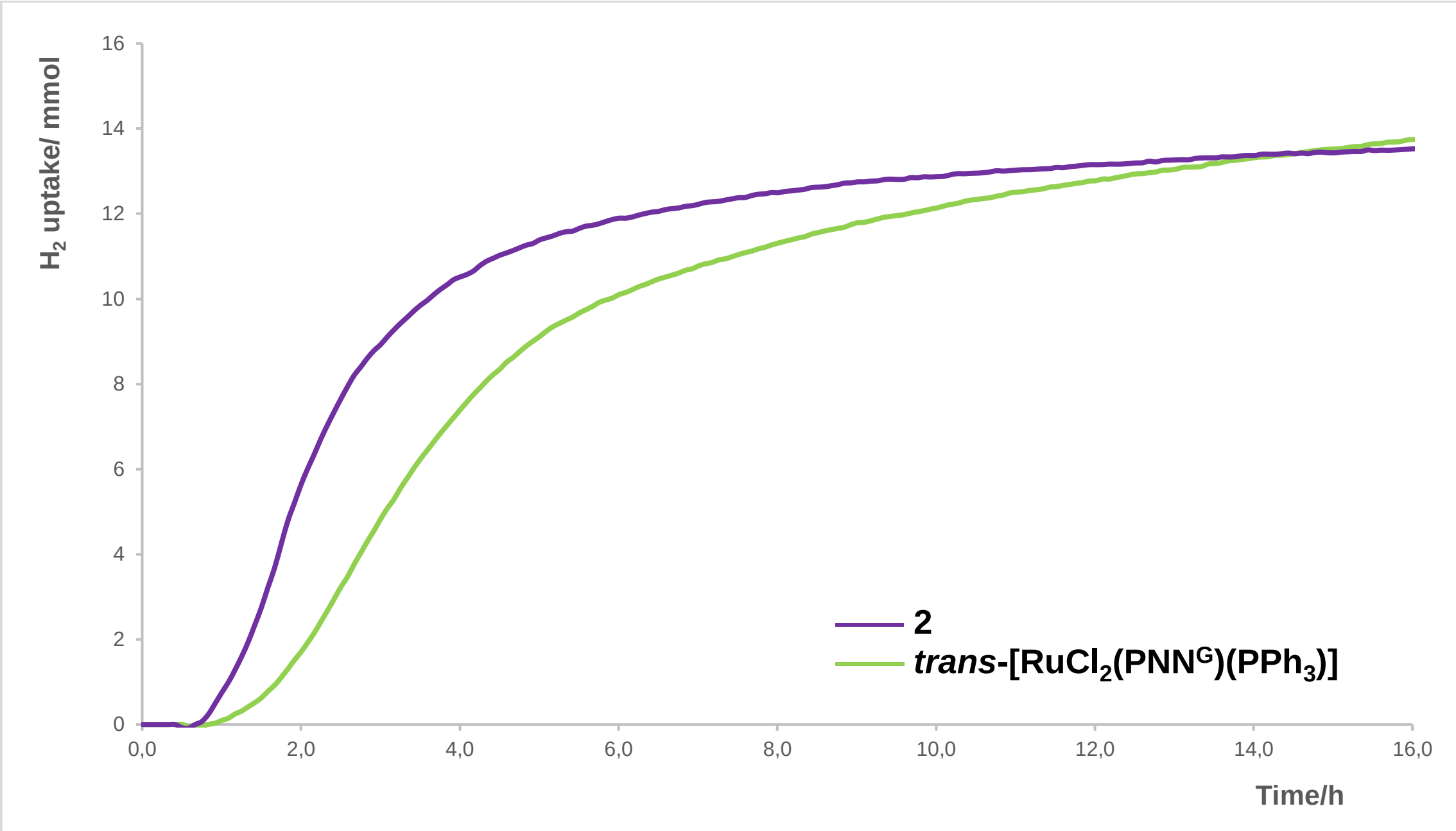

Figure S99. Hydrogen uptake curve of complex **2** and the *trans*-[RuCl$_2$(PNN^G)(PPh$_3$)] derivative for the solvent-free reduction of methyl benzoate **g** at 40 °C, 27.5 bar of H_2, 50 mol% NaOEt (S/C 80000).

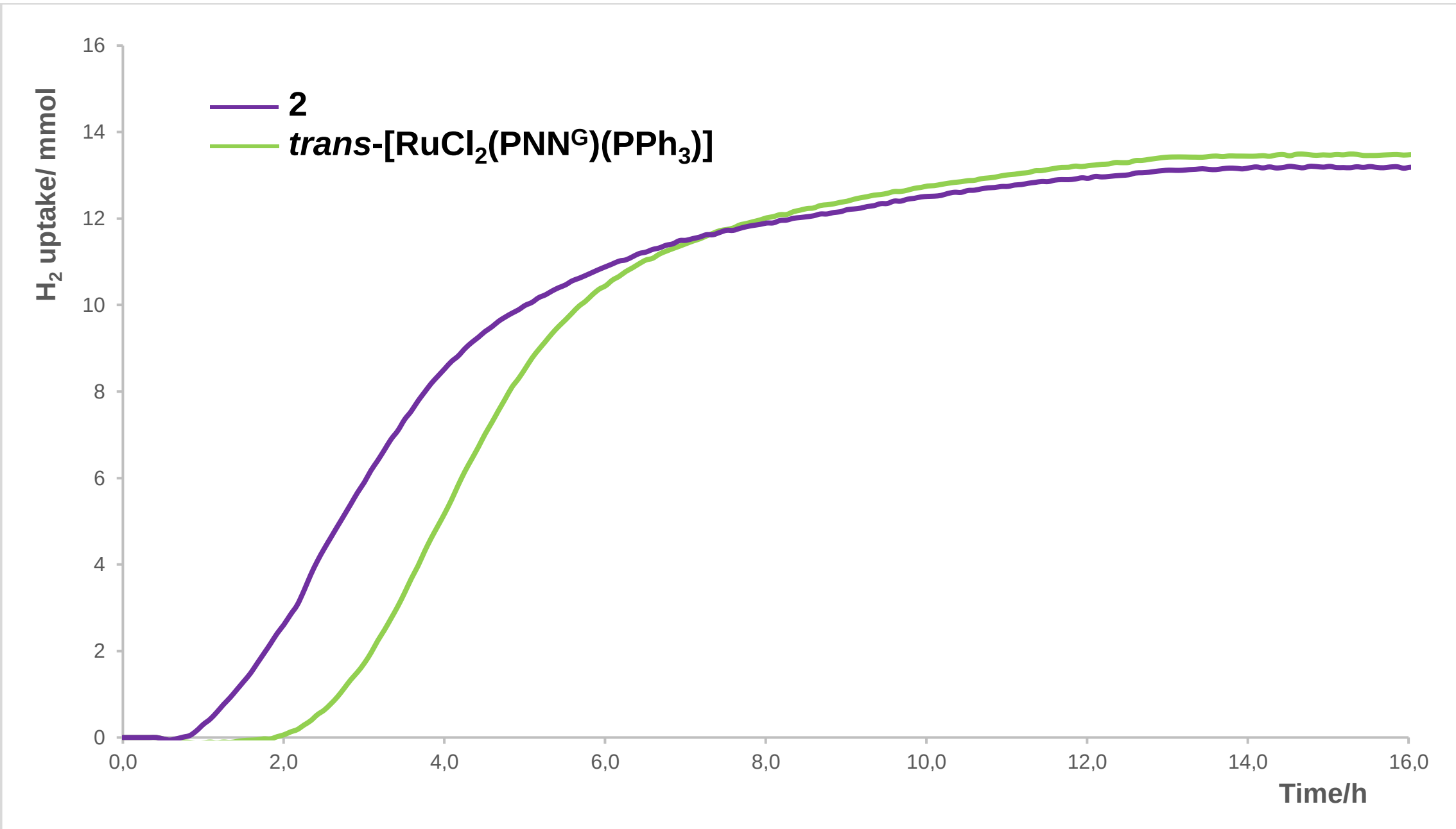

Figure S100. Hydrogen uptake curve of complex **2** and the *trans*-[RuCl$_2$(PNN^G)(PPh$_3$)] derivative for the solvent-free reduction of methyl benzoate **g** at 40 °C, 27.5 bar of H_2, 50 mol% NaOEt (S/C 100000).

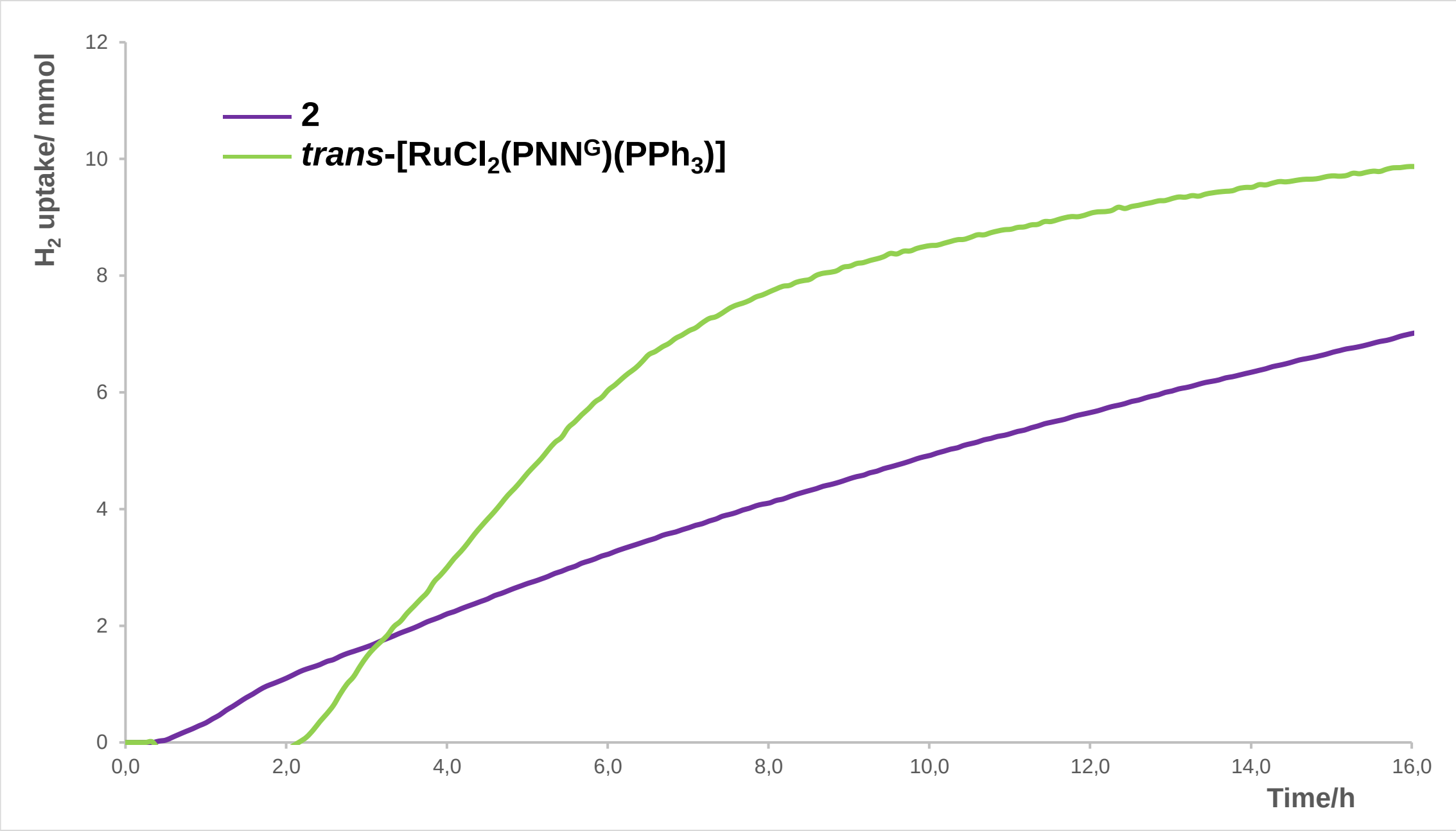

Figure S101. Hydrogen uptake curve of complex **2** and the *trans*-[RuCl$_2$(PNN^G)(PPh$_3$)] derivative for the solvent-free reduction of methyl benzoate **g** at 40 °C, 27.5 bar of H_2, 50 mol% NaOMe (S/C 100000).

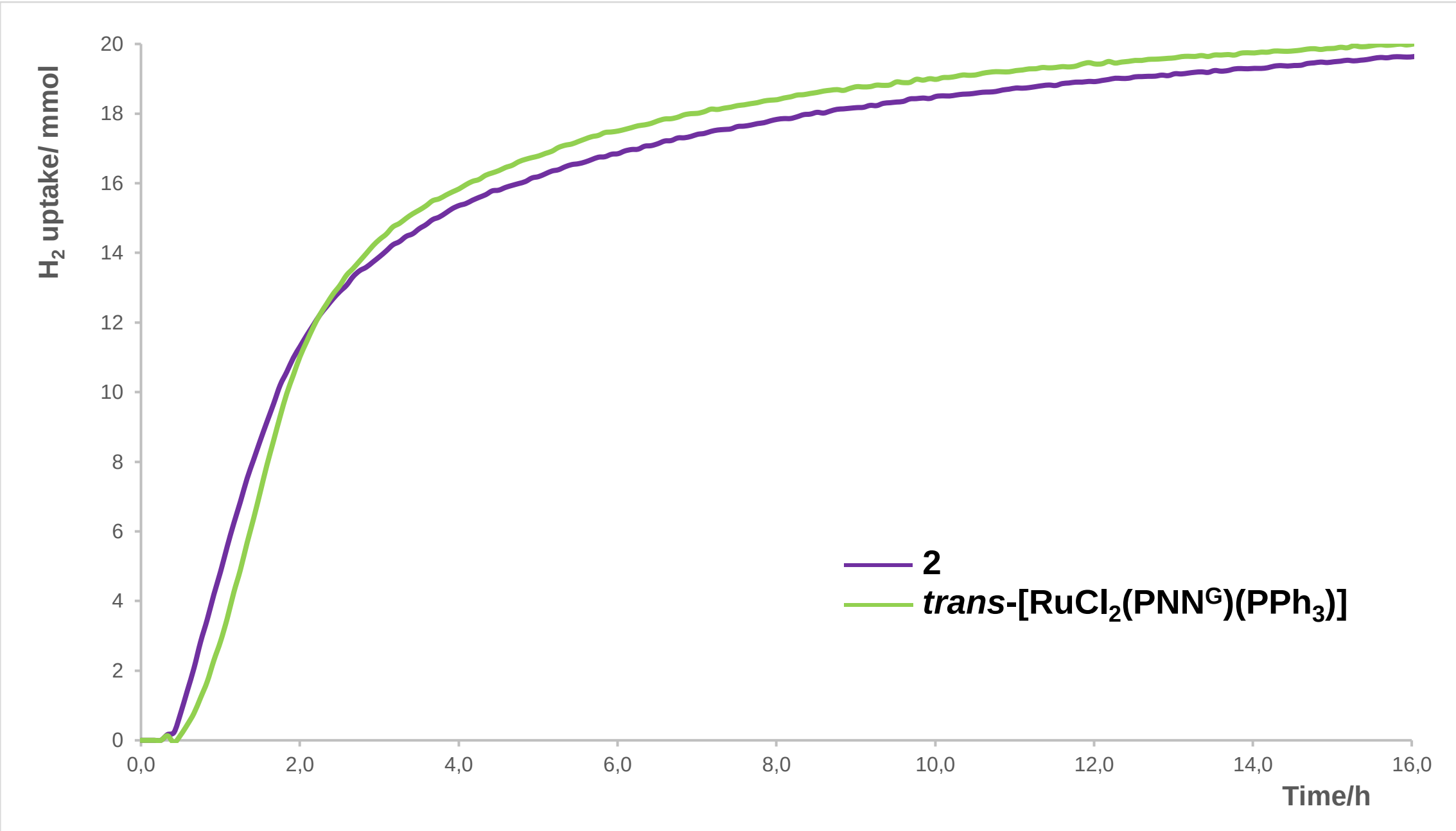

Figure S102. Hydrogen uptake curve of complex **2** and the *trans*-[RuCl$_2$(PNN^G)(PPh$_3$)] derivative for the solvent-free reduction of ethyl benzoate **h** at 40 °C, 27.5 bar of H_2, 50 mol% NaOEt (S/C 100000).

GC analytical method for methyl and ethyl decanoate hydrogenations

The crude reaction mixtures were quenched with HCl (1M in water) and extracted with MTBE before analysis using achiral gas chromatography to measure reaction conversions. The following analytical method was used.

Instrument: Varian GC 3900 system
Column: Agilent HP-88: 60 m x 0.25 mm x 0.2 µm
Column Oven Temperature Gradient: initial T = 115 °C for 1 min, ramped to 187 °C at 3 °C/min, then to 240 °C at 5 °C/min, which is maintained for another 1.4 min for a total of 37 min of analysis.
Carrier Gas: N_2 at 1.2 mL/min.
Detector: FID (T = 240 °C)
Sample diluent: MTBE
Injection volume: 5.0 µL

Retention time of identified compounds

Compound	Elution Time (min)
Methyl decanoate	8.09
Ethyl decanoate	8.80
1-decanol	10.41
Decyl decanoate	24.01

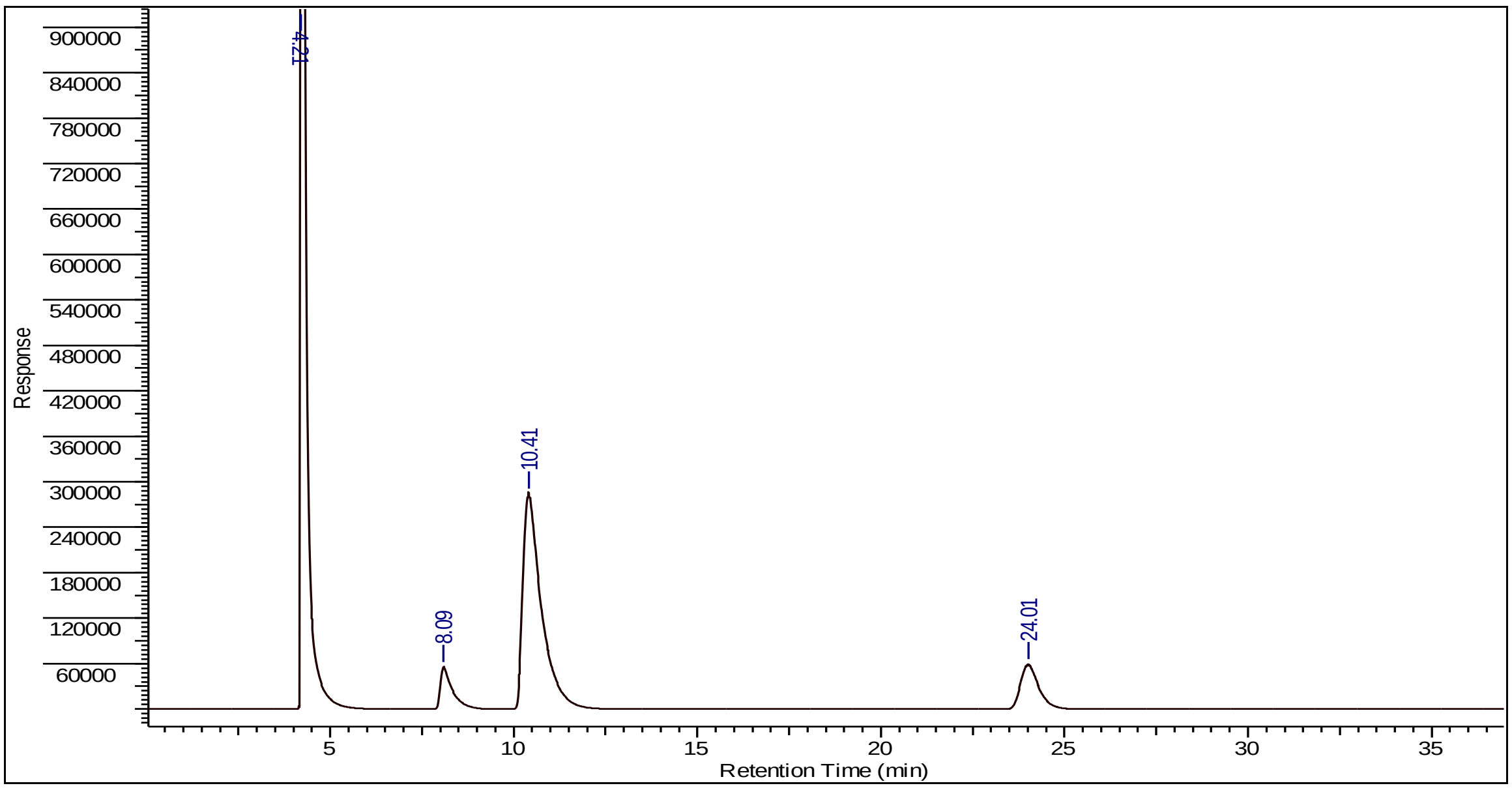

Figure S103. Representative example of GC chromatogram of the catalytic hydrogenation of methyl decanoate **a** using Ru PNN complexes containing all the species involved in the reaction (methyl decanoate, decyl decanoate, and 1-decanol product).

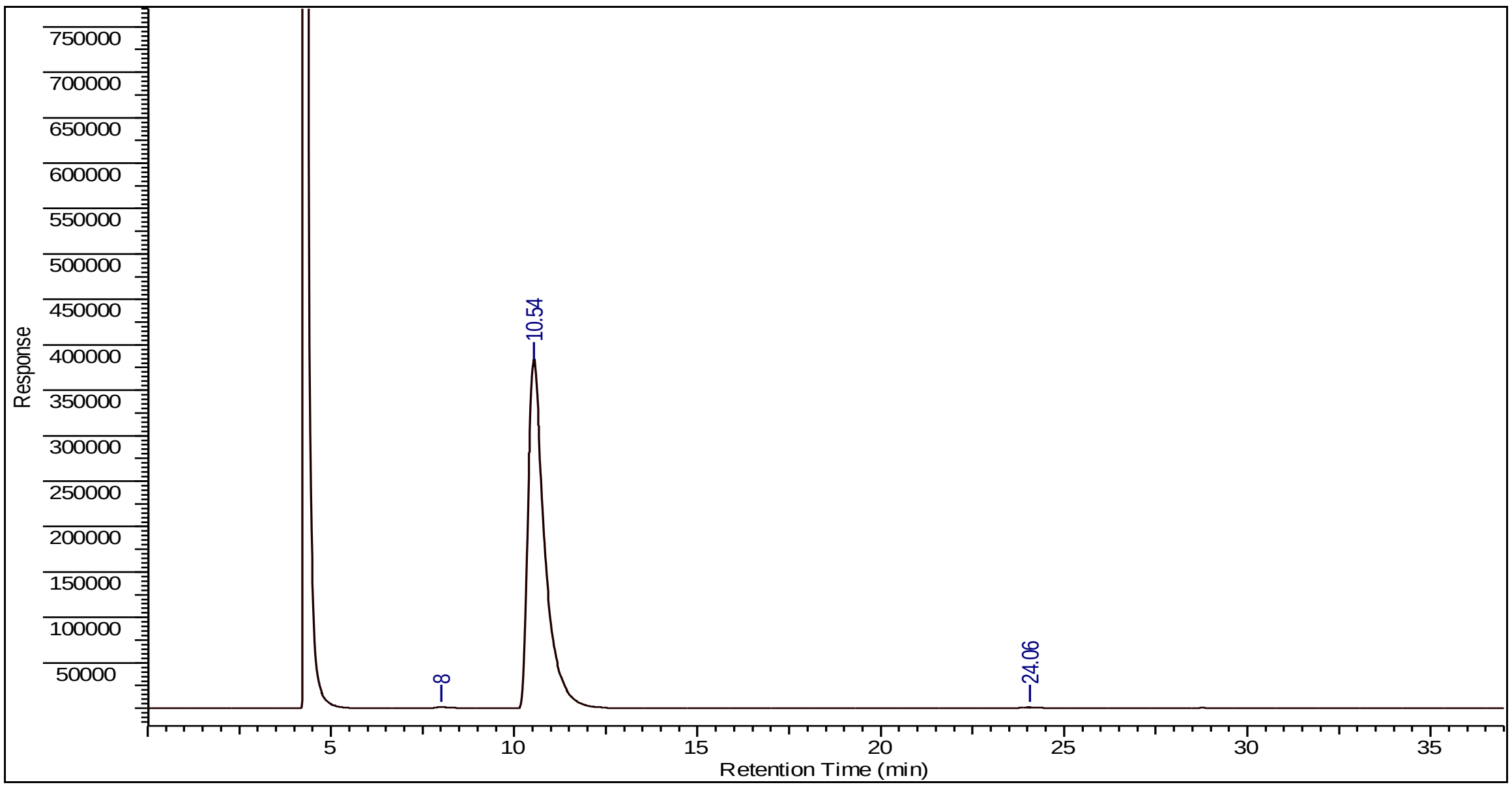

No.	Name	tR	Peak Area	Area Percent	Width
1	methyl decanoate	8.001	38070004.000	0.408	0.352
2	1-decanol	10.542	12307005254.000	99.377	0.887
3	decyl decanoate	24.067	33100005.000	0.215	0.243

Figure S104. GC traces of products of methyl decanoate **a** hydrogenation catalysed by complex **2** (S/C 10000) with 50 mol% NaOEt, 27.5 bar of H_2 and 40 °C, showing full conversion to 1-decanol.

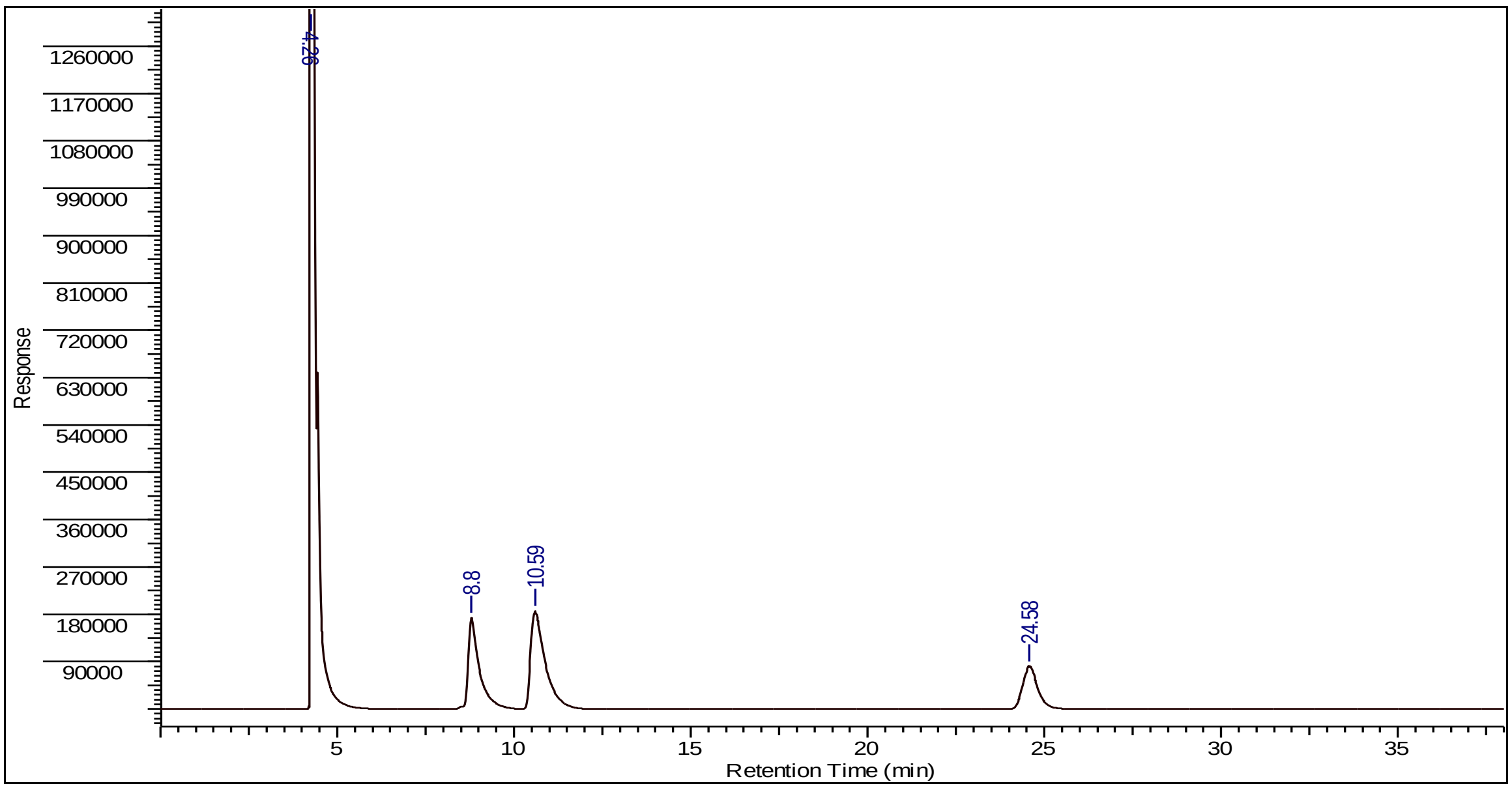

Figure S105. Representative example of GC chromatogram of the catalytic hydrogenation of ethyl decanoate **b** using Ru PNN complexes containing all the species involved in the reaction (ethyl decanoate, decyl decanoate, and 1-decanol product).

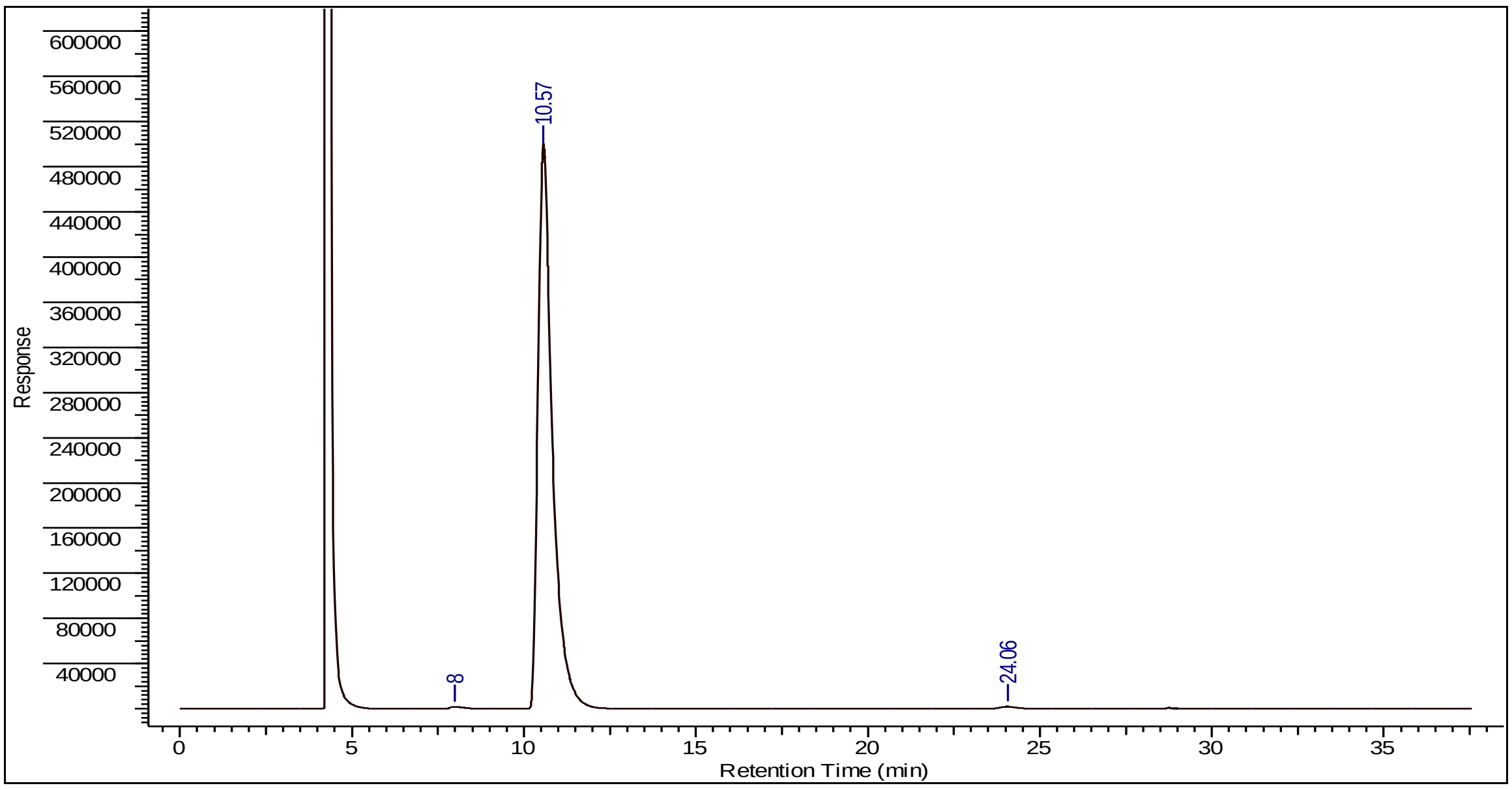

No.	Name	tR	Peak Area	Area Percent	Width
1	ethyl decanoate	8.002	33027759.000	0.234	0.324
2	1-decanol	10.572	1464557475.000	99.456	0.737
3	decyl decanoate	24.058	43134911.000	0.310	0.462

Figure S106. GC traces of products of ethyl decanoate **b** hydrogenation catalysed by complex **2** (S/C 50000) with 50 mol% NaOEt, 27.5 bar of H_2 and 40 °C, showing full conversion to 1-decanol.

GC analytical method for ethyl dodecanoate hydrogenations

The crude reaction mixtures were quenched with HCl (1M in water) and extracted with MTBE before analysis using achiral gas chromatography to measure reaction conversions. The following analytical method was used.

Instrument: Varian GC 3900 system
Column: Agilent HP-88: 60 m x 0.25 mm x 0.2 µm
Column Oven Temperature Gradient: initial T = 115 °C for 1 min, ramped to 187 °C at 3 °C/min, then to 240 °C at 5 °C/min, which is maintained for another 1.4 min for a total of 37 min of analysis.
Carrier Gas: N_2 at 1.2 mL/min.
Detector: FID (T = 240 °C)
Sample diluent: MTBE
Injection volume: 5.0 µL

Retention time of identified compounds

Compound	Elution Time (min)
Ethyl dodecanoate	8.50
1-dodecanol	11.91

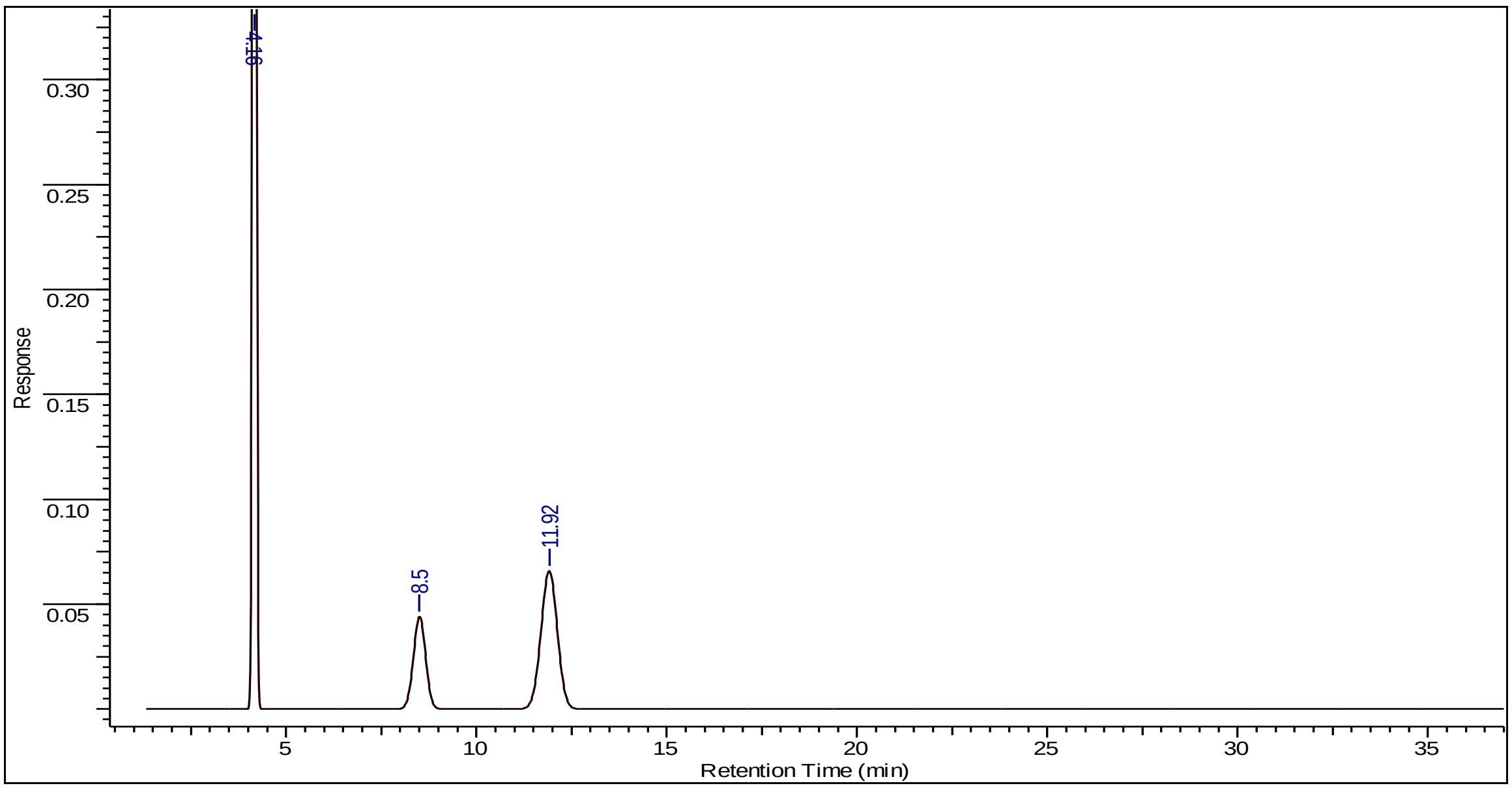

Figure S107. Representative example of GC chromatogram of the catalytic hydrogenation of ethyl dodecanoate **c** using Ru PNN complexes containing all the species involved in the reaction (ethyl dodecanoate and 1-dodecanol product).

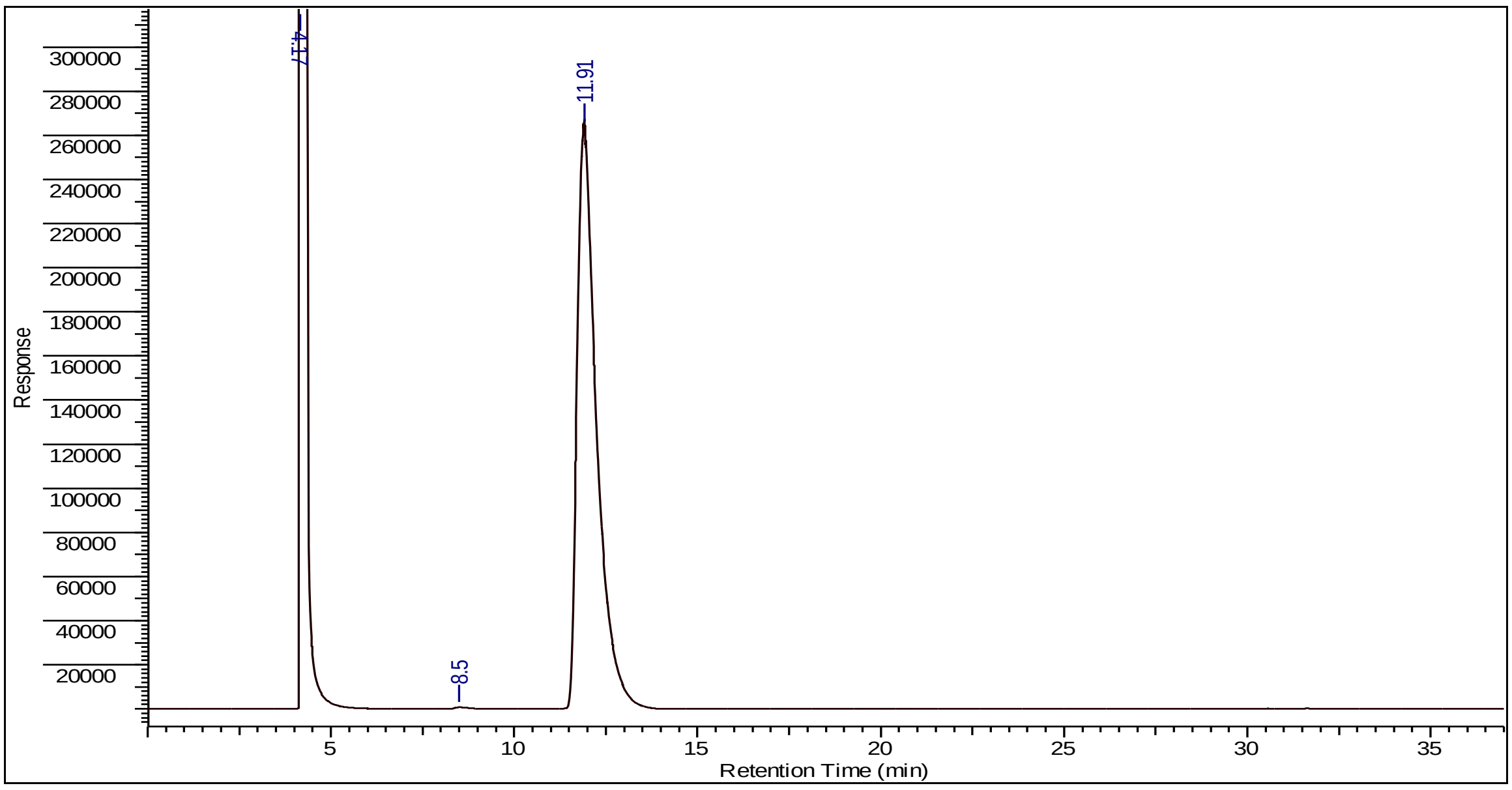

Figure S108. GC traces of products of ethyl dodecanoate **c** hydrogenation catalysed by complex **2** (S/C 44000) with 50 mol% NaOEt, 27.5 bar of H_2 and 40 °C, showing full conversion to 1-dodecanol.

GC analytical method for ethyl 10-undecenoate hydrogenations

The crude reaction mixtures were quenched with HCl (1M in water) and extracted with MTBE before analysis using achiral gas chromatography to measure reaction conversions. The following analytical method was used.

Instrument: Varian GC 3900 system

Column: Agilent HP-88: 60 m x 0.25 mm x 0.2 μm

Column Oven Temperature Gradient: initial T = 115 °C for 1 min, ramped to 187 °C at 3 °C/min, then to 240 °C at 5 °C/min, which is maintained for another 1.4 min for a total of 37 min of analysis.

Carrier Gas: N_2 at 1.2 mL/min.

Detector: FID (T = 240 °C)

Sample diluent: MTBE

Injection volume: 5.0 μL

Retention time of identified compounds

Compound	Elution Time (min)
Ethyl 10-undecenoate	11.01
1-undecanol	12.50
10-undecen-1-ol	14.37

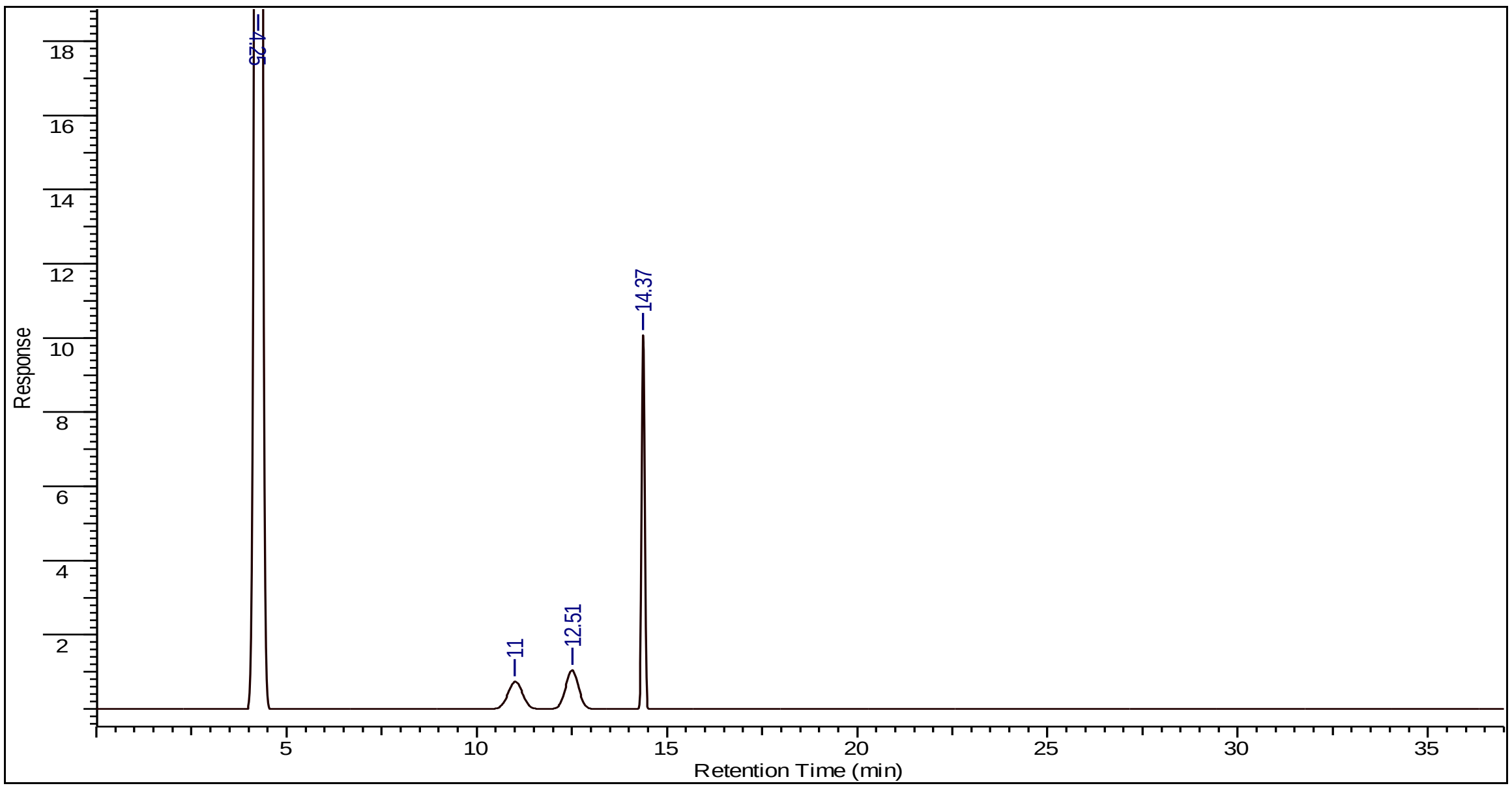

Figure S109. Representative example of GC chromatogram of the catalytic hydrogenation of ethyl 10-undecenoate **d** using Ru PNN complexes containing all the species involved in the reaction (ethyl 10-undecenoate, 1-undecanol and 10-undecen-1-ol).

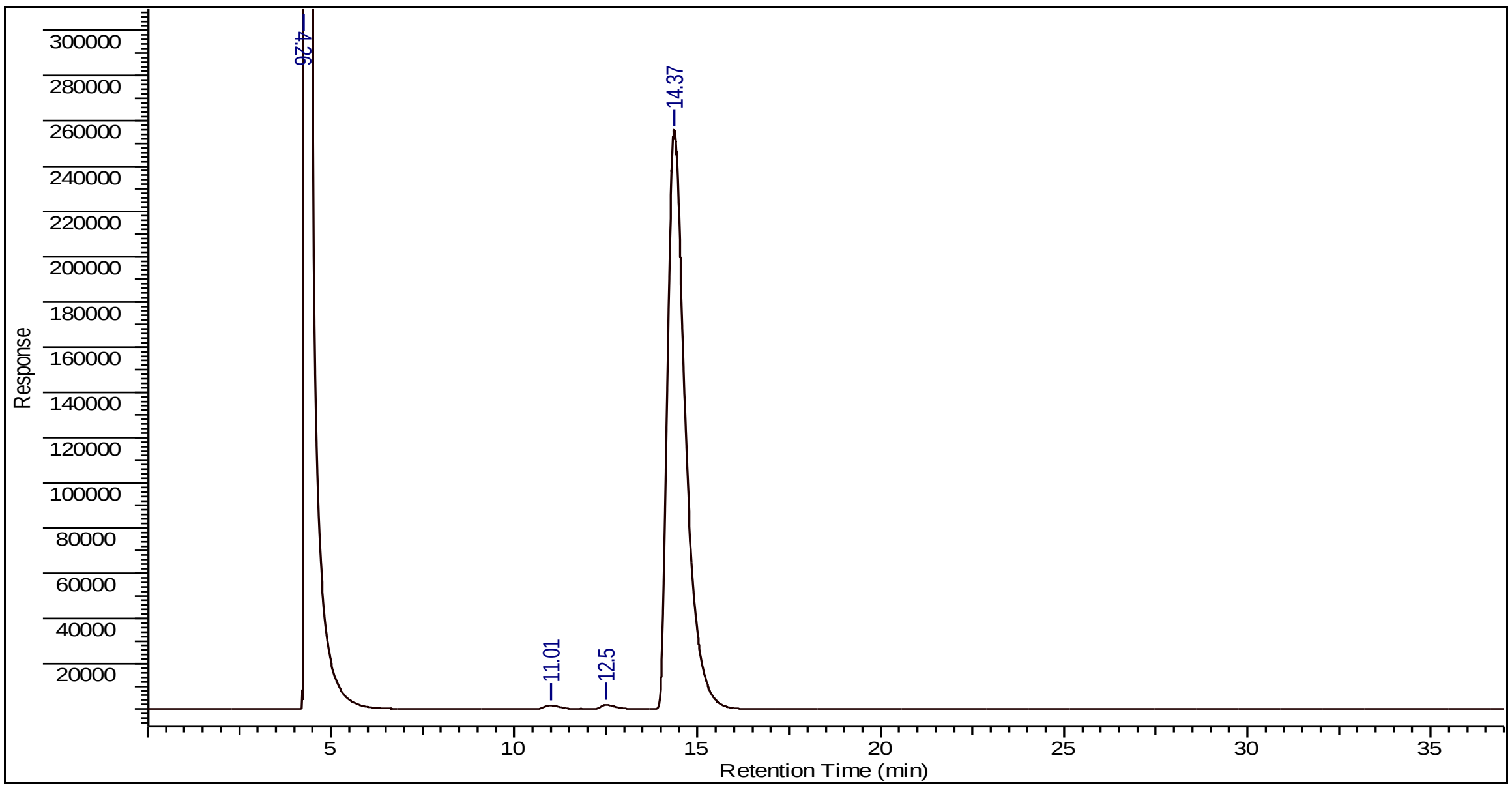

No.	Name	tR	Peak Area	Area Percent	Width
1	ethyl 10-undecenoate	11.011	44033855.000	0.507	0.520
2	1-undecanol	12.504	48669399.000	0.511	0.601
3	10-undecen-1-ol	14.373	8711475432.000	98.982	0.829

Figure S110. GC traces of products of ethyl 10-undecenoate **d** hydrogenation catalysed by complex **2** (S/C 10000) with 50 mol% NaOEt, 27.5 bar of H_2 and 40 °C, showing full conversion to 10-undecen-1-ol.

GC analytical method for methyl and ethyl oleate hydrogenations

The crude reaction mixtures were quenched with HCl (1M in water) and extracted with MTBE before analysis using achiral gas chromatography to measure reaction conversions. The following analytical method was used.

Instrument: Varian GC 3900 system

Column: Agilent HP-88: 60 m x 0.25 mm x 0.2 μm

Column Oven Temperature Gradient: initial T = 115 °C for 1 min, ramped to 187 °C at 3 °C/min, then to 240 °C at 5 °C/min, which is maintained for other 19.4 min for a total of 55 min of analysis.

Carrier Gas: N_2 at 1.2 mL/min.

Detector: FID (T = 240 °C)

Sample diluent: MTBE

Injection volume: 1.0 μL

Retention time of identified compounds

Compound	Elution Time (min)
Methyl Palmitate (C16:0)	19.66
Palmityl Alcohol (C16:0)	22.93
Methyl Stearate (C18:0)	21.15
Stearyl Alcohol (C18:0)	25.05
Methyl Oleate (C18:1)	25.17
Oleyl Alcohol (C18:1 Z)	28.19
Palmitoleyl Alcohol (C16:1 Z)	27.08
Methyl linoleate (C18:2)	26.69
Linoleyl Alcohol (C18:2)	29.54

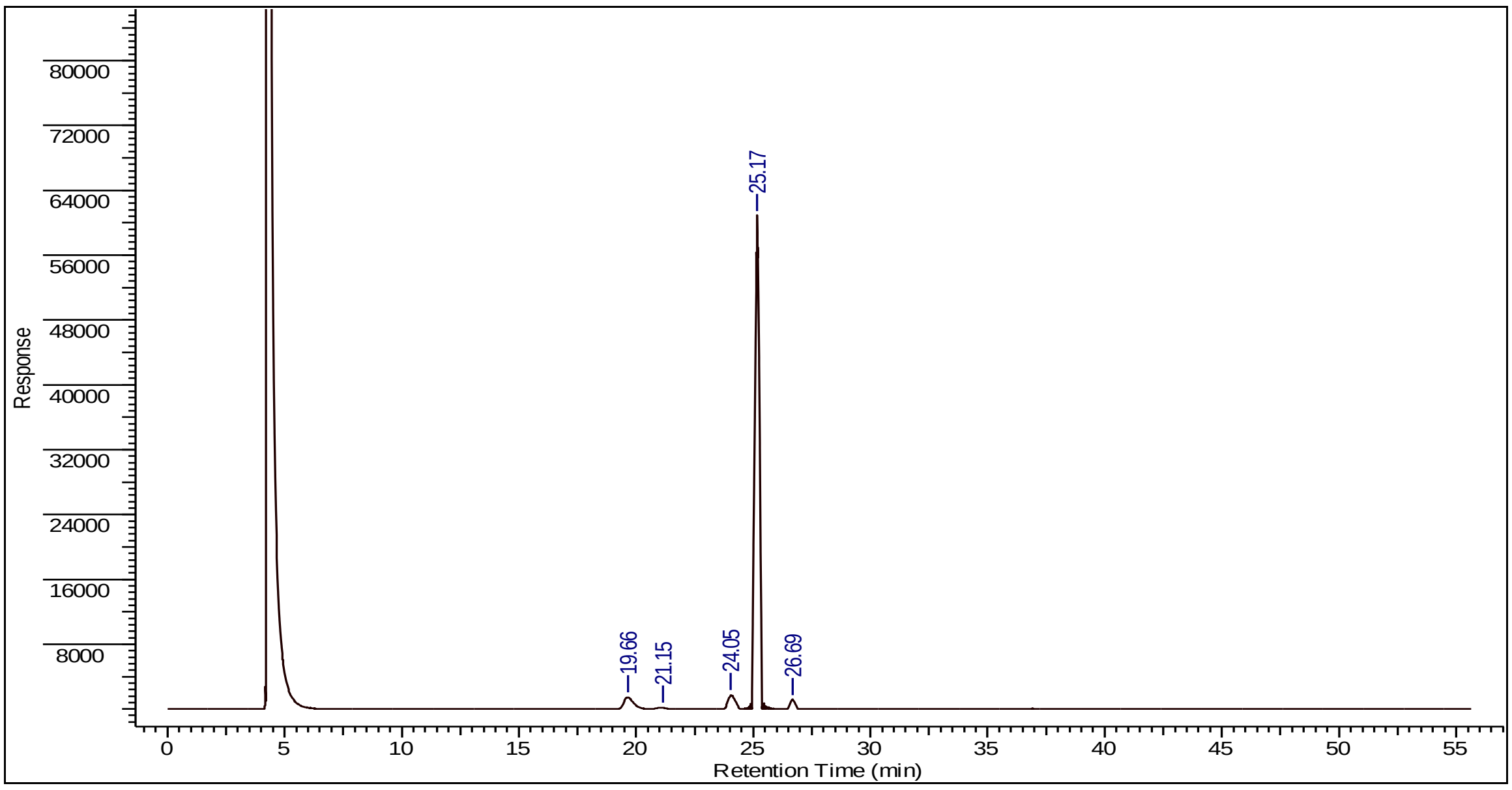

No.	Name	tR	Peak Area	Area Percent	Width
1	Methyl Palmitate (C16:0)	19.661	530326929.000	7.133	0.677
2	Methyl Stearate (C18:0)	21.154	667628.000	1.514	0.442
3	Methyl palmitoleate (C16:1)	24.052	124445364.00	4.211	0.417
4	Methyl Oleate (C18:1)	25.170	8433327520.000	84.112	0.843
5	Methyl linoleate (C18:2)	26.691	2326433678.000	3.030	0.633

Figure S111. Representative GC trace of starting material reference for technical grade methyl oleate **e**, showing composition (%) as a ratio of peak areas.

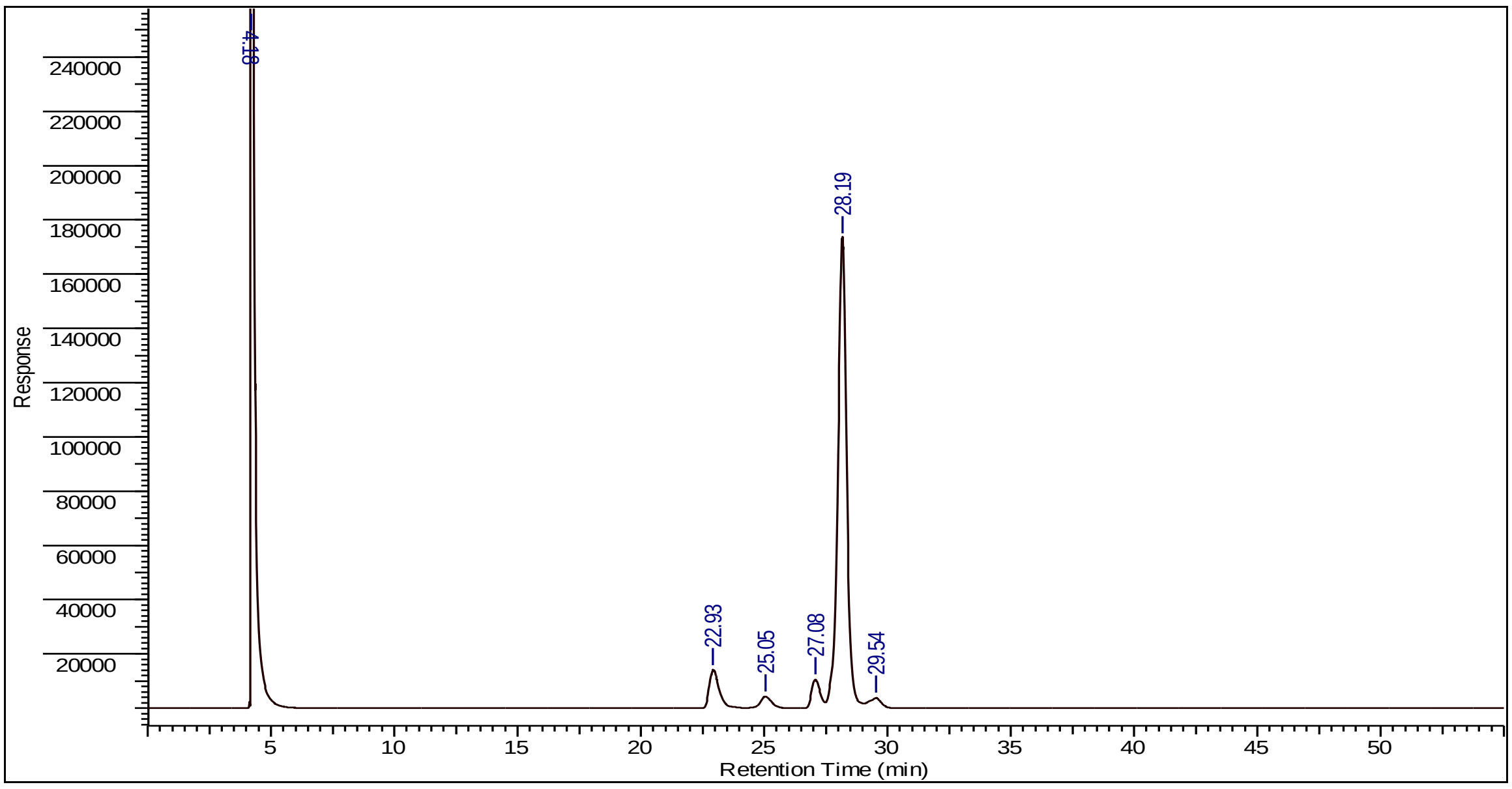

No.	Name	tR	Peak Area	Area Percent	Width
1	Palmityl Alcohol (C16:0)	22.933	394122920.0	7.488	0.650
2	Stearyl Alcohol (C18:0)	25.052	136931344.0	1.605	0.693
3	Palmitoleyl Alcohol (C16:1 (Z))	27.084	252523327.0	4.804	0.498
4	Oleyl Alcohol (C18:1 Z)	28.193	4306665880.0	83.333	0.660
5	Linoleyl Alcohol (C18:2)	29.541	153094477.0	2.770	0.933

Figure S112. Representative GC traces of hydrogenation products of methyl oleate **e** catalysed by complex **2** at 27.5 bar H_2 and 50 °C with 50 mol% NaOMe (S/C 25000).

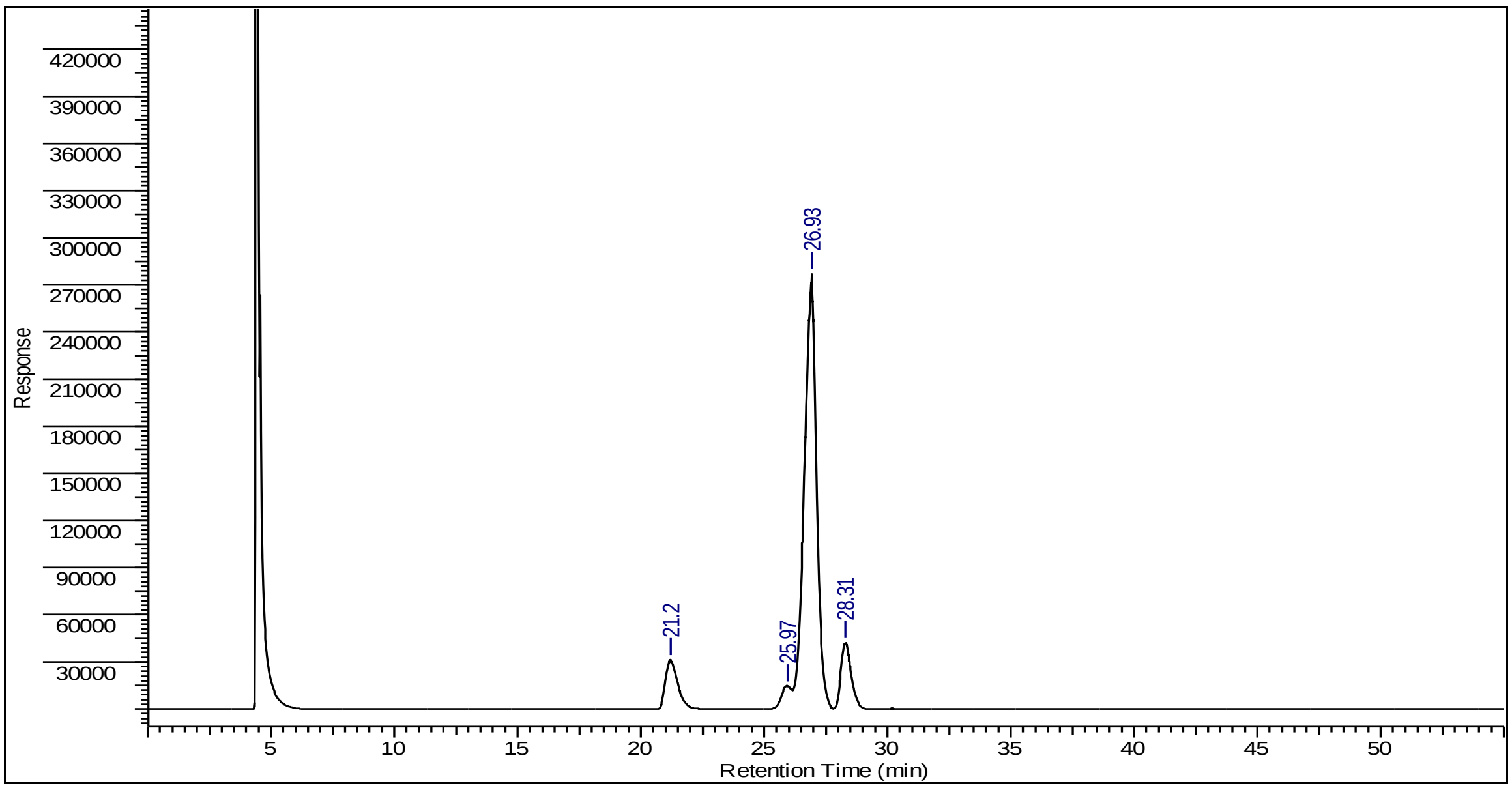

No.	Name	tR	Peak Area	Area Percent	Width
1	ethyl palmitate	21.200	1077972480.0	6.245	0.804
2	ethyl palmitoleate	25.965	387381376.0	1.179	0.870
3	ethyl oleate	26.933	9497743360.0	78.935	0.776
4	ethyl linoleate	28.306	1223611904.0	12.041	0.671

Figure S113. GC traces of starting material reference for technical grade ethyl oleate **f**, showing composition (%) as a ratio of peak areas.

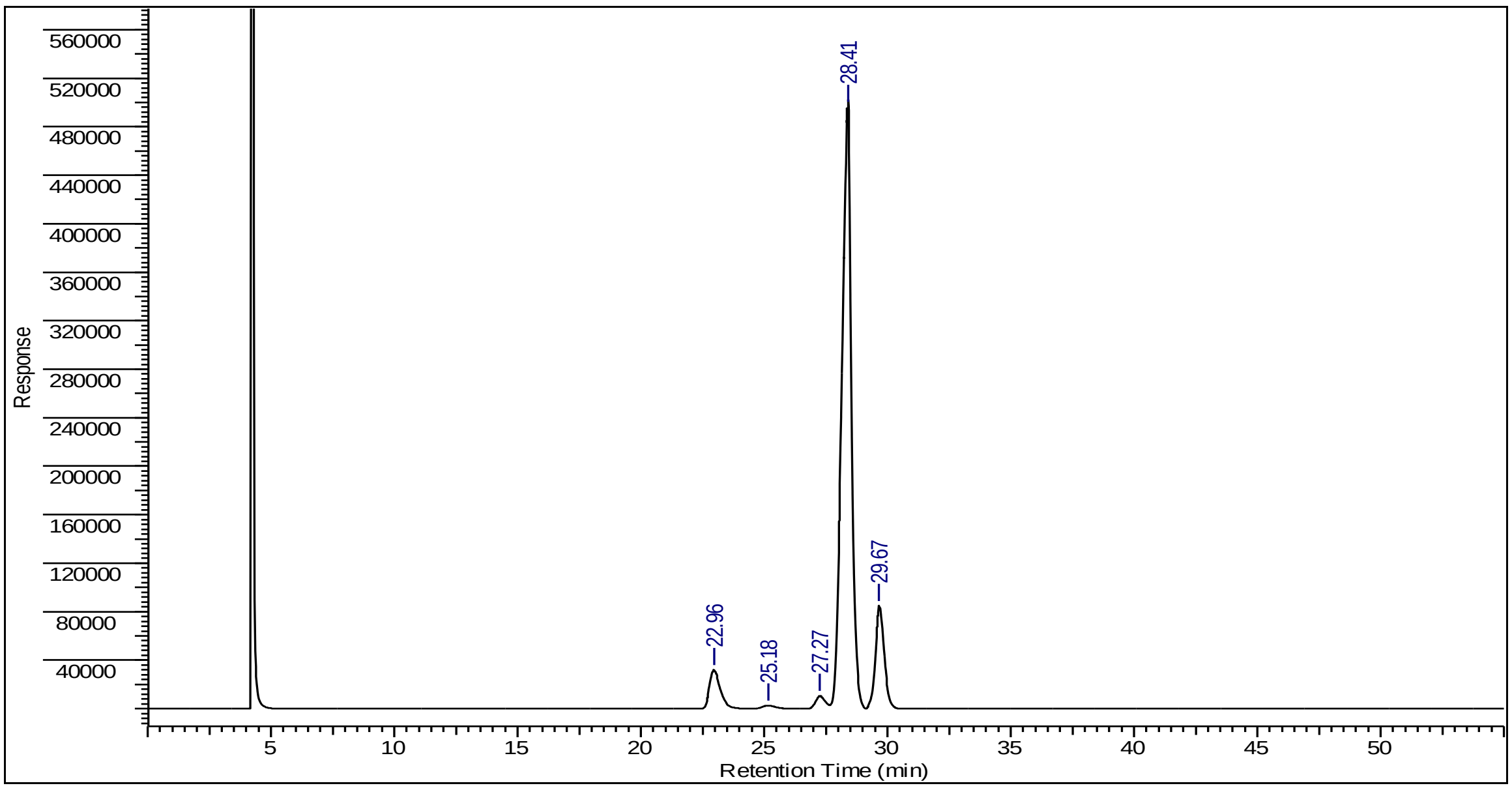

No.	Name	tR	Peak Area	Area Percent	Width
1	Palmityl alcohol (C16:0)	22.961	966001126.0	5.701	0.711
2	Stearyl alcohol (C18:0)	25.179	93811330.0	0.522	0.837
3	Palmitoleyl alcohol (C16:1 (Z))	27.266	249324599.0	1.493	0.665
4	Oleyl alcohol (C18:1 (Z))	28.414	15911775555.0	79.946	0.699
5	Linoleyl alcohol (C18:2 (Z))	29.673	2106273325.0	12.444	0.586

Figure S114. GC trace of hydrogenation products of ethyl oleate **f** catalyzed by complex **2** at 27.5 bar H_2 and 50 °C with 50 mol% NaOEt (S/C 50000).

GC analytical method for methyl and ethyl benzoate hydrogenations

The crude reaction mixtures were quenched with HCl (1M in water) and extracted with MTBE before analysis using achiral gas chromatography to measure reaction conversions. The following analytical method was used.

Instrument: Varian GC 3900 system

Column: Agilent HP-88: 60 m x 0.25 mm x 0.2 μm

Column Oven Temperature Gradient: initial T = 115 °C for 1 min, ramped to 187 °C at 3 °C/min, then to 240 °C at 5 °C/min, which is maintained for another 1.4 min for a total of 37 min of analysis.

Carrier Gas: N_2 at 1.2 mL/min.

Detector: FID (T = 240 °C)

Sample diluent: MTBE

Injection volume: 1.0 μL

Retention time of identified compounds

Compound	Elution Time (min)
Methyl benzoate	6.75
Ethyl benzoate	7.02
Benzyl alcohol	9.38
Benzyl benzoate	19.79

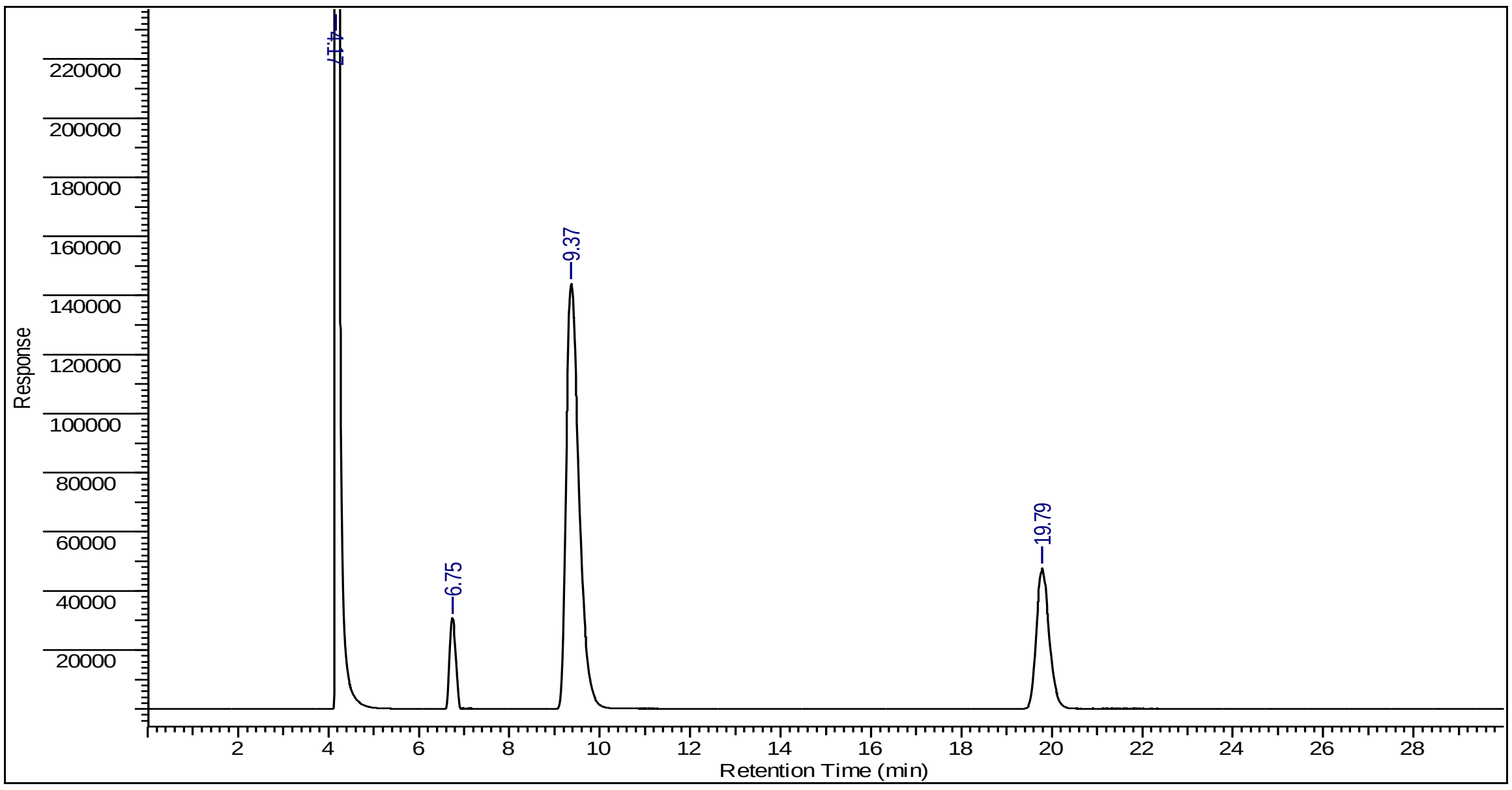

Figure S115. Representative example of GC chromatogram of the catalytic hydrogenation of methyl benzoate **g** using Ru PNN complexes containing all the species involved in the reaction (methyl benzoate, benzyl alcohol and benzyl benzoate).

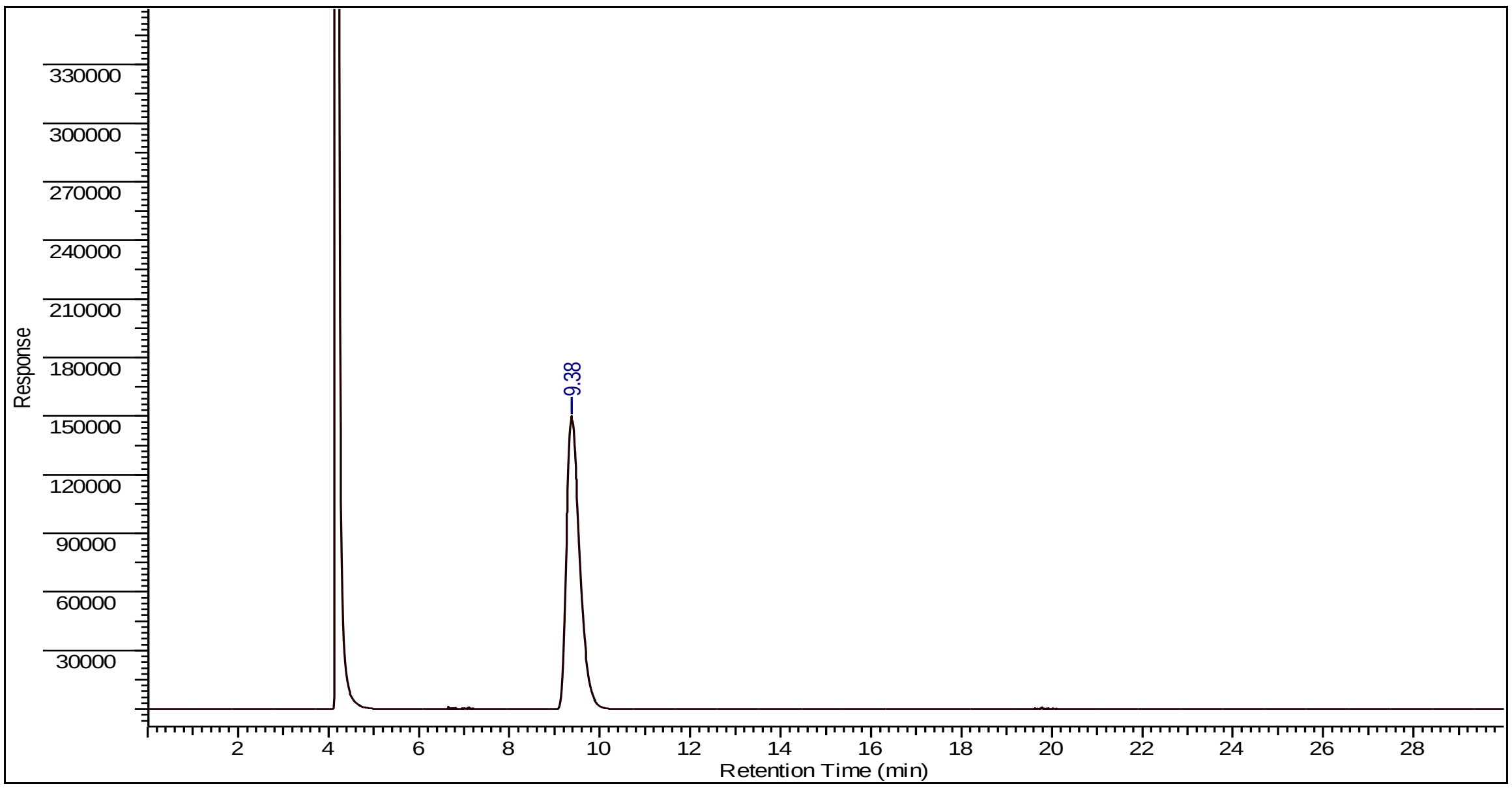

No.	Name	tR	Peak Area	Area Percent	Width
1	benzyl alcohol	9.383	3045447022.000	100.000	0.475

Figure S116. GC traces of products of methyl benzoate **g** hydrogenation catalysed by complex **2** (S/C 10000) with 50 mol% NaOEt, 27.5 bar of H_2 and 40 °C, showing full conversion to benzyl alcohol.

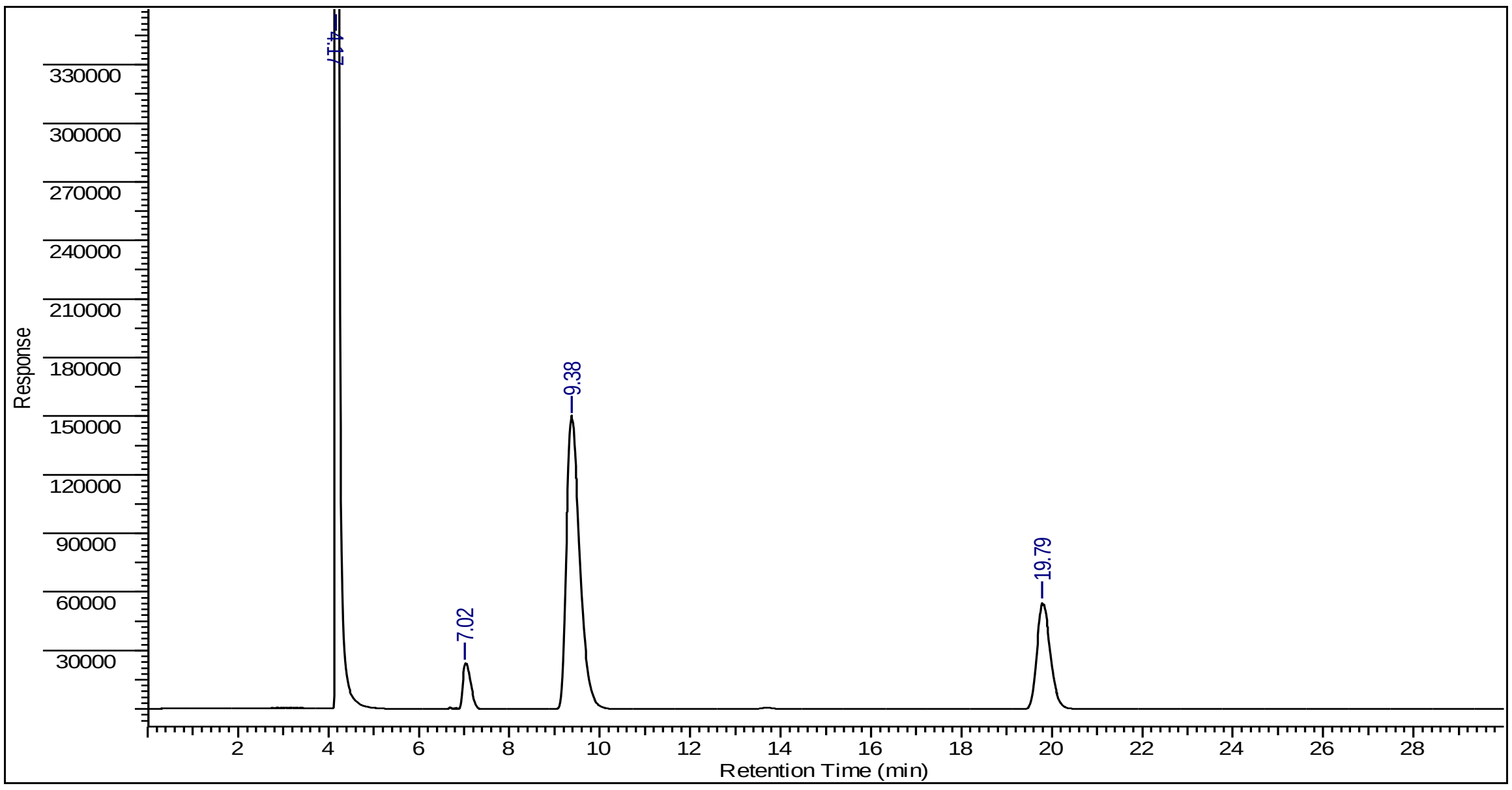

Figure S117. Representative example of GC chromatogram of the catalytic hydrogenation of ethyl benzoate **h** using Ru PNN complexes containing all the species involved in the reaction (ethyl benzoate, benzyl alcohol and benzyl benzoate).

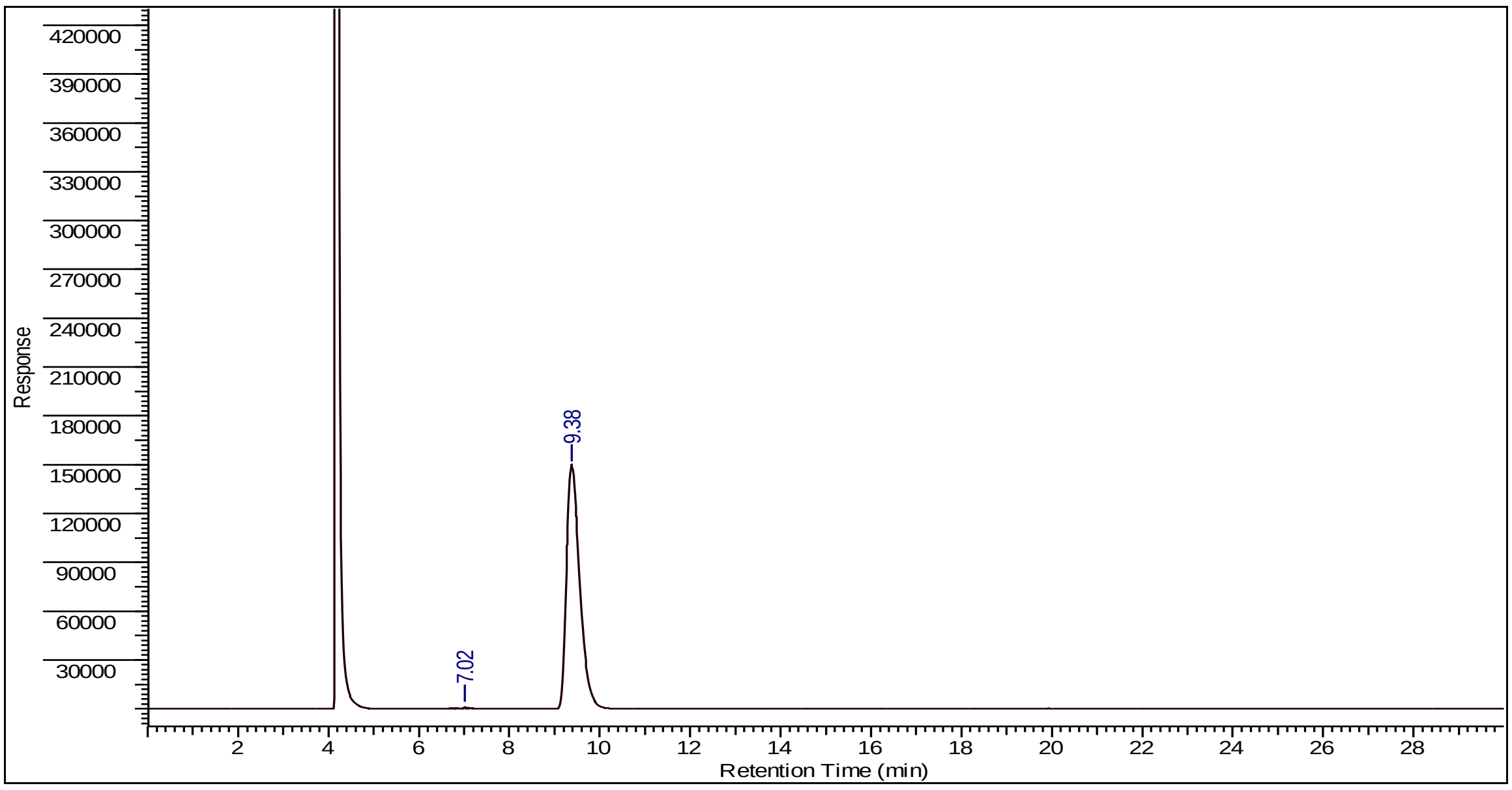

No.	Name	tR	Peak Area	Area Percent	Width
1	ethyl benzoate	7.024	4483884.000	0.143	0.058
2	benzyl alcohol	9.383	30607286108.000	99.857	0.477

Figure S118. GC trace of products of ethyl benzoate **h** hydrogenation catalysed by complex **2** (S/C 50000) with 50 mol% NaOEt, 27.5 bar of H_2 and 40 °C, showing full conversion to benzyl alcohol.

Single Crystal X-Ray Structure Determination of Compound 10 (CCDC 2553530)

General Data

The X-ray intensity data were collected at 100 K on a Bruker D8 VENTURE three-angle diffractometer with either a TXS rotating anode or with an IMS microsource with MoK_{α} radiation (λ = 0.71073 Å) using the APEX4 software.[72] The diffractometer was equipped with a Helios optic monochromator, a Bruker PHOTON II detector, and an Oxford Cryostream low temperature device. Measurements were performed on single crystals coated with perfluorinated ether. The crystals were fixed on top of a Kapton micro sampler and frozen under a stream of cold nitrogen. A matrix scan was used to determine the initial lattice parameters. All data were integrated with the Bruker SAINT V8.40B software package using a narrow-frame algorithm and the reflections were corrected for Lorentz and polarization effects, scan speed, and background.[73] Data were corrected for absorption effects including odd and even ordered spherical harmonics by the multi-scan method (SADABS 2016/2).[74, 75] Space group assignment was based upon systematic absences, E statistics, and successful refinement of the structure. The structure was solved by direct methods using SHELXT and refined by full-matrix least-squares methods against F^2 by minimizing $\Sigma w(F_o^2 - F_c^2)^2$ using SHELXL in conjunction with SHELXLE.[76-79] All non-hydrogen atoms were refined with anisotropic displacement parameters. Hydrogen atoms were refined isotropically on calculated positions using a riding model with their U_{iso} values constrained to 1.5 times the U_{eq} of their pivot atoms for terminal sp^3 carbon atoms and a C–H distance of 0.98 Å. Non-methyl hydrogen atoms were refined using a riding model with methylene, aromatic, and other C–H distances of 0.99 Å, 0.95 Å, and 1.00 Å, respectively, and U_{iso} values constrained to 1.2 times the U_{eq} of their pivot atoms. Molecular graphics was achieved using *PLATON* software that was used to prepare material for publication together with *enCIFer*.[80-82] Neutral atom scattering factors for all atoms and anomalous dispersion corrections for the non-hydrogen atoms were taken from International Tables for Crystallography.[83, 84] Supplementary crystallographic data reported in this paper have been deposited with the Cambridge Crystallographic Data Centre (CCDC 2553530) and can be obtained free of charge from The Cambridge Crystallographic Data Centre via www.ccdc.cam.ac.uk/structures.[85] This report and the CIF file were generated using FinalCif.[86]

Single Crystal X-Ray Structure Determination of Compound 10 (CCDC 2553530)

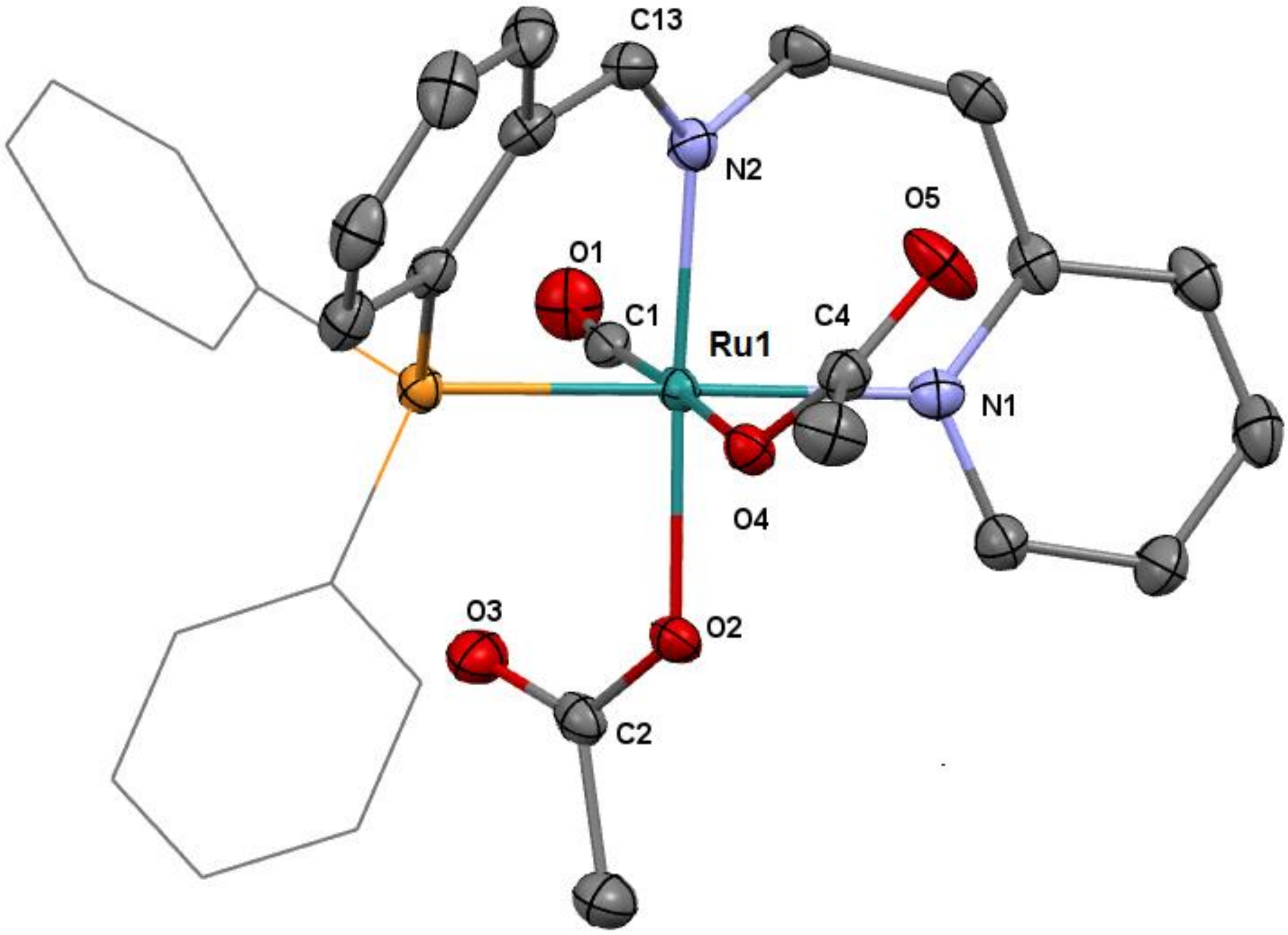

Figure S119. ORTEP representation of compound **10** in the solid state (CCDC 2553530). Ellipsoids are drawn at the 50% probability level. Hydrogen atoms and co-crystallized solvent molecules are omitted, and phenyl groups are simplified as wireframes for clarity. Selected bond lengths [Å] and angles [°]: Ru1–C1 1.833(4), Ru1–O2 2.069(2), Ru1–N1 2.191(3), Ru1–N2 2.054(3), Ru1–O4 2.119(2), Ru1–P1 2.2796(10), O1-C1 1.164(4), N2-C13 1.282(5), C1-Ru1-N2 89.9(2), N2-Ru1-O2 174.4(1), N2-Ru1-O4 91.4(1), C1-Ru1-N1 94.3(1), O2-Ru1-N1 87.9(1), C1-Ru1-P1 92.6(1), O2-Ru1-P1 92.00(8), N1-Ru1-P1 173.06(8), C1-Ru1-O2 95.6(1), C1-Ru1-O4 177.15(1), O2-Ru1-O4 82.6(1), N2-Ru1-N1 90.6(1), O4-Ru1-N1 87.9(1), N2-Ru1-P1 88.77(9), O4-Ru1-P1 85.20(8), C13-N2-Ru1 128.2(3), O1-C1-Ru1 174.6(3).

Single Crystal X-Ray Structure Determination of Compound 10 (CCDC 2553530)

Detailed Crystallographic Data.

Diffractometer operator:	A. Heidecker
Scanspeed	1-20 s per frame
dx	41 mm
Frames:	3269 measured in 12 data sets
phi-scans with delta phi	0.5
omega-scans with delta omega	0.5
shutterless mode	

Crystal Data:

[$C_{31}H_{29}N_2O_5PRu$]	ρ_{calc} = 1.417 g cm^{-3}
M_r = 641.63	Mo*K*α radiation, λ = 0.71073 Å
Triclinic	Cell parameters from 9361 reflections
space group: P -1	θ = 2.30–25.40°
a = 10.299(3) Å	μ = 0.615 mm^{-1}
b = 11.688(3) Å	*T* = 100 K
c = 13.588 (4) Å	Clear yellow block
V = 1503.5(7) Å^3	0.099 × 0.109 × 0.161 mm^3
α = 82.358(10)°	*Z* = 2
β = 81.861(10)°	*F*(000) = 656
γ = 68.799(10)°	

Data collection:

Bruker D8 Venture diffractometer	5540 independent reflections
Radiation source: IMS microsource	5102 reflections with $I > 2\sigma(I)$
Coverage of Independent reflections	99.9%
Helios optic monochromator	R_{int} = 0.0567
	θ_{max} = 25.50°, θ_{min} = 2.13°
phi– and ω–rotation scans	$h = -12$ 12
Absorption correction: multi-scan *SADABS* 2016/2, Bruker	$k = -14$ 14
T_{min} = 0.664, T_{max} = 0.745	$l = -16$ 16
56294 measured reflections	

Data refinement:

Refinement method	Full-matrix least-squares on F2
Refinement program	SHELXL-2019/3 (Sheldrick, 2019)
Structure solution technique	direct methods
Structure solution program	SHELXT 2019/2 (Sheldrick, 2019)
Function minimized	$\Sigma w(Fo^2 - Fc^2)^2$
Data / restraints / parameters	5540 / 0 / 363
Final R indices	R1 = 0.0422, wR2 = 0.0955
	all data R1 = 0.0471, wR2 = 0.0977
Weighting scheme	$w = 1/[\sigma^2(Fo^2) + (0.0302P)^2 + 4.8507P]$ where $P = (Fo^2 + 2Fc^2)/3$
Goodness-of-fit on F^2	1.136
Largest diff. peak and hole	0.995 and -0.739 eÅ^{-3}
R.M.S. deviation from mean	0.092 eÅ^{-3}

References

[69] R. W. Mitchell, A. Spencer, G. Wilkinson, Carboxylato-triphenylphosphine complexes of ruthenium, cationic triphenylphosphine complexes derived from them, and their behaviour as homogeneous hydrogenation catalysts for alkenes. *J. Chem. Soc., Dalton Trans.* **1973**, 846-854. DOI: 10.1039/DT9730000846.

[70] R. Young, G. Wilkinson, S. Komlya, A. Yamamoto, (Acetato)Hydridotris(Triphenylphosphine)-Ruthenium(II) in *Inorg. Synth., Vol. 17*, McGraw-Hill, Inc. , **1977**, ch. 23, pp. 79-80. ISBN: 9780470132487.

[71] A. Spencer, G. Wilkinson, Reactions of μ_3-oxo-Triruthenium Carboxylates with p-acid Ligands. *J. Chem. Soc., Dalton Trans.* **1974**, 786-792. DOI: 10.1039/DT9740000786.

[72] *APEX4 Suite of Crystallographic Software, Version 2022-10.0,* Bruker AXS Inc., Madison, Wisconsin, USA, **2022**.

[73] Bruker, *SAINT*, *V8.40B*, Bruker AXS Inc., Madison, Wisconsin, USA, **2019**.

[74] L. Krause, R. Herbst-Irmer, G. M. Sheldrick, D. Stalke, *J. Appl. Cryst.* **2015**, *48*, 3-10, doi:10.1107/S1600576714022985.

[75] *SADABS*, Version 2016/2. Bruker AXS Inc., Madison, Wisconsin, USA, **2016**.

[76] G. M. Sheldrick, *Acta Cryst.* **2015**, *A71*, 3-8, doi:10.1107/S2053273314026370.

[77] G. M. Sheldrick, *Acta Cryst.* **2015**, *C71*, 3-8, doi:10.1107/S2053229614024218.

[78] C. B. Huebschle, G. M. Sheldrick, B. Dittrich, *J. Appl. Cryst.* **2011**, *44*, 1281-1284, doi:10.1107/S0021889811043202.

[79] H. D. Flack (1983). *Acta Cryst.* **1983**, A39, 876-881, doi:10.1107/S0108767383001762.

[80] A. L. Spek. *PLATON*, A Multipurpose Crystallographic Tool, Utrecht University, Utrecht, The Netherlands, **2011**.

[81] A. L. Spek, *J. Appl. Cryst.* **2003**, *36*, 7-13, doi:10. 1107/S0021889802022112.

[82] F. H. Allen, enCIFer: a program for viewing, editing and visualizing CIFs, *J. Appl. Cryst.* **2004**, *37*, 335-338, doi:10.1107/S0021889804003528.

[83] Ed. E. Prince, *International Tables for Crystallography Volume C, Mathematical, Physical and Chemical Tables*, International Union of Crystallography, Chester, England, **2006**, 500-502; 219-222; 193-199.

[84] A. J. C. Wilson, International Tables for Crystallography, Volume C, Kluwer Academic Publishers, Dordrecht, The Netherlands, **1992**.

[85] C. R. Groom, I. J. Bruno, M. P. Lightfoot, S. C. Ward, *Acta Cryst.* **2016**, *B72*, 171-179, doi:10.1107/S2052520616003954.

[86] D. Kratzert, *FinalCif*, *V153*, https://dkratzert.de/finalcif.html.
